# Transcriptome changes in grapevine (*Vitis vinifera *L.) cv. Malbec leaves induced by ultraviolet-B radiation

**DOI:** 10.1186/1471-2229-10-224

**Published:** 2010-10-20

**Authors:** Mariela A Pontin, Patricia N Piccoli, Rita Francisco, Ruben Bottini, Jose M Martinez-Zapater, Diego Lijavetzky

**Affiliations:** 1 Instituto de Biología Agrícola de Mendoza, Facultad de Ciencias Agrarias, Consejo Nacional de Investigaciones Científicas y Tecnológicas-Universidad Nacional de Cuyo, Almirante Brown 500, M5528AHB Chacras de Coria, Argentina; 2 Estación Experimental Agropecuaria La Consulta INTA, cc8 (5567) La Consulta, San Carlos, Mendoza, Argentina; 3 Plant Molecular Ecophysiology Laboratory, Instituto de Tecnologia Química e Biológica, Oeiras, Portugal; 4 Departamento de Genética Molecular de Plantas, Centro Nacional de Biotecnología, Consejo Superior de Investigaciones Científicas (CSIC), C/Darwin 3, 28049 Madrid, España; 5 Instituto de Ciencias de la Vid y del Vino (Consejo Superior de Investigaciones Científicas, Universidad de La Rioja, Gobierno de La Rioja), CCT, Campus de la Universidad de La Rioja, C/Madre de Dios 51, 26006 Logroño, España

## Abstract

**Background:**

Ultraviolet-B radiation (UV-B, 280-315 nm) is a natural component of sunlight, which has numerous regulatory effects on plant physiology. The nature of the response to UV-B is dependent on fluence rate, dose, duration and wavelength of the UV-B treatment. Some reports have analyzed the changes in gene expression caused by UV-B light on several plant species using microarray technology. However, there is no information on the transcriptome response triggered by UV-B in grapevine. In this paper we investigate the gene expression responses of leaves from *in vitro *cultured *Vitis vinifera *cv. Malbec plants subjected to the same dose of biologically effective UV-B radiation (4.75 kJ m^-2 ^d^-1^) administered at two different fluence rates (16 h at ≅ 8.25 μW cm^-2^, 4 h at ≅ 33 μW cm^-2^) using a new custom made GrapeGen Affymetrix GeneChip^®^.

**Results:**

The number of genes modulated by high fluence rate UV-B doubled the number of genes modulated by low fluence UV-B. Their functional analyses revealed several functional categories commonly regulated by both UV-B treatments as well as categories more specifically modulated depending on UV-B fluence rate. General protective responses, namely the induction of pathways regulating synthesis of UV-B absorbing compounds such as the Phenylpropanoid pathway, the induction of different antioxidant defense systems and the activation of pathways commonly associated with pathogen defense and abiotic stress responses seem to play critical roles in grapevine responses against UV-B radiation. Furthermore, high fluence rate UV-B seemed to specifically modulate additional pathways and processes in order to protect grapevine plantlets against UV-B-induced oxidative stress, stop the cell cycle progression, and control protein degradation. On the other hand, low fluence rate UV-B regulated the expression of specific responses in the metabolism of auxin and abscisic acid as well as in the modification of cell walls that could be involved in UV-B acclimation-like processes.

**Conclusion:**

Our results show the UV-B radiation effects on the leaf transcriptome of grapevine (*Vitis vinifera *cv. Malbec) plantlets. Functional categories commonly modulated under both UV-B treatments as well as transcripts specifically regulated in an UV-B-intensity dependent way were identified. While high fluence rate UV-B had regulatory effects mainly on defense or general multiple-stress responses pathways, low fluence rate UV-B promoted the expression of genes that could be involved in UV-B protection or the amelioration of the UV-B-induced damage. This study also provides an extensive list of genes regulating multiple metabolic pathways involved in the response of grapevine to UV-B that can be used for future researches.

## Background

Ultraviolet-B radiation (UV-B, wavelength range 280 to 315 nm) is a natural component of solar radiation. Most of the UV-B solar radiation is absorbed by the stratospheric ozone layer and other atmospheric gases and therefore only a minor proportion reaches the Earth's surface. The level of UV-B is dependent on several factors such as latitude, season, time of day, cloud cover, and altitude [1].

The effects of UV-B have been analyzed on diverse plants species and vary depending on UV-B fluence rates, duration and wavelength of the UV-B treatment [2-7]. Exposure to UV-B amounts much higher than those found in nature causes tissue necrosis and induces the expression of many genes normally involved in defense, wounding, or general stress responses. That is, several studies have reported damage to DNA, proteins and membranes and the inhibition of protein synthesis and photosynthetic reactions [4,8,9].

Ultraviolet-B radiation is not necessarily a damage-inducing source of stress but instead can act as an important environmental cue in higher plants, regulating several key developmental plant responses. At ambient UV-B levels, crosstalk between wounding and UV-B signaling pathways seem to modify plant-insect interactions [10]. Moreover, exposure to such low non-damaging levels of UV-B has numerous regulatory effects on plant morphology, physiology, and biochemistry [3,5,11]. These low fluence rates of UV-B promote the expression in a range of genes that are known to be involved in UV-B protection or amelioration of UV-B damage [3,5,12,13]. Among the most important protective mechanisms in higher plants are the accumulation of UV-absorbing phenolic compounds in epidermal tissues [9,14,15] and the enhancement of cellular antioxidant systems [3,8]. Similar responses have also been shown in grapevine plants (*Vitis vinifera *L. cv. Malbec) [16,17].

Over the last years efforts have been focused on the study of the molecular responses of plants to UV-B radiation. Several reports have analyzed the changes in gene expression caused by UV-B in many plant species [12,13,18-20] using microarray analysis. Interestingly, these studies have shown that some UV-B response pathways are shared with other environmental cues, while additional pathways may account for UV-B-specific responses [21], pointing out the existence of common as well as stress-specific gene expression profiles [2].

In grapevine (*Vitis vinifera *L.) plants there is no available information on the changes in gene expression triggered by UV-B radiation. However, different microarray platforms have been used in this species to analyze transcriptomic variation during berry development [22-24], water and salinity stresses [25], virus infection [26] and fungal pathogen attack [27].

Malbec is a grapevine cultivar well adapted to the growing conditions of Argentine, were it became the emblematic icon of the country's wine industry. In the province of Mendoza, the major wine-producing region in the country, vineyards are located at heights ranging from 500 to 1500 m a.s.l. Such variations in altitude account for different fluence rates and dosages of UV-B reaching the vineyards [16]. Thus, Malbec plants growing at different altitude develop different UV-B responsive mechanisms that are relevant for the final composition of grape berries and affect the quality of wine [16,17].

In the present study we used a new custom made Affymetrix GrapeGen GeneChip™ (Lijavetzky et *al., In preparation*) to investigate gene expression responses of leaf tissues from *in vitro *cultured grapevine cv. Malbec plants to the environmental level of UV-B radiation found at 1000 m a.s.l. (4.75 kJ m^-2 ^d^-1^, calculated for the range 310-315 nm) [16], administered at two different fluence rates: "low UV-B" (16 h at 8.25 μW cm^-2^) or "high UV-B" (4 h at 33 μW cm^-2 ^UV-B). The minimum and maximum UV-B fluence rate of solar radiation perceived by vineyards at 1,450 m a.s.l. at early morning and noon for the Mendoza's region is of *c.a *9 and 35 μW cm^-2^, respectively [17].

The functional classification of the differentially expressed genes and the pictorial representation of the significantly modulated classes were accomplished by means of the MapMan software [28] using adapted files specifically designed for the custom made Grapegen GeneChip™. Our results showed that transcription in grapevine leaves is differentially affected by UV-B fluence rate. We found that the number of modulated probe sets (up- and down-regulated) under high fluence rate UV-B was two-fold higher than the number of probe sets modulated under low intensity UV-B. Transcripts commonly modulated under both UV-B treatments as well as transcripts specifically regulated in an UV-B-fluence rate dependent way were identified. The functional analysis of the differentially expressed genes showed that while high fluence rate UV-B had regulatory effects on defense or general stress response pathways, low fluence rate UV-B promoted the expression of genes involved in UV-B protection or the alleviation of the UV-B damage, likely contributing to the acclimation of plants to UV-B exposure.

## Results and Discussion

### High UV-B has a stronger effect on gene expression than low UV-B

**Figure 1:**
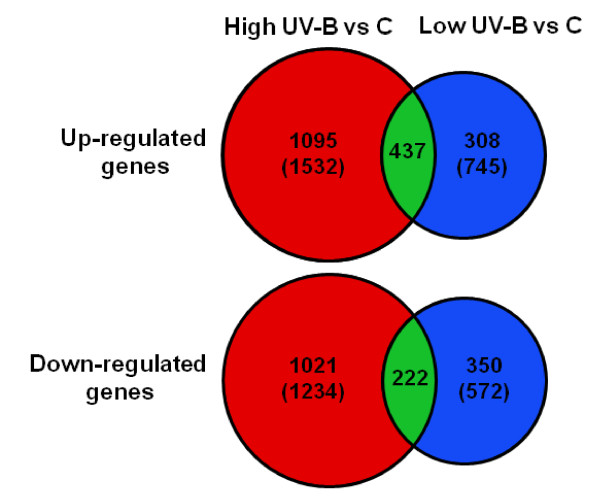
**Venn diagram representing commonly and specifically up- and down-regulated genes in response to two UV-B light treatments**. Values correspond to the comparison of differentially expressed probe sets of non-treated controls plantlets (C) with those of plantlets exposed to high and low UV-B intensity treatments. The values in parentheses correspond to the total number of differentially expressed probe sets up- or down-regulated by each UV-B treatment.

Genome wide analysis of gene expression variation in *Vitis vinifera *cv. Malbec leaves in response to two different UV-B radiation treatments was quantitatively assessed using the GrapeGen Affymetrix GeneChip™ (Lijavetzky et *al., In preparation*). Average presence identified for the 23096 probe sets in all the samples was 65.7% ± 3.63. Differential expression analysis (Clear test; *P *< 0.05) was performed on the total number of probe sets and processed afterwards to eliminate putative redundancies. This analysis revealed that high fluence rate UV-B up-regulated the expression of 1532 probe sets and down-regulated 1243, while low UV-B induced 745 and repressed 572 probe sets, both compared to control leaf tissues from plantlets grown under UV-B filtered light (Figure 1, Additional files 1 and 2). A total of 437 probe sets were commonly up-regulated by the two UV-B treatments, whereas 1095 and 308 were specifically induced by high and low UV-B, respectively. Among the down-regulated probe sets a subset of 222 was commonly repressed by both UV-B treatments, while 1021 and 350 genes were specifically down-regulated under high and low UV-B, respectively (Figure 1). In order to validate the results obtained with the microarray analysis, we carried out quantitative real-time RT-PCR (qRT-PCR) assays on 11 cDNA sequences using gene-specific primers (Additional file 3) based on the corresponding GrapeGen GeneChip™ probe set sequences. The qRT-PCR profiles were analyzed on three biological replicates of control, low UV-B and high UV-B treatments. Linear regression analysis displayed highly significant correlations (average r^2^= 0.90 ± 0.147) for 9 of the 11 evaluated genes (Additional file 4).

### Functional analyses of transcriptome changes in response to UV-B light treatments

Functional analysis of the grapevine genes differentially expressed under high and low UV-B treatments were carried out with the MapMan software [28] using a specifically designed "mapping" file. In order to build the "mapping" file we functionally classified all the genes of the GrapeGen GeneChip™ in 36 major BINs and several subBINs (Additional file 5). The "mapping" file was also adjusted to eliminate probe sets redundancies. The results of the functional analysis are summarized in Tables 1 and 2.

**Table 1:** Significantly modulated processes under High UVB

BIN	Upregulated process	**Genes**^**a**^	P value
4.2	Cellular response overview.Biotic stress	58/240	6.34E-08

5.1	Carbohydrate metabolism.Sugar binding proteins	7/27	4.29E-02

11.4.1	Protein metabolism and modification.Molecular chaperone.HSP	29/131	2.03E-04

16.1.2	Regulation overview.Hormone.Ethylene	21/86	6.84E-04
16.1.2.4	Regulation overview.Hormone.Ethylene.TFs	18/51	7.90E-06
16.2.5.14	Regulation overview.Nucleic acid metabolism.TFs.NAC	9/26	1.43E-02
16.2.5.15	Regulation overview.Nucleic acid metabolism.TFs.Pathogenesis-related	5/15	4.61E-02
16.2.5.20	Regulation overview.Nucleic acid metabolism.TFs.WRKY	10/39	2.21E-02

19	Secondary metabolism	69/364	3.43E-04
19.4	Secondary metabolism.Phenylpropanoids	41/179	2.03E-04
19.4.2	Secondary metabolism.Phenylpropanoids.Phytoalexins	10/35	9.06E-03
19.4.4	Secondary metabolism.Phenylpropanoids.General pathway	8/17	2.03E-04

**BIN**	**Downregulated process**	**Genes**^**b**^	**P value**

4.13	Cellular response overview.Cell growth and death.Cell cycle	31/142	2.03E-04

8.2.1	Signalling.Light signalling.Blue light signalling	4/9	2.45E-02

^a^Up-regulated genes in the BIN/Total genes in the BIN

^b^Down-regulated genes in the BIN/Total genes in the BIN

#### General responses induced by UV-B radiation

Nine functional categories were found to be significantly up-regulated by both UV-B treatments, whereas no functional category was found down-regulated by both UV-B treatments. Interestingly, different functional categories were specifically modulated (either induced or repressed) depending on UV-B fluence rate (Tables 1 and 2). These results are in agreement with previous reports indicating that different UV-B fluence rates can elicit different responses on diverse plant species [3,5-7]. For example, in maize certain genes are only induced above certain thresholds of UV-B irradiance intensity, suggesting the operation of diverse signaling pathways at different fluence rates within the same species [4].

**Figure 2:**
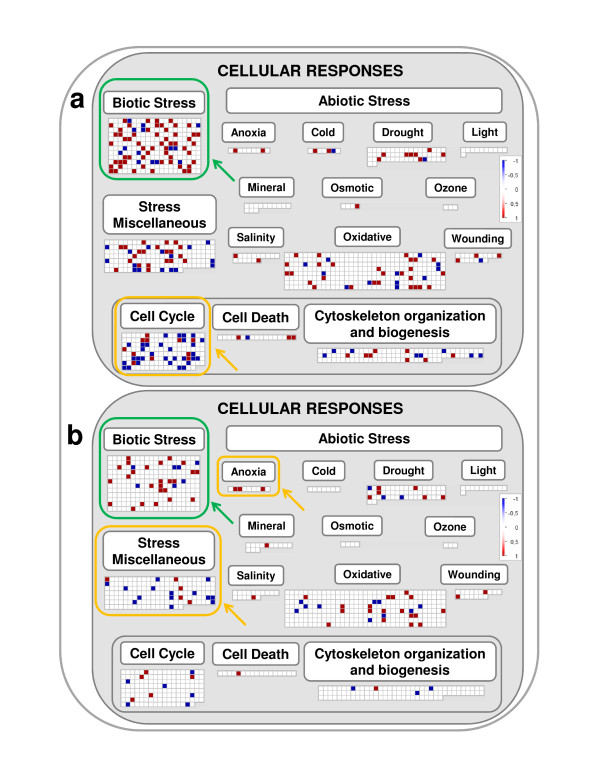
**Cellular responses**. MapMan visualization of the Cellular responses "pathway" modulation under (a) high and (b) low UV-B treatments. Functional categories commonly regulated by both UV-B treatments are enclosed within green boxes, while those specifically regulated are enclosed within yellow boxes.

Over-expression of the functional category "Biotic stress" (BIN 4.2) was a common feature of both UV-B treatments (Tables 1 and 2). However, high fluence rate UV-B had the strongest inductive effect on this category (i.e. the number of high UV-B-induced genes included in the "Biotic stress" category was higher compared with those induced by low UV-B; Figure 2, Additional files 6 and 7). It has been reported that UV-B elicited responses share gene activation and signal transduction pathways commonly associated with biotic stresses [3,10,29]. Consistent with this, several genes putatively involved in pathogen signal transduction and defense responses were found to be up-regulated when the grapevine plantlets were irradiated with either high or low fluence rate UV-B light. Genes encoding for NBS-LRR type Disease Resistance protein, Avr9 Cf-9 rapidly elicited protein, and Syringolide-induced proteins were highlighted in the above category (Additional files 6 and 7). These overlapping responses among UV-B, wounding, and pathogenesis could result from the accumulation of common signaling molecules mediating wound/defense responses such as calcium, reactive oxygen species (ROS, [10]), or hormones such as ethylene. Thus, the effects of UV-B light on the induction of biotic stress associated-genes, could improve plant resistance to pathogens and pests. Moreover, the effects of UV-B light on plant wounding responses to herbivorous insects are well known [30], indicating the use of shared components and a possible mechanism of cross-tolerance.

**Figure 3:**
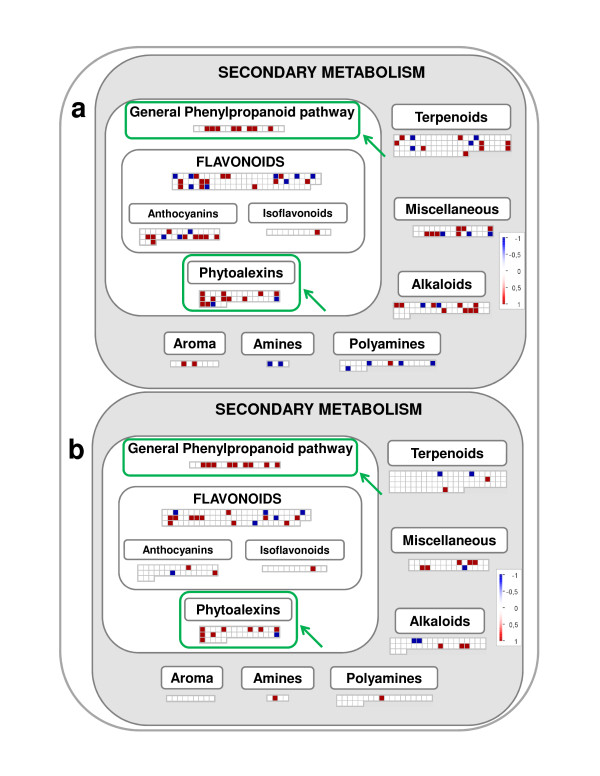
**Secondary metabolism**. MapMan visualization of the Secondary metabolism "pathway" modulation under (a) high and (b) low UV-B treatments. Functional categories commonly regulated by both UV-B treatments are enclosed within green boxes.

One of the most effective mechanisms of protection against potentially damaging UV-B radiation is to reduce its penetration into plant tissues. It is known that biosynthesis of secondary metabolites such as flavonoids and other UV-B absorbing phenolic compounds, which accumulate in the vacuoles of epidermal cells, is an important molecular event underlying UV-B acclimation in plants [12,19,20], including grapevine [17]. Grapevine plantlets exposed to low and high fluence rate UV-B displayed a significant inductive effect on the functional category "Secondary metabolism" (BIN 19, Tables 1 and 2) and more specifically in the Phenylpropanoid pathway (BIN 19.4) and its segments corresponding to "Phenylpropanoid general pathway" (BIN 19.4.4, Tables 1 and 2, Figure 3) and Phytoalexins (BIN 19.4.2, Tables 1 and 2, Figure 3). Several phenylalanine ammonia-lyase (PAL) genes, as well as genes encoding Cinnamate 4-hydroxylase (C4H) and 4-Coumarate CoA ligase (4CL), were upregulated in response to UV-B (Additional files 6 and 7). In addition to the significant expression of genes encoding structural enzymes of the general phenylpropanoid pathway, genes involved in lignin biosynthesis, such as Caffeoyl-CoA O-methyltransferase were also up-regulated under both UV-B treatments (Additional files 6 and 7).

In grapevine, chalcone synthase (CHS) substrates can also be used by stilbene synthase (STS) enzymes for the production of stilbenes, which are classified as phytoalexins because of their role in plant defense mechanisms against fungal pathogens [31,32]. Among stilbenes, resveratrol (*trans*-3,5,4'-trihydroxystilbene) is the most prominent compound [33]. The accumulation of resveratrol in grapevine berries and leaves is induced by several stresses such as fungal infection, injury and UV-B light exposure [16,31]. As shown in Figure 3, many genes assigned to the functional category "Phytoalexins" (BIN 19.4.2) were induced under both UV-B treatments. Among them, genes mainly encoding STS and resveratrol synthases gave significance to this functional category [34] (Additional files 6 and 7). The enhanced representation of "Phenylpropanoid general pathway" and "Phytoalexins" categories show that UV-B promoted the expression of genes involved in the induction of different antioxidant defense systems. This is in agreement with the observation that in grapevine the synthesis of anthocyanins, flavonols, quercetin and kaempferol is promoted by exposure of plants to environmental doses of UV-B light [17].

Plant hormones play important roles in diverse growth and developmental processes as well as in various plant responses to biotic and abiotic stresses. In particular, ethylene, auxins, abscisic acid (ABA) and brassinosteroids have been associated with the UV-acclimated phenotypes [13,17,20,35]. As shown in Tables 1 and 2 and Additional file 8a, the "Ethylene metabolism" (BIN16.1.2) and "Ethylene responsive TFs" (TFs: Transcription Factor/s, BIN 16.1.2.4) were functional categories significantly over represented under both UV-B treatments. It has been established that ethylene as well as ethylene response factor (ERF) proteins play important regulatory roles in plant pathogen resistance and abiotic stress response [36,37]. ERFs belong to a large family of APETALA2-domain-containing TFs that bind to a GCC-box present in the promoters of many ethylene inducible defense-related genes [38]. We found that several genes, putatively annotated as ERFs and ethylene responsive proteins, were commonly over expressed under both UV-B treatments (Additional files 6 and 7). These results are in agreement with a previous study carried out by Ulm et al. [13] in Arabidopsis, showing that activation of the ethylene pathway is a general response to UV-B exposition. In grapevine, ethylene plays an important role in berry development and ripening processes including the regulation of gene expression for anthocyanin biosynthesis and accumulation [39]. As shown here, ethylene also seems to participate in the modulation of leaf responses to UV-B radiation (Tables 1 and 2, Additional file 8a).

**Figure 4:**
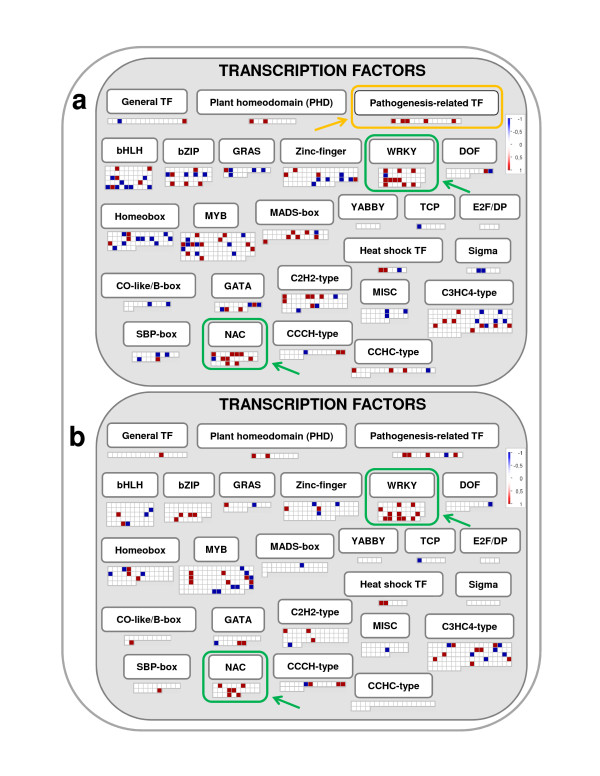
**Transcription factors**. MapMan visualization of the Transcription factors "pathway" modulation under (a) high and (b) low UV-B treatments. Functional categories commonly regulated by both UV-B treatments are enclosed within green boxes, while those specifically regulated are enclosed within yellow boxes.

Many transcription factors are known to be rapidly induced by UV-B [13,21] and these proteins likely play key roles in UV-B-induced responses. Apart from ERFs, two additional categories of TF families were significantly over represented after exposition of grapevine plantlets to both UV-B light: "WRKY TFs" (BIN 16.2.5.20), and "NAC TFs" (BIN 16.2.5.14, Tables 1 and 2, Figure 4). The NAC domain (Petunia NAM and Arabidopsis ATAF1/2 and CUC2, [40]) proteins are plant specific TFs and are expressed in various developmental stages and tissues, although details of their interactions with DNA and with other proteins are still limited [41]. This family comprises more than one hundred genes in Arabidopsis and is involved in diverse processes including growth and development as well as in responses to hormones, light, and biotic and abiotic stresses [13,41].

The WRKY proteins share a DNA binding domain which contains an invariant WRKYGQK sequence (after which the domain was named, [42]). The WRKY TF super family is involved in a diverse set of biological functions including pathogen defense, abiotic stress responses and plant development [43,44]. As shown in Figure 4, several genes encoding WRKY factors showed an increased expression in response to both UV-B treatments (Additional files 6 and 7), suggesting a role for these factors as components of grapevine protection mechanisms against potentially damaging UV-B radiation.

#### Responses specifically regulated by high fluence rate UV-B radiation

High fluence rate UV-B significantly modulated gene expression in additional functional categories. Among the up-regulated processes three functional categories were significantly overrepresented: BIN 5.1 corresponding to "Sugar binding proteins", BIN 11.4.1 "Heat shock proteins" (Hsps) and BIN 16.2.5.15 "Pathogenesis related TFs" (Table 1).

The functional category of "Sugar binding proteins" (Table 1, Additional file 8c) includes genes mainly encoding different kind of lectins (curculin-like, -Mannose-binding- lectin, D-galactoside L-rhamnose binding lectin, and lectin 2) and glycoproteins (Additional file 6). Lectins comprise a miscellaneous group of proteins able to bind carbohydrate residues of different chemical nature in a specific and reversible way. Although protection against pathogens is the major function attributed to lectins, their accumulation in plant tissues have also been described under different abiotic stresses or as a result of growth and developmental processes [45-47]. These proteins could play a role in the protection of plant cell against damaging effects of free radicals as hydrogen peroxide [48]. Thus, in the high fluence UV-B response, the induction of lectins could be interpreted as a mechanism to shorten the duration of the "oxidative burst" and to protect grapevine leaves against high UV-B induced-ROS.

Hsps and their regulatory transcription factors (Hsfs) play a broad role as molecular chaperones in the tolerance to multiple environmental stresses apart from heat stress [49]. Some Hsps act as molecular chaperones counteracting protein denaturation and aggregation while other Hsps including ubiquitin and certain proteases, target nonnative proteins for degradation [50]. Hsps function may extend beyond their chaperone activity, limiting the damage that results from ROS accumulation. In fact, exposure to UV-B radiation, which increases cellular concentrations of hydrogen peroxide, activates Hsfs expression [51]. In agreement with those reports our results show that genes encoding several Hsps (with mitochondrial, chloroplastic, and cytoplasmic localization) as well as Hsfs are differentially up-regulated under high fluence rate UV-B (Additional file 6).

The induction of the expression of genes in the functional category of "Pathogenesis related TFs" detected under high UV-B but not under low UV-B (Table 1, Figure 4) could be part of the same general response to biotic stress (BIN 4.2) observed at both UV-B fluence rates mentioned above. Given the existence of a slight difference in the number of genes induced under each UV-B treatment this category is significantly represented only under high UV-B. However, this category must be relevant under both conditions.

Down regulation of gene expression is also an important effect of UV-B light exposure although as mentioned above fluence rates have specific effects on the functional categories that are down regulated. The strong down-regulation of the category "Cell cycle" (BIN 4.13) is a specific feature of high UV-B (Table 1, Figure 2). Indeed, our results revealed that the activities of several cell cycle-related genes are temporally paused in the leaves of grapevine plantlets exposed to high UV-B (Additional file 9). High UV-B specifically down-regulates the expression of genes encoding cyclins, cyclin-dependent kinase inhibitors, kinesins and cell division control proteins (Additional file 9). It is likely advantageous for grapevine cells to temporally halt cell cycle, to allow for repairing DNA damage and to prevent the introduction of mutations into the DNA of daughter cells. It is well documented that UV-B photons may cause cellular damage by generating photoproducts of DNA [52]. DNA damage is also known to induce cell cycle arrest and rapid protein turnover via the proteasome [15,53]. In fact, it has been proposed that UV-B could delay cell division by arresting cell cycle in the G1/S transition phase [54].

Finally, among the functional categories specifically regulated by high UV-B, "Blue light signaling" (BIN 8.2.1) is also found down-regulated (Table 1, Additional file 8e). Genes encoding TFs acting downstream of phototropin photoreceptors are differentially repressed under high UV-B (Additional file 9). Phototropins (phot1 and phot2) are blue-light (BL)-sensitive receptor kinases, involved in the control of BL-activated stomatal opening and chloroplast relocation in addition to the phototropism response [55,56]. It is known that these phototropins use signal transducers for different photoinduced movement responses in different tissue. Thus, in the phototropic response of hypocotyls, nonphototropic hypocotyl 3 (NPH3) is a common regulator in the phot1-and phot2-signaling pathways [57]. Also, it was shown that NPH3-mediated phototropin signaling optimizes the efficiency of BL-perception by inducing both optimal leaf positioning and leaf flattening [58]. Our results show a significant down-regulation of the NHP3 transcription factor (Additional file 9) suggesting that high UV-B might trigger a negative phototropic response of grapevine leaves as an escape response to protect leaf tissues against potentially damaging UV-B radiation.

#### Differential responses regulated by low UV-B radiation

Similar to the functional categories specifically regulated by high fluence rate UV-B, different processes represented in additional categories are regulated by low UV-B (Table 2). Among them, the functional category "Abscisic acid metabolism" (BIN 16.1.4.1, Table 2, Additional file 8a) is specifically up-regulated by low UV-B light. It is known that ABA mediates adaptive responses to abiotic and biotic stresses in vegetative tissues [59,60], although the role of ABA in UV-B-induced responses has just begun to be elucidated [16,17,61]. Analysis of the microarray data shows that genes encoding Cytochrome P450 and ABA 8'-hydroxylase (the major ABA catabolic pathway in higher plants, Additional file 7) are up-regulated by low UV-B. An increased ABA turnover could be a possible consequence for these results.

**Table 2:** Significantly modulated processes under Low UVB

BIN	Upregulated process	**Genes**^**a**^	P value
4.1	Cellular response overview.Abiotic stress.Anoxia	3/9	1.30E-02
4.2	Cellular response overview.Biotic stress	25/240	1.12E-02

15	ATPase family associated with various cellular activities	6/31	9.42E-03

16.1.2	Regulation overview.Hormone.Ethylene	12/86	1.19E-02
16.1.2.4	Regulation overview.Hormone.Ethylene.TFs	9/51	1.11E-03
16.1.4.1	Regulation overview.Hormone.ABA.Metabolism	2/2	3.86E-05
16.2.5	Regulation overview.Nucleic acid metabolism.TFs	63/738	3.54E-02
16.2.5.14	Regulation overview.Nucleic acid metabolism.TFs.NAC	6/26	2.51E-03
16.2.5.20	Regulation overview.Nucleic acid metabolism.TFs.WRKY	11/39	2.37E-07

19	Secondary metabolism	43/364	3.86E-05
19.4	Secondary metabolism.Phenylpropanoids	31/179	6.40E-07
19.4.2	Secondary metabolism.Phenylpropanoids.Phytoalexins	8/35	2.51E-03
19.4.4	Secondary metabolism.Phenylpropanoids.General pathway	9/17	1.51E-11

**BIN**	**Downregulated process**	**Genes**^**b**^	**P value**

3	Cell wall metabolism	36/426	5.87E-03
3.2	Cell wall metabolism.Cell wall modification	23/198	2.57E-04

4.10	Cellular response overview.Stress Miscellaneous	13/161	4.69E-02

16.1.3	Regulation overview.Hormone.Auxin	10/93	1.94E-02

^a ^Up-regulated genes in the BIN/Total genes in the BIN

^b^Down-regulated genes in the BIN/Total genes in the BIN

Exposure of grapevine plantlets to low UV-B specifically down-regulates genes in the functional category "Stress miscellaneous" (BIN 4.10, Table 2, Figure 2). An overview of the genes which gave significance to this category shows that low UV-B affected components commonly involved in stress responses such as putative Thaumatin-like proteins, Pathogenesis-related protein PR-1 precursor, putative senescence-associated proteins, Allergen V5 Tpx-1 and chitinase-like proteins (Additional file 10) [62,63]. These genes have been associated with plant defense to pathogens attack or environmental stress but they are also developmentally regulated [64]. In our experiment, their expression could be more related to growth processes and secondary metabolic changes induced by low UV-B, than to responses triggered by UV-B stress.

Another functional category whose expression was specifically enhanced by low fluence rate UV-B is the "ATPase family" (BIN 15, Table 2, Additional file 8d). Genes encoding a particular class of ATPases, named AAA ATPase is specifically up-regulated under this UV-B treatment (Additional file 7). ATPase Associated with various cellular Activities (AAA) proteins are characterized by the presence of one or several conserved motives including the Walker A and Walker B required for ATP binding and hydrolysis, respectively, and a highly conserved amino acid sequence termed the second region of homology (SRH, [65]). This protein family is commonly distributed among eukaryotes and is involved in several cellular functions. Information on the role of AAA ATPases is very limited in plants. Some studies have related them with degradation of thylakoid proteins by the 26S proteasome [66]. Our results might suggest an effect of low fluence rate UV-B on chloroplast proteins turnover.

**Figure 5:**
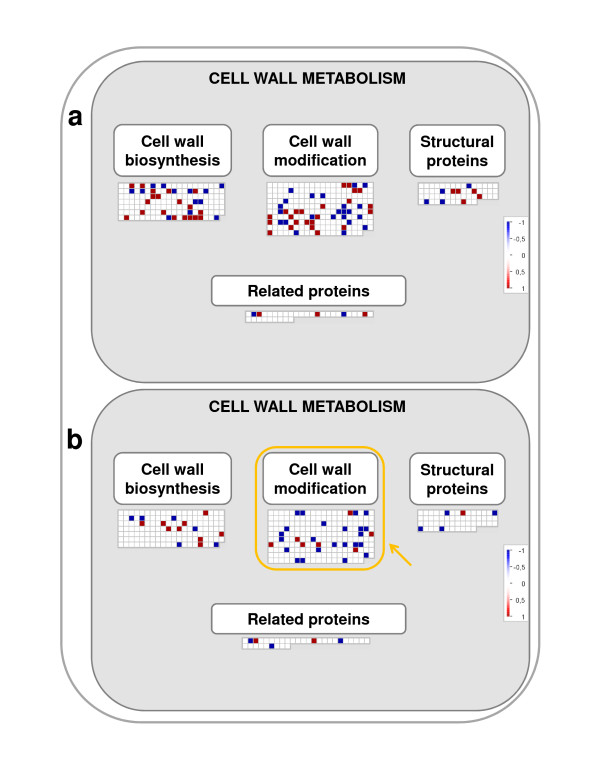
**Cell wall metabolism**. MapMan visualization of the Cell wall metabolism "pathway" modulation under (a) high and (b) low UV-B treatments. Functional categories commonly regulated by both UV-B treatments are enclosed within green boxes.

Our microarray studies with *in vitro *grapevine plantlets exposed to low UV-B shows a specifically down-regulation in gene expression within the category "Cell wall modification" (BIN 3.2, Table 2, Figure 5). Particularly, genes involved in the control of cell wall loosening give significance to this category (Additional file 10). Among them, genes encoding pectate lyase, xylan 1,4-beta-xylosidase, glucanases, polygalacturonases, β-1,3-glucanase and expansins are specifically down-regulated in response to low UV-B (Additional file 10). Thus, a reduction in the hydrolysis of matrix polysaccharides by low UV-B is an identifiable effect in the cell wall class of genes. These results are in agreement with those found by Hectors et al. [20], who showed a reduction of cell wall loosening gene expression as part of UV-B acclimation mechanism.

Finally, exposure of grapevine plantlets to low UV-B has a repressive effect on the category "Auxin metabolism" (BIN 16.1.3, Table 2, Additional file 8a). Our results show that genes belonging to auxin responsive SAUR and Aux/IAA family, auxin response factors and auxin transporter-like proteins are down-regulated in the grapevine leaves exposed to low UV-B (Additional file 10). Thus, down-regulation of auxin signaling components or auxin transport supports a role for auxins in the response to low UV-B fluence light. Similar results were found in the study of pathogen resistance responses, where a repression of a number of auxin responsive genes (including genes *SAUR*, *Aux/IAA*, auxin importer *AUX1*, auxin exporter *PIN7*) were significantly repressed [67]; supporting the idea that down-regulation of auxin signaling contributes to induce immune responses in plants [68].

## Conclusions

The leaf transcriptome of grapevine was differentially affected by UV-B fluence rate. The number of genes modulated (up- and down-regulated) by UV-B light exposure was two-fold higher under high fluence rate UV-B than under low fluence rate UV-B. Analysis of the functional categories differentially represented under high and low UV-B revealed that the existence of common and specific responses regulated at different UV-B intensity. General protective responses such as the induction of pathways regulating the synthesis of UV-B absorbing phenolic compounds, the induction of antioxidant defense systems, and the activation of signal transduction pathways associated with biotic and abiotic stress responses played critical roles independently of fluence rate. Furthermore, increased fluence rates triggered additional stress responses involving the induction of heat shock and sugar binding proteins and causing a rapid decline in the expression of genes involved in cell cycle. Then again, low fluence rate UV-B promoted additional transcriptional responses that could be interpreted as acclimation processes such as the induction of ABA catabolism and chloroplast protein turnover associated ATPases or the repression of genes involved in cell wall modification and auxin metabolism and responses. These findings provide a preliminary functional genomics framework to understand the complexity of grapevine UV-B radiation responses.

## Methods

### Plant material and in vitro culture

Wood cuttings corresponding to three-nodal lignified segments derived from virus-free plants of *Vitis vinifera *L. cv. Malbec were planted in 2.5 l plastic pots containing hydrated-perlite and grown under greenhouse conditions. One-node cuttings coming from three-month old plants were collected and used as explants for *in vitro *culture. Explants were surface sterilized with 75% ethanol for 3 min and then with 15% sodium hypochlorite for 15 min. The cuttings were rinsed three times in sterile dH_2_O and cultivated on solid medium composed of full MS medium salts and vitamins [69], 30 g l^-1 ^sucrose, 1 mg l^-1 ^6-benzylaminopurine (BAP), and 7.5 g l^-1 ^agar for shoot initiation and proliferation of grapevine shoot-tip explants. *In vitro *shoot tips were subcultured (five times) at 35-40-day intervals into half-strength MS micro- and macro-nutrients, excluding FeEDTA (0.1 mM Na_2_EDTA + 0.1 mM FeSO_4_) and supplemented with full-strength MS vitamins, 30 g l^-1 ^sucrose, 0.5 μM 1-naphthaleneacetic acid and 7.5 g l^-1 ^agar. Only one explant per bottle (12 cm height × 6.5 cm diameter) was planted. Cultures were maintained in a growth chamber at 25 ± 2 ºC under 16 h photoperiod provided by cool-white fluorescents tubes at a photosynthetic photon flux density of 80 μmol m^-2 ^s^-1^. Bottle tops were covered with low-density polyethylene, which does not absorb any wavelength of the photosynthetic light.

### UV-B exposure conditions

*In vitro *grown plants (45-48 d old, having 6 full expanded leaves) were exposed to UV-B radiation in the same controlled growth chambers described above. Throughout the UV-B treatment, all plantlets were exposed to a background intensity of photosynthetically active radiation (PAR, 80 μmol m^-2 ^s^-1^) provided by cool-white fluorescent lamps. For different light treatments, supplemental UV-B was given using a Philips TL 100W/01 tube (311 and 313 nm spectrum peaking, Philips, Eindhoven, The Netherlands) suspended 40 cm above the pots. A total effective dose of 311 nm normalized UV-B corresponding to 4.75 kJ m^-2 ^d^-1 ^was provided in two different treatments: 1) "low UV-B" (16 h at ≅8.25 μW cm^-2 ^irradiance) and "high UV-B" (4 h at ≅33 μW cm^-2 ^irradiance) supplied at the end of the 16h-day photoperiod). As a control of no UV-B radiation, plantlets were exposed for 16 h under the same UV-B tube covered with a polyester filter (100 μm clear polyester plastic, Oeste Aislante, Buenos Aires, Argentina), which absorbed more than 95% of UV-B without affecting PAR. It is worth mentioning that UV-B irradiance registered at *ca*. 1,500 m a.s.l., the altitude where the most reputed vineyards are located in the Mendoza's region, is about 35 μW cm^-2 ^solar noon. A LI-250A light meter with a LI-190 quantum sensor (LI-COR Inc., Lincoln, NE, USA) and a PMA2200 radiometer with a PMA2102 UV-B detector (Solar Light Company Inc., Glenside, USA) were used to measure PPFD and UV-B, respectively.

As a control for circadian effects, only the fully expanded-upper leaves from two plantlets collected from each experimental treatment were harvested and pooled immediately at the end of the light treatments. Samples were immediately frozen in liquid nitrogen and conserved at -80 ºC until RNA extraction. Three independently grown, harvested, and extracted sets of samples corresponding to each experimental treatment were prepared as biological replicates.

### RNA isolation and GeneChip^® ^hybridization

Total RNA was extracted from leaf tissues using the TRIZOL reagent (Invitrogen) according to standard procedures. DNase digestion of contaminating DNA in the RNA samples was carried out with the RNase-Free DNase Set (QIAGEN). Final RNA purification was performed using the RNeasy Mini Kit (QIAGEN) according to standard protocols. Samples were analyzed at the Genomics Unit of the Spanish National Centre for Biotechnology (CNB-CSIC, Madrid). RNA integrity analyses were done with an Agilent's Bioanalyzer 2100. Probe synthesis, microarrays hybridization, washing, staining and scanning with the GeneChip™ Scanner 3000 were performed according to the Affymetrix GeneChip^® ^Expression Analysis Technical Manual.

### Differential expression and functional analyses

The mRNA expression profiles of the different treatments described above were compared using the Affymetrix GrapeGen custom GeneChip™ (Lijavetzky et *al., In preparation*). This new GeneChip generated for grapevine (genome size about 475 Mb) contains 23096 probe sets. Probe sets design was based on the publicly available Unigen information at the National Center for Biotechnology Information [70] by July 2006 (342576 grapevine ESTs). The probe sets represent *Vitis vinifera *consensus sequences (when overlapped) from Cabernet Sauvignon, Muscat Hamburg, Pinot Noir, Chardonnay and Shiraz cultivars. In order to determine the number of non-redundant genes represented in the GeneChip we mapped the original 23096 probe sets to the recently released 12X grapevine genomic sequence [71] and we determined that the GeneChip contains probes sets for ca. 15800 different annotated genes. Differential expression analyses were performed using the entire probe set information. In order to carry out the functional analyses with unique probe sets we processed the corresponding produced gene list using a Microsoft Excel spreadsheet containing both the original and "12X" probe set information. Non-redundant probe sets codes were replaced with their unique gene identifier at the "12X" while redundant probe sets were merged and we assigned their median value to the corresponding unique identifier at the 12X.

Data analysis for each treatment was performed using biological triplicates. Normalization was performed with the help of the normalization tool within the GEPAS 4.0 suite [72,73]. For this procedure we used all the default Affymetrix methods defined by the GEPAS 4.0 system which were implemented in the "affy" package from Bioconductor [74]: a) background correction by Robust Multi-array Average (RMA) expression measure [75], b) between array standardization based upon quantiles [76], and c) PM-MM adjustment using "pmonly" (just PM values are used). Differential expression analysis (*P *< 0.05) was carried out using the Clear Test method [77] implemented at the GEPAS 4.0 suite. This method tries to avoid the problem caused by *t*-tests when declaring genes with relatively small expression changes as differentially expressed if they show low observed variances. The Clear Test proposes to use the *z*-test as an alternative and combines that test with a χ2 test to evaluate variability [77] what helps to prevent the identification of genes with high observed variances as differentially expressed. Functional analyses of the differentially expressed genes were done with the MapMan software based on the Wilcoxon Rank Sum Test [28].

### Classification of the GrapeGen GeneChip™ probesets and MapMan files preparation

Original *Vitis vinifera *sequences used for the GrapeGen GeneChip™ probe design and annotation were classified into a MapMan "mapping file" after performing automated and manual database searches. Sequence were blasted against the UniProtKB/Swiss-Prot plant proteins sequence database [78] and classified into a modified and adapted "mapping file" using as a reference model the original *Arabidopsis *file [28] as well as the related information present in other databases such as GO [79], KEGG [80], Swiss-Prot [78] and TAIR [81]. Out of the 23046 grapevine sequences, 15783 were actually sorted in 36 BINs and several subBINs (Additional file 3). A total of 22 pictorial "pathway" representations were also mainly adapted from *Arabidopsis *while some specific files were originally developed (Figures 2, 3, 4 and 5, Additional file 8).

### Real time quantitative RT-PCR (qRT-PCR)

Reactions and cDNA synthesis were performed according to Lijavetzky et al. [82]. Transcript levels were determined using a 7300 Real-Time PCR System (Applied Biosystems) and SYBR Green dye (Applied Biosystems). Gene specific primers (Additional file 3) were designed using the Oligo Explorer 1.2 software (Gene Link) based on the corresponding probe set sequence from the custom GrapeGen GeneChip™. No-template controls were included for each primer pair, and each PCR reaction was completed in triplicate. Data were analyzed using the 7300 SDS software 1.3 (Applied Biosystems). Dissociation curves for each amplicon were then analyzed to verify the specificity of each amplification reaction. Transcript level was calculated using the standard curve method and normalized against grapevine EFα1 gene (UniGene Vvi.1750) used as reference control.

### Data Availability

All microarray expression data produced in our study are publically available at the ArrayExpress database [83] under the accession number E-MEXP-2541.

## Authors' contributions

MP designed and coordinated the study, carried out *in vitro Vitis vinifera *cv. Malbec plantlets and UV-B experiments, drafted the manuscript, and together with PP carried out the RNA isolations. PP participated in the design of the study and together with RB helped with critical discussions on the work. RF participated in the design of functional analyses tools. RB and JMMZ conceived the study and participated in the manuscript draft. DL carried out qRT-PCR amplifications and analyses, performed statistical analysis on microarray data, designed functional analyses tools and participated in the draft and edition of the manuscript. All authors read and approved the final manuscript.

## Supplementary Material

Additional file 1 **Full list of genes differentially expressed under high UV-B radiation**. PDF file showing a complete list of the genes differentially expressed in the high UV-B treatment including Probe-set ID, Unique grapevine gene ID, Annotation and fold-change. Click here for file

Additional file 2 **Full list of genes differentially expressed under low UV-B radiation**. PDFgenes file showing a complete list of the genes differentially expressed in the low UV-B treatment including Probe-set ID, Unique grapevine gene ID, Annotation and fold-change. Click here for file

Additional file 3 **qRT-PCR primers**. PDF file describing the DNA primers used for quantitative real time reverse transcription polymerase chain reaction (qRT-PCR). Click here for file

Additional file 4 **qRT-PCR expression validation**. PDF file illustrating the comparison of gene expression values reported by the GrapeGen *Vitis vinifera *Affymetrix GeneChip^® ^and by quantitative real-time RT-PCR (qRT-PCR). The microarray log_2 _(expression ratio) values (y-axis) are plotted against the log_2 _(expression ratio) obtained by qRT-PCR (x-axis). Linear regression analyses (r^2 ^values) are shown as insets. Click here for file

Additional file 5 **MapMan annotation**. PDF file describing the MapMan BIN structure and the number of genes included in each BIN and subBIN. Click here for file

Additional file 6 **Up-regulated functional classes High UV-B**. PDF file showing the full list of differentially expressed genes included in the up-regulated functional categories under high UV-B radiation described in Table 1. Positive and negative symbols represent higher or lower transcript levels under UV-B light compared with the control, respectively. Click here for file

Additional file 7 **Up-regulated functional classes Low UV-B**. PDF file showing the full list of differentially expressed genes included in the up-regulated functional categories under low UV-B radiation described in Table 2. Positive and negative symbols represent higher or lower transcript levels under UV-B light compared with the control, respectively. Click here for file

Additional file 8 **MapMan diagrams of all significantly modulated functional categories not included as main Figures in the text**. PDF file displaying the pictorial representation of the differentially expressed genes included in the following classes: (a) "Hormone", (b) "Protein metabolism and modification", (c) "Carbohydrate Metabolism", (d) "Metabolism-enzyme families", (e) "Signalling". Functional categories commonly regulated by both UV-B treatments are enclosed within green boxes, while those specifically regulated are enclosed within yellow boxes. Click here for file

Additional file 9 **Down-regulated functional classes High UV-B**. PDF file showing the full list of differentially expressed genes included in the down-regulated functional categories under high UV-B radiation described in Table1. Positive and negative symbols represent higher or lower transcript levels under UV-B light compared with the control, respectively. Click here for file

Additional file 10 **Down-regulated functional classes Low UV-B**. PDF file showing the full list of differentially expressed genes included in the down-regulated functional categories under low UV-B radiation described in Table 2. Positive and negative symbols represent higher or lower transcript levels under UV-B light compared with the control, respectively. Click here for file

## References

[B1] Madronich S McKenzie RL Caldwell MM Björn LO Changes in ultraviolet radiation reaching the Earth's surface AMBIO 1995 24 143 152 10.1016/s1011-1344(98)00182-1 9894350

[B2] Blanding CR Simmons SJ Casati P Walbot V Stapleton AE Coordinated regulation of maize genes during increasing exposure to ultraviolet radiation: identification of ultraviolet-responsive genes, functional processes and associated potential promoter motifs Plant Biotechnol J 2007 5 6 677 695 10.1111/j.1467-7652.2007.00282.x 17924934

[B3] Brosché M Strid Å Molecular events following perception of ultraviolet-B radiation by plants Physiol Plant 2003 117 1 1 10 10.1034/j.1399-3054.2003.1170101.x

[B4] Casati P Walbot V Rapid transcriptome responses of maize (*Zea mays*) to UV-B in irradiated and shielded tissues Genome Biol 2004 5 3 R16 10.1186/gb-2004-5-3-r16 15003119 PMC395766

[B5] Frohnmeyer H Staiger D Ultraviolet-B radiation-mediated responses in plants. Balancing damage and protection Plant Physiol 2003 133 1420 1428 10.1104/pp.103.030049 14681524 PMC1540342

[B6] Jenkins GI Signal transduction in responses to UV-B radiation Annu Rev Plant Biol 2009 60 407 431 10.1146/annurev.arplant.59.032607.092953 19400728

[B7] Ulm R Nagy F Signalling and gene regulation in response to ultraviolet light Curr Opin Plant Biol 2005 8 5 477 482 10.1016/j.pbi.2005.07.004 16039155

[B8] Jansen MAK Gaba V Greenberg BM Higher plants and UV-B radiation: balancing damage, repair and acclimation Trends Plant Sci 1998 3 4 131 135 10.1016/S1360-1385(98)01215-1

[B9] Rozema J van de Staaij J Björn LO Caldwell M UV-B as an environmental factor in plant life: stress and regulation Trends Ecol Evol 1997 12 1 22 28 10.1016/S0169-5347(96)10062-8 21237957

[B10] Stratmann J Ultraviolet-B radiation co-opts defense signaling pathways Trends Plant Sci 2003 8 11 526 533 10.1016/j.tplants.2003.09.011 14607097

[B11] Caldwell MM Ballaré CL Bornman JF Flint SD Björn LO Teramura AH Kulandaivelu G Tevini M Terrestrial ecosystems, increased solar ultraviolet radiation and interactions with other climatic change factors Photochem Photobiol Sci 2003 2 1 29 38 10.1039/b211159b 12659537

[B12] Casati P Walbot V Gene expression profiling in response to ultraviolet radiation in maize genotypes with varying flavonoid content Plant Physiol 2003 132 4 1739 1754 10.1104/pp.103.022871 12913132 PMC181262

[B13] Ulm R Baumann A Oravecz A Máté Z Ádám É Oakeley EJ Schafer E Nagy F Genome-wide analysis of gene expression reveals function of the bZIP transcription factor HY5 in the UV-B response of Arabidopsis Proc Natl Acad Sci USA 2004 101 5 1397 1402 10.1073/pnas.0308044100 14739338 PMC337064

[B14] Bornman JF Reuber S Cen YP Weissenböck G Lumsden PJ Ultraviolet radiation as a stress factor and the role of protective pigments Plants and UV-B: Responses to Environmental Change 1997 Cambridge: Cambridge University Press 157 168 full_text

[B15] Logemann E Wu SC Schroder J Schmelzer E Somssich IE Hahlbrock K Gene activation by UV light, fungal elicitor or fungal infection in Petroselinum crispum is correlated with repression of cell cycle-related genes Plant J 1995 8 6 865 876 10.1046/j.1365-313X.1995.8060865.x 8580959

[B16] Berli F D'Angelo J Cavagnaro B Bottini R Wuilloud R Silva MF Phenolic Composition in Grape (Vitis vinifera L. cv. Malbec) Ripened with Different Solar UV-B Radiation Levels by Capillary Zone Electrophoresis J Agric Food Chem 2008 56 9 2892 2898 10.1021/jf073421+ 18412357

[B17] Berli F Moreno D Piccoli P Hespanhol-Viana L Silva MF Bressan-Smith R Cavagnaro B Bottini R Abscisic acid is involved in the response of grape (*Vitis vinifera *L.) cv. Malbec leaf tissues to ultraviolet-B radiation by enhancing ultraviolet-absorbing compounds, antioxidant enzymes and membrane sterols Plant Cell Environ 2009 33 1 1 10 19781012 10.1111/j.1365-3040.2009.02044.x

[B18] Brosché M Schuler MA Kalbina I Connor L Strid Å Gene regulation by low level UV-B radiation: identification by DNA array analysis Photochem Photobiol Sci 2002 1 9 656 664 10.1039/b202659g 12665302

[B19] Casati P Stapleton AE Blum JE Walbot V Genome-wide analysis of high-altitude maize and gene knockdown stocks implicates chromatin remodeling proteins in response to UV-B Plant J 2006 46 4 613 627 10.1111/j.1365-313X.2006.02721.x 16640598

[B20] Hectors K Prinsen E De Coen W Jansen MAK Guisez Y *Arabidopsis thaliana *plants acclimated to low dose rates of ultraviolet B radiation show specific changes in morphology and gene expression in the absence of stress symptoms New Phytol 2007 175 2 255 270 10.1111/j.1469-8137.2007.02092.x 17587374

[B21] Kilian J Whitehead D Horak J Wanke D Weinl S Batistic O D'Angelo C Bornberg-Bauer E Kudla J Harter K The AtGenExpress global stress expression data set: protocols, evaluation and model data analysis of UV-B light, drought and cold stress responses Plant J 2007 50 2 347 363 10.1111/j.1365-313X.2007.03052.x 17376166

[B22] Deluc LG Grimplet J Wheatley MD Tillett RL Quilici DR Osborne C Schooley DA Schlauch KA Cushman JC Cramer GR Transcriptomic and metabolite analyses of Cabernet Sauvignon grape berry development BMC Genomics 2007 8 429 10.1186/1471-2164-8-429 18034876 PMC2220006

[B23] Grimplet J Deluc LG Tillett RL Wheatley MD Schlauch KA Cramer GR Cushman JC Tissue-specific mRNA expression profiling in grape berry tissues BMC Genomics 2007 8 187 10.1186/1471-2164-8-187 17584945 PMC1925093

[B24] Terrier N Glissant D Grimplet J Barrieu F Abbal P Couture C Ageorges A Atanassova R Léon C Renaudin JP Isogene specific oligo arrays reveal multifaceted changes in gene expression during grape berry (*Vitis vinifera *L.) development Planta 2005 222 5 832 847 10.1007/s00425-005-0017-y 16151847

[B25] Cramer GR Ergül A Grimplet J Tillett RL Tattersall EAR Bohlman MC Vincent D Sonderegger J Evans J Osborne C Water and salinity stress in grapevines: early and late changes in transcript and metabolite profiles Funct Integr Genomics 2007 7 2 111 134 10.1007/s10142-006-0039-y 17136344

[B26] Espinoza C Vega A Medina C Schlauch K Cramer G Arce-Johnson P Gene expression associated with compatible viral diseases in grapevine cultivars Funct Integr Genomics 2007 7 2 95 110 10.1007/s10142-006-0031-6 16775684

[B27] Figueiredo A Fortes AM Ferreira S Sebastiana M Choi YH Sousa L Acioli-Santos B Pessoa F Verpoorte R Pais MS Transcriptional and metabolic profiling of grape (*Vitis vinifera *L.) leaves unravel possible innate resistance against pathogenic fungi J Exp Bot 2008 59 12 3371 3381 10.1093/jxb/ern187 18648103

[B28] Thimm O Blasing O Gibon Y Nagel A Meyer S Kruger P Selbig J Muller LA Rhee SY Stitt M MAPMAN: a user-driven tool to display genomics data sets onto diagrams of metabolic pathways and other biological processes Plant J 2004 37 6 914 939 10.1111/j.1365-313X.2004.02016.x 14996223

[B29] Green R Fluhr R UV-B-induced PR-1 accumulation is mediated by active oxygen species Plant Cell 1995 7 2 203 212 10.1105/tpc.7.2.203 12242373 PMC160776

[B30] Ballaré CL Rousseaux MC Searles PS Zaller JG Giordano CV Robson TM Caldwell MM Sala OE Scopel AL Impacts of solar ultraviolet-B radiation on terrestrial ecosystems of Tierra del Fuego (southern Argentina). An overview of recent progress J Photochem Photobiol B: Biol 2001 62 67 77 10.1016/S1011-1344(01)00152-X 11693368

[B31] Jeandet P Douillet-Breuil AC Bessis R Debord S Sbaghi M Adrian M Phytoalexins from the *Vitaceae*: biosynthesis, phytoalexin gene expression in transgenic plants, antifungal activity, and metabolism J Agric Food Chem 2002 50 10 2731 2741 10.1021/jf011429s 11982391

[B32] Langcake P Pryce RJ A new class of phytoalexins from grapevines Experientia 1977 33 2 151 152 10.1007/BF02124034 844529

[B33] Lijavetzky D Almagro L Belchi-Navarro S Martínez-Zapater JM Bru R Pedreno MA Synergistic effect of methyljasmonate and cyclodextrin on stilbene biosynthesis pathway gene expression and resveratrol production in Monastrell grapevine cell cultures BMC Res Notes 2008 1 132 10.1186/1756-0500-1-132 19102745 PMC2628674

[B34] Tang K Zhan JC Yang HR Huang WD Changes of resveratrol and antioxidant enzymes during UV-induced plant defense response in peanut seedlings J Plant Physiol 2010 167 2 95 102 10.1016/j.jplph.2009.07.011 19716623

[B35] Jansen MAK Ultraviolet-B radiation effects on plants: induction of morphogenic responses Physiol Plant 2002 116 3 423 429 10.1034/j.1399-3054.2002.1160319.x

[B36] Ludwig AA Saitoh H Felix G Freymark G Miersch O Wasternack C Boller T Jones JDG Romeis T Ethylene-mediated cross-talk between calcium-dependent protein kinase and MAPK signaling controls stress responses in plants Proc Natl Acad Sci USA 2005 102 30 10736 10741 10.1073/pnas.0502954102 16027369 PMC1176231

[B37] Park JM Park CJ Lee SB Ham BK Shin R Paek KH Overexpression of the tobacco Tsi1 gene encoding an EREBP/AP2-type transcription factor enhances resistance against pathogen attack and osmotic stress in tobacco Plant Cell 2001 13 5 1035 1046 10.1105/tpc.13.5.1035 11340180 PMC135557

[B38] Hao D Ohme-Takagi M Sarai A Unique mode of GCC box recognition by the DNA-binding domain of ethylene-responsive element-binding factor (ERF domain) in plants J Biol Chem 1998 273 41 26857 26861 10.1074/jbc.273.41.26857 9756931

[B39] Chervin C El-Kereamy A Roustan JP Latché A Lamon J Bouzayen M Ethylene seems required for the berry development and ripening in grape, a non-climacteric fruit Plant Sci 2004 167 6 1301 1305 10.1016/j.plantsci.2004.06.026

[B40] Olsen AN Ernst HA Leggio LL Skriver K DNA-binding specificity and molecular functions of NAC transcription factors Plant Sci 2005 169 4 785 797 10.1016/j.plantsci.2005.05.035 15708345

[B41] Olsen AN Ernst HA Leggio LL Skriver K NAC transcription factors: structurally distinct, functionally diverse Trends Plant Sci 2005 10 79 87 10.1016/j.tplants.2004.12.010 15708345

[B42] Eulgem T Rushton PJ Robatzek S Somssich IE The WRKY superfamily of plant transcription factors Trends Plant Sci 2000 5 5 199 206 10.1016/S1360-1385(00)01600-9 10785665

[B43] Eulgem T Somssich IE Networks of WRKY transcription factors in defense signaling Curr Opin Plant Biol 2007 10 4 366 371 10.1016/j.pbi.2007.04.020 17644023

[B44] Seki M Narusaka M Ishida J Nanjo T Fujita M Oono Y Kamiya A Nakajima M Enju A Sakurai T Monitoring the expression profiles of 7000 Arabidopsis genes under drought, cold and high-salinity stresses using a full-length cDNA microarray Plant J 2002 31 3 279 292 10.1046/j.1365-313X.2002.01359.x 12164808

[B45] Babosha AV Inducible lectins and plant resistance to pathogens and abiotic stress Biochem (Moscow) 2008 73 7 812 825 10.1134/S0006297908070109 18707590

[B46] Mishkind M Keegstra K Palevitz BA Distribution of wheat germ agglutinin in young wheat plants Plant Physiol 1980 66 950 955 10.1104/pp.66.5.950 16661559 PMC440759

[B47] Hoson T Masuda Y Concanavalin A inhibits auxin-induced elongation and breakdown of (1-3), (1-4) beta-D-glucans in segments of rice coleoptiles Plant Cell Physiol 1995 36 517 523

[B48] Bhaglal P Singh P Bhullar SS Kumar S Drought stress induced accumulation of wheat germ agglutinin in the developing embryos of wheat (*Triticum aestivum *L.) and its likely role in vivo J Plant Physiol 1998 153 163 166

[B49] Swindell WR Huebner M Weber AP Transcriptional profiling of Arabidopsis heat shock proteins and transcription factors reveals extensive overlap between heat and non-heat stress response pathways BMC genomics 2007 8 1 125 10.1186/1471-2164-8-125 17519032 PMC1887538

[B50] Gurley WB HSP101: a key component for the acquisition of thermotolerance in plants Plant Cell 2000 12 4 457 460 10.1105/tpc.12.4.457 10760235 PMC526003

[B51] Miller G Mittler R Could heat shock transcription factors function as hydrogen peroxide sensors in plants? Ann Bot 2006 98 2 279 288 10.1093/aob/mcl107 16740587 PMC2803459

[B52] Britt AB DNA damage and repair in plants Annu Rev Plant Biol 1996 47 1 75 100 10.1146/annurev.arplant.47.1.75 15012283

[B53] Molinier J Oakeley EJ Niederhauser O Kovalchuk I Hohn B Dynamic response of plant genome to ultraviolet radiation and other genotoxic stresses Mutat Res 2005 571 1-2 235 247 15748650 10.1016/j.mrfmmm.2004.09.016

[B54] Hirt H Mink M Pfosser M Bogre L Gyorgyey J Jonak C Gartner A Dudits D Heberle-Bors E Alfalfa cyclins: differential expression during the cell cycle and in plant organs Plant Cell 1992 4 12 1531 1538 10.1105/tpc.4.12.1531 1307238 PMC160239

[B55] Kinoshita T Doi M Suetsugu N Kagawa T Wada M Shimazaki K Phot1 and phot2 mediate blue light regulation of stomatal opening Nature 2001 414 656 660 10.1038/414656a 11740564

[B56] Sakai T Kagawa T Kasahara M Swartz TE Christie JM Briggs WR Wada M Okada K Arabidopsis nph1 and npl1: blue light receptors that mediate both phototropism and chloroplast relocation Proc Natl Acad Sci USA 2001 98 6969 6974 10.1073/pnas.101137598 11371609 PMC34462

[B57] Inada S Ohgishi M Mayama T Okada K Sakai T RPT2 Is a signal transducer involved in phototropic response and stomatal opening by association with phototropin 1 in *Arabidopsis thaliana* Plant Cell 2004 16 887 896 10.1105/tpc.019901 15031408 PMC412863

[B58] Inouea SI Kinoshitaa T Takemiyaa A Doib M Shimazakia KI Leaf positioning of Arabidopsis in response to blue Light Mol Plant 2008 1 1 15 26 10.1093/mp/ssm001 20031912

[B59] de Torres-Zabala M Truman W Bennett MH Lafforgue G Mansfield JW Egea PR Bögre L Grant M Pseudomonas syringae pv. tomato hijacks the Arabidopsis abscisic acid signalling pathway to cause disease EMBO J 2007 26 5 1434 10.1038/sj.emboj.7601575 17304219 PMC1817624

[B60] Zhu JK Salt and drought stress signal transduction in plants Annu Rev Plant Biol 2002 53 1 247 273 10.1146/annurev.arplant.53.091401.143329 12221975 PMC3128348

[B61] Liu Y Zhong ZC Interactive effects of a-NAA and UV-B radiation on the endogenous hormone contents and growth of *Trichosanthes kirilowii *Maxim seedlings Acta Ecologica Sinica 2009 29 244 248 10.1016/j.chnaes.2009.08.007

[B62] Pocock KF Hayasaka Y McCarthy M Waters EJ Thaumatin-like proteins and chitinases, the haze-forming proteins of wine, accumulate during ripening of grape (Vitis vinifera) berries and drought stress does not affect the final levels per berry at maturity J Agric Food Chem 2000 48 1637 1643 10.1021/jf9905626 10820071

[B63] Salzman RA Tikhonova I Bordelon BP Hasegawa PM Bressan RA Coordinate accumulation of antifungal proteins and hexoses constitutes a developmentally controlled defense response during fruit ripening in grape Plant Physiol 1998 117 465 472 10.1104/pp.117.2.465 9625699 PMC34966

[B64] Goes da Silva F Iandolino A Al-Kayal F Bohlmann MC Cushman MA Lim H Ergul A Figueroa R Kabuloglu EK Osborne C Characterizing the Grape Transcriptome. Analysis of Expressed Sequence Tags from Multiple Vitis Species and Development of a Compendium of Gene Expression during Berry Development Plant Physiol 2005 139 2 574 597 10.1104/pp.105.065748 16219919 PMC1255978

[B65] Beyer A Sequence analysis of the AAA protein family Protein Sci 1997 6 10 2043 2058 10.1002/pro.5560061001 9336829 PMC2143574

[B66] Ogura T Wilkinson AJ AAA+ superfamily ATPases: common structure--diverse function Genes to Cells 2001 6 7 575 597 10.1046/j.1365-2443.2001.00447.x 11473577

[B67] Wang D Pajerowska-Mukhtar K Culler AH Dong X Salicylic acid inhibits pathogen growth in plants through repression of the auxin signaling pathway Curr Biol 2007 17 1784 1790 10.1016/j.cub.2007.09.025 17919906

[B68] Bari R Jones JDG Role of plant hormones in plant defence responses Plant Mol Biol 2009 69 4 473 488 10.1007/s11103-008-9435-0 19083153

[B69] Murashige T Skoog F A revised medium for rapid growth and bioassays with tobacco tissue culture Physiol Plant 2000 15 473 497 10.1111/j.1399-3054.1962.tb08052.x

[B70] National Center for Biotechnology Information http://www.ncbi.nlm.nih.gov/

[B71] Grape Genome Browser (12X) http://www.genoscope.cns.fr/externe/GenomeBrowser/Vitis/

[B72] Gene Expression Profile Analysis Suite (GEPAS) http://gepas.bioinfo.cipf.es/

[B73] Tarraga J Medina I Carbonell J Huerta-Cepas J Minguez P Alloza E Al-Shahrour F Vegas-Azcarate S Goetz S Escobar P GEPAS, a web-based tool for microarray data analysis and interpretation Nucleic Acids Res 2008 36 Web Server W308 314 10.1093/nar/gkn303 18508806 PMC2447723

[B74] BIOCONDUCTOR, an open source software for bioinformatics http://www.bioconductor.org/

[B75] Irizarry RA Hobbs B Collin F Beazer-Barclay YD Antonellis KJ Scherf U Speed TP Exploration, normalization, and summaries of high density oligonucleotide array probe level data Biostatistics 2003 4 2 249 264 10.1093/biostatistics/4.2.249 12925520

[B76] Bolstad BM Irizarry RA Astrand M Speed TP A comparison of normalization methods for high density oligonucleotide array data based on variance and bias Bioinformatics 2003 19 2 185 193 10.1093/bioinformatics/19.2.185 12538238

[B77] Valls J Grau M Sole X Hernandez P Montaner D Dopazo J Peinado MA Capella G Moreno V Pujana MA CLEAR-test: combining inference for differential expression and variability in microarray data analysis J Biomed Inform 2008 41 1 33 45 10.1016/j.jbi.2007.05.005 17597009

[B78] Schneider M Lane L Boutet E Lieberherr D Tognolli M Bougueleret L Bairoch A The UniProtKB/Swiss-Prot knowledgebase and its Plant Proteome Annotation Program J Proteomics 2009 72 3 567 573 10.1016/j.jprot.2008.11.010 19084081 PMC2689360

[B79] Ashburner M Ball CA Blake JA Botstein D Butler H Cherry JM Davis AP Dolinski K Dwight SS Eppig JT Gene ontology: tool for the unification of biology. The Gene Ontology Consortium Nat Genet 2000 25 1 25 29 10.1038/75556 10802651 PMC3037419

[B80] Kanehisa M Goto S Kawashima S Okuno Y Hattori M The KEGG resource for deciphering the genome Nucleic acids research 2004 32 Database D277 10.1093/nar/gkh063 14681412 PMC308797

[B81] Cosgrove DJ Relaxation in a high-stress environment: The molecular bases of extensible cell walls and cell enlargement Plant Cell 1997 9 1031 1041 10.1105/tpc.9.7.1031 9254929 PMC156977

[B82] Lijavetzky D Almagro L Belchi-Navarro S Martinez-Zapater JM Bru R Pedreno MA Synergistic effect of methyljasmonate and cyclodextrin on stilbene biosynthesis pathway gene expression and resveratrol production in Monastrell grapevine cell cultures BMC Res Notes 2008 1 132 10.1186/1756-0500-1-132 19102745 PMC2628674

[B83] ArrayExpress database http://www.ebi.ac.uk/microarray-as/ae/

