# Supplementary material for: Transcriptome changes in grapevine (Vitis vinifera L.) cv. Malbec leaves induced by ultraviolet-B radiation
Source: BMC Plant Biol. 2010 Oct 20;10:224. doi: 10.1186/1471-2229-10-224 (PMC3017828; doi:10.1186/1471-2229-10-224)
Supplement: Additional file 1 — Full list of genes differentially expressed under high UV-B radiation. PDF file showing a complete list of the genes differentially expressed in the high UV-B treatment including Probe-set ID, Unique grapevine gene ID, Annotation and fold-change. [file 1471-2229-10-224-S1.PDF]

High UVB treatment

Probe set	Unique Gene ID	Annotation	Fold-change
VVTU11765_at	GSVIVP00004049001	AY670089 Vitis vinifera clone 325905_S3 stilbene synthase mRNA, partial cds.	57.9
VVTU9622_at	TC64430	Q0WYB9 Hypothetical protein related cluster	44.2
VVTU34551_x_at	GSVIVP00031875001	AY670148 Vitis vinifera clone 357851_M2 stilbene synthase mRNA, partial cds.	43.3
VVTU2839_at	GSVIVP00001453001	Q4AEC3 Cys2-His2 type Zinc finger protein related cluster	38.8
VVTU34913_at	AJ862932	AY670143 Vitis vinifera clone 357844_R1 stilbene synthase mRNA, partial cds.	34.3
VVTU2928_at	GSVIVP00021517001	Q9M663 Harpin inducinG protein related cluster	32.7
VVTU13781_s_at	GSVIVP00026228001	O80843 Hypothetical protein At2g45760 related cluster	31.0
VVTU759_at	GSVIVP00020989001	Q93Z27 AT5g39670 MIJ24_140 related cluster	30.0
VVTU9041_at	TC63592	Q75KH8 Hypothetical protein OJ1057_G07.4 related cluster	27.8
VVTU17051_at	TC69703	Q6H515 Hypothetical protein OSJNBa0073A21.9 related cluster	27.5
VVTU2102_at	GSVIVP00002706001	O64594 F17O7.4 related cluster	25.6
VVTU5257_x_at	GSVIVP00014231001	Q6RZW8 Putative ethylene response factor 4 related cluster	23.4
VVTU11030_at	GSVIVP00023009001	Q5ZEM1 Putative lectin 2 related cluster	22.8
VVTU5874_s_at	CF204867	Q8LC91 Hypothetical protein related cluster	22.6
VVTU14066_at	GSVIVP00022375001	Q6QB11 Little protein 1 related cluster	21.4
VVTU35958_s_at	CA814441	Q9FQY9 Avr9 Cf-9 rapidly elicited protein 75 related cluster	19.5
VVTU4088_s_at	TC70295	Q8GYA5 Hypothetical protein related cluster	19.0
VVTU14937_at	GSVIVP00020853001	Q9FQZ3 Avr9 Cf-9 rapidly elicited protein 231 precursor related cluster	17.8
VVTU25456_at	GSVIVP00020045001	Q2R227 Zinc finger, C3HC4 type family protein, expressed related cluster	17.7
VVTU2014_at	GSVIVP00014690001	Q8L3R2 calmodulin-like protein 41 related cluster	17.5
VVTU6727_at	GSVIVP00016518001	Q9LYH2 Hypothetical protein F14F18_180 related cluster	14.9
VVTU14989_at	CA816537	O22086 ZPT2-14 related cluster	14.7
VVTU13742_at	GSVIVP00014265001	Q6RZW8 Putative ethylene response factor 4 related cluster	14.7
VVTU12997_s_at	TC65008	Q1G3J0 Hypothetical protein related cluster	14.3
VVTU12705_s_at	GSVIVP00024561001	Q6L3H6 Adenylatekinase isoenzyme 6 , putative related cluster	14.1
VVTU8172_at	GSVIVP00009541001	O80337 ethylene-responsive transcription factor 1A related cluster	14.1
VVTU2527_at	GSVIVP00026904001	O82357 3-methyl-2-oxobutanoate hydroxy-methyl-transferase related cluster	13.2
VVTU35944_at	GSVIVP00037648001	Q6RZW9 Putative WRKY4 transcription factor related cluster	12.9
VVTU38576_at	GSVIVP00018787001	Q0JEB3 Os04g0303100 protein related cluster	12.9
VVTU7830_at	GSVIVP00029583001	Q9LJB7 Emb|CAA19725.1 related cluster	12.6
VVTU35718_at	GSVIVP00024738001	Q9LID5 Disease Resistance response protein-like related cluster	12.6
VVTU13742_s_at	GSVIVP00014263001	Q6RZW8 Putative ethylene response factor 4 related cluster	12.2
VVTU7250_s_at	GSVIVP00020826001	Q1S520 AAA ATPase related cluster	11.9
VVTU32983_x_at	CB341421	Q5M9R1 Hypothetical protein orf138c related cluster	11.8
VVTU19113_s_at	TC62328	Q7XYW1 seed specific protein Bn15D18B related cluster	11.4
VVTU15353_at	GSVIVP00017017001	Q9M4U0 Cinnamate 4-hydroxylase CYP73 related cluster	11.2
VVTU3375_at	GSVIVP00014947001	Q9XEU0 Zinc-finger protein 1 related cluster	11.2
VVTU1951_at	GSVIVP00025595001	Q7XI29 Hypothetical protein P0506C07.1 related cluster	11.2
VVTU12901_at	GSVIVP00010117001	DQ366302 Vitis vinifera resveratrol synthase (STS2) mRNA, complete cds.	11.1
VVTU39708_s_at	GSVIVP00031477001	Q6VAB2 UDP-glycosyltransferase 71B1 related cluster	11.0
VVTU28485_at	GSVIVP00014244001	Q6RZW8 Putative ethylene response factor 4 related cluster	10.8
VVTU2511_at	GSVIVP00013642001	Q6ZD72 Hypothetical protein P0450B04.17 related cluster	10.8
VVTU6420_at	GSVIVP00038540001	Q941F6 Leucine-rich repeat receptor-like kinase F21M12.36 related cluster	10.7
VVTU33059_s_at	GSVIVP00015213001	O82161 Phi-1 protein related cluster	10.6
VVTU28418_at	GSVIVP00016194001	Q1S9M3 Lipase, active site related cluster	10.6
VVTU3094_at	TC68360	Q8RXY3 Hypothetical protein At5g10695 related cluster	10.2
VVTU31475_at	CB341953	Q10EL1 Zinc finger C-x8-C-x5-C-x3-H type family protein, expressed related cluster	10.0
VVTU2469_at	GSVIVP00029153001	Q9AR56 Putative membrane protein related cluster	9.7
VVTU11587_at	GSVIVP00028163001	O49020 Myb-like DNA-binding domain protein related cluster	9.5
VVTU21823_at	GSVIVP00016657001	Q1SND5 Protein kinase related cluster	9.5
VVTU14627_at	GSVIVP00014754001	Q9FZ13 Tuber-specific and sucrose-responsive element binding factor related cluster	9.4
VVTU6018_at	GSVIVP00005362001	Q9SGP6 F3M18.8 related cluster	9.2
VVTU27967_at	GSVIVP00001320001	Q93X02 Putative ammonium transporter AMT2 related cluster	9.1
VVTU3968_s_at	TC57893	Q9FLD2 Arabidopsis thaliana genomic DNA, chromosome 5, TAC clone:K18I23 related cluster	8.9
VVTU3091_at	GSVIVP00003524001	Q9ZSD5 Syntaxin-related protein Nt-syr1 related cluster	8.7
VVTU9962_at	GSVIVP00020821001	Q1S528 AAA ATPase related cluster	8.6
VVTU9420_at	GSVIVP00006193001	Q9LD82 Putative heme bindinG protein 2 related cluster	8.4
VVTU1866_at	GSVIVP00006872001	Q0JP37 Os01g0246700 protein related cluster	8.4
VVTU35437_at	GSVIVP00014260001	Q6RZW8 Putative ethylene response factor 4 related cluster	8.3
VVTU22537_at	GSVIVP00007523001	P93392 S25-XP1 DNA bindinG protein related cluster	8.2
VVTU28352_at	GSVIVP00024745001	Q9C523 dirigent protein, putative related cluster	8.0
VVTU5290_at	GSVIVP00017370001	Q1SXN3 Harpin-induced 1 related cluster	8.0
VVTU6040_x_at	GSVIVP00013930001	X75967 V.vinifera PAL mRNA for phenylalanine ammonia lyase.	8.0
VVTU22872_at	GSVIVP00004742001	Q8VX50 Putative receptor-like serine-threonine protein kinase related cluster	7.9
VVTU1315_at	TC61492	Q9M4H3 Putative Metallothionein-like protein related cluster	7.9
VVTU40324_at	GSVIVP00014328001	Q6ZD72 Hypothetical protein P0450B04.17 related cluster	7.8
VVTU11112_at	VVTU11112_at	Q8LGJ9 Hypothetical protein related cluster	7.7
VVTU2377_at	GSVIVP00009741001	Q2HTZ5 At1g61340 related cluster	7.5
VVTU22090_at	GSVIVP00021134001	Q9LHJ2 UDP-glucose Glucosyltransferase-like protein related cluster	7.5
VVTU29284_at	GSVIVP00011194001	Q8S8Z5 Syringolide-induced protein B13-1-1 related cluster	7.4
VVTU14440_at	GSVIVP00023927001	O04682 Pathogenesis-related genes transcriptional activator PTI6 related cluster	7.4
VVTU16103_at	GSVIVP00015343001	Q2PHJ7 flavonol synthase related cluster	7.4
VVTU1920_at	GSVIVP00020018001	AY156051 Vitis vinifera putative alanine acetyl transferase (AAT) mRNA, partial cds.	7.3
VVTU3104_at	GSVIVP00009608001	O80432 Mitochondrial small Heat shock protein related cluster	7.3
VVTU15680_at	GSVIVP00020726001	Q8LDZ7 Cinnamoyl CoA reductase-like protein related cluster	7.3

Probe set	Unique Gene ID	Annotation	Fold-change
VVTU13641_at	GSVIVP00020000001	P09444 lateembryogenesis abundant protein D-34 related cluster	7.2
VVTU8153_at	GSVIVP00017555001	Q9C9B0 Putative Glucosyltransferase; 88035-86003 related cluster	7.1
VVTU876_at	GSVIVP00029253001	Q9FQ21 Putative Hs1pro-1-like receptor related cluster	7.1
VVTU2850_at	GSVIVP00019287001	Q6RH27 NAC domain protein related cluster	7.1
VVTU1581_s_at	TC53821	Q9LW49 ethylene-responsive transcription factor 4 related cluster	7.1
VVTU9258_at	TC60871	Q9FKM0 Arabidopsis thaliana genomic DNA, chromosome 5, P1 clone:MUA2 related cluster	7.0
VVTU15081_at	GSVIVP00036272001	Q8LEZ7 Hypothetical protein related cluster	6.9
VVTU9329_at	GSVIVP00035949001	Q76DY0 AG-motif binding protein-4 related cluster	6.6
VVTU1998_at	GSVIVP00033242001	Q1RW45 Hypothetical protein related cluster	6.4
VVTU33497_at	CA809237	Q0JBV4 Os04g0508200 protein related cluster	6.4
VVTU5830_at	GSVIVP00016176001	Q1S9M3 Lipase, active site related cluster	6.3
VVTU24019_at	GSVIVP00032350001	Q8GZN1 Pollen-specific calmodulin-bindinG protein related cluster	6.3
VVTU21514_x_at	GSVIVP00024741001	Q9LID5 Disease Resistance response protein-like related cluster	6.3
VVTU38062_at	TC64171	Q4X953 Hypothetical protein related cluster	6.2
VVTU11871_s_at	GSVIVP00025506001	AF305093 Vitis vinifera polygalacturonase inhibitinG protein mRNA, complete cds.	6.2
VVTU10579_at	GSVIVP00015549001	Q43537 ORF related cluster	6.2
VVTU709_at	GSVIVP00036931001	Q52QR5 NAC domain protein NAC1 related cluster	6.1
VVTU11054_at	GSVIVP00029466001	Q9M6E1 DNA-bindinG protein 3 related cluster	6.1
VVTU31365_at	CB342852	Q6RZW8 Putative ethylene response factor 4 related cluster	6.1
VVTU33818_at	GSVIVP00009607001	O80432 Mitochondrial small Heat shock protein related cluster	6.1
VVTU39783_at	GSVIVP00022086001	Q6JJ29 Prephenate dehydratase related cluster	6.1
VVTU16229_at	GSVIVP00031394001	Q9LLC2 Xyloglucan endotransglycosylase XET2 related cluster	6.1
VVTU26285_at	GSVIVP00013936001	O80406 Phenylalanine ammonia-lyase related cluster	6.0
VVTU9947_at	GSVIVP00017829001	Q8VZG8 AT4g08850 T32A17_160 related cluster	5.9
VVTU14975_s_at	GSVIVP00013875001	Q6E593 Benzoyl coenzyme A: benzyl alcohol benzoyl transferase related cluster	5.9
VVTU13727_at	GSVIVP00016280001	Q9CA77 Putative receptor protein kinase; 10992-14231 related cluster	5.8
VVTU21831_at	VVTU21831_at	Q1RZK4 Hypothetical protein related cluster	5.7
VVTU15006_at	GSVIVP00014758001	Q2V454 Protein At2g26530 related cluster	5.7
VVTU27091_at	CF414894	Q9FQZ5 Avr9 Cf-9 rapidly elicited protein 169 related cluster	5.7
VVTU38545_at	GSVIVP00017721001	Q8VYG0 Hypothetical protein At5g38210 related cluster	5.7
VVTU39122_at	GSVIVP00000755001	Q9SB74 Hypothetical protein F28M20.240 related cluster	5.6
VVTU17005_at	GSVIVP00014683001	Q1S566 GNS1 SUR4 membrane protein related cluster	5.6
VVTU25337_at	GSVIVP00002450001	Q1SEE9 UDP-glucuronosyl UDP-Glucosyltransferase related cluster	5.6
VVTU34812_s_at	GSVIVP00031317001	Q9SXP4 DNA-bindinG protein NtWRKY3 related cluster	5.6
VVTU7456_at	GSVIVP00025108001	Q9FFD0 Putative auxin efflux carrier component 8 related cluster	5.6
VVTU1780_s_at	GSVIVP00029187001	O82615 T9A4.6 protein related cluster	5.5
VVTU5584_at	GSVIVP00031406001	Q8S902 Syringolide-induced protein 19-1-5 related cluster	5.5
VVTU40342_at	TC55783	Q1S379 auxin responsive SAUR protein related cluster	5.5
VVTU3517_at	GSVIVP00015738001	Q1T6L3 Ribosome-binding factor A related cluster	5.5
VVTU32121_at	CB349010	P48502 Ubiquinol-cytochrome c reductase complex 14 kDa protein related cluster	5.5
VVTU34740_s_at	GSVIVP00029302001	Q9XEE6 Hypothetical Cys-3-His Zinc finger protein related cluster	5.4
VVTU1524_at	TC64418	Q1SBD7 Hypothetical protein related cluster	5.4
VVTU10418_at	VVTU10418_at	Q5ZA84 Embryogenesis transmembrane protein-like related cluster	5.4
VVTU33588_at	GSVIVP00007928001	Q8VYV9 Putative chloroplast nucleoid DNA-bindinG protein related cluster	5.3
VVTU24739_at	GSVIVP00037835001	O64757 Putative Disease Resistance protein related cluster	5.3
VVTU26819_at	GSVIVP00029666001	O82140 Beta-Amyrin Synthase related cluster	5.3
VVTU6661_at	GSVIVP00005104001	Q1RVF4 dirigent-like protein related cluster	5.3
VVTU11329_at	GSVIVP00030027001	UPI000034F3E8 Cluster related to UPI000034F3E8; catalytic hydrolase	5.3
VVTU38966_at	TC62971	O23757 Reverse transcriptase related cluster	5.3
VVTU31508_at	CB341745	Q1S312 Quinoprotein amine dehydrogenase, beta chain-like related cluster	5.2
VVTU3717_at	GSVIVP00027568001	Q9LSQ8 Arabidopsis thaliana genomic DNA, chromosome 5, BAC clone:F24B18 related cluster	5.2
VVTU8591_at	GSVIVP00033187001	Q6TN17 Gibberellin 2-oxidase related cluster	5.1
VVTU9572_at	GSVIVP00032363001	Q9ZQ28 Putative auxin-regulated protein related cluster	5.1
VVTU2373_at	GSVIVP00034024001	Q94EN7 Small heat stress protein class CIII related cluster	5.0
VVTU2860_at	GSVIVP00027730001	Q6TKQ3 Putative ethylene response factor ERF3b related cluster	5.0
VVTU7470_at	GSVIVP00029825001	Q1S4Y7 Berberine and berberine like, putative related cluster	5.0
VVTU20769_at	GSVIVP00018101001	Q9LMV7 F5M15.18 related cluster	4.9
VVTU37609_at	GSVIVP00029823001	Q3E9Y2 Protein At4g20830 related cluster	4.9
VVTU2645_at	GSVIVP00031383001	O24145 4-coumarate--CoA ligase 1 related cluster	4.9
VVTU37513_at	GSVIVP00021999001	Q5DMW5 MRGH12 related cluster	4.9
VVTU16393_at	GSVIVP00037297001	Q2I314 Ribulose-1,5-bisphophate carboxylase oxygenase small subunit related cluster	4.9
VVTU2514_at	GSVIVP00029304001	Q0GIK4 Cys-3-His Zinc finger protein related cluster	4.9
VVTU2502_at	TC66625	Q9SX33 Putative phospholipid-transporting ATPase 9 related cluster	4.9
VVTU13421_at	GSVIVP00038842001	Q1S529 RNA-directed DNA polymerase(Reverse transcriptase); Endonuclease exonuclease phosphatase related cluster	4.9
VVTU2063_at	TC58313	Q6KBB0 Putative inorganic pyrophosphatase related cluster	4.9
VVTU31097_s_at	TC53372	Q2HUL7 Integrase, catalytic region; Zinc finger, CCHC-type related cluster	4.8
VVTU4107_at	GSVIVP00028179001	Q1SSW0 Protein phosphatase 2C related cluster	4.8
VVTU7778_at	GSVIVP00034070001	Q0PNH1 Cytochrome P450 related cluster	4.8
VVTU28268_at	CF074523	Q0UUC3 Hypothetical protein related cluster	4.8
VVTU2622_at	GSVIVP00015121001	Q2PEQ8 Putative mitochondrial dicarboxylatecarrier protein related cluster	4.8
VVTU9349_at	TC67083	Q337C0 IMP dehydrogenase GMP reductase domain containing protein, expressed related cluster	4.7
VVTU40391_at	TC68698	Q0UKB6 Predicted protein related cluster	4.6

Probe set	Unique Gene ID	Annotation	Fold-change
VVTU20931_at	VVTU20931_at	Q9LVB8 HSR203J protein-like protein related cluster	4.6
VVTU3248_at	GSVIVP00028564001	Q6K989 Putative ATP-dependent transporter related cluster	4.6
VVTU6523_at	GSVIVP00032242001	Q9XGS6 Cytosolic class II low molecular weight Heat shock protein related cluster	4.6
VVTU6382_at	GSVIVP00002541001	Q9SMH1 ACC synthase related cluster	4.5
VVTU32222_at	CB348262	O81362 Sulfite reductase related cluster	4.5
VVTU21899_at	GSVIVP00027054001	Q9M3B1 Hypothetical protein F2K15.70 related cluster	4.5
VVTU28443_x_at	CD799913	Q52W18 Replicase related cluster	4.5
VVTU11732_at	GSVIVP00000442001	Q9FN67 Gb|AAD56319.1 related cluster	4.5
VVTU33985_at	TC67922	Q9M510 Dicyanin related cluster	4.5
VVTU10306_at	GSVIVP00032404001	Q94LF5 Hypothetical protein related cluster	4.5
VVTU34286_at	BQ796990	Q0UIT1 Predicted protein related cluster	4.5
VVTU33920_at	CB003136	Q2HU07 Hypothetical protein related cluster	4.5
VVTU15608_at	GSVIVP00018812001	Q9SXK8 Heat shock factor related cluster	4.4
VVTU31924_at	CB350300	Q9LNC5 F9P14.8 protein related cluster	4.4
VVTU35551_s_at	GSVIVP00022807001	Q8LKS5 Long chain acyl-CoA synthetase 7 related cluster	4.4
VVTU40539_at	TC59731	Q0UEY3 Hypothetical protein related cluster	4.4
VVTU23360_at	DV223961	Q8H6Q8 CTV.20 related cluster	4.4
VVTU23936_at	DT030644	UPI00003C004D Cluster related to UPI00003C004D; PREDICTED: similar to centromere protein E	4.4
VVTU15742_at	CN545589	Q10M91 VQ motif family protein, expressed related cluster	4.4
VVTU29819_at	GSVIVP00033041001	Q0E7D3 Sucrose synthase related cluster	4.4
VVTU2863_at	GSVIVP00036239001	Q43537 ORF related cluster	4.4
VVTU23043_at	VVTU23043_at	Q6XWA6 Resistance protein Sorb5 related cluster	4.4
VVTU8280_at	GSVIVP00023746001	Q1RYG7 Plant lipid transfer protein Par allergen related cluster	4.3
VVTU32415_x_at	CB347102	Q9M4H3 Putative Metallothionein-like protein related cluster	4.3
VVTU10590_at	VVTU10590_at	Q4IIU9 Hypothetical protein related cluster	4.3
VVTU35597_at	VVTU35597_at	Q0WYB9 Hypothetical protein related cluster	4.3
VVTU16405_at	CD801282	UPI000034F181 Cluster related to UPI000034F181; aspartic-type endopeptidase pepsin A	4.3
VVTU40158_at	CB341993	Q1SDH7 RNA-directed DNA polymerase(Reverse transcriptase); Haem peroxidase, plant fungal bacterial related cluster	4.3
VVTU20262_at	VVTU20262_at	UPI000034F475 Cluster related to UPI000034F475; unknown protein	4.3
VVTU36296_at	GSVIVP00013372001	Q9ATW1 Cinnamyl alcohol dehydrogenase related cluster	4.3
VVTU2526_at	GSVIVP00027223001	Q0J0T5 Os09g0484800 protein related cluster	4.3
VVTU3937_at	GSVIVP00006920001	Q84RC3 Gibberellin 2-oxidase 1 related cluster	4.2
VVTU14456_at	GSVIVP00023703001	Q3KU27 Nectarin IV related cluster	4.2
VVTU40693_at	TC66801	Q17CQ1 Hypothetical protein related cluster	4.2
VVTU7906_at	GSVIVP00023866001	Q9SXS8 ethylene-responsive transcription factor 3 related cluster	4.2
VVTU29836_at	GSVIVP00008555001	Q0JBH6 Os04g0531500 protein related cluster	4.2
VVTU26356_at	GSVIVP00014325001	Q6ZD72 Hypothetical protein P0450B04.17 related cluster	4.1
VVTU2507_s_at	GSVIVP00025952001	Q5VLJ53-hydroxy-3-methylglutaryl oxidase related cluster	4.1
VVTU2849_at	GSVIVP00001464001	Q9LJN4 Beta-1,4-xylosidase related cluster	4.1
VVTU26850_at	GSVIVP00035638001	Q9MFE3 cytochrome c biogenesis protein related cluster	4.1
VVTU11089_at	GSVIVP00032805001	Q9M8L3 Hypothetical protein T21F11.22 related cluster	4.1
VVTU21246_at	GSVIVP00036945001	Q1SLN2 Calcium-binding EF-hand related cluster	4.1
VVTU19941_at	GSVIVP00018981001	Q8S7E5 Putative phragmoplastin related cluster	4.1
VVTU33015_at	CA814508	Q940V6 Heat shock transcription factor related cluster	4.0
VVTU228_at	GSVIVP00003425001	Q9SIT5 Putative Na H antiporter related cluster	4.0
VVTU10215_at	GSVIVP00008657001	Q9ZPX9 Putative caltrActin related cluster	4.0
VVTU2004_s_at	GSVIVP00029315001	P17639 EMB-1 protein related cluster	4.0
VVTU26668_at	CF517509	Q1T553 Reverse transcriptase (RNA-dependent DNA polymerase), putative related cluster	4.0
VVTU9490_s_at	GSVIVP00008846001	P25766 Ras-related protein RGP1 related cluster	4.0
VVTU21860_at	GSVIVP00017875001	Q7Y036 MutT-like protein related cluster	4.0
VVTU11532_at	VVTU11532_at	Q4I3D3 Hypothetical protein related cluster	4.0
VVTU10907_at	GSVIVP00037817001	Q0E3P6 Os02g0161700 protein related cluster	3.9
VVTU32112_x_at	CB349093	Q42428 Chitinase Ib related cluster	3.9
VVTU17388_at	GSVIVP00015361001	Q8LDL8 TINY-like protein related cluster	3.9
VVTU24508_at	DT011795	Q8W228 Cytochrome P450 related cluster	3.9
VVTU25740_at	GSVIVP00008529001	Q8RVP4 Bacterial-induced class III peroxidase related cluster	3.9
VVTU16509_at	GSVIVP00038131001	Q9FKR3 Similarity to salt-inducible protein related cluster	3.9
VVTU21912_at	GSVIVP00031599001	Q4ADU9 peroxidase related cluster	3.8
VVTU22385_x_at	GSVIVP00029189001	O82615 T9A4.6 protein related cluster	3.8
VVTU22766_at	GSVIVP00036302001	Q6DBQ1 At4g35390 related cluster	3.8
VVTU4581_at	GSVIVP00003018001	O04681 Pathogenesis-related genes transcriptional activator PTI5 related cluster	3.8
VVTU13759_at	GSVIVP00038581001	Q1RSY7 Allergen V5 Tpx-1 related related cluster	3.7
VVTU21976_x_at	GSVIVP00017761001	Q9FHB6 genomic DNA, chromosome 5, TAC clone:K24M7 related cluster	3.7
VVTU15154_s_at	GSVIVP00022182001	Q7XE25 Heavy metal-associated domain containing protein, expressed related cluster	3.7
VVTU609_at	GSVIVP00003294001	Q1SH60 Disease Resistance protein; AAA ATPase related cluster	3.7
VVTU12576_at	GSVIVP00019400001	Q9SJ02 Expressed protein related cluster	3.7
VVTU2504_s_at	GSVIVP00030663001	Q6TY49 Reductase 1 related cluster	3.7
VVTU4057_at	GSVIVP00007673001	Q2PAJ1 Putative Laccase related cluster	3.7
VVTU11268_at	VVTU11268_at	Q9M0C3 Hypothetical protein AT4g30370 related cluster	3.7
VVTU4133_s_at	GSVIVP00019488001	Q9SXK9 Heat shock factor related cluster	3.7
VVTU4934_at	GSVIVP00009604001	Q940R4 AT4g16560 dl4305c related cluster	3.7
VVTU2671_at	GSVIVP00010116001	AF274281 Vitis vinifera resveratrol synthase (RS1) mRNA, complete cds.	3.6
VVTU36467_at	GSVIVP00015444001	Q7XUT0 OSJNBb0086G13.12 protein related cluster	3.6

Probe set	Unique Gene ID	Annotation	Fold-change
VVTU21508_at	GSVIVP00003703001	Q9ZVN3 T22H22.1 protein related cluster	3.6
VVTU12306_at	GSVIVP00014228001	Q84T91 oleosin related cluster	3.6
VVTU5434_at	GSVIVP00021464001	UPI000034EE33 Cluster related to UPI000034EE33; carbohydrate transporter organic anion transporter sugar porter	3.6
VVTU13148_at	GSVIVP00018441001	Q9M2S4 Probable serine threonine-specific protein kinase related cluster	3.6
VVTU39358_at	GSVIVP00017705001	Q0JR08 Os01g0127700 protein related cluster	3.6
VVTU22478_at	GSVIVP00037770001	Q1S5Q1 Leucine-rich repeat; Leucine-rich repeat, cysteine-containing type related cluster	3.6
VVTU9828_at	GSVIVP00030478001	Q8H2B1 DnaJ-like protein related cluster	3.6
VVTU40463_at	TC66419	Q1SD84 Integrase, catalytic region related cluster	3.6
VVTU26310_s_at	GSVIVP00031885001	AY670213 Vitis vinifera clone 361641_M1 stilbene synthase mRNA, partial cds.	3.6
VVTU7676_at	GSVIVP00006413001	Q402G4 Hypothetical protein GmTDF-5 related cluster	3.6
VVTU36599_at	TC64235	Q9FE41 Oryza sativa (japonica cultivar-group) genomic DNA, chromosome 1, PAC clone:P0433F09 related cluster	3.6
VVTU25244_at	CX016669	UPI0000449A9A Cluster related to UPI0000449A9A; PREDICTED: similar to MGC53359 protein	3.6
VVTU24322_at	GSVIVP00031684001	Q9FLP6 Ubiquitin-like protein SMT3-like related cluster	3.5
VVTU35161_at	GSVIVP00002683001	Q9XQB4 Photosystem I reaction center subunit III related cluster	3.5
VVTU12860_at	GSVIVP00011638001	Q5I6D6 Sinapyl alcohol dehydrogenase-like protein related cluster	3.5
VVTU12478_at	GSVIVP00000649001	Q1T4Y2 VQ related cluster	3.5
VVTU12788_at	GSVIVP00034626001	Q7XB39 Class IV Chitinase related cluster	3.5
VVTU21613_at	GSVIVP00026935001	Q9SF29 Syntaxin-71 related cluster	3.5
VVTU30295_at	GSVIVP00034242001	UPI000034EDBF Cluster related to UPI000034EDBF; ATP binding protein binding protein kinase protein serine threonine kinase protein-tyrosine kinase	3.5
VVTU68_at	GSVIVP00038107001	Q0WW94 Kanadaptin-like protein related cluster	3.5
VVTU26251_at	CF606158	UPI00005BF91D Cluster related to UPI00005BF91D; PREDICTED: Hypothetical protein XP_598749	3.5
VVTU22155_at	GSVIVP00009530001	Q9FGM5 Gb|AAF25996.1 related cluster	3.5
VVTU4146_at	GSVIVP00030508001	Q1S8S7 Hypothetical protein related cluster	3.5
VVTU21935_at	GSVIVP00009149001	Q43587 PAR-1a protein related cluster	3.5
VVTU9645_at	GSVIVP00033458001	Q7XQN1 OSJNBa0089K21.5 protein related cluster	3.5
VVTU34474_at	GSVIVP00009744001	Q9S9T5 T32N4.4 protein related cluster	3.5
VVTU27312_at	CF405273	O80668 Expressed protein related cluster	3.5
VVTU20129_at	GSVIVP00036284001	Q0IZR6 Os09g0554000 protein related cluster	3.5
VVTU25260_at	CX016427	Q1S3W7 Hypothetical protein related cluster	3.5
VVTU566_at	GSVIVP00026043001	P17069 Isocitrate lyase related cluster	3.5
VVTU10864_at	GSVIVP00028143001	Q9SYJ2 Probable Glycerol-3-phosphate acyltransferase 3 related cluster	3.5
VVTU35455_at	VVTU35455_at	Q9FM83 Arabidopsis thaliana genomic DNA, chromosome 5, P1 clone:MCD7 related cluster	3.5
VVTU29102_at	CD012055	Q5PP70 At5g44450 related cluster	3.5
VVTU36387_at	TC57865	Q0J6S9 Os08g0271300 protein related cluster	3.4
VVTU5436_x_at	GSVIVP00037740001	Q84TK8 Hypothetical protein related cluster	3.4
VVTU36277_at	GSVIVP00034517001	Q8GU90 pleiotropic drug Resistance protein 3 related cluster	3.4
VVTU6288_at	GSVIVP00022846001	Q84MC0 Putative GPI-anchored protein At3g06035 precursor related cluster	3.4
VVTU14205_at	GSVIVP00030766001	Q1SDM5 Zinc finger, RING-type; RINGv related cluster	3.4
VVTU16497_at	TC55232	Q1SD84 Integrase, catalytic region related cluster	3.4
VVTU25111_at	DT004114	Q1S7J8 Cyclin-like F-box related cluster	3.4
VVTU11255_at	GSVIVP00015429001	Q1SJX6 Natural resistance-associated macrophage protein related cluster	3.4
VVTU5788_at	GSVIVP00016580001	Q1S019 Hypothetical protein related cluster	3.4
VVTU22839_at	GSVIVP00012684001	Q949G3 pleiotropic drug Resistance protein 1 related cluster	3.4
VVTU20403_at	VVTU20403_at	UPI00003C0762 Cluster related to UPI00003C0762; PREDICTED: similar to ribosomal protein L18 CG8615-PA	3.4
VVTU16581_at	GSVIVP00016120001	Q9XIJ5 T10O24.19 related cluster	3.4
VVTU22907_at	GSVIVP00020254001	Q9AVQ9 Phosphate transporter related cluster	3.4
VVTU23389_s_at	TC57708	Q1SIY6 Integrase, catalytic region; Zinc finger, CCHC-type related cluster	3.3
VVTU18717_at	VVTU18717_at	Q8S3L0 Peroxiredoxin related cluster	3.3
VVTU6307_at	GSVIVP00032438001	Q9LU58 Gb|AAF43949.1 related cluster	3.3
VVTU34029_at	GSVIVP00007568001	Q0ZCB7 Integrase related cluster	3.3
VVTU6196_at	GSVIVP00015262001	Q6Z8J0 Putative phosphoglycerate mutase related cluster	3.3
VVTU1077_at	GSVIVP00035097001	P25858 Glyceraldehyde-3-phosphate dehydrogenase, cytosolic related cluster	3.3
VVTU23703_at	GSVIVP00008556001	Q1S9U2 Protein kinase; Concanavalin A-like lectin Glucanase related cluster	3.3
VVTU4608_at	GSVIVP00025920001	Q2V3T9 Protein At3g21610 related cluster	3.3
VVTU24697_at	GSVIVP00034475001	Q8H6S3 NBS-LRR type Disease Resistance protein related cluster	3.3
VVTU33494_at	CA809289	Q8S8K8 Expressed protein related cluster	3.3
VVTU10857_at	GSVIVP00022728001	Q1RSM3 Calcium-binding EF-hand; Ferric reductase-like transmembrane component related cluster	3.3
VVTU8905_at	GSVIVP00031146001	Q8RXR4 Hypothetical protein At4g14750 related cluster	3.3
VVTU12304_at	BQ799314	Q5ZFS4 Thioredoxin-dependent peroxidase related cluster	3.3
VVTU1572_at	GSVIVP00002810001	Q6UA14 Fiber protein Fb25 related cluster	3.3
VVTU3202_at	GSVIVP00033629001	Q6ZXI3 Putative calmodulin-like protein related cluster	3.3
VVTU31626_at	CB345759	P93394 Uracil phosphoribosyltransferase related cluster	3.3
VVTU5931_at	GSVIVP00003135001	Q9LHE8 Arabidopsis thaliana genomic DNA, chromosome 3, P1 clone: MZE19 related cluster	3.3
VVTU26881_at	GSVIVP00016969001	Q1SG27 Mak10 subunit, NatC N(Alpha)-terminal acetyltransferase related cluster	3.3
VVTU14998_at	GSVIVP00034975001	Q94DE5 Putative betaine proline transporter related cluster	3.2
VVTU15531_at	GSVIVP00014915001	Q67UI9 Putative MADS-box protein related cluster	3.2

Probe set	Unique Gene ID	Annotation	Fold-change
VVTU31499_at	CB341814	Q1S312 Quinoprotein amine dehydrogenase, beta chain-like related cluster	3.2
VVTU2508_at	GSVIVP00023389001	Q9SXP4 DNA-bindinG protein NtWRKY3 related cluster	3.2
VVTU9364_at	TC55587	Q1SV12 Kunitz inhibitor ST1-like related cluster	3.2
VVTU7854_at	GSVIVP00021423001	Q9STG2 Copper transporter protein homolog related cluster	3.2
VVTU8151_at	GSVIVP00018450001	O22282 Expressed protein related cluster	3.2
VVTU35803_at	GSVIVP00021418001	Q9FQE5 glutathione S-transferase GST 13 related cluster	3.2
VVTU10873_at	VVTU10873_at	Q9FLQ1 Arabidopsis thaliana genomic DNA, chromosome 5, P1 clone:MCO15 related cluster	3.2
VVTU24686_at	GSVIVP00034443001	Q8H6R0 NBS-LRR type Disease Resistance protein related cluster	3.2
VVTU16575_at	GSVIVP00032006001	Q71RI6 Resistance protein related cluster	3.2
VVTU14346_s_at	GSVIVP00025925001	Q1S4G4 Hypothetical protein related cluster	3.2
VVTU52_at	GSVIVP00027396001	Q6T3R2 NDR1-like protein related cluster	3.2
VVTU487_at	GSVIVP00031380001	Q9FKM1 Gb|AAF08572.1 related cluster	3.2
VVTU15079_at	GSVIVP00025073001	Q9SW54 Hypothetical protein T11I11.40 related cluster	3.2
VVTU22257_at	GSVIVP00037808001	Q1RYJ4 Protein kinase; Curculin-like (Mannose-binding) lectin; Apple- like related cluster	3.2
VVTU21737_at	GSVIVP00016399001	Q2VEU3 Dehydration-responsive element bindinG protein 3 related cluster	3.2
VVTU5709_at	GSVIVP00021719001	Q9FFN2 Similarity to limonene cyclase related cluster	3.2
VVTU39906_at	GSVIVP00030446001	Q9C996 Putative proline-rich APG protein; 47176-45828 related cluster	3.2
VVTU35617_at	GSVIVP00014154001	Q6L974 GAG-POL related cluster	3.1
VVTU26945_at	CF512551	Q8GYD0 Hypothetical protein At1g61670 T13M11_2 related cluster	3.1
VVTU15768_at	GSVIVP00037558001	Q6F4D6 UDP-glucose Glucosyltransferase related cluster	3.1
VVTU40651_at	GSVIVP00030080001	Q9C507 PPR-repeat protein, putative related cluster	3.1
VVTU7427_at	GSVIVP00017622001	Q9M9A2 F27J15.21 related cluster	3.1
VVTU26707_at	TC71212	Q9LKG8 TIP related cluster	3.1
VVTU7639_s_at	GSVIVP00037700001	Q6V7U8 Putative anthocyanin permease related cluster	3.1
VVTU24660_at	GSVIVP00013069001	Q0ZDG6 lipoxygenase 1 related cluster	3.1
VVTU35955_x_at	DV941169	Q9SYL9 50S ribosomal protein L13, chloroplast precursor related cluster	3.1
VVTU36063_at	GSVIVP00001529001	Q1T1H3 Cadmium-transporting ATPase; ATPase, E1-E2 type related cluster	3.1
VVTU36215_at	TC58724	Q2QP36 Retrotransposon protein, putative, Ty1-copia subclass related cluster	3.1
VVTU17486_at	TC64515	Q1DYJ4 Hypothetical protein related cluster	3.1
VVTU13875_at	TC69900	UPI0000DA2A79 Cluster related to UPI0000DA2A79; PREDICTED: similar to CG13731-PA	3.1
VVTU35444_at	GSVIVP00019463001	Q84QZ3 Hypothetical protein OSJNBa0093I13.23 related cluster	3.1
VVTU10157_at	VVTU10157_at	Q6RF46 Hypothetical protein related cluster	3.1
VVTU39119_at	GSVIVP00032551001	Q1SYJ9 Hypothetical protein related cluster	3.1
VVTU368_at	GSVIVP00030622001	Q8W3C9 Putative receptor-like protein kinase related cluster	3.1
VVTU5990_at	GSVIVP00037164001	AF239740 Vitis vinifera caffeic acid O-methyltransferase mRNA, complete cds.	3.1
VVTU11577_at	VVTU11577_at	Q2V328 Protein At5g37474 related cluster	3.1
VVTU18000_at	TC52990	Q0ZHF0 Hypothetical protein related cluster	3.1
VVTU2139_at	TC56922	Q9LXJ1 Hypothetical protein F3C22_140 related cluster	3.1
VVTU37360_at	TC67737	Q5MAL3 Coat protein related cluster	3.1
VVTU30599_at	CB923289	Q1S5C0 Polynucleotidyl transferase, Ribonuclease H fold related cluster	3.1
VVTU8614_at	GSVIVP00031398001	Q8S902 Syringolide-induced protein 19-1-5 related cluster	3.1
VVTU35903_at	TC64868	Q6XZH4 Nematode resistance-like protein related cluster	3.1
VVTU3785_at	GSVIVP00018109001	Q84K31 Putative Zinc finger transcription factor related cluster	3.0
VVTU6768_at	GSVIVP00022492001	Q0IPV0 Os12g0166000 protein related cluster	3.0
VVTU22610_at	GSVIVP00034362001	Q6K7V7 Isochorismatase hydrolase-like related cluster	3.0
VVTU648_s_at	GSVIVP00038763001	DQ277694 Vitis vinifera cultivar Grand Noir transcription factor MybA (MybA) gene, MybA2-1 allele, partial cds.	3.0
VVTU21530_s_at	GSVIVP00032899001	Q7XBP8 Ubiquitin Actin fusion protein 2 related cluster	3.0
VVTU13620_at	GSVIVP00032630001	Q1S5C8 ATPase, AFG1 family, putative related cluster	3.0
VVTU38963_at	GSVIVP00032950001	Q850H6 Gag-pol Polyprotein related cluster	3.0
VVTU27402_at	GSVIVP00018564001	Q9ZT78 Hypothetical protein F9H3.13 related cluster	3.0
VVTU6595_s_at	GSVIVP00015077001	Q9FME9 Emb|CAB89373.1 related cluster	3.0
VVTU1957_at	DT030498	Q1S0B8 auxin responsive protein related cluster	3.0
VVTU17273_s_at	GSVIVP00016633001	O48628 Pyrophosphate-dependent phosphofructo-1-kinase related cluster	3.0
VVTU26856_at	CF514253	Q2QNF1 Retrotransposon protein, putative, unclassified related cluster	3.0
VVTU875_s_at	TC60860	Q259M7 H0723C07.4 protein related cluster	3.0
VVTU39698_at	GSVIVP00028783001	Q02096 polygalacturonase precursor related cluster	3.0
VVTU17473_at	GSVIVP00028257001	P55242 Glucose-1-phosphate adenylyltransferase large subunit 2, chloroplast precursor related cluster	3.0
VVTU38935_at	GSVIVP00006933001	Q1T3J4 Protein kinase; EPSP synthase related cluster	3.0
VVTU11661_at	GSVIVP00001056001	Q9LWA3 Subtilisin-like protease related cluster	3.0
VVTU38842_at	GSVIVP00006808001	Q9FI74 Mutator-like transposase-like protein related cluster	3.0
VVTU38412_at	GSVIVP00026569001	Q19PN8 TIR-NBS-LRR type Disease Resistance protein related cluster	3.0
VVTU25373_at	GSVIVP00024309001	Q6KAC4 Putative X1 related cluster	3.0
VVTU35016_s_at	GSVIVP00010602001	Q8LLR1 MADS-box protein 3 related cluster	3.0
VVTU22494_at	GSVIVP00020408001	Q5CCP3 glutathione S-transferase GST 18 related cluster	2.9
VVTU37192_at	GSVIVP00017518001	Q0WYC0 Hypothetical protein related cluster	2.9
VVTU39169_at	TC58498	O15788 CG1 related cluster	2.9
VVTU26958_at	GSVIVP00028100001	Q1RUT0 Zinc finger, RING-type; RINGv related cluster	2.9
VVTU31082_at	CB838213	Q144U6 Hypothetical protein related cluster	2.9
VVTU9822_at	GSVIVP00033642001	Q6K4U8 Putative RNA helicase related cluster	2.9
VVTU38991_at	TC56719	Q1SD84 Integrase, catalytic region related cluster	2.9
VVTU16719_at	TC64536	Q7XJE6 Metacaspase 1 related cluster	2.9
VVTU7992_s_at	TC66627	Q6JVN1 germin-like protein 3 related cluster	2.9
VVTU13775_at	GSVIVP00004134001	Q9LDL7 SCARECROW gene regulator-like related cluster	2.9

Probe set	Unique Gene ID	Annotation	Fold-change
VVTU26107_at	GSVIVP00033815001	Q9FE45 cytokininoxidase related cluster	2.9
VVTU40387_at	TC68944	UPI000034F094 Cluster related to UPI000034F094; Hypothetical protein At4g15180	2.9
VVTU25586_at	CN545722	Q01561 NADH-ubiquinone oxidoreductase chain 5 related cluster	2.9
VVTU15314_at	GSVIVP00002438001	Q9C7W2 AP2-containing DNA-binding protein; 51686-52693 related cluster	2.9
VVTU13918_at	GSVIVP00036506001	Q6AV32 Putative oxidoreductase related cluster	2.9
VVTU35585_at	GSVIVP00035122001	Q9STM6 Lipase-like protein related cluster	2.9
VVTU33048_at	GSVIVP00024211001	Q9ZW24 Putative glutathione S-transferase related cluster	2.9
VVTU8469_at	VVTU8469_at	Q94JW8 Squamosa promoter-binding-like protein 6 related cluster	2.9
VVTU15665_at	GSVIVP00029307001	Q8LK94 Chloroplast omega-3 desaturase related cluster	2.9
VVTU22784_at	VVTU22784_at	Q9LQR4 T4O12.25 related cluster	2.9
VVTU6348_at	GSVIVP00016910001	UPI000034F491 Cluster related to UPI000034F491; Hypothetical protein At1g52565	2.9
VVTU34064_at	CA818101	Q6VPE7 Putative Reverse transcriptase related cluster	2.9
VVTU24918_at	GSVIVP00035931001	Q84KZ2 Purple Acid phosphatase related cluster	2.9
VVTU10481_at	GSVIVP00010513001	Q949G9 HcrVf1 protein related cluster	2.9
VVTU30802_at	GSVIVP00026433001	Q1RU52 Disease Resistance protein; AAA ATPase related cluster	2.8
VVTU587_at	GSVIVP00036115001	Q1T694 D-galactoside L-rhamnose binding SUEL lectin; Galactose-binding like related cluster	2.8
VVTU20204_at	GSVIVP00013347001	Q30D02 Putative 3-deoxy-D-arabino-heptulosonate 7-phosphate synthase 3 related cluster	2.8
VVTU22492_at	GSVIVP00038547001	Q506K3 Squalene Monooxygenase related cluster	2.8
VVTU28474_at	GSVIVP00025241001	Q9SQN2 Hypothetical protein F19K16.31 related cluster	2.8
VVTU40210_at	GSVIVP00020069001	Q1RTM0 Tetratricopeptide-like helical related cluster	2.8
VVTU26386_at	CF605096	Q4IPJ5 Hypothetical protein related cluster	2.8
VVTU10757_at	VVTU10757_at	Q2HVP1 Hypothetical protein related cluster	2.8
VVTU21674_at	GSVIVP00010704001	Q40096 receptor protein kinase related cluster	2.8
VVTU38266_s_at	GSVIVP00035348001	Q1S4B7 TGF-beta receptor, type I II extracellular region; ABC transporter related related cluster	2.8
VVTU13564_s_at	GSVIVP00024944001	Q9FL02 Arabidopsis thaliana genomic DNA, chromosome 5, P1 clone:MUD21 related cluster	2.8
VVTU35405_at	GSVIVP00038100001	Q1KUU3 Hypothetical protein related cluster	2.8
VVTU23042_at	GSVIVP00035929001	Q4KU02 Purple Acid phosphatase related cluster	2.8
VVTU7937_at	GSVIVP00028041001	Q1RV35 Pathogenesis-related transcriptional factor and ERF related cluster	2.8
VVTU32098_at	CB349140	Q8L9Y1 ADP,ATP carrier-like protein related cluster	2.8
VVTU27760_at	GSVIVP00007714001	Q40489 Cyclin A-like protein related cluster	2.8
VVTU40465_at	GSVIVP00023110001	Q940P8 AT5g20890 F22D1_60 related cluster	2.8
VVTU31996_at	CB349848	O04545 F20P5.27 protein related cluster	2.8
VVTU6536_at	GSVIVP00030518001	Q9M4G9 Putative Ripening-related protein related cluster	2.8
VVTU35544_at	GSVIVP00021538001	Q1ZZ69 Secoisolariciresinol dehydrogenase related cluster	2.8
VVTU14251_at	TC66518	Q3ED39 Protein At1g32540 related cluster	2.8
VVTU38740_at	TC54628	Q1SS72 Integrase, catalytic region related cluster	2.8
VVTU16753_at	GSVIVP00018535001	O80638 nodulin-like protein related cluster	2.8
VVTU15030_at	GSVIVP00034555001	Q6Z6D9 Putative 6-4 photolyase related cluster	2.8
VVTU16815_at	GSVIVP00006100001	Q0WQ63 Putative sugar transporter related cluster	2.8
VVTU17347_at	TC67734	Q6XWA3 Resistance protein Tsu4 related cluster	2.8
VVTU24371_at	GSVIVP00017587001	Q9SUD4 Senescence-associated protein-like related cluster	2.8
VVTU37653_s_at	TC66034	Q67TV5 Nuclear transport factor 2 (NTF2)-like protein related cluster	2.8
VVTU3411_s_at	GSVIVP00015146001	Q7XV53 OSJNBa0086B14.2 protein related cluster	2.8
VVTU40457_at	TC63828	Q0TY36 Predicted protein related cluster	2.8
VVTU39682_at	TC66109	Q9JH31 ORF1 related cluster	2.8
VVTU39468_at	CB979639	Q1SSC3 Hypothetical protein related cluster	2.8
VVTU25971_at	GSVIVP00010314001	Q9LNA8 F5O11.10 related cluster	2.8
VVTU27447_at	GSVIVP00035038001	Q9AXG2 Regulator of gene silencing related cluster	2.8
VVTU30559_at	GSVIVP00003035001	Q6VCW5 Putative O-methyltransferase related cluster	2.8
VVTU36598_at	GSVIVP00035257001	Q5M9Y0 Hypothetical protein orf155 related cluster	2.8
VVTU20601_at	VVTU20601_at	UPI0000D57259 Cluster related to UPI0000D57259; PREDICTED: similar to CG6015-PA	2.8
VVTU5618_at	GSVIVP00017980001	Q94CD1 Putative N-hydroxycinnamoyl benzoyltransferase related cluster	2.8
VVTU6376_at	GSVIVP00017259001	Q2PF06 Putative hydroxymethylglutaryl-CoA lyase related cluster	2.8
VVTU15195_at	CB982913	Q9MAA7 Probable Gibberellin receptor GID1L1 related cluster	2.8
VVTU16208_at	GSVIVP00001496001	Q9M2I9 Hypothetical protein F9D24.180 related cluster	2.8
VVTU4379_at	GSVIVP00030602001	Q9ZRY7 RNA-directed RNA polymerase related cluster	2.7
VVTU40245_at	GSVIVP00006742001	Q58IJ5 UDP-D-glucose epimerase 2 related cluster	2.7
VVTU4427_at	TC57709	Q68AN1 BZip transcription factor related cluster	2.7
VVTU5095_at	GSVIVP00009618001	Q96569 L-lactate dehydrogenase related cluster	2.7
VVTU35584_s_at	GSVIVP00017878001	Q84XG6 Erwinia induced protein 2 related cluster	2.7
VVTU38786_at	TC62035	Q1SAS4 RNase H, putative related cluster	2.7
VVTU38504_at	TC68745	Q710T7 Gag-pol Polyprotein related cluster	2.7
VVTU40370_at	TC68907	Q5EN04 40S ribosomal protein S18-like protein related cluster	2.7
VVTU4328_s_at	GSVIVP00029295001	Q9LY50 Receptor kinase-like protein related cluster	2.7
VVTU20961_at	GSVIVP00037300001	Q42965 Nitrilase 4 related cluster	2.7
VVTU20514_at	GSVIVP00019397001	O65012 Cytochrome P450 78A4 related cluster	2.7
VVTU8264_at	GSVIVP00023306001	Q2MJ10 Cytochrome P450 monooxygenase CYP98A37 related cluster	2.7
VVTU13257_s_at	GSVIVP00000491001	Q0IYU2 Os10g0162100 protein related cluster	2.7
VVTU1753_at	TC67646	Q10FB7 Expressed protein related cluster	2.7
VVTU11227_at	GSVIVP00037364001	Q9FMQ6 Arabidopsis thaliana genomic DNA, chromosome 5, P1 clone:MWD9 related cluster	2.7
VVTU22595_at	VVTU22595_at	Q9LFS2 Hypothetical protein F1N13_160 related cluster	2.7

Probe set	Unique Gene ID	Annotation	Fold-change
VVTU34862_at	CB347897	Q42428 Chitinase Ib related cluster	2.7
VVTU2837_s_at	GSVIVP00009539001	Q6RZW7 Putative ethylene response factor 5 related cluster	2.7
VVTU40257_at	GSVIVP00033742001	Q9SX38 Putative Disease Resistance protein At1g50180 related cluster	2.7
VVTU23016_at	VVTU23016_at	Q9SH23 F2K11.25 related cluster	2.7
VVTU5774_at	GSVIVP00002724001	Q8L5Y9 Pantothenate kinase 2 related cluster	2.7
VVTU6205_at	CF404926	Q0DLC2 Os05g0110000 protein related cluster	2.7
VVTU37308_at	GSVIVP00031498001	Q6VAB2 UDP-glycosyltransferase 71E1 related cluster	2.7
VVTU5941_at	GSVIVP00016408001	Q9LMB2 T29M8.1 protein related cluster	2.7
VVTU33334_at	CA810723	Q2I314 Ribulose-1,5-bisphophate carboxylase oxygenase small subunit related cluster	2.7
VVTU29032_at	CD012238	Q1SD84 Integrase, catalytic region related cluster	2.7
VVTU324_at	GSVIVP00023638001	Q0DKT9 Os05g0143800 protein related cluster	2.7
VVTU16372_at	GSVIVP00003061001	Q9SGN6 F3M18.18 related cluster	2.7
VVTU22215_s_at	GSVIVP00029219001	Q0PJC1 Myb transcription factor Myb122 related cluster	2.7
VVTU4517_at	GSVIVP00017813001	Q40196 RAB11F related cluster	2.7
VVTU16354_at	GSVIVP00019372001	Q69X84 Putative beta-1,3-Glucanase related cluster	2.7
VVTU20635_at	VVTU20635_at	Q9ZR40 U2 snRNP auxiliary factor, large subunit related cluster	2.7
VVTU2433_at	GSVIVP00024282001	Q1SGH7 Zinc finger, RING-type; Thioredoxin-related related cluster	2.7
VVTU23521_at	GSVIVP00027031001	Q9FGB8 Emb|CAB71103.1 related cluster	2.7
VVTU26609_at	CF518413	Q1SD84 Integrase, catalytic region related cluster	2.7
VVTU2436_at	GSVIVP00008328001	Q9SUU4 Hypothetical protein F8B4.180 related cluster	2.7
VVTU6322_at	VVTU6322_at	Q84W66 Nuclear transcription factor Y subunit B-6 related cluster	2.7
VVTU19599_at	GSVIVP00032568001	Q0DFR7 Os05g0574700 protein related cluster	2.7
VVTU28782_at	CD714724	UPI00000A2CBF Cluster related to UPI00000A2CBF; P0408G07.7	2.7
VVTU7462_at	GSVIVP00011952001	Q6TPK4 PhytoCystatin related cluster	2.7
VVTU14413_at	GSVIVP00033009001	Q1M0P1 UDP-glucuronic acid decarboxylase 2 related cluster	2.6
VVTU28070_at	CF209169	Q1SRY2 Hypothetical protein related cluster	2.6
VVTU27407_at	CF404702	Q9FME8 Oligopeptide transporter 4 related cluster	2.6
VVTU15199_at	GSVIVP00012292001	Q9SWX8 receptor-like kinase CHRK1 related cluster	2.6
VVTU22894_at	GSVIVP00038246001	Q9C9H1 Putative Zinc finger protein; 21453-22187 related cluster	2.6
VVTU40036_at	GSVIVP00007643001	Q338J0 Pyridoxal-dependent decarboxylase conserved domain containing protein, expressed related cluster	2.6
VVTU25919_at	CF609321	Q1T2L2 Hypothetical protein related cluster	2.6
VVTU13872_at	GSVIVP00011453001	Q9FKQ1 Emb|CAB89401.1 related cluster	2.6
VVTU27285_at	CF405437	Q6AUG1 Hypothetical protein OSJNBb0053D02.10 related cluster	2.6
VVTU29113_at	GSVIVP00030081001	Q9ZQ74 Hypothetical protein At2g03380 related cluster	2.6
VVTU26125_at	GSVIVP00000627001	Q9LZE6 Hypothetical protein F12E4_170 related cluster	2.6
VVTU21167_at	GSVIVP00008637001	Q9LQR0 LOB domain-containinG protein 1 related cluster	2.6
VVTU40163_at	GSVIVP00021789001	Q6Q3H2 terpenoid synthetase related cluster	2.6
VVTU4467_s_at	TC58280	Q9FWQ7 F17F16.6 protein related cluster	2.6
VVTU5924_at	GSVIVP00024395001	Q8L8B6 Hypothetical protein At2g28690 T8O18.2 related cluster	2.6
VVTU26793_at	GSVIVP00038789001	Q6T3R3 Bacterial spot Disease Resistance protein 4 related cluster	2.6
VVTU37924_at	GSVIVP00009481001	Q5BQ17 Hypothetical protein related cluster	2.6
VVTU32448_at	CB346810	P10973 Nonspecific lipid-transfer protein A related cluster	2.6
VVTU15537_at	GSVIVP00000686001	Q9ZVC3 Putative Embryo-abundant protein related cluster	2.6
VVTU8490_at	GSVIVP00019036001	Q93Z79 AT5g24910 F6A4_120 related cluster	2.6
VVTU32825_x_at	CB344789	Q1RXY0 Hypothetical protein related cluster	2.6
VVTU30763_at	GSVIVP00024640001	UPI0000163499 Cluster related to UPI0000163499; Kinase-like protein	2.6
VVTU37085_at	GSVIVP00036459001	O48716 En Spm-like transposon protein related cluster	2.6
VVTU9835_at	GSVIVP00027018001	Q9SKD8 Expressed protein related cluster	2.6
VVTU39729_at	GSVIVP00030668001	Q6BDH2 Aldo-keto reductase related cluster	2.6
VVTU14454_at	GSVIVP00005756001	P49295 Glutamyl-tRNA reductase 2, chloroplast precursor related cluster	2.6
VVTU12127_s_at	GSVIVP00031388001	Q5K4H9 Putative membrane protein related cluster	2.6
VVTU10659_at	GSVIVP00018127001	Q9ZUV4 nodulin-like protein related cluster	2.6
VVTU20555_at	GSVIVP00016768001	Q41901 NADH ubiquinone oxidoreductase subunit related cluster	2.6
VVTU70_s_at	GSVIVP00038135001	Q8LAM9 Pollen coat-like protein related cluster	2.6
VVTU4093_at	TC65025	Q9C9V8 Hypothetical protein T23K23.23 related cluster	2.6
VVTU6887_at	GSVIVP00028914001	O48679 F3I6.5 protein related cluster	2.6
VVTU38458_at	GSVIVP00034601001	Q1SW19 Disease Resistance protein related cluster	2.6
VVTU9999_at	GSVIVP00030120001	Q655W3 RING Zinc finger protein-like related cluster	2.6
VVTU10835_at	VVTU10835_at	Q6RKN5 Glutamate receptor 3.1 precursor related cluster	2.6
VVTU14968_at	GSVIVP00014377001	Q1S8F9 Lipolytic enzyme, G-D-S-L related cluster	2.6
VVTU13165_at	GSVIVP00001048001	Q9M4G8 Putative Ripening-related P-450 enzyme related cluster	2.6
VVTU13299_at	GSVIVP00028448001	Q1SA57 Hypothetical protein related cluster	2.6
VVTU35516_s_at	DV220078	Q1SF40 Four F5 protein related cluster	2.6
VVTU10492_at	GSVIVP00018168001	O22143 Putative transketolase related cluster	2.6
VVTU25994_at	GSVIVP00020921001	Q2V3R6 Protein At3g28760 related cluster	2.6
VVTU12168_s_at	GSVIVP00018415001	P13905 elongation factor 1-alpha related cluster	2.6
VVTU10790_at	VVTU10790_at	Q4IFN8 Hypothetical protein related cluster	2.6
VVTU4318_at	DY473668	Q1EPJ3 DNA-binding WRKY domain-containinG protein related cluster	2.6
VVTU982_at	TC66741	Q14K84 Hypothetical protein related cluster	2.6
VVTU8201_at	GSVIVP00021433001	Q9FXS6 NtEIG-E80 protein related cluster	2.6
VVTU33024_at	CA814334	Q0V4W1 Hypothetical protein related cluster	2.6
VVTU36245_at	GSVIVP00012846001	Q43260 glutamate dehydrogenase related cluster	2.6
VVTU6580_s_at	GSVIVP00012763001	UPI0000E1F3EA Cluster related to UPI0000E1F3EA; PREDICTED: similar to RPS27A protein	2.6
VVTU2810_at	GSVIVP00009601001	Q39079 chaperone protein DnaJ 13 related cluster	2.6
VVTU32254_at	CB348040	Q9M4H3 Putative Metallothionein-like protein related cluster	2.6

Probe set	Unique Gene ID	Annotation	Fold-change
VVTU11543_at	GSVIVP00011105001	O81002 Putative Embryo-abundant protein related cluster	2.6
VVTU9265_at	GSVIVP00001190001	UPI000034F0B9 Cluster related to UPI000034F0B9; electron carrier	2.5
VVTU8785_at	GSVIVP00020633001	Q9LY84 Early nodule-specific protein-like related cluster	2.5
VVTU15513_at	GSVIVP00037696001	Q0WWD0 Hypothetical protein At4g25640 related cluster	2.5
VVTU8951_at	GSVIVP00031724001	Q1RYL4 serine threonine protein kinase, active site related cluster	2.5
VVTU23957_at	GSVIVP00038293001	Q19PN9 NBS type Disease Resistance protein related cluster	2.5
VVTU12806_at	GSVIVP00011451001	Q84KA9 RING C3HC4 PHD Zinc finger-like protein related cluster	2.5
VVTU13756_at	GSVIVP00002451001	Q9FQZ4 Avr9 Cf-9 rapidly elicited protein 194 related cluster	2.5
VVTU28772_at	CD715232	Q1SCY9 Integrase, catalytic region; Zinc finger, CCHC-type related cluster	2.5
VVTU35011_at	VVTU35011_at	Q963B6 60S ribosomal protein L10a related cluster	2.5
VVTU22002_at	GSVIVP00032596001	Q1S420 HCO3-transporter related cluster	2.5
VVTU40143_at	GSVIVP00010495001	Q9FFA7 Similarity to photomorphogenesis repressor protein related cluster	2.5
VVTU26741_at	CF516433	Q1SNS9 Integrase, catalytic region related cluster	2.5
VVTU11029_at	GSVIVP00011873001	Q45R28 Hypothetical protein related cluster	2.5
VVTU13580_s_at	GSVIVP00034590001	Q2HVW4 F9L1.35 protein-Arabidopsis thaliana related cluster	2.5
VVTU23746_at	DT035057	Q7XHQ4 Hypothetical protein P0565A07.109 related cluster	2.5
VVTU10975_at	GSVIVP00015367001	Q9SUF1 nodulin-like protein related cluster	2.5
VVTU39622_at	GSVIVP00021474001	Q8LLM2 AER related cluster	2.5
VVTU8229_at	GSVIVP00008737001	Q6ZH46 Hypothetical protein OJ1217_F02.5 related cluster	2.5
VVTU9148_at	TC63404	Q29Q81 At5g40460 related cluster	2.5
VVTU4731_at	GSVIVP00025337001	Q944B1 Beta-1,3-Glucanase related cluster	2.5
VVTU21895_at	GSVIVP00031443001	Q40289 Anthocyanidin 3-O-Glucosyltransferase related cluster	2.5
VVTU21843_at	GSVIVP00033339001	Q1SI93 E-class P450, group I related cluster	2.5
VVTU33659_at	CB340094	Q09G57 ribosomal protein S2 related cluster	2.5
VVTU8499_at	DY474310	Q9ZPY7 Importin-alpha re-exporter related cluster	2.5
VVTU14607_at	DY474945	Q9LW53 Arabidopsis thaliana genomic DNA, chromosome 3, P1 clone: MLM24 related cluster	2.5
VVTU34161_at	TC71208	Q6L3H4 Hypothetical protein related cluster	2.5
VVTU10918_at	VVTU10918_at	Q8GYU9 Hypothetical protein At4g14380 dl3230w related cluster	2.5
VVTU31231_at	GSVIVP00029924001	Q1SMG9 Extradiol ring-cleavage dioxygenase, class III enzyme, subunit B related cluster	2.5
VVTU25213_at	CX017204	Q29JD5 GA21535-PA related cluster	2.5
VVTU27214_at	CF405887	Q93XJ6 Glutamine synthetase related cluster	2.5
VVTU28802_at	CD713658	Q40392 TMV Resistance protein N related cluster	2.5
VVTU14620_at	GSVIVP00001853001	Q9ZWQ3 UDP-glycose:flavonoid glycosyltransferase related cluster	2.5
VVTU14035_at	GSVIVP00000341001	Q5VNK5 Hypothetical protein B1109A06.18 related cluster	2.5
VVTU31963_x_at	VVTU31963_x_at	P62577 Chloramphenicol acetyltransferase related cluster	2.5
VVTU37101_at	TC66641	Q9STX4 Hypothetical protein T22A6.30 related cluster	2.5
VVTU14429_at	TC63265	Q9ZWC0 F21M11.6 protein related cluster	2.5
VVTU25628_at	GSVIVP00006387001	Q8LD14 Hypothetical protein related cluster	2.5
VVTU34134_at	CA816164	Q2AA99 Gag-pol Polyprotein, related related cluster	2.5
VVTU22252_s_at	GSVIVP00018226001	Q9FRM3 Hypothetical protein OSJNBa0056G17.7 related cluster	2.5
VVTU6636_s_at	GSVIVP00022607001	Q9LEB7 Common plant regulatory factor 6 related cluster	2.5
VVTU40199_at	GSVIVP00023330001	Q4R0I0 Pinoresinol-lariciresinol reductase related cluster	2.5
VVTU39281_at	TC61634	Q6L975 GAG-POL related cluster	2.5
VVTU15626_s_at	GSVIVP00019769001	O04133 SRC2 related cluster	2.5
VVTU545_at	GSVIVP00011405001	Q93ZB9 AT4g33790 T16L1_280 related cluster	2.5
VVTU12948_at	GSVIVP00010265001	Q5HZ39 At4g27460 related cluster	2.5
VVTU35557_at	TC67429	Q9ARH3 Plastid-targeted protein 3 related cluster	2.5
VVTU26798_at	CF515675	Q1SWP8 ATP-requiring DNA helicase RecQ related cluster	2.5
VVTU7774_at	GSVIVP00011809001	Q66PF2 Putative UDP-rhamnose:rhamnosyltransferase related cluster	2.5
VVTU31722_at	CB345250	Q5XTQ8 Progesterone 5-beta-reductase related cluster	2.5
VVTU20679_at	GSVIVP00013567001	Q9SD07 Mucin-like protein related cluster	2.5
VVTU22174_at	GSVIVP00003000001	Q0D972 Os07g0103100 protein related cluster	2.5
VVTU20852_at	VVTU20852_at	Q1SBS4 Anthocyanin acyltransferase related cluster	2.4
VVTU22488_at	GSVIVP00000376001	Q1SCY9 Integrase, catalytic region; Zinc finger, CCHC-type related cluster	2.4
VVTU26941_at	GSVIVP00014711001	Q6QLL5 WAK-like kinase related cluster	2.4
VVTU35330_at	GSVIVP00025378001	Q9FNW2 seed maturation protein LEA 4 related cluster	2.4
VVTU31820_at	CB344665	Q1T5X1 Hypothetical protein related cluster	2.4
VVTU32400_at	CB347187	Q9M4H3 Putative Metallothionein-like protein related cluster	2.4
VVTU10458_at	GSVIVP00028125001	Q4PT42 invrtase related cluster	2.4
VVTU26625_x_at	CF518161	Q1T152 IMP dehydrogenase GMP reductase related cluster	2.4
VVTU31362_x_at	CB342902	Q9SWB5 seed maturation protein PM37 related cluster	2.4
VVTU38345_at	GSVIVP00027204001	Q5JMF1 Hypothetical protein P0512C01.34 related cluster	2.4
VVTU32092_at	GSVIVP00008142001	Q0KIN0 Integrase core domain containinG protein related cluster	2.4
VVTU11232_at	GSVIVP00037412001	Q9M009 Aldose reductase-like protein related cluster	2.4
VVTU36301_at	GSVIVP00035748001	Q2AA50 Retrotransposon gaG protein related cluster	2.4
VVTU15261_at	GSVIVP00030574001	Q0DTV2 Os03g0223000 protein related cluster	2.4
VVTU7966_at	CB915119	Q9FK33 Selenium-binding protein-like related cluster	2.4
VVTU2564_at	GSVIVP00008275001	Q6YXC5 Putative Zinc finger protein ID1 related cluster	2.4
VVTU33374_at	GSVIVP00036953001	Q2QS77 Retrotransposon protein, putative, unclassified related cluster	2.4
VVTU15185_at	GSVIVP00029640001	O64398 receptor-like protein kinase related cluster	2.4
VVTU28004_at	CF209946	Q1T5Q8 RNA-directed DNA polymeraserelated cluster	2.4
VVTU1839_s_at	GSVIVP00038077001	AF373601 Vitis vinifera MADS-box protein 2 (MADS2) mRNA, complete cds.	2.4
VVTU23743_at	DT035127	Q9SH73 F22C12.1 related cluster	2.4
VVTU31195_at	CB343557	Q9SX29 F24J5.10 protein related cluster	2.4
VVTU1905_x_at	GSVIVP00025581001	Q1SMG9 Extradiol ring-cleavage dioxygenase, class III enzyme, subunit B related cluster	2.4

Probe set	Unique Gene ID	Annotation	Fold-change
VVTU40016_at	TC58301	Q4WFC9 Hypothetical protein related cluster	2.4
VVTU28638_at	CD721664	Q2GW63 Hypothetical protein related cluster	2.4
VVTU21006_at	VVTU21006_at	Q1S4Z4 RNA-directed DNA polymeraserelated cluster	2.4
VVTU40516_at	TC54989	Q8IAW5 Hypothetical protein PF08_0083 related cluster	2.4
VVTU31751_at	CB345134	Q9RXQ4 Hypothetical protein related cluster	2.4
VVTU38313_at	GSVIVP00017917001	Q9SJF2 T27G7.8 related cluster	2.4
VVTU22481_at	GSVIVP00029809001	O49747 ERT2 protein related cluster	2.4
VVTU5317_s_at	GSVIVP00016915001	Q3E9A5 Protein At5g20190 related cluster	2.4
VVTU10494_at	GSVIVP00034367001	Q2HU50 Uncharacterized Cys-rich domain related cluster	2.4
VVTU10279_at	GSVIVP00002419001	Q8GVF0 Putative bHLH transcription factor related cluster	2.4
VVTU11835_at	GSVIVP00036846001	DQ979341 Vitis vinifera Flowering-related B-class MADS-box protein (TM6) mRNA, complete cds.	2.4
VVTU3958_s_at	GSVIVP00029424001	Q9LY04 Hypothetical protein T5P19_10 related cluster	2.4
VVTU10162_s_at	GSVIVP00025849001	Q0J0U0 Os09g0483500 protein related cluster	2.4
VVTU33918_at	CB003191	Q6L974 GAG-POL related cluster	2.4
VVTU37348_at	TC67508	P29149 RNA1 Polyprotein (P1) [Contains: P1A protein (1A) (protease cofactor); Putative ATP-dependent helicase (EC 3.6.1.-) (NTP-binding protein) (NTB) (1B) (Membrane-binding protein); Viral genome-linked protein (1C-VPg); Picornain 3C-like protease (EC 3.4.22.-) (3C-like protease) (1D-PRO); RNA-directed RNA polymerase (EC 2.7.7.48) (1E-POL)] related cluster	2.4
VVTU35304_at	GSVIVP00025127001	AY634281 Vitis viniferadehydrin mRNA, complete cds.	2.4
VVTU35409_at	GSVIVP00026902001	O04609 WRKY transcription factor 22 related cluster	2.4
VVTU25022_at	DT005129	Q9SD94 Hypothetical protein F13G24.100 related cluster	2.4
VVTU24464_at	GSVIVP00026768001	Q6XW51 Resistance protein RPP8-like protein related cluster	2.4
VVTU27972_at	GSVIVP00012707001	O82676 1-deoxyxylulose 5-phosphate synthase related cluster	2.4
VVTU22025_at	VVTU22025_at	Q9LYU3 Hypothetical protein T31B5_150 related cluster	2.4
VVTU36261_at	TC59194	Q0IT37 Os11g0420000 protein related cluster	2.4
VVTU20940_at	GSVIVP00002849001	Q8RW34 Beta 1,3-glycosyltransferase-like protein I related cluster	2.4
VVTU21945_at	GSVIVP00019304001	Q5ZF80 Amino acid permease related cluster	2.4
VVTU3021_at	TC59474	O81977 Rudimentary enhancer related cluster	2.4
VVTU40186_at	GSVIVP00016877001	Q676X4 ribosomal protein L7 related cluster	2.4
VVTU9687_at	GSVIVP00027228001	Q8S397 Sodium hydrogen exchanger 4 (Na(+) H(+) exchanger 4) related cluster	2.4
VVTU26964_at	CF512326	Q1S5B7 Hypothetical protein related cluster	2.4
VVTU38780_at	TC58047	Q1SFS4 RNA-directed DNA polymerase(Reverse transcriptase); Integrase, catalytic region; Ribonuclease H; Retrotransposon gaG protein related cluster	2.4
VVTU544_at	GSVIVP00024147001	P48422 Cytochrome P450 86A1 related cluster	2.4
VVTU15624_at	CV179321	Q84NN3 Putative DNA-directed RNA polymerase III subunit 22.9 kDa polypeptide related cluster	2.4
VVTU25448_at	CN548047	Q8LEN0 Hypothetical protein related cluster	2.4
VVTU27064_at	GSVIVP00037787001	Q1SK89 Hypothetical protein related cluster	2.4
VVTU10496_at	VVTU10496_at	UPI0000DA263B Cluster related to UPI0000DA263B; PREDICTED: Hypothetical protein	2.4
VVTU3310_s_at	GSVIVP00016789001	Q1SD74 Hypothetical protein related cluster	2.4
VVTU7240_at	GSVIVP00015883001	Q1T3X9 Response regulator, RegA PrrA ActR type related cluster	2.4
VVTU1391_at	GSVIVP00013348001	Q2PER8 Putative Histone deacetylase related cluster	2.4
VVTU39752_at	GSVIVP00030961001	Q6PWU1 terpenoid synthase related cluster	2.4
VVTU21217_at	VVTU21217_at	Q56WD9 3-ketoacyl-CoA thiolase 2, peroxisomal precursor related cluster	2.4
VVTU40564_at	GSVIVP00013141001	Q3EBZ2 Protein At2g17030 related cluster	2.4
VVTU38527_at	TC63202	Q7XE06 HAT family dimerisation domain containinG protein related cluster	2.4
VVTU6264_at	GSVIVP00034745001	Q68UW1 polygalacturonase related cluster	2.4
VVTU10863_at	VVTU10863_at	Q4HW03 Hypothetical protein related cluster	2.4
VVTU39025_at	GSVIVP00014818001	Q8LF37 Cytochrome P450, putative related cluster	2.4
VVTU31164_at	CB343788	P26969 Glycine dehydrogenase [decarboxylating], mitochondrial precursor related cluster	2.4
VVTU10919_at	GSVIVP00022687001	Q3E8Z8 Protein At5g27870 related cluster	2.3
VVTU3155_s_at	GSVIVP00038254001	Q4ACU1 Delta7 sterol C-5 desaturase related cluster	2.3
VVTU35637_at	GSVIVP00018316001	Q8S9A7 Glucosyltransferase-2 related cluster	2.3
VVTU38118_at	TC51865	Q1RTD3 IMP dehydrogenase GMP reductase related cluster	2.3
VVTU24557_at	GSVIVP00028200001	Q9M1Q4 Hypothetical protein T17J13.160 related cluster	2.3
VVTU2778_at	GSVIVP00006737001	Q1SI70 Phosphatidylinositol 3-and 4-kinase, catalytic; Ubiquitin related cluster	2.3
VVTU13841_s_at	TC65299	Q9ZS84 Polyprotein related cluster	2.3
VVTU20709_x_at	VVTU20709_x_at	Q8S9K1 At1g23880 T23E23_8 related cluster	2.3
VVTU30555_at	GSVIVP00009976001	P17840 S-locus-specific glycoprotein S13 precursor related cluster	2.3
VVTU38262_at	GSVIVP00026682001	Q1S585 Hypothetical protein related cluster	2.3
VVTU2923_at	GSVIVP00016325001	Q8GTD8 Hypothetical protein 275 related cluster	2.3
VVTU16417_at	GSVIVP00026781001	Q1SFF0 Heavy metal transport detoxification protein related cluster	2.3
VVTU7521_at	TC58147	Q8HQ06 cytochrome b related cluster	2.3
VVTU14447_at	VVTU14447_at	Q1SAF9 Mov34 MPN PAD-1 related cluster	2.3
VVTU21630_at	GSVIVP00003718001	O64865 Hypothetical protein At2g44300 related cluster	2.3
VVTU27653_at	CF372116	Q1SY04 Disease Resistance protein; AAA ATPase related cluster	2.3
VVTU34564_s_at	GSVIVP00008671001	AF359521 Vitis vinifera inward rectifying shaker-like K+ channel (SIRK) mRNA, complete cds.	2.3
VVTU36870_at	TC59559	Q1SD84 Integrase, catalytic region related cluster	2.3
VVTU31055_at	GSVIVP00030451001	Q1SEK6 Hypothetical protein related cluster	2.3
VVTU32105_x_at	CB349114	Q0KIN0 Integrase core domain containinG protein related cluster	2.3
VVTU12846_at	GSVIVP00027854001	Q9SUJ6 SGP1 monomeric G-protein related cluster	2.3

Probe set	Unique Gene ID	Annotation	Fold-change
VVTU26232_at	CF606237	UPI00005BCE7E Cluster related to UPI00005BCE7E; PREDICTED: similar to elongation factor 1 homolog (ELF1, S. cerevisiae) isoform 1	2.3
VVTU23944_at	DT030533	Q5XWL3 Gag-pol Polyprotein-like related cluster	2.3
VVTU25877_at	GSVIVP00014691001	Q0E358 Os02g0193000 protein related cluster	2.3
VVTU35202_at	VVTU35202_at	Q5Y9B9 MADS9 protein related cluster	2.3
VVTU13363_at	GSVIVP00030282001	AJ865335 Vitis vinifera mRNA for leucoAnthocyanidin reductase 1 (lar1 gene, lar1-2 allele).	2.3
VVTU37929_s_at	TC64715	Q6L3Q0 Polyprotein, putative related cluster	2.3
VVTU31655_x_at	VVTU31655_x_at	Q9M4H3 Putative Metallothionein-like protein related cluster	2.3
VVTU26725_x_at	CB980619	Q506K1 Putative aquaPorin related cluster	2.3
VVTU35901_at	TC64587	Q1SD84 Integrase, catalytic region related cluster	2.3
VVTU1601_at	GSVIVP00034381001	Q65CJ7 Hydroxyphenylpyruvate reductase related cluster	2.3
VVTU37177_at	GSVIVP00027346001	Q7XIL3 Putative 5-alpha-taxadienol-10-beta-hydroxylase related cluster	2.3
VVTU6049_at	TC56969	Q9LIE5 Far-red impaired response protein; Mutator-like transposase-like protein; Phytochrome A signaling protein-like related cluster	2.3
VVTU35042_at	GSVIVP00003362001	Q39224 SRG1 protein related cluster	2.3
VVTU39357_at	GSVIVP00013917001	Q2LAK3 Cytochrome P450 monooxygenase CYP89H3 related cluster	2.3
VVTU6970_at	TC57624	Q1SZ16 Protein kinase related cluster	2.3
VVTU22207_at	GSVIVP00037958001	Q1SMR9 Pathogenesis-related transcriptional factor and ERF related cluster	2.3
VVTU13683_at	TC54025	Q5MA57 Orf297a protein related cluster	2.3
VVTU37369_at	TC68909	P29149 RNA1 Polyprotein (P1) [Contains: P1A protein (1A) (protease cofactor); Putative ATP-dependent helicase (EC 3.6.1.-) (NTP-binding protein) (NTB) (1B) (Membrane-binding protein); Viral genome-linked protein (1C-VPg); Picornain 3C-like protease (EC 3.4.22.-) (3C-like protease) (1D-PRO); RNA-directed RNA polymerase (EC 2.7.7.48) (1E-POL)] related cluster	2.3
VVTU7537_at	GSVIVP00031600001	Q9SHF2 T19E23.8 related cluster	2.3
VVTU6673_at	GSVIVP00017993001	Q0J0H9 Os09g0508300 protein related cluster	2.3
VVTU38683_at	TC54185	Q7RWU1 Predicted protein related cluster	2.3
VVTU3478_at	GSVIVP00022112001	Q6K9T1 Oligopeptidase A-like related cluster	2.3
VVTU34108_at	CA816863	Q7XU53 OSJNBa0006A01.18 protein related cluster	2.3
VVTU16445_at	GSVIVP00017750001	Q2L360 Putative CC-NBS-LRR Resistance protein related cluster	2.3
VVTU25414_at	GSVIVP00031796001	Q9LNK3 F12K21.25 related cluster	2.3
VVTU11368_at	GSVIVP00020395001	UPI00004DBC98 Cluster related to UPI00004DBC98	2.3
VVTU36683_at	GSVIVP00023595001	Q1ZZV9 Na+ H+ antiporter related cluster	2.3
VVTU2698_at	GSVIVP00033375001	Q67V42 MutT domain protein-like related cluster	2.3
VVTU26977_at	GSVIVP00005754001	Q49RB3 Gip1-like protein related cluster	2.3
VVTU6540_at	GSVIVP00019382001	Q1RTW4 6,7-dimethyl-8-ribityllumazine synthase related cluster	2.3
VVTU23748_at	DT034979	Q9MFC5 Orf152 protein related cluster	2.3
VVTU14602_at	GSVIVP00005434001	Q94BU1 At1g71810 F14O23_17 related cluster	2.3
VVTU246_at	GSVIVP00038692001	O82702 Vacuolar ATP synthase subunit G 1 related cluster	2.3
VVTU22664_at	GSVIVP00038326001	Q2PAJ1 Putative Laccase related cluster	2.3
VVTU33583_at	CB341046	Q1SB30 HSF ETS, DNA-binding related cluster	2.3
VVTU20781_at	VVTU20781_at	Q9FZ44 F6I1.16 protein related cluster	2.3
VVTU27852_x_at	GSVIVP00031410001	Q8GTJ0 Xyloglucan endotransglycosylase related cluster	2.3
VVTU39157_at	GSVIVP00003763001	Q9SEL2 Gag-pol Polyprotein related cluster	2.3
VVTU21967_at	GSVIVP00020202001	Q84MB3 At1g06620 related cluster	2.3
VVTU10865_at	GSVIVP00028037001	Q05967 polygalacturonase precursor related cluster	2.3
VVTU17598_at	GSVIVP00010903001	Q9XED4 receptor-like protein kinase homolog RK20-1 related cluster	2.3
VVTU11570_at	GSVIVP00026069001	O23042 YUP8H12.11 protein related cluster	2.3
VVTU33956_x_at	GSVIVP00000642001	Q1S4H6 RNA-directed DNA polymerase(Reverse transcriptase); peptidase aspartic, catalytic related cluster	2.3
VVTU39188_at	GSVIVP00001301001	Q69TX5 Putative lectin-like receptor kinase related cluster	2.3
VVTU13715_at	GSVIVP00015392001	Q9STN5 Hypothetical protein T28D5.20 related cluster	2.3
VVTU10304_at	GSVIVP00014906001	Q9FN19 Arabidopsis thaliana genomic DNA, chromosome 5, TAC clone:K8K14 related cluster	2.3
VVTU36650_at	TC57350	Q8HD79 Orf5 protein related cluster	2.3
VVTU3359_at	GSVIVP00023194001	Q9FF51 Emb|CAB72159.1 related cluster	2.3
VVTU25370_at	GSVIVP00020308001	Q56WE5 Hypothetical protein At4g14310 related cluster	2.3
VVTU17103_at	TC67943	O81225 Extra-large G-protein related cluster	2.2
VVTU21409_at	GSVIVP00016150001	Q53J16 Pollen-specific protein SF3, putative related cluster	2.2
VVTU268_at	GSVIVP00035712001	Q2QYA4 Hypothetical protein related cluster	2.2
VVTU24672_at	GSVIVP00020976001	Q67X53 Putative beta-1,3-galactosyltransferase related cluster	2.2
VVTU26934_at	GSVIVP00027084001	Q67UZ4 Putative fiber Annexin related cluster	2.2
VVTU17102_s_at	GSVIVP00024088001	Q0GIK4 Cys-3-His Zinc finger protein related cluster	2.2
VVTU461_at	GSVIVP00035900001	Q5ZDY7 Conserved transmembrane protein-like related cluster	2.2
VVTU6572_at	GSVIVP00029166001	Q9MBC0 polygalacturonase related cluster	2.2
VVTU3420_at	GSVIVP00032192001	P41152 Heat shock factor protein HSF30 related cluster	2.2
VVTU5758_at	GSVIVP00030297001	Q9C535 Hypothetical protein F15D2.25 related cluster	2.2
VVTU24539_at	DT010783	Q9C6Q9 Hypothetical protein T18I24.12 related cluster	2.2
VVTU13088_at	GSVIVP00019566001	AY953543 Vitis vinifera sucrose responsive element bindinG protein (SREBP) mRNA, complete cds.	2.2
VVTU738_at	GSVIVP00038899001	Q6DW76 Digalactosyldiacylglycerol synthase 1, chloroplast precursor related cluster	2.2
VVTU9489_at	GSVIVP00032680001	Q3E959 Protein At5g25320 related cluster	2.2
VVTU703_s_at	GSVIVP00018175001	Q94C45 Phenylalanine ammonia-lyase 1 related cluster	2.2
VVTU28661_at	CD720151	Q0ZCC5 CCHC-type Integrase related cluster	2.2
VVTU36970_at	GSVIVP00016766001	Q6ZXB7 Plasma membrane sulphate transporter related cluster	2.2

Probe set	Unique Gene ID	Annotation	Fold-change
VVTU5058_s_at	GSVIVP00013502001	Q8W100 Hypothetical protein related cluster	2.2
VVTU4824_at	GSVIVP00032938001	Q8L8L6 Hypothetical protein related cluster	2.2
VVTU16060_at	CF511976	Q9ZT06 receptor-like protein kinase related cluster	2.2
VVTU12930_s_at	GSVIVP00033763001	Q9FSC6 cinnamoyl-CoA reductase related cluster	2.2
VVTU23049_at	GSVIVP00029126001	Q1SPZ8 Leucine-rich repeat; Leucine-rich repeat, cysteine-containing type related cluster	2.2
VVTU21329_at	GSVIVP00013897001	Q2LAK3 Cytochrome P450 monooxygenase CYP89H3 related cluster	2.2
VVTU37545_at	VVTU37545_at	Q6D9Z7 Putative membrane protein related cluster	2.2
VVTU25069_at	GSVIVP00011578001	Q0IZV3 Os09g0547200 protein related cluster	2.2
VVTU34081_at	GSVIVP00030268001	O04390 Nuclear matrix constituent protein 1 related cluster	2.2
VVTU15218_at	GSVIVP00021451001	Q59IV5 Plastidic phosphate translocator-like protein2 related cluster	2.2
VVTU39364_at	GSVIVP00037726001	P51819 Heat shock protein 83 related cluster	2.2
VVTU39324_at	TC58373	Q8RU52 Putative copia-like retrotransposon Hopscotch Polyprotein related cluster	2.2
VVTU1412_at	GSVIVP00001132001	Q651M3 Hypothetical protein OSJNBa0047P18.32-1 related cluster	2.2
VVTU13941_at	GSVIVP00002118001	Q1SSK6 Heat shock protein HSP20 related cluster	2.2
VVTU38757_at	TC54571	Q2HUL7 Integrase, catalytic region; Zinc finger, CCHC-type related cluster	2.2
VVTU11544_at	GSVIVP00035198001	Q9SUP0 Hypothetical protein F9F13.40 related cluster	2.2
VVTU23099_at	GSVIVP00035452001	Q19PN7 NBS type Disease Resistance protein related cluster	2.2
VVTU26302_at	CF605782	P91374 60S ribosomal protein L15 related cluster	2.2
VVTU401_at	GSVIVP00025768001	Q9LSI2 Gb|AAF27147.1 related cluster	2.2
VVTU10958_at	VVTU10958_at	Q4ID60 Hypothetical protein related cluster	2.2
VVTU33869_s_at	GSVIVP00002205001	Q8W5R4 Phosphoenolpyruvate carboxylase kinase related cluster	2.2
VVTU14624_at	GSVIVP00010566001	Q9FLM8 Similarity to Metallothionein-I gene transcription activator related cluster	2.2
VVTU40797_x_at	GSVIVP00012302001	Q84JX3 Putative Xyloglucan endotransglycosylase related cluster	2.2
VVTU38365_at	GSVIVP00012626001	Q0WTZ4 Hypothetical protein At1g04945 related cluster	2.2
VVTU22782_at	VVTU22782_at	O42952 40S ribosomal protein S30 related cluster	2.2
VVTU25630_at	CN545281	Q7XXA4 OSJNBa0019G23.12 protein related cluster	2.2
VVTU16558_at	GSVIVP00011745001	Q9SIJ0 Putative C2H2-type Zinc finger protein (Zinc finger (C2H2 type) family protein) related cluster	2.2
VVTU31964_at	CB350033	P62577 Chloramphenicol acetyltransferase related cluster	2.2
VVTU31285_at	CB343185	Q9C933 Hypothetical protein F14G24.14 related cluster	2.2
VVTU2222_at	GSVIVP00026298001	O64640 Expressed protein related cluster	2.2
VVTU2761_at	GSVIVP00014226001	Q5V9L2 cytochrome b5 isoform Cb5-C related cluster	2.2
VVTU38_at	GSVIVP00038218001	Q1KUM5 Hypothetical protein related cluster	2.2
VVTU6357_at	GSVIVP00030043001	Q2QQ91 Ureide permease 2, putative, expressed related cluster	2.2
VVTU8380_at	GSVIVP00022604001	Q9SXV1 KN1-type Homeobox protein related cluster	2.2
VVTU36018_at	GSVIVP00018381001	Q93WW5 Putative Subtilisin related cluster	2.2
VVTU38117_s_at	TC64930	Q8LMV3 Putative Reverse transcriptase related cluster	2.2
VVTU181_at	GSVIVP00017888001	Q1SIZ0 Zinc finger, NF-X1-type; Single-stranded Nucleic acid binding R3H; Zinc finger, RING-type; Zinc finger, PHD-type related cluster	2.2
VVTU15727_at	GSVIVP00025598001	Q2PYZ2 3 -5 -exoribonuclease RNA binding protein-like protein related cluster	2.2
VVTU28014_at	GSVIVP00024340001	Q9SNC4 Hypothetical protein F12A12.60 related cluster	2.2
VVTU24365_at	GSVIVP00028707001	Q19PN7 NBS type Disease Resistance protein related cluster	2.2
VVTU27471_at	GSVIVP00020090001	Q53LY5 Similar to periplasmic serine proteinase related cluster	2.2
VVTU9719_at	GSVIVP00020730001	Q9LK89 Arabidopsis thaliana genomic DNA, chromosome 3, TAC clone: K16N12 related cluster	2.2
VVTU28225_at	CF074703	Q6X5R6 lipoxygenase related cluster	2.2
VVTU21014_at	GSVIVP00022423001	Q0EDB1 Myb-related transcription factor VvMybA22-cs related cluster	2.2
VVTU502_at	VVTU502_at	Q9C7Y8 Hypothetical protein T2J15.13 related cluster	2.2
VVTU10167_at	VVTU10167_at	Q2HVT9 Thioredoxin-related related cluster	2.2
VVTU25700_at	GSVIVP00018692001	UPI00000AB55C Cluster related to UPI00000AB55C; unnamed protein product	2.2
VVTU35979_at	GSVIVP00016087001	Q1T4R9 IMP dehydrogenase GMP reductase related cluster	2.2
VVTU10756_at	VVTU10756_at	O64693 Hypothetical protein At2g34540 related cluster	2.2
VVTU2346_at	GSVIVP00000517001	Q1SFJ9 Leucine zipper, Homeobox-associated; homeodomain-related related cluster	2.2
VVTU30394_at	GSVIVP00026572001	Q1S5W6 Hypothetical protein related cluster	2.2
VVTU15473_at	GSVIVP00014921001	Q7FPQ4 Cytochrome P450 related cluster	2.2
VVTU13152_at	GSVIVP00015817001	Q9FFT6 translation Initiation factor-like protein related cluster	2.2
VVTU19306_at	GSVIVP00009549001	Q8VYI9 AT5g64080 MHJ24_6 related cluster	2.2
VVTU7500_at	GSVIVP00032351001	Q1SM50 Deoxyribodipyrimidine photolyase, class 1 related cluster	2.2
VVTU37836_at	TC52733	Q1SS89 Integrase, catalytic region related cluster	2.2
VVTU38988_at	TC62767	Q2QZZ1 Expressed protein related cluster	2.2
VVTU37843_at	GSVIVP00001441001	Q9C6C7 Hypothetical protein F10F5.13 related cluster	2.2
VVTU9491_at	GSVIVP00018104001	Q8LRL7 Nam-like protein 8 related cluster	2.2
VVTU2189_at	GSVIVP00014741001	Q76KU9 DNA binding with one finger 5 protein related cluster	2.2
VVTU20348_at	VVTU20348_at	O49506 glycolateoxidase - like protein related cluster	2.2
VVTU16609_at	GSVIVP00020893001	Q9MAR8 Putative serine carboxypeptidases related cluster	2.2
VVTU8384_at	GSVIVP00025008001	Q8LF94 Avr9 Cf-9 rapidly elicited protein 231 related cluster	2.2
VVTU24037_at	DT027564	Q4MJW2 Hypothetical protein related cluster	2.1
VVTU24090_at	GSVIVP00033262001	Q1RWV0 Ankyrin related cluster	2.1
VVTU21498_at	GSVIVP00016086001	Q9ZUC4 F5O8.26 protein related cluster	2.1
VVTU37459_at	GSVIVP00027076001	Q1SS92 E-class P450, group I related cluster	2.1
VVTU7346_at	GSVIVP00010918001	Q1T1W2 VQ related cluster	2.1
VVTU37148_at	GSVIVP00011769001	Q50J79 NAM-like protein related cluster	2.1
VVTU267_at	GSVIVP00013488001	Q1SJD7 Hypothetical protein related cluster	2.1
VVTU35361_at	GSVIVP00027111001	Q2V3W9 Protein At3g11930 related cluster	2.1
VVTU10574_at	GSVIVP00016841001	Q1RVL0 Agenet related cluster	2.1

Probe set	Unique Gene ID	Annotation	Fold-change
VVTU34019_at	GSVIVP00004640001	Q1T3P8 alcohol dehydrogenase superfamily, zinc-containing; D-isomer specific 2-hydroxyacid dehydrogenase, NAD-binding related cluster	2.1
VVTU38208_s_at	TC52841	Q6Z3S5 Hypothetical protein OSJNBa0025J22.29 related cluster	2.1
VVTU8297_at	DV221737	Q9FNE1 receptor-like serine threonine kinase related cluster	2.1
VVTU10613_at	GSVIVP00026922001	Q6YY41 Putative UDP-Glucosyltransferase related cluster	2.1
VVTU27441_at	GSVIVP00035040001	Q1T0A3 Calcium-binding EF-hand related cluster	2.1
VVTU22379_at	GSVIVP00010479001	Q7XA40 Putative Disease Resistance protein RGA3 related cluster	2.1
VVTU4756_at	GSVIVP00016152001	Q8W437 PBng143 related cluster	2.1
VVTU15250_at	GSVIVP00016421001	Q1RTN6 Quinoprotein amine dehydrogenase, beta chain-like related cluster	2.1
VVTU2688_s_at	GSVIVP00012737001	Q66NX3 Polyphenol oxidase related cluster	2.1
VVTU469_at	VVTU469_at	Q6ID09 At4g02250 related cluster	2.1
VVTU2403_at	GSVIVP00012657001	Q9ZT07 receptor-like protein kinase related cluster	2.1
VVTU31502_x_at	CB341767	Q8GV53 Photosystem II 10 kDa protein related cluster	2.1
VVTU1268_at	GSVIVP00025270001	Q9ASW4 At3g21211 related cluster	2.1
VVTU32044_at	CB349544	Q6NLD7 At1g64640 related cluster	2.1
VVTU36224_at	DT033507	Q84TB4 Putative Reverse transcriptase related cluster	2.1
VVTU12918_s_at	GSVIVP00023775001	Q5F2L4 Putative receptor associated protein related cluster	2.1
VVTU23072_at	GSVIVP00029949001	Q1T435 Leucine-rich repeat; Leucine-rich repeat, cysteine-containing type related cluster	2.1
VVTU31546_at	CB341410	Q2I314 Ribulose-1,5-bisphophate carboxylase oxygenase small subunit related cluster	2.1
VVTU33709_at	CB339508	Q0ZIZ0 Photosystem II phosphoprotein related cluster	2.1
VVTU13199_at	GSVIVP00015964001	Q8H5F8 Hypothetical protein OJ1165_F02.106 related cluster	2.1
VVTU10180_at	VVTU10180_at	Q0UUY6 Hypothetical protein related cluster	2.1
VVTU33115_at	GSVIVP00024030001	Q9LMJ9 F10K1.23 related cluster	2.1
VVTU26447_at	TC56382	Q39436 SIEP1L protein precursor related cluster	2.1
VVTU26176_at	CF606567	Q4B930 Hypothetical protein related cluster	2.1
VVTU7790_at	GSVIVP00002183001	Q84WB6 Hypothetical protein At1g54650 related cluster	2.1
VVTU27378_at	GSVIVP00015442001	Q0WN40 Hypothetical protein At4g38170 related cluster	2.1
VVTU32004_at	CB349789	P62577 Chloramphenicol acetyltransferase related cluster	2.1
VVTU18379_at	VVTU18379_at	O82528 60S ribosomal protein L15 related cluster	2.1
VVTU39811_s_at	GSVIVP00028317001	Q1SSA7 ZIM related cluster	2.1
VVTU4640_at	CF202056	Q6TPK3 Cystatin related cluster	2.1
VVTU15110_at	GSVIVP00001621001	Q1S1F5 Sulfotransferase related cluster	2.1
VVTU6132_at	GSVIVP00013919001	Q6E439 ACT11D09.3 related cluster	2.1
VVTU11902_at	GSVIVP00030060001	Q96558 UDP-glucose 6-dehydrogenase related cluster	2.1
VVTU21847_at	GSVIVP00019120001	Q9M219 Hypothetical protein T8B10_60 related cluster	2.1
VVTU27023_at	CF511310	Q9LEL9 Matrix metalloproteinase related cluster	2.1
VVTU14991_at	GSVIVP00026981001	Q6ZA15 Putative phospholipaserelatedprotein related cluster	2.1
VVTU33153_x_at	CA812309	Q9M4H3 Putative Metallothionein-like protein related cluster	2.1
VVTU8974_at	GSVIVP00036840001	Q6IV45 ferulate5-hydroxylase related cluster	2.1
VVTU25171_at	CX017888	Q49IG6 19 kDa protein related cluster	2.1
VVTU4441_at	GSVIVP00020175001	Q8LAV4 Putative DNA-bindinG protein related cluster	2.1
VVTU31378_at	CB342733	P62577 Chloramphenicol acetyltransferase related cluster	2.1
VVTU27194_at	CF405983	Q6KBB0 Putative inorganic pyrophosphatase related cluster	2.1
VVTU114_at	VVTU114_at	UPI0000D56419 Cluster related to UPI0000D56419; PREDICTED: similar to CG31884-PA, isoform A	2.1
VVTU35918_at	GSVIVP00025473001	Q1RTR5 Hypothetical protein related cluster	2.1
VVTU32629_at	CB349128	O82149 Low-molecular-weight Heat shock protein related cluster	2.1
VVTU11165_at	GSVIVP00033106001	Q1S1I4 Hypothetical protein related cluster	2.1
VVTU4200_at	GSVIVP00010579001	O81008 Putative amino acid acetyltransferase related cluster	2.1
VVTU13444_at	TC54607	Q6F2D6 Putative Polyprotein, identical related cluster	2.1
VVTU27675_at	CF371758	Q69P58 Hypothetical protein OJ1740_D06.21 related cluster	2.1
VVTU37561_at	TC64748	Q4K6E8 Polysaccharide biosynthesis protein related cluster	2.1
VVTU22372_at	GSVIVP00000653001	Q9M1K2 Putative phosphatidylinositol-4-phosphate 5-kinase 4 (EC 2.7.1.68) (AtPIP5K4) (1-phosphatidylinositol-4-phosphate kinase 4) (PtdIns(4)P- 5-kinase 4) related cluster	2.1
VVTU31605_at	CB345852	Q0WRN6 PolyUbiquitin 4 UBQ4 related cluster	2.1
VVTU5309_at	VVTU5309_at	Q9FX73 F19K19.12 protein related cluster	2.1
VVTU9820_at	GSVIVP00011830001	Q6WNU4 Subtilisin-like protease related cluster	2.1
VVTU16654_at	TC63371	O23193 Hypothetical protein C7A10.450 related cluster	2.1
VVTU25217_at	CX017161	Q7PSB4 ENSANGP00000011006 related cluster	2.1
VVTU38141_s_at	GSVIVP00022829001	Q8W0Y7 Enod8.3 related cluster	2.1
VVTU3880_at	GSVIVP00018573001	Q0IYU2 Os10g0162100 protein related cluster	2.1
VVTU932_s_at	GSVIVP00014676001	Q9M9N8 NAM-like protein related cluster	2.1
VVTU13490_at	TC58565	Q8W452 Reverse transcriptase related cluster	2.1
VVTU20639_at	GSVIVP00029636001	O64555 YUP8H12R.46 protein related cluster	2.1
VVTU25693_at	GSVIVP00018176001	Q7XA06 Synaptotagmin C related cluster	2.1
VVTU22331_at	GSVIVP00031598001	Q4ADU9 peroxidase related cluster	2.1
VVTU25725_at	GSVIVP00020075001	Q1SF23 Cation transporting ATPase, C-terminal related cluster	2.1
VVTU3678_s_at	GSVIVP00019334001	Q8GXJ5 Hypothetical protein related cluster	2.1
VVTU16578_at	TC58555	Q19KB4 PSI P700 apoprotein A2 related cluster	2.1
VVTU26500_at	CF603859	Q0TZP4 Hypothetical protein related cluster	2.1
VVTU2530_at	GSVIVP00031791001	Q9C8N5 Zinc finger protein, putative; 58191-56692 related cluster	2.1
VVTU33496_at	CA809277	Q6EPY8 SOUL heme-binding protein-like related cluster	2.1
VVTU10979_at	GSVIVP00025565001	P93005 Hypothetical protein At2g33680 related cluster	2.1
VVTU21186_at	GSVIVP00001462001	Q9LJN4 Beta-1,4-xylosidase related cluster	2.1
VVTU9640_at	GSVIVP00023723001	Q9FXV9 Copalyldiphosphate synthase No1 related cluster	2.1
VVTU14013_at	GSVIVP00036257001	Q681N2 Arm repeat containinG protein related cluster	2.1

Probe set	Unique Gene ID	Annotation	Fold-change
VVTU26608_at	CF518421	Q7XU53 OSJNBa0006A01.18 protein related cluster	2.1
VVTU29362_at	GSVIVP00037785001	Q1S8T9 Protein kinase related cluster	2.1
VVTU20580_at	VVTU20580_at	Q1T5K4 RNA-directed DNA polymeraserelated cluster	2.1
VVTU37150_at	GSVIVP00032713001	Q9LEU1 Hypothetical protein T30N20_270 related cluster	2.1
VVTU5825_at	GSVIVP00002884001	Q6ZH46 Hypothetical protein OJ1217_F02.5 related cluster	2.1
VVTU21176_at	GSVIVP00015010001	Q7XAU6 Class IV Chitinase related cluster	2.1
VVTU22805_at	VVTU22805_at	Q2HVW3 Hypothetical protein related cluster	2.1
VVTU37801_at	TC51838	Q2RAL0 Retrotransposon protein, putative, unclassified related cluster	2.1
VVTU36714_at	TC57105	Q6T6V4 Hypothetical protein related cluster	2.1
VVTU6031_at	GSVIVP00002673001	Q8GSZ9 Armadillo repeat-containing protein-like related cluster	2.1
VVTU31338_at	CB343065	Q8RWP3 GTP-bindinG protein LepA homolog related cluster	2.1
VVTU38840_at	TC55894	Q1SAZ0 Integrase, catalytic region related cluster	2.1
VVTU20488_at	GSVIVP00020875001	Q69JV9 Zinc finger (C3HC4-type RING finger)-like protein related cluster	2.1
VVTU10640_at	GSVIVP00034529001	Q9LRP9 Gb|AAF35421.1 related cluster	2.1
VVTU415_at	GSVIVP00020596001	Q8H998 Phosphoprotein NtEPb2 related cluster	2.1
VVTU20698_at	VVTU20698_at	Q19PL5 NBS-LRR type Disease Resistance protein related cluster	2.0
VVTU5331_at	GSVIVP00006978001	Q0ISK1 Os11g0506800 protein related cluster	2.0
VVTU11829_at	TC64824	Q9LSM3 Arabidopsis thaliana genomic DNA, chromosome 5, TAC clone:K21L13 related cluster	2.0
VVTU7988_s_at	GSVIVP00022483001	Q2HUZ1 Hypothetical protein related cluster	2.0
VVTU20570_x_at	GSVIVP00036130001	Q1T416 SKP1 component related cluster	2.0
VVTU1022_at	GSVIVP00010584001	Q1SKP9 At5g67620 related cluster	2.0
VVTU6982_at	GSVIVP00031502001	Q40285 Anthocyanidin 3-O-Glucosyltransferase related cluster	2.0
VVTU8600_at	GSVIVP00034949001	Q7PC84 Probable pleiotropic drug Resistance protein 11 related cluster	2.0
VVTU16929_at	CB343368	O81790 NAM CUC2 -like protein related cluster	2.0
VVTU22337_at	GSVIVP00005992001	Q1RYA5 auxin efflux Carrier related cluster	2.0
VVTU35574_at	GSVIVP00011267001	P52835 Flavonol 3-sulfotransferase related cluster	2.0
VVTU37738_s_at	TC60941	Q04357 Potato DNA for copia-like transposable element related cluster	2.0
VVTU3303_s_at	GSVIVP00035835001	Q3SAJ9 WRKY-A1244 related cluster	2.0
VVTU27319_at	CF405189	Q9SWB5 seed maturation protein PM37 related cluster	2.0
VVTU25975_at	GSVIVP00017511001	Q2HSH2 Hypothetical protein related cluster	2.0
VVTU10291_at	VVTU10291_at	Q9FI38 Cytochrome P450-like protein related cluster	2.0
VVTU6987_at	GSVIVP00019144001	O24625 Anther-specific protein related cluster	2.0
VVTU9655_at	TC61182	Q0WYX3 lipid transfer protein related cluster	2.0
VVTU2415_at	GSVIVP00025011001	Q8L3R2 calmodulin-like protein 41 related cluster	2.0
VVTU27847_at	GSVIVP00018880001	Q9FI59 Gb|AAD29063.1 related cluster	2.0
VVTU4724_at	GSVIVP00024445001	Q1SQN1 peptidase S26A, signal peptidase I; HAD-superfamily hydrolase, subfamily IIA related cluster	2.0
VVTU22191_at	VVTU22191_at	Q9LSV7 Gb|AAD21483.1 related cluster	2.0
VVTU38385_at	GSVIVP00038198001	Q9ZW93 F5A8.5 protein related cluster	2.0
VVTU21889_at	GSVIVP00010516001	Q9M636 Hypothetical protein related cluster	2.0
VVTU21891_s_at	DV218500	Q6IM91 DVL10 related cluster	2.0
VVTU31661_at	CB345548	Q9M4H3 Putative Metallothionein-like protein related cluster	2.0
VVTU3405_at	GSVIVP00024728001	Q8RU85 At2g31940 F20M17.2 related cluster	2.0
VVTU31323_at	CB343105	Q677H3 Chloroplast chlorophyll A-B bindinG protein related cluster	2.0
VVTU31091_at	CB837500	O82276 Putative non-LTR retroelement Reverse transcriptase related cluster	2.0
VVTU20782_at	GSVIVP00022569001	Q5VMK0 Synbindin-like related cluster	2.0
VVTU5756_at	GSVIVP00014365001	Q945Q4 At2g16800 T24I21.21 related cluster	2.0
VVTU16057_at	GSVIVP00014710001	Q2LAJ7 Cytochrome P450 monooxygenase CYP74A3 related cluster	2.0
VVTU14585_at	CB981848	Q9LMX5 F21F23.17 protein related cluster	2.0
VVTU27776_at	GSVIVP00023181001	Q7Y250 arabinogalactan protein related cluster	2.0
VVTU6543_at	GSVIVP00023980001	Q1RZ42 Transferase related cluster	2.0
VVTU31585_at	CB341248	UPI00001A38B9 Cluster related to UPI00001A38B9; Putative Pop3 protein	2.0
VVTU581_at	GSVIVP00025015001	Q9XIM7 Similar to cold acclimation protein WCOR413 related cluster	2.0
VVTU10283_at	GSVIVP00022657001	Q1RV39 Calponin-like Actin-binding; Kinesin, motor region related cluster	2.0
VVTU5808_at	GSVIVP00036982001	Q8VYI1 At1g69640 F24J1.22 related cluster	2.0
VVTU10888_at	GSVIVP00014235001	Q1SW57 HEC Ndc80p related cluster	2.0
VVTU27333_at	CF405094	Q1SJK7 WD40-like related cluster	2.0
VVTU37786_at	GSVIVP00026201001	O81790 NAM CUC2 -like protein related cluster	2.0
VVTU30728_at	CB919079	Q9SA17 F28K20.17 protein related cluster	2.0
VVTU4736_at	TC69701	Q3ED30 Protein At1g33060 related cluster	2.0
VVTU2474_at	TC61710	Q1SSW0 Protein phosphatase 2C related cluster	2.0
VVTU10239_at	GSVIVP00020922001	Q1SSI2 Tyrosine protein kinase, active site related cluster	2.0
VVTU1229_at	GSVIVP00032188001	O82645 Hypothetical protein AT4g33050 related cluster	2.0
VVTU15417_at	GSVIVP00027372001	Q69Y38 Hypothetical protein P0021H10.8-1 related cluster	2.0
VVTU15919_at	GSVIVP00008074001	Q8RXR4 Hypothetical protein At4g14750 related cluster	2.0
VVTU1811_at	GSVIVP00015965001	Q8S343 Hypothetical protein upa10 related cluster	2.0
VVTU33225_at	CA811504	Q29II4 GA18962-PA related cluster	2.0
VVTU38381_at	GSVIVP00027898001	Q49N12 Putative receptor-like protein kinase 2 related cluster	2.0
VVTU16626_at	GSVIVP00004727001	Q9FWE6 Mucin-like protein related cluster	2.0
VVTU34012_at	GSVIVP00017653001	Q75W19 Cytochrome P450 related cluster	2.0
VVTU8211_s_at	GSVIVP00030292001	Q4G3H5 RAV transcription factor related cluster	2.0
VVTU9224_at	GSVIVP00030679001	Q9AR41 Glutamate decarboxylase related cluster	2.0
VVTU2288_at	TC58973	Q6NMS1 At2g07725 related cluster	2.0
VVTU9637_at	GSVIVP00025519001	Q94B15 polygalacturonase PG1 related cluster	2.0
VVTU20738_at	GSVIVP00016116001	Q6YWR1 Hypothetical protein OSJNBa0072H09.27 related cluster	2.0

Probe set	Unique Gene ID	Annotation	Fold-change
VVTU10868_at	GSVIVP00011849001	Q1SL70 Leucine-rich repeat; Leucine-rich repeat, cysteine-containing type related cluster	2.0
VVTU5230_at	GSVIVP00025836001	Q9SKT9 Putative sugar transporter related cluster	2.0
VVTU21840_at	GSVIVP00036425001	Q8LB59 Minor allergen related cluster	2.0
VVTU12992_at	TC60155	Q1S9K1 Integrase, catalytic region; Zinc finger, CCHC-type; peptidase aspartic, catalytic related cluster	2.0
VVTU24449_at	GSVIVP00037687001	Q9FHB7 Similarity to unknown protein related cluster	2.0
VVTU302_at	GSVIVP00020664001	Q9CAB3 Putative ABC transporter; 82503-78258 related cluster	2.0
VVTU473_at	GSVIVP00023098001	Q9LFG8 Probable White-brown complex homoloG protein 20 related cluster	2.0
VVTU28354_at	CD801434	Q6YSL4 cytochrome c biogenesis ccmC related cluster	2.0
VVTU21207_at	GSVIVP00025271001	P93194-2 Isoform INRPK1a of P93194 related cluster	2.0
VVTU6520_at	GSVIVP00015404001	Q1SJZ1 peptidase M20 related cluster	2.0
VVTU6092_at	TC65987	Q8L8G0 Nam-like protein 1 related cluster	2.0
VVTU32153_at	CB348744	Q05539 Acidic 26 kDa endoChitinase precursor related cluster	2.0
VVTU25474_at	GSVIVP00017604001	Q1S4Z2 Protein tyrosine kinase, putative related cluster	2.0
VVTU22071_at	GSVIVP00014719001	Q1SJP3 E-class P450, group I related cluster	2.0
VVTU7299_at	GSVIVP00038266001	Q9C9N8 Hypothetical protein F4N21_3 related cluster	2.0
VVTU38040_at	TC65185	Q2QUR7 Retrotransposon protein, putative, LINE subclass related cluster	2.0
VVTU8024_at	GSVIVP00010960001	Q67V42 MutT domain protein-like related cluster	2.0
VVTU21236_at	GSVIVP00026285001	Q9M4G8 Putative Ripening-related P-450 enzyme related cluster	2.0
VVTU27133_at	CF414198	Q43321 enolase related cluster	2.0
VVTU40019_at	TC53045	UPI0000D56130 Cluster related to UPI0000D56130; PREDICTED: similar to CG8983-PA, isoform A	2.0
VVTU13691_at	TC56088	Q338D5 Endonuclease exonuclease phosphatase family protein, putative, expressed related cluster	2.0
VVTU29520_at	GSVIVP00002358001	Q2AA51 Retrotransposon gaG protein related cluster	2.0
VVTU3038_at	GSVIVP00032812001	Q84NG9 2S albumin related cluster	2.0
VVTU31598_at	CB345886	Q8GV53 Photosystem II 10 kDa protein related cluster	2.0
VVTU6353_at	GSVIVP00024605001	Q9SYD7 F11M15.21 protein related cluster	2.0
VVTU33594_at	CB340926	UPI0000DBFC45 Cluster related to UPI0000DBFC45	2.0
VVTU31790_x_at	CB344837	Q0JIM3 Os01g0790900 protein related cluster	2.0
VVTU27643_at	CF372224	Q1S2L8 Transcriptional factor B3; Cupredoxin; TonB box, N-terminal related cluster	2.0
VVTU31834_at	CB344568	P29463 Triose phosphate phosphate translocator, chloroplast precursor related cluster	2.0
VVTU10715_at	GSVIVP00010644001	Q9T071 Probable hexokinase-like protein related cluster	2.0
VVTU21595_at	GSVIVP00000180001	Q2XPW6 NAD-dependent epimerase dehydratase family protein-like protein related cluster	2.0
VVTU38715_at	TC53951	Q5GA65 Hypothetical protein related cluster	2.0
VVTU33315_s_at	GSVIVP00037681001	Q6RVV4 Short-chain dehydrogenase Tic32 related cluster	2.0
VVTU26004_x_at	GSVIVP00033062001	Q4F883 SAG101 related cluster	2.0
VVTU38248_at	GSVIVP00000383001	Q7XMK5 OSJNBb0039L24.13 protein related cluster	2.0
VVTU35586_at	GSVIVP00036899001	Q1T5Q9 CAMP response element binding (CREB) protein related cluster	2.0
VVTU3692_at	GSVIVP00035725001	Q652J4 Probable Potassium transporter 13 related cluster	2.0
VVTU24878_at	DT006469	Q0E285 Os02g0261600 protein related cluster	2.0
VVTU40098_at	TC55235	Q0ZIW0 Hypothetical chloroplast RF1 related cluster	2.0
VVTU12652_at	TC56428	O23285 Hypothetical protein related cluster	2.0
VVTU15413_at	GSVIVP00025381001	O48522 Hypothetical protein At2g42140 related cluster	2.0
VVTU25858_x_at	GSVIVP00014102001	P52596 glutamate dehydrogenase related cluster	2.0
VVTU16207_at	TC55666	Q9LTA6 Retroelement pol Polyprotein-like related cluster	2.0
VVTU38978_at	GSVIVP00028636001	Q5K3V8 Monosaccharide transporter related cluster	2.0
VVTU26364_at	CF605348	Q25129 ADT ATP translocase related cluster	2.0
VVTU10867_at	GSVIVP00002102001	Q49QW7 Myb family transcription factor-related protein related cluster	2.0
VVTU9074_at	GSVIVP00032335001	Q1SYX6 Protein kinase related cluster	2.0
VVTU7255_at	GSVIVP00000056001	Q8RX55 At1g74800 F25A4_38 related cluster	2.0
VVTU33120_at	CA812635	Q6L974 GAG-POL related cluster	2.0
VVTU22166_at	GSVIVP00019092001	Q9SST7 ethylene-responsive element binding protein1 homolog related cluster	2.0
VVTU23047_at	GSVIVP00019860001	Q8VZI5 AT5g18840 F17K4_90 related cluster	2.0
VVTU23429_at	GSVIVP00020896001	Q5VQ69 Putative GTP-binding membrane protein LepA related cluster	2.0
VVTU27019_at	GSVIVP00032488001	Q1SZC3 DNA-directed DNA polymerase; helicase, C-terminal related cluster	1.9
VVTU33744_s_at	CB339208	P04280 Basic salivary proline-rich protein 1 precursor (Salivary proline-rich protein) [Contains: Basic peptide IB-6; Peptide P-H] related cluster	1.9
VVTU35525_at	VVTU35525_at	Q96321 Importin alpha-1 subunit related cluster	1.9
VVTU10005_at	GSVIVP00024602001	Q9LIH4 Arabidopsis thaliana genomic DNA, chromosome 3, P1 clone:MFD22 related cluster	1.9
VVTU22920_at	GSVIVP00024884001	Q9FYZ9 Benzoate carboxyl methyltransferase related cluster	1.9
VVTU4597_at	GSVIVP00028703001	Q9LIP9 Glutamine-fructose-6-phosphate transaminase 2 related cluster	1.9
VVTU3583_at	GSVIVP00011024001	Q38M75 Ripening regulated protein-like related cluster	1.9
VVTU11701_at	VVTU11701_at	Q40545 pyruvate kinase isozyme A, chloroplast precursor related cluster	1.9
VVTU31142_at	CB834382	Q3HNG0 Reverse transcriptase related cluster	1.9
VVTU842_at	GSVIVP00015239001	AY663846 Vitis vinifera hexose transporter HT2 mRNA, complete cds.	1.9
VVTU5844_at	GSVIVP00027691001	Q9SZS3 Hypothetical protein F27G19.80 related cluster	1.9
VVTU21362_at	GSVIVP00003201001	Q940P4 AT5g07010 MOJ9_18 related cluster	1.9
VVTU31342_x_at	CB343042	Q5M9R1 Hypothetical protein orf138c related cluster	1.9
VVTU20847_at	VVTU20847_at	Q65XH1 Hypothetical protein OJ1126_B11.8 related cluster	1.9
VVTU20236_at	GSVIVP00008243001	Q1SY04 Disease Resistance protein; AAA ATPase related cluster	1.9
VVTU37173_at	GSVIVP00010271001	Q8LF75 Putative receptor ser thr protein kinase related cluster	1.9
VVTU32812_at	CB345024	Q4YMP6 Hypothetical protein related cluster	1.9
VVTU37982_at	TC68241	Q6C4I6 Histone H2A related cluster	1.9

Probe set	Unique Gene ID	Annotation	Fold-change
VVTU414_at	GSVIVP00003193001	Q5VRL9 Hypothetical protein OSJNBa0019F11.17 related cluster	1.9
VVTU8346_at	GSVIVP00000626001	Q8S8F1 Expressed protein related cluster	1.9
VVTU38245_s_at	GSVIVP00026040001	Q9LSZ3 Arabidopsis thaliana genomic DNA, chromosome 3, P1 clone: MSD21 related cluster	1.9
VVTU11294_at	GSVIVP00012270001	Q9SFV5 Putative RNA-bindinG protein related cluster	1.9
VVTU17293_at	GSVIVP00025407001	Q0DTT5 Os03g0226400 protein related cluster	1.9
VVTU21264_at	GSVIVP00006717001	Q3Y6V1 cellulose synthase-like protein CslG related cluster	1.9
VVTU26966_at	GSVIVP00028731001	Q2Z1Z0 Cinnamyl alcohol dehydrogenase related cluster	1.9
VVTU31806_at	CB344768	UPI0000DB7FA7 Cluster related to UPI0000DB7FA7; PREDICTED: similar to Chromodomain helicase-DNA-bindinG protein Mi-2 homolog (ATP-dependent helicase Mi-2) (dMi-2)	1.9
VVTU9498_at	GSVIVP00002561001	Q5VRN4 Hypothetical protein OSJNBa0004I20.1 related cluster	1.9
VVTU39171_at	TC61086	Q1JTB7 Dynein heavy chain, putative related cluster	1.9
VVTU32217_at	CB348286	Q8LDK5 Hypothetical protein related cluster	1.9
VVTU10147_at	GSVIVP00024341001	Q2HTK4 Protein kinase; U box related cluster	1.9
VVTU5158_at	GSVIVP00006946001	Q93ZM9 AT5g13220 T31B5_40 related cluster	1.9
VVTU5792_at	GSVIVP00009747001	Y09590 V.vinifera mRNA for hexose transporter.	1.9
VVTU35131_at	VVTU35131_at	Q8LID8 Cyst nematode Resistance protein-like protein related cluster	1.9
VVTU5052_at	GSVIVP00038099001	Q1SHV7 GDSL-like lipaseAcylhydrolase related cluster	1.9
VVTU21923_at	VVTU21923_at	Q6Q3H2 terpenoid synthetase related cluster	1.9
VVTU23608_at	DT037853	Q6L974 GAG-POL related cluster	1.9
VVTU38079_at	TC70669	Q7XPT5 OSJNBa0083N12.5 protein related cluster	1.9
VVTU4403_at	GSVIVP00013099001	Q2L3C0 Hypothetical protein related cluster	1.9
VVTU17065_s_at	GSVIVP00021619001	Q1S6L1 Hypothetical protein related cluster	1.9
VVTU31901_at	CB344118	Q9M4Y3 30S ribosomal protein S10, chloroplast precursor related cluster	1.9
VVTU10681_at	GSVIVP00038589001	Q9ZVK1 Probable Xyloglucan endotransglucosylase hydrolase protein 10 precursor related cluster	1.9
VVTU9661_at	GSVIVP00031154001	Q3L181 Perakine reductase related cluster	1.9
VVTU21946_at	GSVIVP00009398001	Q9LUC6 Cytochrome P450 related cluster	1.9
VVTU37649_at	TC60294	Q5GA65 Hypothetical protein related cluster	1.9
VVTU36696_at	TC57736	Q1S5B9 Hypothetical protein related cluster	1.9
VVTU29116_at	CB002091	Q2QNF1 Retrotransposon protein, putative, unclassified related cluster	1.9
VVTU37514_at	GSVIVP00003816001	Q2HVC0 Protein kinase related cluster	1.9
VVTU39790_at	GSVIVP00034106001	Q94AX9 At1g72650 F28P22_16 related cluster	1.9
VVTU25431_s_at	GSVIVP00001442001	Q6ZXI0 Putative Leucine-rich repeat protein related cluster	1.9
VVTU34309_at	GSVIVP00016037001	Q0JM23 Os01g0548600 protein related cluster	1.9
VVTU1802_at	GSVIVP00036344001	Q05762 Bifunctional dihydrofolatereductase-thymidylatesynthase 1 (DHFR-TS 1) [Includes: Dihydrofolatereductase (EC 1.5.1.3); Thymidylatesynthase (EC 2.1.1.45)] related cluster	1.9
VVTU4884_at	GSVIVP00002825001	Q2YHM9 Caffeoyl-CoA O-methyltransferase related cluster	1.9
VVTU31250_at	CB343306	Q9S7V6 F17A17.13 protein related cluster	1.9
VVTU39256_at	GSVIVP00015089001	Q9STU7 Hypothetical protein T22A6.310 related cluster	1.9
VVTU15272_at	GSVIVP00011475001	Q9ZRX9 Cyclin D2.1 protein related cluster	1.9
VVTU22308_at	GSVIVP00009699001	Q9FX85 peroxidase 10 precursor related cluster	1.9
VVTU5163_at	GSVIVP00015856001	Q9FGQ9 Arabidopsis thaliana genomic DNA, chromosome 5, TAC clone:K14A3 related cluster	1.9
VVTU23294_at	GSVIVP00030031001	Q9S7I6 Hypothetical protein F1C9.8 related cluster	1.9
VVTU11210_at	GSVIVP00004605001	Q9SY81 F14N23.27 related cluster	1.9
VVTU3432_at	GSVIVP00016830001	Q69X62 Putative beta-ketoacyl-CoA synthase related cluster	1.9
VVTU105_at	GSVIVP00000651001	Q6K4N6 Amino acid transporter-like related cluster	1.9
VVTU16560_s_at	GSVIVP00005232001	Q1S8T9 Protein kinase related cluster	1.9
VVTU40080_at	TC54061	Q10M26 Retrotransposon protein, putative, Ty1-copia subclass, expressed related cluster	1.9
VVTU11170_at	VVTU11170_at	Q84JR3 Hypothetical protein At4g21705 related cluster	1.9
VVTU32199_at	CB348447	O82560 Glutamine synthetase cytosolic isozyme 2 related cluster	1.9
VVTU26361_at	CF605369	Q95033 Phosphoribulokinase precursor related cluster	1.9
VVTU23068_at	GSVIVP00024427001	Q2HU31 Glycoside hydrolase, family 18 related cluster	1.9
VVTU36312_at	TC57606	Q1SB62 Integrase, catalytic region; Zinc finger, CCHC-type; peptidase aspartic, catalytic related cluster	1.9
VVTU22482_at	GSVIVP00014233001	Q75I98 Hypothetical protein OSJNBa0004L11.12 related cluster	1.9
VVTU22808_at	GSVIVP00032782001	Q9SZ50 Hypothetical protein AT4g31830 related cluster	1.9
VVTU37627_at	TC62975	Q6URA2 TIR-NBS-LRR type R protein 7 related cluster	1.9
VVTU652_s_at	GSVIVP00019830001	AY634283 Vitis vinifera nhx1 antiporter mRNA, complete cds.	1.9
VVTU9977_at	GSVIVP00035825001	Q84QD7 Avr9 Cf-9 rapidly elicited protein 276 related cluster	1.9
VVTU16171_at	GSVIVP00015302001	Q9C8X5 Hypothetical protein F7F23.10 related cluster	1.9
VVTU36311_at	GSVIVP00038430001	Q259Z5 H0315F07.7 protein related cluster	1.9
VVTU40764_s_at	TC62469	Q7XTW0 OSJNBa0010D21.5 protein related cluster	1.9
VVTU16052_at	GSVIVP00035869001	Q8S8E3 Expressed protein related cluster	1.9
VVTU24705_at	DT008521	Q9FFU5 genomic DNA, chromosome 5, P1 clone:MBG8 related cluster	1.9
VVTU4526_at	GSVIVP00013471001	Q94K88 Hypothetical protein At4g37210 related cluster	1.9
VVTU12707_s_at	GSVIVP00017657001	Q9M9A8 F27J15.15 related cluster	1.9
VVTU31354_at	CB342949	Q9XIN6 Expressed protein related cluster	1.9
VVTU4246_at	GSVIVP00016458001	Q4ZJ73 12-oxophytodienoate reductase related cluster	1.9
VVTU8162_at	GSVIVP00020070001	Q43183 Sulfate adenylyltransferase related cluster	1.9
VVTU14689_at	TC71044	Q1S8G9 Hypothetical protein related cluster	1.9
VVTU7497_s_at	GSVIVP00000261001	Q84KB4 MRGH5 related cluster	1.9
VVTU27411_at	GSVIVP00025444001	Q9FG09 Gb|AAD30234.1 related cluster	1.9
VVTU38747_at	GSVIVP00035099001	Q7FMW1 MRP-like ABC transporter related cluster	1.9

Probe set	Unique Gene ID	Annotation	Fold-change
VVTU9801_at	GSVIVP00012495001	Q9SFU5 T1B9.19 protein related cluster	1.9
VVTU7837_at	GSVIVP00014576001	Q0ZR50 Hypothetical protein related cluster	1.9
VVTU31836_at	CB344566	O82062 39 kDa EF-Hand containinG protein related cluster	1.9
VVTU6283_at	GSVIVP00027216001	Q6F2U6 Expressed protein related cluster	1.9
VVTU40594_at	GSVIVP00023825001	O48780 3-ketoacyl-CoA synthase 11 related cluster	1.9
VVTU31310_at	CB343137	P12329 chlorophyll a-b bindinG protein 1, chloroplast precursor related cluster	1.9
VVTU36878_at	GSVIVP00024586001	Q9C8J6 Hypothetical protein F5D21.19 related cluster	1.9
VVTU6882_at	GSVIVP00019034001	Q2QYH7 Cytochrome P450 family protein, expressed related cluster	1.9
VVTU32276_at	CB347857	Q719L3 Rubber synthesis protein related cluster	1.9
VVTU36068_at	TC57945	Q22C81 RNase H family protein related cluster	1.9
VVTU8169_at	CB002246	Q1SKU9 Hypothetical protein related cluster	1.9
VVTU15740_at	GSVIVP00016199001	Q9FNR3 Leucine zipper protein-like related cluster	1.9
VVTU21781_at	VVTU21781_at	Q0V1Q1 Predicted protein related cluster	1.9
VVTU17004_at	CB348242	Q1SHW5 Pathogenesis-related transcriptional factor and ERF related cluster	1.9
VVTU7995_at	GSVIVP00019659001	Q9FVE7 Plasma membrane Ca2+-ATPase related cluster	1.9
VVTU11571_at	VVTU11571_at	Q0GPI4 BZIP transcription factor bZIP35 related cluster	1.9
VVTU37727_at	GSVIVP00019578001	Q1T642 Protein kinase related cluster	1.9
VVTU39704_at	TC63373	Q2QZX3 Retrotransposon protein, putative, Ty1-copia subclass related cluster	1.9
VVTU40435_at	GSVIVP00034543001	Q9XI23 Boron transporter-like protein 2 related cluster	1.9
VVTU20717_at	VVTU20717_at	Q9SFG2 F2O10.6 protein related cluster	1.9
VVTU23210_at	GSVIVP00025111001	Q8GUJ0 Hypothetical protein At5g16520 related cluster	1.9
VVTU8221_at	GSVIVP00020136001	Q1SHV7 GDSL-like lipaseAcylhydrolase related cluster	1.9
VVTU15741_at	CN545606	Q1SGY4 Hypothetical protein related cluster	1.9
VVTU37933_at	GSVIVP00028930001	O04568 T7N9.26 related cluster	1.9
VVTU25536_at	GSVIVP00030275001	Q8RX58 At2g34960 F19I3.19 related cluster	1.9
VVTU35356_at	GSVIVP00022287001	Q3C210 flavonoid 3 ,5 -hydroxylase related cluster	1.9
VVTU11385_s_at	VVTU11385_s_at	Q8GXY3 Hypothetical protein At3g18510 MYF24_22 related cluster	1.9
VVTU37826_at	GSVIVP00027475001	Q9LHQ8 Arabidopsis thaliana genomic DNA, chromosome 3, BAC clone: F3H11 related cluster	1.9
VVTU1775_at	GSVIVP00032128001	Q9XGZ3 T1N24.3 protein related cluster	1.9
VVTU17167_at	GSVIVP00025530001	Q9LIC3 Selenium-binding protein-like related cluster	1.9
VVTU406_at	GSVIVP00026077001	Q0WPC5 Uclacyanin I-like predicted GPI-anchored protein related cluster	1.9
VVTU13276_s_at	GSVIVP00037218001	Q0INB8 Os12g0484700 protein related cluster	1.9
VVTU34053_at	CA818367	Q2HS29 transcription factor IIA, beta-barrel related cluster	1.9
VVTU28000_at	GSVIVP00026429001	Q8LHG0 Membrane protein-like related cluster	1.9
VVTU10739_at	VVTU10739_at	Q4IJ62 Hypothetical protein related cluster	1.8
VVTU15142_at	GSVIVP00001433001	Q8LD21 RING-H2 finger protein RHY1a related cluster	1.8
VVTU27115_at	GSVIVP00026923001	Q8S342 Putative Anthocyanidine rhamnosyl-transferase related cluster	1.8
VVTU36838_at	GSVIVP00035898001	O49522 Hypothetical protein F28J12.200 related cluster	1.8
VVTU38075_at	GSVIVP00016255001	Q8VY00 Putative RNA helicase related cluster	1.8
VVTU1994_at	GSVIVP00036860001	AF159124 Vitis vinifera beta-galactosidase mRNA, partial cds.	1.8
VVTU858_at	GSVIVP00015830001	Q42581 Ribose-phosphate pyrophosphokinase 1 related cluster	1.8
VVTU6605_s_at	GSVIVP00011870001	Q52QR0 NAC domain protein NAC6 related cluster	1.8
VVTU5531_at	GSVIVP00019268001	O65168 auxin-induced protein related cluster	1.8
VVTU10434_at	GSVIVP00006868001	Q9FLQ2 Similarity to NPK1-related protein kinase related cluster	1.8
VVTU11553_at	VVTU11553_at	Q2HSI9 Hypothetical protein related cluster	1.8
VVTU13438_s_at	GSVIVP00037037001	Q0KIN0 Integrase core domain containinG protein related cluster	1.8
VVTU22382_at	GSVIVP00038029001	Q9LH16 Similarity to unknown protein related cluster	1.8
VVTU35641_x_at	GSVIVP00012190001	AF501625 Vitis vinifera glutathione-S-transferase mRNA, complete cds.	1.8
VVTU6220_at	GSVIVP00031771001	Q6NMB7 At1g43650 related cluster	1.8
VVTU39593_at	GSVIVP00009546001	Q8GRK2 Somatic Embryogenesis receptor kinase 1 related cluster	1.8
VVTU26685_at	CF517413	Q1ST43 Integrase, catalytic region; peptidase aspartic, catalytic related cluster	1.8
VVTU31302_at	CB343151	Q9M0S3 Hypothetical protein AT4g09040 related cluster	1.8
VVTU32158_at	GSVIVP00018344001	Q6WHC0 Chloroplast small Heat shock protein class I related cluster	1.8
VVTU32919_x_at	CB349065	Q5M9R1 Hypothetical protein orf138c related cluster	1.8
VVTU131_at	GSVIVP00018969001	Q5W964 PPR986-12 related cluster	1.8
VVTU14606_at	CB910409	Q5JQV3 OSJNBa0061C06.2 protein related cluster	1.8
VVTU16196_at	TC67438	Q9XEY5 Nt-iaa28 deduced protein related cluster	1.8
VVTU16261_at	GSVIVP00027470001	Q9FIV1 Gb|AAF02153.1 related cluster	1.8
VVTU35959_at	DV940985	Q1SBY9 RNA polymerase subunit, RPB5; RNA polymerase Rpb5, N-terminal related cluster	1.8
VVTU10591_at	VVTU10591_at	Q4WKT8 Endo-polygalacturonase related cluster	1.8
VVTU25679_at	CN007229	Q1RWU8 Di-trans-poly-cis-decaprenylcistransferase related cluster	1.8
VVTU31477_x_at	CB341947	Q2A9V6 Plastid ribosomal protein L19, putative related cluster	1.8
VVTU36918_at	GSVIVP00020739001	Q1RU40 Lipolytic enzyme, G-D-S-L related cluster	1.8
VVTU38097_at	GSVIVP00003060001	Q6K297 Pentatricopeptide (PPR) repeat-containing protein-like related cluster	1.8
VVTU34030_at	CB001082	Q1SD84 Integrase, catalytic region related cluster	1.8
VVTU15268_at	GSVIVP00021629001	Q9SJM8 Expressed protein related cluster	1.8
VVTU20954_at	VVTU20954_at	Q66TV8 tubulin related cluster	1.8
VVTU33672_at	CB339925	Q9LUN0 Arabidopsis thaliana genomic DNA, chromosome 3, P1 clone: MKP6 related cluster	1.8
VVTU39365_at	GSVIVP00014984001	Q9SY57 F14N23.3 related cluster	1.8
VVTU505_at	GSVIVP00036532001	Q9LHL3 RNA-binding protein-like related cluster	1.8
VVTU55_at	GSVIVP00023845001	Q9M9Y7 F4H5.11 protein related cluster	1.8
VVTU6339_at	GSVIVP00020606001	P52425 Glycerol-3-phosphate dehydrogenase [NAD+] related cluster	1.8
VVTU5132_at	GSVIVP00037017001	Q9M9S6 F14L17.13 protein related cluster	1.8

Probe set	Unique Gene ID	Annotation	Fold-change
VVTU32847_x_at	CB344492	UPI0000E23028 Cluster related to UPI0000E23028; PREDICTED: Hypothetical protein	1.8
VVTU35331_at	GSVIVP00027713001	Q6Z493 Putative MATE efflux protein family protein related cluster	1.8
VVTU34076_at	CA817592	Q9AT32 Poly(A)-bindinG protein related cluster	1.8
VVTU36888_at	GSVIVP00032642001	Q8GZM8 Putative serine hydrolase related cluster	-1.8
VVTU8563_at	GSVIVP00025360001	Q9SNV3 Squamosa promoter binding protein-homologue 5 related cluster	-1.8
VVTU35751_s_at	GSVIVP00013693001	Q53HY2 Hypothetical protein mc410 related cluster	-1.8
VVTU4644_at	GSVIVP00020251001	Q9SK61 Expressed protein related cluster	-1.8
VVTU5956_at	GSVIVP00018930001	AF265562 Vitis vinifera MAD-box transcripion factor (mads1) mRNA, complete cds.	-1.8
VVTU3553_at	TC53996	Q9SHJ6 F12K11.14 related cluster	-1.8
VVTU5715_at	GSVIVP00023446001	Q9FGV1 auxin response factor 8 related cluster	-1.8
VVTU929_at	GSVIVP00001050001	Q9BBU5 Cu Zn-superoxide dismutase copper chaperone precursor related cluster	-1.8
VVTU10351_at	GSVIVP00019349001	Q9ZR56 Ribonucleotide reductase related cluster	-1.8
VVTU24711_at	DT008480	Q9SD94 Hypothetical protein F13G24.100 related cluster	-1.8
VVTU33089_at	GSVIVP00036297001	Q9FY83 Hypothetical protein T5E8_120 related cluster	-1.8
VVTU39020_at	GSVIVP00033369001	O04630 Threonyl-tRNA synthetase, mitochondrial precursor related cluster	-1.8
VVTU5125_s_at	CF203829	O80567 Expressed protein related cluster	-1.8
VVTU8019_at	GSVIVP00033191001	Q2QNI2 Zinc knuckle family protein, putative, expressed related cluster	-1.8
VVTU23314_at	GSVIVP00019254001	Q6YYY1 Hypothetical protein P0604E01.3 related cluster	-1.8
VVTU2679_at	GSVIVP00037013001	AF021809 Vitis vinifera putative sucrose transporter(VvSUC12) mRNA, complete cds.	-1.8
VVTU40669_at	GSVIVP00016818001	Q9SN39 Hypothetical protein F28A21.160 related cluster	-1.8
VVTU1587_at	GSVIVP00015770001	O82167 Expressed protein related cluster	-1.8
VVTU25333_s_at	GSVIVP00016577001	Q1SAG1 Hypothetical protein related cluster	-1.8
VVTU31781_at	TC66512	UPI0000DB7FA7 Cluster related to UPI0000DB7FA7; PREDICTED: similar to Chromodomain helicase-DNA-bindinG protein Mi-2 homolog (ATP-dependent helicase Mi-2) (dMi-2)	-1.8
VVTU4179_at	GSVIVP00024021001	Q1SIP8 Probable indole-3-acetate beta-Glucosyltransferase T27E13.11- Arabidopsis thaliana related cluster	-1.8
VVTU40377_s_at	GSVIVP00019369001	Q0JQ42 Os01g0182900 protein related cluster	-1.8
VVTU9993_at	GSVIVP00026678001	Q9FHS8 Similarity to unknown protein related cluster	-1.8
VVTU5711_at	TC57198	Q3ECE6 Protein At1g70620 related cluster	-1.8
VVTU24257_at	GSVIVP00026375001	Q7XAM0 Hypothetical protein OJ1753_E03.102 related cluster	-1.8
VVTU20500_at	VVTU20500_at	Q8GYD2 Hypothetical protein At4g29170 F19B15_200 related cluster	-1.8
VVTU17156_s_at	GSVIVP00009515001	Q2V065 Cytochrome P450 related cluster	-1.8
VVTU22822_at	GSVIVP00000116001	Q8LFH1 Aldose 1-epimerase-like protein related cluster	-1.8
VVTU2447_s_at	GSVIVP00002792001	Q9C5U1 Histidine kinase related cluster	-1.8
VVTU27807_at	GSVIVP00037008001	Q5MG89 Putative cyclophilin type Peptidyl-prolyl cis-trans isomerase related cluster	-1.8
VVTU506_at	GSVIVP00019544001	Q8GS71 Kinesin-like protein related cluster	-1.8
VVTU17047_s_at	DT007771	Q8GZX2 Hypothetical protein OSJNBa0090O10.11 related cluster	-1.8
VVTU3146_at	GSVIVP00021808001	Q9LTY0 Arabidopsis thaliana genomic DNA, chromosome 5, TAC clone:K9P8 related cluster	-1.8
VVTU37074_at	TC59709	Q1S6D5 Zinc finger, BED-type predicted; HAT dimerisation related cluster	-1.8
VVTU38324_at	GSVIVP00025685001	Q9LDL9 Gb|AAF34828.1 related cluster	-1.8
VVTU22495_at	VVTU22495_at	Q1S650 Hypothetical protein related cluster	-1.8
VVTU25068_at	GSVIVP00027543001	Q8H821 Putative receptor-like protein kinase related cluster	-1.8
VVTU9115_at	GSVIVP00023895001	Q9SIW2 Hypothetical protein At2g16390 related cluster	-1.8
VVTU20244_at	GSVIVP00027063001	Q9SZE6 Putative serine threonine-specific receptor protein kinase related cluster	-1.8
VVTU35008_s_at	CB974204	Q0INB8 Os12g0484700 protein related cluster	-1.8
VVTU9300_at	GSVIVP00025233001	Q0WQF4 Hypothetical protein At1g50500 related cluster	-1.8
VVTU11184_s_at	GSVIVP00014554001	Q6IDJ6 At1g35180 related cluster	-1.8
VVTU24810_at	GSVIVP00004384001	Q84W19 Putative methylenetetrahydrofolatedehydrogenase related cluster	-1.8
VVTU5978_at	GSVIVP00027253001	Q9SZ15 Hypothetical protein F10M23.90 related cluster	-1.8
VVTU4064_at	GSVIVP00002265001	Q1SF68 AAA ATPase; DEAD DEAH box helicase, N-terminal related cluster	-1.8
VVTU3456_s_at	TC60027	P92567 Hypothetical mitochondrial protein AtMg01410 related cluster	-1.8
VVTU20928_at	GSVIVP00015092001	O04492 F21M12.9 protein related cluster	-1.8
VVTU21248_at	VVTU21248_at	Q7XAU6 Class IV Chitinase related cluster	-1.8
VVTU3329_at	GSVIVP00036251001	Q0IZW9 Os09g0544800 protein related cluster	-1.8
VVTU8273_at	GSVIVP00026716001	Q8LFH3 Hypothetical protein related cluster	-1.8
VVTU25917_at	GSVIVP00033040001	Q67ZU5 MRNA, complete cds, clone: RAFL22-73-C24 related cluster	-1.8
VVTU26089_at	CA815066	Q9LZS6 Cucumisin-like protein related cluster	-1.8
VVTU27395_at	GSVIVP00006396001	Q9FZP1 Heparanase-like protein 3 precursor related cluster	-1.8
VVTU4499_at	GSVIVP00010646001	Q9LKZ4 receptor-like protein kinase 3 related cluster	-1.8
VVTU3383_at	TC64019	Q8H0V4 Hypothetical protein At3g62300 related cluster	-1.8
VVTU8444_s_at	GSVIVP00029610001	Q9LJX4 RNA binding protein-like related cluster	-1.8
VVTU21991_at	GSVIVP00035415001	Q71RI4 Resistance protein related cluster	-1.8
VVTU39754_at	GSVIVP00022272001	Q3E7L8 Protein At5g19400 related cluster	-1.8
VVTU8524_at	GSVIVP00020712001	Q76KU6 DNA methyltransferase related cluster	-1.8
VVTU5955_at	GSVIVP00026832001	O80833 Hypothetical protein At2g45860 related cluster	-1.8
VVTU26325_at	GSVIVP00008396001	O22724 F11P17.6 protein related cluster	-1.9
VVTU4593_at	CD012443	Q5I6E8 3-phosphoinositide-dependent protein kinase-1 related cluster	-1.9
VVTU5748_at	GSVIVP00017147001	Q1SL72 Ubiquitin related cluster	-1.9
VVTU11101_at	GSVIVP00018645001	Q0DFY8 Os05g0562400 protein related cluster	-1.9
VVTU35004_at	GSVIVP00014801001	Q9FMZ0 Arabidopsis thaliana genomic DNA, chromosome 5, P1 clone:MJB21 related cluster	-1.9
VVTU13462_s_at	TC65427	Q0JB38 Os04g0558700 protein related cluster	-1.9
VVTU25364_s_at	GSVIVP00036697001	O64760 Putative translation Initiation factor eIF-2B epsilon subunit related cluster	-1.9
VVTU35976_at	GSVIVP00029039001	Q8GYL6 Regulator of chromosome condensation like related cluster	-1.9
VVTU29247_at	GSVIVP00032285001	UPI000034F164 Cluster related to UPI000034F164; XI-I; motor protein binding	-1.9

Probe set	Unique Gene ID	Annotation	Fold-change
VVTU5530_at	CD798919	O22845 Putative calcium bindinG protein related cluster	-1.9
VVTU9573_at	GSVIVP00011441001	Q1SDL2 Von Willebrand factor, type C; Zinc finger, RING-type; Zinc finger, C6HC-type; Zinc finger, C2H2-type; ATP-dependent helicase HrpA related cluster	-1.9
VVTU14233_at	GSVIVP00001437001	Q0DIM8 Os05g0376300 protein related cluster	-1.9
VVTU305_at	GSVIVP00010861001	Q94F11 T12E18.6:T12E18.7 protein related cluster	-1.9
VVTU4_at	GSVIVP00035066001	Q93WI8 Urease related cluster	-1.9
VVTU22981_at	GSVIVP00035375001	Q1SKS5 SAM (And some other nucleotide) binding motif related cluster	-1.9
VVTU5046_at	GSVIVP00026418001	O81316 F6N15.20 protein related cluster	-1.9
VVTU7965_at	GSVIVP00030757001	Q9LIN2 nodulin-like protein protein related cluster	-1.9
VVTU20879_at	GSVIVP00002439001	Q9FJ26 DNA polymerasealpha subunit IV (Primase)-like protein related cluster	-1.9
VVTU14115_at	GSVIVP00034901001	Q8W240 GT-2 factor related cluster	-1.9
VVTU26423_at	GSVIVP00008726001	Q9SHL3 Hypothetical protein At2g17550 related cluster	-1.9
VVTU18050_at	GSVIVP00013480001	Q9ATU2 Putative Cytochrome P450 related cluster	-1.9
VVTU34351_at	GSVIVP00012883001	Q9ZPL3 Pulvinus outward-rectifying channel for potassium SPOCK1 related cluster	-1.9
VVTU6310_at	GSVIVP00014357001	Q8LMP9 Hypothetical protein OSJNBa0011L14.12 related cluster	-1.9
VVTU25466_at	GSVIVP00020997001	Q9LXF2 Hypothetical protein F8M21_190 related cluster	-1.9
VVTU8547_at	TC64727	Q8RV04 Expressed protein related cluster	-1.9
VVTU21101_s_at	GSVIVP00000516001	Q9FYG4 F1N21.11 related cluster	-1.9
VVTU1582_at	GSVIVP00036277001	Q9SII5 Expressed protein related cluster	-1.9
VVTU11123_at	GSVIVP00026967001	Q9M310 Hypothetical protein F2A19.190 related cluster	-1.9
VVTU10763_at	VVTU10763_at	Q34937 MURF2 protein related cluster	-1.9
VVTU2754_at	GSVIVP00023582001	Q9LYC6 Glutaredoxin-like protein related cluster	-1.9
VVTU4276_at	GSVIVP00029891001	Q2VT54 DC1.2-like related cluster	-1.9
VVTU10179_at	GSVIVP00016650001	Q9M0E5 Hypothetical protein AT4g29380 related cluster	-1.9
VVTU269_at	GSVIVP00035000001	P37124 Cytochrome P450 77A2 related cluster	-1.9
VVTU34302_at	GSVIVP00035338001	Q0JCZ2 Os04g0442200 protein related cluster	-1.9
VVTU493_at	GSVIVP00034860001	Q6Q4D0 Protein BRUSHY 1 related cluster	-1.9
VVTU9379_at	GSVIVP00021264001	O04098 receptor-kinase isolog, 5 partial; 115640-113643 related cluster	-1.9
VVTU34367_at	GSVIVP00031472001	Q6ZB67 Hypothetical protein OJ1119_B10.8 related cluster	-1.9
VVTU1297_at	GSVIVP00034294001	Q8LAL2 auxin-responsive protein IAA26 related cluster	-1.9
VVTU37828_at	TC63750	Q1SPW3 Hypothetical protein related cluster	-1.9
VVTU6152_at	GSVIVP00021510001	Q259M6 H0723C07.6 protein related cluster	-1.9
VVTU9292_at	GSVIVP00002733001	P42347 Phosphatidylinositol 3-kinase, Root isoform related cluster	-1.9
VVTU5480_at	GSVIVP00018040001	Q64M78 Putative AML1 related cluster	-1.9
VVTU6474_at	GSVIVP00010653001	Q8H7A8 Hypothetical protein related cluster	-1.9
VVTU13074_at	GSVIVP00008087001	Q10EQ6 SWIB complex BAF60b domain-containing protein, putative, expressed related cluster	-1.9
VVTU24884_at	GSVIVP00031456001	Q1SZG0 Xylose isomerase related cluster	-1.9
VVTU8050_at	GSVIVP00029238001	Q10LY8 Expressed protein related cluster	-1.9
VVTU8696_at	GSVIVP00006913001	Q0WR89 Probable Xyloglucan endotransglucosylase hydrolase protein 8 precursor related cluster	-1.9
VVTU7325_at	GSVIVP00024955001	Q1S4F5 Hypothetical protein related cluster	-1.9
VVTU40525_at	TC54741	Q1S8Y2 Hypothetical protein related cluster	-1.9
VVTU40783_s_at	TC62804	Q2QNF1 Retrotransposon protein, putative, unclassified related cluster	-1.9
VVTU7009_at	GSVIVP00004261001	Q1SG93 Hypothetical protein related cluster	-1.9
VVTU2294_at	GSVIVP00038841001	Q9MAT9 F13M7.10 protein related cluster	-1.9
VVTU3198_at	GSVIVP00005660001	Q8H0U2 Hypothetical protein At2g12400 related cluster	-1.9
VVTU3293_at	GSVIVP00029321001	Q1SIA7 Hypothetical protein related cluster	-1.9
VVTU16312_at	GSVIVP00007254001	Q8L733 Hypothetical protein At4g12130 related cluster	-1.9
VVTU25312_at	CO819619	Q7XNY0 OSJNBb0015N08.11 protein related cluster	-1.9
VVTU27673_at	GSVIVP00022053001	Q9M1P3 Protein kinase-like protein related cluster	-1.9
VVTU3250_at	TC54578	Q6T2Z2 Cyclin-dependent kinase inhibitor 1;2 related cluster	-1.9
VVTU8131_s_at	GSVIVP00002241001	Q9AUZ8 Putative phosphatase subunit, 3 -partial related cluster	-1.9
VVTU12973_at	GSVIVP00029906001	Q8VXX5 Monocopper oxidase-like protein SKS1 precursor related cluster	-1.9
VVTU6015_at	GSVIVP00015232001	Q9XHD8 Cryptochrome 1 related cluster	-1.9
VVTU38599_at	TC67818	Q36379 Atp6 protein related cluster	-1.9
VVTU11839_s_at	GSVIVP00000417001	P12459 tubulin beta-1 chain related cluster	-1.9
VVTU3530_at	GSVIVP00020392001	Q6K884 Transducin-like related cluster	-1.9
VVTU15104_at	GSVIVP00010316001	Q1SIS3 peptidase M14, carboxypeptidase A related cluster	-1.9
VVTU7535_x_at	GSVIVP00016966001	Q2QNF1 Retrotransposon protein, putative, unclassified related cluster	-1.9
VVTU17517_at	TC69010	Q53IN6 Ubiquitin related cluster	-1.9
VVTU39693_at	GSVIVP00008924001	Q2Z1Z0 Cinnamyl alcohol dehydrogenase related cluster	-1.9
VVTU11825_at	GSVIVP00030331001	Q9LEC9 Alpha-glucosidase related cluster	-1.9
VVTU14781_at	TC64024	Q1SKI6 Hypothetical protein related cluster	-1.9
VVTU3701_at	GSVIVP00033182001	Q9FGY8 Similarity to unknown protein related cluster	-1.9
VVTU2237_at	GSVIVP00010958001	Q8LJQ5 LEC1-like protein related cluster	-1.9
VVTU9806_at	GSVIVP00003819001	Q9FFL2 Nucleolar protein-like related cluster	-1.9
VVTU10348_at	GSVIVP00031663001	Q9SL02 DNA repair protein RAD50 related cluster	-1.9
VVTU21095_at	GSVIVP00028668001	Q1RU52 Disease Resistance protein; AAA ATPase related cluster	-1.9
VVTU146_at	GSVIVP00014640001	Q9MAJ4 F27F5.23 related cluster	-1.9
VVTU16165_at	GSVIVP00009706001	Q7Y0B7 Putative Myb-like DNA-bindinG protein related cluster	-1.9
VVTU38295_at	GSVIVP00032409001	Q1S5S0 Protein kinase related cluster	-1.9
VVTU14976_at	GSVIVP00023357001	Q1SJS5 Zinc finger, RING-type; RINGv related cluster	-1.9
VVTU22294_at	VVTU22294_at	UPI00000A2CBF Cluster related to UPI00000A2CBF; P0408G07.7	-1.9
VVTU12226_at	TC55284	Q8LBL4 Putative Thaumatin-like protein related cluster	-1.9
VVTU10015_at	TC59250	UPI000034F4BB Cluster related to UPI000034F4BB; unknown protein	-1.9

Probe set	Unique Gene ID	Annotation	Fold-change
VVTU522_at	VVTU522_at	Q2IB39 cellulose synthase 5 related cluster	-1.9
VVTU5242_at	GSVIVP00034660001	Q7XJU2 BHLH transcription factor related cluster	-1.9
VVTU8056_at	GSVIVP00019039001	Q0J999 Os04g0665000 protein related cluster	-1.9
VVTU10442_at	VVTU10442_at	UPI00005886B6 Cluster related to UPI00005886B6; PREDICTED: similar to lipoxygenase homology domains 1, partial	-1.9
VVTU7770_at	VVTU7770_at	O80604 T27I1.15 protein related cluster	-1.9
VVTU4463_s_at	GSVIVP00003171001	Q93Y12 Alpha glucosidase-like protein related cluster	-1.9
VVTU4998_s_at	GSVIVP00022213001	Q8W0W3 Transcription initiation factor IIB related cluster	-1.9
VVTU15746_s_at	GSVIVP00022579001	Q96416 Cyclophylin related cluster	-1.9
VVTU5806_s_at	TC67876	Q8GWZ6 Protein SENSITIVITY TO RED LIGHT REDUCED 1 related cluster	-1.9
VVTU12161_at	TC70642	Q9LUQ6 60S ribosomal protein L19-2 related cluster	-1.9
VVTU26815_at	GSVIVP00025191001	Q0J1P2 Os09g0423200 protein related cluster	-1.9
VVTU987_at	GSVIVP00034469001	Q1STK3 Hypothetical protein related cluster	-1.9
VVTU38881_at	TC54915	Q1SRN1 Integrase, catalytic region related cluster	-1.9
VVTU16789_s_at	CB921958	Q1SGL5 Hypothetical protein related cluster	-1.9
VVTU22838_at	GSVIVP00033928001	Q9ZVC9 Mutator-like transposase related cluster	-1.9
VVTU26041_at	GSVIVP00018236001	Q1T2K4 IQ calmodulin-binding region related cluster	-1.9
VVTU29338_at	GSVIVP00021399001	Q5QLR7 S-receptor kinase-like related cluster	-1.9
VVTU35089_s_at	GSVIVP00031453001	Q1SZF7 Protein kinase related cluster	-1.9
VVTU1209_at	GSVIVP00000310001	Q9SSD2 F18B13.15 protein related cluster	-1.9
VVTU21034_at	GSVIVP00002702001	Q2Q062 Carbonic anhydrase 3 related cluster	-1.9
VVTU23783_x_at	DT034200	Q944A7 AT4g35230 F23E12_210 related cluster	-1.9
VVTU5427_at	GSVIVP00006109001	O65759 Histone H2AX related cluster	-1.9
VVTU6664_at	GSVIVP00030800001	O22161 F-box protein family, AtFBX5 related cluster	-1.9
VVTU22347_at	VVTU22347_at	Q9CAB3 Putative ABC transporter; 82503-78258 related cluster	-1.9
VVTU500_at	GSVIVP00031633001	Q1SA28 Down-regulated in metastasis related cluster	-1.9
VVTU3377_at	GSVIVP00030118001	O49434 HyuC-like protein related cluster	-1.9
VVTU6179_at	GSVIVP00009629001	Q940Y1 AT4g22540 F7K2_120 related cluster	-1.9
VVTU8935_at	GSVIVP00002903001	Q1T007 Cyclin-like F-box; Serine threonine Protein phosphatase, BSU1 related cluster	-1.9
VVTU4859_at	GSVIVP00001831001	Q9SK95 Similar to peptide transporter related cluster	-1.9
VVTU10308_at	VVTU10308_at	Q5Z938 Hypothetical protein P0659D09.46 related cluster	-1.9
VVTU40733_at	TC52511	Q9M3C7 Hypothetical protein T26I12.120 related cluster	-1.9
VVTU9335_at	GSVIVP00029398001	Q8LII5 Hypothetical protein OJ1167_G06.116 related cluster	-1.9
VVTU7942_at	GSVIVP00031100001	Q8W4E3 Hypothetical protein related cluster	-1.9
VVTU35104_s_at	GSVIVP00003054001	Q01197 Protein E6 related cluster	-1.9
VVTU15460_at	GSVIVP00014068001	Q6Z8D4 Hypothetical protein P0459B01.34 related cluster	-1.9
VVTU7249_at	GSVIVP00019338001	Q8VZH2 AT4g33090 F4I10_20 related cluster	-1.9
VVTU35155_at	GSVIVP00010426001	Q9LFE2 WD40-repeat protein related cluster	-1.9
VVTU13427_at	GSVIVP00005262001	Q7XQI1 OSJNBa0067K08.9 protein related cluster	-1.9
VVTU7791_at	GSVIVP00017527001	Q682J0 Hypothetical protein At5g22120 related cluster	-1.9
VVTU1502_at	GSVIVP00019542001	Q0WQM7 Putative RNA-bindinG protein related cluster	-1.9
VVTU2858_at	GSVIVP00030638001	Q9AT33 EndoXyloglucan transferase related cluster	-1.9
VVTU3816_at	GSVIVP00021442001	Q1T2V3 Concanavalin A-like lectin Glucanase related cluster	-1.9
VVTU8344_at	GSVIVP00028822001	P82658 Glucosyltransferase 19 kDa protein, chloroplast precursor related cluster	-1.9
VVTU23522_at	GSVIVP00006962001	Q84N26 Leucine-rich repeat protein related cluster	-1.9
VVTU9610_at	GSVIVP00022249001	Q5JKW8 Putative Cytochrome P450-dependent fatty acid hydroxylase related cluster	-1.9
VVTU21483_s_at	GSVIVP00034207001	Q84WI8 NADP-specific glutatamate dehydrogenase, putative related cluster	-1.9
VVTU35996_at	GSVIVP00034353001	Q1SP75 WD40-like related cluster	-1.9
VVTU7806_at	DT034613	Q8GT59 Hypothetical protein 276 related cluster	-1.9
VVTU3525_at	GSVIVP00023649001	Q3E6N2 Protein At3g07140 related cluster	-1.9
VVTU40149_at	TC51987	UPI0000D56244 Cluster related to UPI0000D56244; PREDICTED: similar to CG7269-PA, isoform A	-1.9
VVTU8406_at	GSVIVP00015042001	Q9SXC5 T17H3.9 related cluster	-1.9
VVTU14496_s_at	GSVIVP00020661001	Q8VZ30 Hypothetical protein At5g40500 related cluster	-1.9
VVTU8937_at	GSVIVP00034000001	Q1RTC8 Remorin, C-terminal region related cluster	-1.9
VVTU6341_at	GSVIVP00019893001	Q9M8K0 F28L1.12 protein related cluster	-1.9
VVTU14889_at	CF603609	Q9MAS8 F13M7.22 protein related cluster	-1.9
VVTU4936_at	GSVIVP00022983001	Q710C3 Sigma factor related cluster	-1.9
VVTU6519_s_at	GSVIVP00006412001	Q1RTE9 AIG1 related cluster	-1.9
VVTU6937_s_at	GSVIVP00031642001	Q9LIS3 Nucleotide sugar epimerase-like protein related cluster	-1.9
VVTU14933_at	GSVIVP00027943001	Q9SYM9 Hypothetical protein T30F21.14 related cluster	-1.9
VVTU15778_at	GSVIVP00017686001	Q8LKN9 Nam-like protein 15 related cluster	-1.9
VVTU8354_at	CF405035	Q9FX08 T3F24.2 protein related cluster	-1.9
VVTU4614_at	GSVIVP00012227001	Q9LSZ5 Arabidopsis thaliana genomic DNA, chromosome 5, P1 clone:MQM1 related cluster	-2.0
VVTU4409_at	GSVIVP00019088001	Q0GPH0 BZIP transcription factor bZIP80 related cluster	-2.0
VVTU31689_x_at	CB345404	Q25563 tubulin alpha-13 chain related cluster	-2.0
VVTU5753_at	GSVIVP00031312001	Q69XJ5 Putative allyl alcohol dehydrogenase related cluster	-2.0
VVTU17406_s_at	GSVIVP00035852001	Q6DXR6 Putative permease related cluster	-2.0
VVTU7572_at	GSVIVP00017845001	Q4R1J3 SEL-1 related cluster	-2.0
VVTU13022_s_at	GSVIVP00016926001	Q9FXS4 NtEIG-A1 protein related cluster	-2.0
VVTU3458_at	GSVIVP00024062001	Q93XR7 Fructose-6-phosphate 2-kinase fructose-2,6-bisphosphatase related cluster	-2.0
VVTU1122_at	GSVIVP00008668001	Q3ED49 Protein At1g29980 related cluster	-2.0
VVTU9668_at	GSVIVP00017821001	Q94GS4 Hypothetical protein OSJNBb0022E02.3 related cluster	-2.0
VVTU18078_s_at	GSVIVP00034459001	Q5ZDJ3 Acetyltransferase 1-like related cluster	-2.0
VVTU4300_at	GSVIVP00023815001	Q9LY72 Hypothetical protein MAA21_60 related cluster	-2.0

Probe set	Unique Gene ID	Annotation	Fold-change
VVTU6302_at	GSVIVP00032609001	Q1S410 AT3g01400 T13O15_4, putative related cluster	-2.0
VVTU14459_at	GSVIVP00005961001	Q0D4Z0 Os07g0596000 protein related cluster	-2.0
VVTU21149_at	GSVIVP00001024001	Q5VQ09 Putative Kinesin related cluster	-2.0
VVTU12371_at	GSVIVP00023726001	Q40434 PSI-D1 precursor related cluster	-2.0
VVTU9112_at	GSVIVP00014643001	Q7XI89 Hypothetical protein P0453G03.31 related cluster	-2.0
VVTU8271_at	GSVIVP00023772001	Q8SA64 NIMA-related protein kinase related cluster	-2.0
VVTU21478_at	CF201099	O80784 nodulin-like protein related cluster	-2.0
VVTU28425_s_at	GSVIVP00035203001	O04893 Alpha-glucosidase precursor related cluster	-2.0
VVTU1135_at	GSVIVP00010759001	Q43846 Soluble Starch synthase 3, chloroplast precursor related cluster	-2.0
VVTU25952_at	GSVIVP00026851001	Q5XNL4 Resistance protein-like protein related cluster	-2.0
VVTU31915_at	CB343912	Q10SV7 Expressed protein related cluster	-2.0
VVTU6387_at	GSVIVP00030712001	Q9SAF0 F3F19.19 protein related cluster	-2.0
VVTU7623_at	GSVIVP00030538001	AF378125 Vitis vinifera GAI-like protein 1 (GAI1) gene, complete cds.	-2.0
VVTU6355_at	GSVIVP00019494001	AY337615 Vitis vinifera zeaxanthin epoxidase (ZEP) mRNA, complete cds.	-2.0
VVTU2271_at	GSVIVP00031430001	Q9FKL3 Similarity to protein kinase related cluster	-2.0
VVTU9582_at	GSVIVP00025602001	Q8GWK2 Putative AP2 domain transcription factor related cluster	-2.0
VVTU40295_at	CF204759	Q2QP84 Vesicle tethering family protein, putative, expressed related cluster	-2.0
VVTU5428_at	GSVIVP00026255001	Q9LPC7 F22M8.7 protein related cluster	-2.0
VVTU38452_at	GSVIVP00031799001	Q09K01 Caffeic acid O-methyltransferase related cluster	-2.0
VVTU1756_s_at	TC63012	Q2YHP6 Snakin-like cysteine rich protein related cluster	-2.0
VVTU15241_at	GSVIVP00024162001	Q0DHQ5 Os05g0450600 protein related cluster	-2.0
VVTU16294_at	GSVIVP00004251001	Q949K3 Suppressor-like protein related cluster	-2.0
VVTU17059_at	CA810758	Q1SQS4 ClpX, ATPase regulatory subunit related cluster	-2.0
VVTU11168_at	GSVIVP00003979001	Q1S0L2 Hypothetical protein related cluster	-2.0
VVTU27444_at	GSVIVP00016129001	Q647I4 Putative RNA-dependent RNA polymerase RdRP2 related cluster	-2.0
VVTU37743_at	GSVIVP00014143001	Q9T0C2 Hypothetical protein T4F9.110 related cluster	-2.0
VVTU6717_at	GSVIVP00001030001	Q9LN98 F5O11.21 related cluster	-2.0
VVTU10785_at	GSVIVP00017683001	Q9LG94 Hypothetical protein P0408F06.22 related cluster	-2.0
VVTU39443_at	GSVIVP00018142001	Q93YU5 Probable exocyst complex component 4 related cluster	-2.0
VVTU39404_at	GSVIVP00003867001	Q5XF11 At4g35930 related cluster	-2.0
VVTU4892_at	GSVIVP00036274001	Q2GMP5 Predicted protein related cluster	-2.0
VVTU22933_s_at	TC61798	Q3HRQ2 Glyoxal oxidase related cluster	-2.0
VVTU16502_at	GSVIVP00026366001	O22128 Expressed protein related cluster	-2.0
VVTU9331_at	GSVIVP00028544001	Q9SD94 Hypothetical protein F13G24.100 related cluster	-2.0
VVTU23834_at	DT032780	O00369 P40 related cluster	-2.0
VVTU7217_s_at	GSVIVP00036498001	Q9XIM0 Expressed protein related cluster	-2.0
VVTU5275_at	GSVIVP00012868001	Q1T1V6 AAA ATPase related cluster	-2.0
VVTU11469_at	GSVIVP00028488001	Q1S805 DENN; dDENN; uDENN related cluster	-2.0
VVTU11076_at	GSVIVP00024231001	Q941T0 Pentatricopeptide (PPR) repeat-containing protein-like related cluster	-2.0
VVTU12676_s_at	GSVIVP00033843001	Q5PXG5 Cyclin dependent kinase inhibitor related cluster	-2.0
VVTU4385_at	TC59645	Q304A5 Protein At4g19160 related cluster	-2.0
VVTU7420_at	GSVIVP00026978001	O82340 Hypothetical protein At2g46300 related cluster	-2.0
VVTU8542_at	GSVIVP00000597001	Q9LVF2 Arabidopsis thaliana genomic DNA, chromosome 3, P1 clone: MIL23 related cluster	-2.0
VVTU4181_s_at	GSVIVP00037337001	Q9ZQH4 Putative nitrilase related cluster	-2.0
VVTU4576_at	GSVIVP00023470001	Q9M9P0 expansin-A13 precursor related cluster	-2.0
VVTU12204_at	GSVIVP00027189001	Q9C585 Ubiquitin-specific protease-like protein related cluster	-2.0
VVTU38234_at	TC65489	Q3EAA4 Protein At4g05520 related cluster	-2.0
VVTU9566_at	TC68622	Q0IZG4 Os09g0570500 protein related cluster	-2.0
VVTU5791_at	GSVIVP00010274001	Q0IWN4 Os10g0498300 protein related cluster	-2.0
VVTU26029_at	GSVIVP00027918001	Q9LH75 Ac transposase-like protein related cluster	-2.0
VVTU16843_at	GSVIVP00011948001	Q2V469 Protein At2g22660 related cluster	-2.0
VVTU9368_at	GSVIVP00015534001	Q658B2 Putative prolyl endopeptidase related cluster	-2.0
VVTU12295_at	GSVIVP00019026001	Q7XJE6 Metacaspase 1 related cluster	-2.0
VVTU28012_at	GSVIVP00017910001	Q9ZRW0 Nucleolar protein related cluster	-2.0
VVTU39696_at	TC63554	Q1SUI9 Reverse transcriptase-beet retrotransposon related cluster	-2.0
VVTU6655_at	GSVIVP00018753001	Q9SJI6 Hypothetical protein At2g42700 related cluster	-2.0
VVTU782_at	GSVIVP00015313001	O80378 181 related cluster	-2.0
VVTU8234_at	GSVIVP00030123001	Q67XI5 Hypothetical protein At4g20030 related cluster	-2.0
VVTU342_at	GSVIVP00028063001	Q8VYF4 Hypothetical protein At3g62660 related cluster	-2.0
VVTU14435_at	GSVIVP00016864001	Q6NM36 At5g20110 related cluster	-2.0
VVTU5649_at	GSVIVP00018848001	Q9FNN2 WD-repeat protein-like related cluster	-2.0
VVTU7340_at	GSVIVP00024105001	O64455 Ca2+ H+ exchanger related cluster	-2.0
VVTU22592_at	GSVIVP00015673001	Q39639 Glycerol-3-phosphate acyltransferase, chloroplast precursor related cluster	-2.0
VVTU37524_at	GSVIVP00028112001	Q8VZF3 At2g47390 T8I13.23 related cluster	-2.0
VVTU10970_at	GSVIVP00014091001	Q9LU57 Arabidopsis thaliana genomic DNA, chromosome 5, P1 clone:MWD22 related cluster	-2.0
VVTU12712_at	GSVIVP00010892001	Q2HT37 IQ calmodulin-binding region; Apoptosis regulator Bcl-2 protein, BAG related cluster	-2.0
VVTU1197_at	GSVIVP00037469001	Q9SCU4 Hypothetical protein T18N14.20 related cluster	-2.0
VVTU13053_at	GSVIVP00020448001	Q8LAU9 GATA transcription factor 1 related cluster	-2.0
VVTU10689_at	GSVIVP00034349001	Q1SP72 Ferredoxin related cluster	-2.0
VVTU16934_x_at	GSVIVP00003318001	P46256 Fructose-bisphosphate aldolase, cytoplasmic isozyme 1 related cluster	-2.0
VVTU31021_at	CB911122	O22060 sucrose-phosphate synthase 1 related cluster	-2.0
VVTU9607_at	GSVIVP00015307001	Q84WU6 auxin response factor 17 related cluster	-2.0
VVTU12225_s_at	GSVIVP00029129001	Q9FKH4 Similarity to beta-1 related cluster	-2.0

Probe set	Unique Gene ID	Annotation	Fold-change
VVTU1747_at	GSVIVP00025944001	O48768 Expressed protein related cluster	-2.0
VVTU24481_at	GSVIVP00028020001	Q9SC88 Gamma-tubulin complex component 4 homolog related cluster	-2.0
VVTU5776_at	VVTU5776_at	Q93ZW1 Hypothetical protein At5g20680 related cluster	-2.0
VVTU13723_x_at	GSVIVP00011134001	Q84KB0 Pol protein related cluster	-2.0
VVTU2554_at	TC65191	Q1SSF2 Protein F10A5.7 [imported]-Arabidopsis thaliana-related related cluster	-2.0
VVTU5353_at	GSVIVP00031956001	Q9C814 CLP protease regulatory subunit CLPX, putative; 15869-19379 related cluster	-2.0
VVTU9220_at	GSVIVP00028825001	Q9ZUB7 F5O8.34 related cluster	-2.0
VVTU4777_at	TC68807	Q259I7 H0101F08.6 protein related cluster	-2.0
VVTU32896_at	CB343395	Q1SPW3 Hypothetical protein related cluster	-2.0
VVTU6614_at	GSVIVP00000997001	Q93YV5 Hypothetical protein At1g78420 related cluster	-2.0
VVTU390_at	GSVIVP00006699001	Q3Y6V1 cellulose synthase-like protein CslG related cluster	-2.0
VVTU2125_at	GSVIVP00017546001	Q0GLE8 Dof4 related cluster	-2.0
VVTU1844_at	GSVIVP00024075001	Q9LUZ6 Arabidopsis thaliana genomic DNA, chromosome 5, P1 clone:MZN1 related cluster	-2.0
VVTU24236_at	GSVIVP00018389001	Q9STF6 Subtilisin-like proteinase homolog related cluster	-2.0
VVTU5551_at	GSVIVP00003502001	Q9M6S0 Pumilio domain-containinG protein PPD1 related cluster	-2.0
VVTU12189_at	GSVIVP00034177001	Q1SK06 GroEL-like chaperone, ATPase related cluster	-2.0
VVTU14635_at	GSVIVP00030704001	Q2QTS8 Regulator of chromosome condensation, putative, expressed related cluster	-2.0
VVTU16374_s_at	TC61429	Q9M0N8 Hypothetical protein AT4g09550 related cluster	-2.0
VVTU31174_at	CB343723	Q3V4Z9 translation Initiation factor IF-1, chloroplast related cluster	-2.0
VVTU16360_at	CF207187	Q9LHJ9 Protein phosphatase 2C related cluster	-2.0
VVTU10703_at	GSVIVP00025582001	Q1S6X1 Disease Resistance protein; Short-chain dehydrogenase reductase SDR related cluster	-2.0
VVTU7532_at	CA817358	Q8S9A7 Glucosyltransferase-2 related cluster	-2.0
VVTU20102_at	GSVIVP00026003001	Q1SKW7 Hypothetical protein related cluster	-2.0
VVTU8058_at	GSVIVP00002559001	Q2PF02 Hypothetical protein related cluster	-2.0
VVTU20744_at	VVTU20744_at	Q9LUB8 polygalacturonase related cluster	-2.0
VVTU7904_at	GSVIVP00030414001	Q0JLD5 Os01g0607200 protein related cluster	-2.0
VVTU37974_s_at	GSVIVP00020058001	O82623 T9A4.1 protein related cluster	-2.0
VVTU35618_s_at	GSVIVP00002473001	Q8RW34 Beta 1,3-glycosyltransferase-like protein I related cluster	-2.0
VVTU34497_at	GSVIVP00021559001	Q8H0W4 Hypothetical protein At3g11570 related cluster	-2.0
VVTU8952_at	GSVIVP00031145001	Q8SA64 NIMA-related protein kinase related cluster	-2.0
VVTU7113_at	GSVIVP00009273001	Q4R1I9 Anthocyanidin 5,3-O-Glucosyltransferase related cluster	-2.0
VVTU10984_at	GSVIVP00019637001	Q1SC08 BTB POZ; NPH3 related cluster	-2.0
VVTU1352_at	GSVIVP00025807001	Q9ASQ3 At1g17220 F20D23_8 related cluster	-2.0
VVTU1636_at	GSVIVP00017802001	Q1SN14 Hypothetical protein related cluster	-2.0
VVTU26090_at	CF607237	Q9LNV9 F22G5.26 related cluster	-2.0
VVTU10673_at	GSVIVP00020011001	Q9ZVZ4 T25N20.9 related cluster	-2.0
VVTU9603_at	GSVIVP00015456001	Q3ED78 Protein At1g21570 related cluster	-2.0
VVTU15926_at	GSVIVP00006691001	O22987 T19F6.16 protein related cluster	-2.0
VVTU15662_at	GSVIVP00013498001	Q1ENZ8 Hypothetical protein related cluster	-2.1
VVTU5702_at	GSVIVP00025915001	Q9SVH5 Hypothetical protein F21C20.70 related cluster	-2.1
VVTU4462_s_at	GSVIVP00027767001	Q2LFC4 AGO1-1 related cluster	-2.1
VVTU17368_at	TC67473	Q6RZV0 transposase-like protein related cluster	-2.1
VVTU36685_s_at	GSVIVP00035827001	Q9MAG2 F12M16.29 related cluster	-2.1
VVTU21758_at	GSVIVP00009973001	Q93VD3 At1g30270 F12P21_6 related cluster	-2.1
VVTU3443_at	GSVIVP00032833001	Q9FRS5 F22O13.8 related cluster	-2.1
VVTU40141_at	GSVIVP00008763001	Q9FPN1 Putative Cytochrome P450 related cluster	-2.1
VVTU504_at	VVTU504_at	Q9S842 Hypothetical protein F14G6.4 related cluster	-2.1
VVTU17084_at	GSVIVP00031954001	Q9C814 CLP protease regulatory subunit CLPX, putative; 15869-19379 related cluster	-2.1
VVTU7023_at	GSVIVP00015565001	Q9SGQ9 T23E18.18 related cluster	-2.1
VVTU801_s_at	GSVIVP00002301001	Q6NQ48 Hypothetical protein At1g34320 related cluster	-2.1
VVTU11008_at	VVTU11008_at	Q2V301 Protein At5g50175 related cluster	-2.1
VVTU1186_at	GSVIVP00031196001	Q8RW14 Aux IAA protein related cluster	-2.1
VVTU1310_at	GSVIVP00016814001	O81016 Probable pleiotropic drug Resistance protein 4 related cluster	-2.1
VVTU10686_at	GSVIVP00035014001	Q0JDG0 Os04g0405800 protein related cluster	-2.1
VVTU15210_s_at	GSVIVP00001819001	Q9LEN5 Hypothetical protein related cluster	-2.1
VVTU3343_at	GSVIVP00020602001	Q9SRP6 T21P5.16 protein related cluster	-2.1
VVTU24714_at	GSVIVP00038586001	Q5ZDI8 Hypothetical protein P0686E09.31 related cluster	-2.1
VVTU39812_at	GSVIVP00027786001	Q9LNC4 F9P14.9 protein related cluster	-2.1
VVTU11371_at	GSVIVP00037516001	Q6YXW7 Hypothetical protein OSJNBa0064G16.1 related cluster	-2.1
VVTU7006_at	GSVIVP00028663001	Q8W0V0 Type IIB calcium ATPase related cluster	-2.1
VVTU1648_at	GSVIVP00035337001	Q5DMX3 DRP related cluster	-2.1
VVTU4000_at	GSVIVP00024560001	Q9FF49 Retroelement pol Polyprotein-like related cluster	-2.1
VVTU22959_s_at	GSVIVP00037237001	O82471 Protein phosphatase-2C related cluster	-2.1
VVTU9708_x_at	GSVIVP00005893001	Q2MJ18 Cytochrome P450 monooxygenase CYP72A59 related cluster	-2.1
VVTU4127_s_at	GSVIVP00023900001	Q5QML5 Hypothetical protein P0454H12.5 related cluster	-2.1
VVTU249_at	VVTU249_at	Q1SS78 ABI3-interActinG protein 2 related cluster	-2.1
VVTU8422_at	CO818376	Q7XPT4 OSJNBa0083N12.6 protein related cluster	-2.1
VVTU28198_x_at	GSVIVP00026925001	Q8MA69 ATP synthase epsilon subunit related cluster	-2.1
VVTU1845_at	GSVIVP00030755001	Q9M7E8 Eukaryotic Initiation factor 4B related cluster	-2.1
VVTU15437_at	GSVIVP00022867001	Q688N0 Hypothetical protein P0605G01.9 related cluster	-2.1
VVTU22370_at	GSVIVP00033493001	Q94IP3 Cold-induced glucosyl transferase related cluster	-2.1
VVTU17174_at	CD012735	Q9SRT0 F21O3.7 protein related cluster	-2.1
VVTU30048_at	GSVIVP00001684001	UPI0000163575 Cluster related to UPI0000163575; unknown protein	-2.1
VVTU37837_at	TC59620	Q2QNF1 Retrotransposon protein, putative, unclassified related cluster	-2.1

Probe set	Unique Gene ID	Annotation	Fold-change
VVTU17425_s_at	TC63217	Q0ZJ11 Acetyl-CoA carboxylase carboxyltransferase beta subunit related cluster	-2.1
VVTU38436_at	GSVIVP00028835001	Q9SGH2 T13O15.10 protein related cluster	-2.1
VVTU13895_at	GSVIVP00037103001	Q5VM93 Hypothetical protein P0046E09.31 related cluster	-2.1
VVTU11343_at	VVTU11343_at	Q9LUA1 Arabidopsis thaliana genomic DNA, chromosome 3, P1 clone: MPE11 related cluster	-2.1
VVTU4541_at	GSVIVP00016889001	Q2HVT7 Hypothetical protein related cluster	-2.1
VVTU5609_at	GSVIVP00019364001	Q1SQT7 Protein kinase related cluster	-2.1
VVTU1900_at	GSVIVP00034700001	Q1SUH6 Hypothetical protein related cluster	-2.1
VVTU26190_at	CF606513	Q4BHU6 Integrase, catalytic region related cluster	-2.1
VVTU28398_x_at	CD801065	Q0KIP5 Polyprotein, putative related cluster	-2.1
VVTU34419_at	GSVIVP00013497001	Q0D4B1 Os07g0636900 protein related cluster	-2.1
VVTU409_at	VVTU409_at	Q9LU58 Gb|AAF43949.1 related cluster	-2.1
VVTU569_at	GSVIVP00036418001	O81129 Phosphatidylinositol 4-kinase related cluster	-2.1
VVTU7603_at	GSVIVP00026013001	Q75ID3 Expressed protein related cluster	-2.1
VVTU19897_at	VVTU19897_at	Q3EAL6 Protein At3g51880 related cluster	-2.1
VVTU5607_at	GSVIVP00021803001	Q8W5R2 Shoot gravitropism 2 related cluster	-2.1
VVTU6245_at	GSVIVP00016688001	Q1SRU6 Hypothetical protein related cluster	-2.1
VVTU9631_at	GSVIVP00037761001	Q940R3 AT5g62650 MRG21_7 related cluster	-2.1
VVTU16759_at	GSVIVP00001825001	Q9SK95 Similar to peptide transporter related cluster	-2.1
VVTU11501_at	GSVIVP00034821001	Q9FLF8 auxin-independent growth promoter-like protein related cluster	-2.1
VVTU5641_at	GSVIVP00029160001	P49063 Exopolygalacturonase clone GBGA483 precursor related cluster	-2.1
VVTU10256_at	GSVIVP00032098001	Q5Z9R9 Putative amino acid transporter related cluster	-2.1
VVTU8892_at	GSVIVP00032880001	Q6K992 Hypothetical protein OJ1520_C09.39 related cluster	-2.1
VVTU39590_at	GSVIVP00021675001	UPI00000ABE00 Cluster related to UPI00000ABE00; P0665D10.14	-2.1
VVTU24708_at	GSVIVP00016721001	Q6K7U1 Putative Pentatricopeptide (PPR) repeat-containinG protein related cluster	-2.1
VVTU5061_at	GSVIVP00023113001	Q8H1N1 Hypothetical protein At2g37390 F3G5.18 related cluster	-2.1
VVTU5661_s_at	GSVIVP00028415001	UPI000034F426 Cluster related to UPI000034F426; unknown protein	-2.1
VVTU11401_at	GSVIVP00015672001	Q84SE0 Putative TPA: Cgi67 serine protease related cluster	-2.1
VVTU38409_at	TC56169	Q7XUA5 OSJNBa0019D11.15 protein related cluster	-2.1
VVTU16778_at	GSVIVP00037609001	Q9FGI4 Arabidopsis thaliana genomic DNA, chromosome 5, P1 clone:MPA22 related cluster	-2.1
VVTU2356_at	GSVIVP00018272001	Q9M1S3 RNA binding protein-like related cluster	-2.1
VVTU39542_at	TC60670	O49432 Hypothetical protein AT4g20050 related cluster	-2.1
VVTU4418_at	GSVIVP00034814001	Q9SKH2 Expressed protein related cluster	-2.1
VVTU22211_at	DT030643	Q6I5S8 Putative Polyprotein related cluster	-2.1
VVTU1875_at	GSVIVP00006915001	Q0PJH5 Myb transcription factor Myb73 related cluster	-2.1
VVTU38983_x_at	TC66028	Q9M3J0 NAD(P)H-quinone oxidoreductase chain 4, chloroplast (EC 1.6.5.-) (NAD(P)H dehydrogenase, chain 4) related cluster	-2.1
VVTU3194_s_at	TC63633	Q9FN38 Arabidopsis thaliana genomic DNA, chromosome 5, TAC clone:K19P17 related cluster	-2.1
VVTU769_s_at	GSVIVP00031570001	Q8RVP5 Class III peroxidase related cluster	-2.1
VVTU38395_at	GSVIVP00027033001	Q8RX80 AT4g23250 F21P8_140 related cluster	-2.1
VVTU6480_at	CD798395	Q9SB56 Hypothetical protein F22K18.190 related cluster	-2.1
VVTU24609_at	GSVIVP00028359001	Q6ZLQ0 Putative ERD4 protein related cluster	-2.1
VVTU12259_at	GSVIVP00025472001	Q9FK56 Arabidopsis thaliana genomic DNA, chromosome 5, P1 clone:MRG7 related cluster	-2.1
VVTU13388_at	GSVIVP00002127001	Q1SXL0 Surfeit locus 5 related cluster	-2.1
VVTU24564_at	GSVIVP00003147001	Q4TVR0 NRG1 related cluster	-2.1
VVTU11173_at	GSVIVP00019262001	Q7XUI5 OSJNBa0005N02.6 protein related cluster	-2.1
VVTU1947_at	GSVIVP00002585001	Q9LHE3 Nucleoid chloroplast DNA-binding protein-like related cluster	-2.1
VVTU23842_at	DT032651	Q9M1J4 Hypothetical protein F24I3.140 related cluster	-2.1
VVTU12042_at	GSVIVP00001032001	Q6U5H33-hydroxy-3-methylglutaryl synthase related cluster	-2.1
VVTU11245_at	GSVIVP00035588001	O05000 NADH-ubiquinone oxidoreductase chain 2 related cluster	-2.1
VVTU38050_at	GSVIVP00035935001	Q1S047 MCM; Nucleic acid-binding, OB-fold related cluster	-2.1
VVTU11481_at	GSVIVP00025242001	Q8RX60 At1g79720 F19K16_30 related cluster	-2.1
VVTU11283_at	GSVIVP00019226001	Q1S1T0 DDT related cluster	-2.1
VVTU2249_at	GSVIVP00021217001	P93472 Cell elongation protein diminuto related cluster	-2.1
VVTU22689_at	GSVIVP00019064001	Q7X703 OSJNBa0079A21.4 protein related cluster	-2.1
VVTU4721_at	GSVIVP00021029001	Q9C520 Hypothetical protein F10D13.5 related cluster	-2.1
VVTU30333_at	GSVIVP00025642001	Q93YX7 Type IIB calcium ATPase related cluster	-2.1
VVTU1482_at	GSVIVP00027724001	Q53KT8 Hypothetical protein related cluster	-2.1
VVTU1101_at	GSVIVP00016372001	Q7XZU2 SAC domain protein 3 related cluster	-2.1
VVTU39562_at	GSVIVP00027196001	Q9LJY1 Gb|AAC31834.1 related cluster	-2.1
VVTU15148_at	GSVIVP00024547001	Q9SKN5 auxin response factor 10 related cluster	-2.1
VVTU6026_at	GSVIVP00037667001	Q10A81 Expressed protein related cluster	-2.2
VVTU35432_at	GSVIVP00029442001	Q1SIQ3 CAMP response element binding (CREB) protein related cluster	-2.2
VVTU5253_at	TC70054	Q9ZQG9-2 Isoform 2 of Q9ZQG9 related cluster	-2.2
VVTU20644_s_at	GSVIVP00013844001	Q6YZI7 Hypothetical protein P0562A06.9 related cluster	-2.2
VVTU2217_at	GSVIVP00026982001	O82337 Expressed protein related cluster	-2.2
VVTU233_at	GSVIVP00018460001	Q10BD5 FtsJ-like methyltransferase family protein, putative, expressed related cluster	-2.2
VVTU16903_at	CB911242	Q6SSJ2 Phytocalpain related cluster	-2.2
VVTU11720_at	VVTU11720_at	Q0ZPV0 CXE carboxylesterase related cluster	-2.2
VVTU10942_at	GSVIVP00037838001	Q9XF71 CLC-Nt2 protein related cluster	-2.2
VVTU13318_at	GSVIVP00035830001	Q5JNJ1 Putative Trehalose-6-phosphate synthase phosphatase related cluster	-2.2
VVTU4981_at	GSVIVP00024267001	Q10NF9 Retrotransposon protein, putative, unclassified, expressed related cluster	-2.2

Probe set	Unique Gene ID	Annotation	Fold-change
VVTU15686_at	GSVIVP00010277001	Q2HUE1 Glycosyltransferase sugar-binding region containing DXD motif; Alpha 1,4-glycosyltransferase conserved region related cluster	-2.2
VVTU35753_s_at	GSVIVP00031340001	O65570 Villin-4 related cluster	-2.2
VVTU34750_s_at	GSVIVP00019328001	Q9FKY4 Gb|AAD32907.1 related cluster	-2.2
VVTU5837_at	GSVIVP00016348001	UPI000034EFAB Cluster related to UPI000034EFAB; guanyl-nucleotide exchange factor	-2.2
VVTU24543_at	GSVIVP00001255001	AY159562 Vitis vinifera putative O-methyltransferase (COMT) mRNA, partial cds.	-2.2
VVTU14788_at	DT031278	UPI00000A7565 Cluster related to UPI00000A7565; putative DEIH-box RNA DNA helicase	-2.2
VVTU6295_at	GSVIVP00027317001	Q1SIC8 Hypothetical protein related cluster	-2.2
VVTU1273_at	GSVIVP00002688001	Q40161 Polygalacturonase-1 non-catalytic subunit beta precursor related cluster	-2.2
VVTU9050_at	GSVIVP00023840001	O24542 auxin-induced protein 22D related cluster	-2.2
VVTU37888_at	GSVIVP00010959001	Q2A9Q8 DNA-binding protein-related related cluster	-2.2
VVTU35484_at	GSVIVP00001491001	Q8GXE3 Hypothetical protein At1g49975 F2J10.22 related cluster	-2.2
VVTU200_at	GSVIVP00026604001	Q1SW57 HEC Ndc80p related cluster	-2.2
VVTU37412_s_at	GSVIVP00031449001	Q1SZF1 Allergen V5 Tpx-1 related related cluster	-2.2
VVTU732_at	GSVIVP00031334001	O22613 Plasma membrane proton ATPase related cluster	-2.2
VVTU16446_at	CF204972	Q1SYX5 Protein kinase related cluster	-2.2
VVTU30737_at	GSVIVP00018816001	Q7F956 OSJNBb0002J11.24 protein related cluster	-2.2
VVTU1264_at	CD008996	Q1S4P9 Isopenicillin N synthetase; KH, type 1 related cluster	-2.2
VVTU6365_at	GSVIVP00016345001	Q93ZD8 AT5g20880 F22D1_50 related cluster	-2.2
VVTU6561_at	GSVIVP00017670001	Q10E18 CCT motif family protein, expressed related cluster	-2.2
VVTU8850_at	GSVIVP00013327001	Q3EBW4 Protein At2g22720 related cluster	-2.2
VVTU33508_at	CA809124	Q9SPU5 Putative transposase protein related cluster	-2.2
VVTU3276_at	GSVIVP00016505001	O24518 Unconventional Myosin related cluster	-2.2
VVTU12158_at	GSVIVP00026270001	O64634 Hypothetical protein At2g45540 related cluster	-2.2
VVTU37430_at	GSVIVP00025817001	Q1S928 Hypothetical protein related cluster	-2.2
VVTU7467_s_at	TC59606	P48786 Pathogenesis-related homeodomain protein related cluster	-2.2
VVTU1537_at	GSVIVP00015373001	Q84WK2 At1g44770 related cluster	-2.2
VVTU16841_s_at	TC59660	Q2QX93 WD repeat domain 48, putative, expressed related cluster	-2.2
VVTU3973_at	GSVIVP00026339001	Q9SDZ2 2 -hydroxy isoflavone dihydroflavonol reductase homolog related cluster	-2.2
VVTU2066_at	GSVIVP00032197001	Q1SK20 Zinc finger, C2H2-type related cluster	-2.2
VVTU35181_s_at	GSVIVP00024383001	Q9SI96 Histone H2B.3 related cluster	-2.2
VVTU4709_at	GSVIVP00035871001	Q9LP48 F28N24.12 protein related cluster	-2.2
VVTU8033_s_at	GSVIVP00011797001	Q2V9C4 Hypothetical protein related cluster	-2.2
VVTU2336_at	GSVIVP00008759001	Q93YZ5 AT5g51550 K17N15_10 related cluster	-2.2
VVTU13271_s_at	GSVIVP00030937001	Q1RU46 Response regulator receiver related cluster	-2.2
VVTU3274_at	GSVIVP00030283001	Q9FNN3 Similarity to unknown protein related cluster	-2.2
VVTU15677_at	CN548040	Q67WQ6 Putative Senescence-associated protein 5 related cluster	-2.2
VVTU4920_at	GSVIVP00017032001	Q5Z818 Squamosa promoter bindinG protein 2-like related cluster	-2.2
VVTU10518_at	GSVIVP00021010001	Q9FLY2 Arabidopsis thaliana genomic DNA, chromosome 5, P1 clone:MUL8 related cluster	-2.2
VVTU10268_at	GSVIVP00021713001	Q9LZR0 homeodomain-like protein related cluster	-2.2
VVTU4624_s_at	GSVIVP00025996001	Q10LV7 Possible Photosystem II reaction center Psb27 protein, putative, expressed related cluster	-2.2
VVTU3446_s_at	GSVIVP00014282001	Q700J9 Putative Pathogenesis-related protein related cluster	-2.2
VVTU11025_at	GSVIVP00027707001	Q9LNN8 F8L10.1 protein related cluster	-2.2
VVTU27998_at	GSVIVP00000456001	UPI00000A2CBF Cluster related to UPI00000A2CBF; P0408G07.7	-2.2
VVTU945_at	GSVIVP00030153001	Q2MGR3 Hypothetical protein related cluster	-2.2
VVTU7592_s_at	GSVIVP00023516001	Q5Z7F9 Hypothetical protein OSJNBa0090E14.34 related cluster	-2.2
VVTU25629_at	CN545286	Q84M57 Hypothetical protein OSJNBa0059E14.19 related cluster	-2.2
VVTU4283_at	GSVIVP00006953001	Q5HZ54 At2g33510 related cluster	-2.2
VVTU739_at	GSVIVP00020600001	Q949T9 Hypothetical protein At5g14370 related cluster	-2.2
VVTU34644_at	GSVIVP00037508001	Q10LV1 Serine threonine-protein kinase AtPK19, putative, expressed related cluster	-2.2
VVTU3668_at	GSVIVP00032780001	Q1SI22 BTB POZ; NPH3 related cluster	-2.2
VVTU5358_at	GSVIVP00022133001	Q9SJJ7 RING-H2 finger protein ATL2E related cluster	-2.2
VVTU15969_at	CF603296	Q9FHN2 Ripening-related protein-like related cluster	-2.2
VVTU16253_at	GSVIVP00028114001	Q9LZQ1 Hypothetical protein T12C14_90 related cluster	-2.2
VVTU40219_x_at	TC64774	Q1SBU3 Hypothetical protein related cluster	-2.2
VVTU14546_at	GSVIVP00030267001	Q1SBD8 Unnamed protein product; contains similarity to elicitor-inducible receptor EIR gene_id:T5M7.12 related cluster	-2.2
VVTU8927_at	GSVIVP00014909001	O04388 A-type Cyclin related cluster	-2.2
VVTU24151_at	GSVIVP00031508001	Q10NQ5 Harpin-induced protein 1 containing protein, expressed related cluster	-2.2
VVTU3885_at	TC70962	Q04129 Wound induced protein related cluster	-2.2
VVTU39097_at	GSVIVP00023494001	Q0PZK5 Phytoene synthase protein related cluster	-2.2
VVTU5816_s_at	GSVIVP00020624001	Q45GG8 Hypothetical protein related cluster	-2.2
VVTU3279_at	GSVIVP00026364001	Q9LPC6 F22M8.8 protein related cluster	-2.2
VVTU37691_at	GSVIVP00018224001	Q9FT57 Hypothetical protein T25B15_20 related cluster	-2.2
VVTU9218_s_at	GSVIVP00008089001	Q9LPZ0 T23J18.11 related cluster	-2.2
VVTU9564_at	GSVIVP00038083001	Q9FMB4 Arabidopsis thaliana genomic DNA, chromosome 5, TAC clone:K15E6 related cluster	-2.2
VVTU327_at	GSVIVP00012886001	Q8LCS9 Hypothetical protein related cluster	-2.2
VVTU6609_at	GSVIVP00036307001	Q9SZL8 Hypothetical protein F20D10.300 related cluster	-2.3
VVTU9304_at	GSVIVP00011799001	Q3EBA7 Protein At3g07525 related cluster	-2.3
VVTU24082_at	GSVIVP00030732001	Q944N6 Cytochrome P450 related cluster	-2.3
VVTU35990_at	GSVIVP00001484001	Q9ZVK7 Hypothetical protein At2g14680 related cluster	-2.3
VVTU36905_at	GSVIVP00035833001	Q5Z938 Hypothetical protein P0659D09.46 related cluster	-2.3
VVTU104_at	GSVIVP00002427001	Q9LTK8 Arabidopsis thaliana genomic DNA, chromosome 5, BAC clone:F14A1 related cluster	-2.3

Probe set	Unique Gene ID	Annotation	Fold-change
VVTU22924_s_at	GSVIVP00003869001	O65629 Hypothetical protein T19K4.50 related cluster	-2.3
VVTU15542_at	GSVIVP00030649001	Q2V4B4 Protein At2g01910 related cluster	-2.3
VVTU4131_s_at	GSVIVP00026137001	Q1SHH7 auxin responsive SAUR protein related cluster	-2.3
VVTU35915_x_at	GSVIVP00037593001	Q94FS9 Gamma-aminobutyrate transaminase subunit precursor related cluster	-2.3
VVTU8242_s_at	GSVIVP00016474001	Q2R122 Early Flowering 4, putative, expressed related cluster	-2.3
VVTU602_at	VVTU602_at	Q93VU9 Hypothetical protein P0487H02.28 related cluster	-2.3
VVTU2731_s_at	GSVIVP00019025001	Q76E23 Eukaryotic translation Initiation factor 4G related cluster	-2.3
VVTU35251_at	GSVIVP00015210001	Q8L5C7 UDP-glucuronosyltransferase related cluster	-2.3
VVTU5264_s_at	GSVIVP00013985001	Q1SZU4 Protein kinase PKN PRK1, effector related cluster	-2.3
VVTU19282_s_at	DT039600	Q8LBL4 Putative Thaumatin-like protein related cluster	-2.3
VVTU37002_at	GSVIVP00001466001	Q10QB5 Radical SAM enzyme, Cfr family protein, expressed related cluster	-2.3
VVTU34193_at	GSVIVP00024727001	Q9M1T5 Serine threonine-protein kinase-like protein related cluster	-2.3
VVTU226_s_at	GSVIVP00023708001	Q9FEA1 Anthocyanin 1 related cluster	-2.3
VVTU8556_at	CF371922	Q700D2 Hypothetical protein related cluster	-2.3
VVTU17405_at	GSVIVP00019141001	O22155 Hypothetical protein At2g44970 related cluster	-2.3
VVTU1314_at	GSVIVP00018907001	Q9SJG7 Hypothetical protein At2g42900 related cluster	-2.3
VVTU35014_at	GSVIVP00032341001	Q9SDM0 Vacuolar H+-ATPase catalytic subunit related cluster	-2.3
VVTU2994_at	GSVIVP00015727001	Q9SDM5 P-glycoprotein related cluster	-2.3
VVTU721_at	GSVIVP00010755001	Q5BMC5 Phosphomannose isomerase related cluster	-2.3
VVTU7865_at	GSVIVP00020304001	Q2V3I5 Protein At4g14310 related cluster	-2.3
VVTU14598_at	GSVIVP00002527001	Q9FFS1 Gb|AAC67354.1 related cluster	-2.3
VVTU2459_at	GSVIVP00029518001	Q9LZ72 3-ketoacyl-CoA synthase 21 related cluster	-2.3
VVTU29946_x_at	GSVIVP00022096001	Q1SRU1 Glycosyl transferase, family 2 related cluster	-2.3
VVTU22827_at	GSVIVP00035461001	Q1SWP8 ATP-requiring DNA helicase RecQ related cluster	-2.3
VVTU5732_at	GSVIVP00000088001	Q2VTE6 HDZip I protein related cluster	-2.3
VVTU5875_at	GSVIVP00018455001	Q1SYU9 Peptidylprolyl isomerase, FKBP-type related cluster	-2.3
VVTU25365_at	GSVIVP00036192001	Q9SZM5 Hypothetical protein F20M13.10 related cluster	-2.3
VVTU3856_at	GSVIVP00011910001	Q3E9P4 Protein At4g37080 related cluster	-2.3
VVTU33991_at	CB001813	Q10M20 Expressed protein related cluster	-2.3
VVTU5201_at	GSVIVP00036030001	Q10QA7 Expressed protein related cluster	-2.3
VVTU1330_at	GSVIVP00029418001	DQ465410 Vitis vinifera KUP2 mRNA, complete cds.	-2.3
VVTU32485_s_at	GSVIVP00005636001	Q9ZQF5 Putative RING-H2 Zinc finger protein related cluster	-2.3
VVTU35221_x_at	GSVIVP00020243001	Q6QGY1 Merlot proline-rich protein 2 related cluster	-2.3
VVTU7314_at	GSVIVP00032280001	Q7Y051 Paa2 P-type ATPase related cluster	-2.3
VVTU38022_at	TC58434	Q1T092 Hypothetical protein related cluster	-2.3
VVTU7736_at	GSVIVP00024319001	Q9AYE4 Expressed protein related cluster	-2.3
VVTU35813_at	GSVIVP00032018001	Q8VXE0 Glyceraldehyde-3-phosphate dehydrogenase related cluster	-2.3
VVTU9824_at	GSVIVP00036226001	Q9FFC7 Alanyl-tRNA synthetase related cluster	-2.3
VVTU2420_at	GSVIVP00032739001	Q1RW45 Hypothetical protein related cluster	-2.3
VVTU3658_at	GSVIVP00035577001	Q0ZJ39 Maturase K related cluster	-2.3
VVTU11141_at	GSVIVP00029722001	Q8L5A1 Hypothetical protein related cluster	-2.3
VVTU10943_at	GSVIVP00027197001	Q6ZK57 Putative RNA recognition motif (RRM)-containinG protein related cluster	-2.3
VVTU15769_at	CN007015	UPI0000583DCA Cluster related to UPI0000583DCA; PREDICTED: Hypothetical protein XP_794817	-2.3
VVTU16628_at	GSVIVP00035285001	Q8H6X8 GSK-3-like protein MsK4 related cluster	-2.3
VVTU12755_at	GSVIVP00027325001	Q9AV43 Hypothetical protein OSJNBa0001O14.25 related cluster	-2.3
VVTU12999_at	GSVIVP00000513001	O22977 T19F6.6 protein related cluster	-2.3
VVTU3157_at	GSVIVP00024354001	Q1S928 Hypothetical protein related cluster	-2.3
VVTU23403_at	DV222456	Q9FFT3 genomic DNA, chromosome 5, P1 clone:MBG8 related cluster	-2.3
VVTU9554_at	GSVIVP00013881001	Q9SLX3 Sigma factor related cluster	-2.3
VVTU1261_at	GSVIVP00025539001	Q1SHY3 Heat shock protein DnaJ related cluster	-2.3
VVTU6252_at	GSVIVP00000886001	Q2LFC4 AGO1-1 related cluster	-2.3
VVTU8017_at	GSVIVP00037903001	Q6K609 Glutaredoxin-like related cluster	-2.3
VVTU3209_at	GSVIVP00011879001	O81804 Hypothetical protein F8D20.240 related cluster	-2.3
VVTU14337_at	GSVIVP00015749001	Q1S3P1 Helix-loop-helix DNA-binding related cluster	-2.3
VVTU7945_at	GSVIVP00027007001	Q9M159 Hypothetical protein AT4g01040 related cluster	-2.3
VVTU16036_at	GSVIVP00022875001	Q2HTP0 Lipolytic enzyme, G-D-S-L related cluster	-2.3
VVTU6769_at	GSVIVP00030554001	Q9LQU1 F10B6.30 related cluster	-2.3
VVTU1225_at	TC69112	O64835 Expressed protein related cluster	-2.3
VVTU16665_s_at	GSVIVP00036501001	Q6AV32 Putative oxidoreductase related cluster	-2.3
VVTU20468_at	VVTU20468_at	Q94B59 Hypothetical protein T5E8_250 related cluster	-2.3
VVTU20441_at	GSVIVP00000505001	Q1SN07 Hypothetical protein related cluster	-2.4
VVTU6993_at	GSVIVP00027753001	Q9ARP7 Hypothetical protein OSJNBa0010K01.25 related cluster	-2.4
VVTU22566_at	GSVIVP00006705001	Q3Y6V1 cellulose synthase-like protein CslG related cluster	-2.4
VVTU16703_at	GSVIVP00023189001	Q9S7D9 Hypothetical protein F11F8.37 related cluster	-2.4
VVTU25232_at	GSVIVP00037941001	Q2HUL4 Short-chain dehydrogenase reductase SDR related cluster	-2.4
VVTU2746_s_at	GSVIVP00038837001	Q2V3I8 Protein At4g14147 related cluster	-2.4
VVTU14960_at	TC68504	Q1KUN0 Hypothetical protein related cluster	-2.4
VVTU25410_s_at	GSVIVP00036466001	Q6XL72 cytochrome P-450-like protein related cluster	-2.4
VVTU538_at	GSVIVP00031153001	Q10D12 Transferase family protein, expressed related cluster	-2.4
VVTU5699_at	GSVIVP00031934001	Q9SU97 Hypothetical protein T16H5.10 related cluster	-2.4
VVTU6544_at	GSVIVP00023771001	Q1SMI4 Sulphate transporter related cluster	-2.4
VVTU12489_at	GSVIVP00023056001	Q75L11 Probable Histone H2A.6 related cluster	-2.4
VVTU20591_at	GSVIVP00034411001	Q9CA94 Hypothetical protein F19K16.13 related cluster	-2.4
VVTU3508_at	GSVIVP00024439001	Q40269 Protein kinase related cluster	-2.4
VVTU647_at	GSVIVP00038071001	DQ235273 Vitis vinifera brassinosteroid-6-oxidase (BR6OX1) mRNA, complete cds.	-2.4

Probe set	Unique Gene ID	Annotation	Fold-change
VVTU26689_at	GSVIVP00033345001	Q9LG20 F14J16.17 related cluster	-2.4
VVTU20317_at	VVTU20317_at	Q5CAZ5 UDP-xylose phenolic glycosyltransferase related cluster	-2.4
VVTU34063_at	GSVIVP00022521001	Q1SEA8 Hypothetical protein related cluster	-2.4
VVTU8724_at	GSVIVP00005267001	Q8L6Y4 Polycomb protein EmbryoNIC Flower 2 related cluster	-2.4
VVTU15232_at	GSVIVP00028467001	Q2N1D8 CMV 1a interActinG protein 2 related cluster	-2.4
VVTU25616_at	GSVIVP00016049001	Q1T393 IMP dehydrogenase GMP reductase related cluster	-2.4
VVTU2117_at	GSVIVP00020577001	Q32SG1 Protein phosphatase 2A regulatory subunit B related cluster	-2.4
VVTU252_at	VVTU252_at	Q9FJZ5 Arabidopsis thaliana genomic DNA, chromosome 5, TAC clone:K1F13 related cluster	-2.4
VVTU36869_at	TC57678	Q1SS87 Gag-pol Polyprotein-related related cluster	-2.4
VVTU28820_at	CD712848	Q2QM08 Expressed protein related cluster	-2.4
VVTU4947_at	GSVIVP00020705001	Q0JQB6 Os01g0171800 protein related cluster	-2.4
VVTU12822_at	GSVIVP00008852001	UPI00005A5838 Cluster related to UPI00005A5838; PREDICTED: similar to Histone 1, H2ai (predicted)	-2.4
VVTU35262_at	GSVIVP00007879001	Q1STS4 Calcium-binding EF-hand related cluster	-2.4
VVTU10952_at	VVTU10952_at	Q0WW94 Kanadaptin-like protein related cluster	-2.4
VVTU12341_at	GSVIVP00019139001	Q8RXD6 Hypothetical protein At2g44950:At2g44960 related cluster	-2.4
VVTU37316_at	VVTU37316_at	Q51613 , complete genome related cluster	-2.4
VVTU40723_s_at	TC67634	Q1T324 RNA-directed DNA polymerase(Reverse transcriptase); Endonuclease exonuclease phosphatase related cluster	-2.4
VVTU8498_at	GSVIVP00038471001	Q9STK2 Peptidyl-prolyl cis-trans isomerase related cluster	-2.4
VVTU16874_at	GSVIVP00026338001	Q64MA3 Putative single-strand DNA Endonuclease-1 related cluster	-2.4
VVTU35247_at	TC67561	Q1SA49 Response regulator receiver related cluster	-2.4
VVTU38252_at	GSVIVP00022102001	Q6JJ39 Putative adapitin protein related cluster	-2.4
VVTU5426_at	GSVIVP00027202001	Q0J5I8 Os08g0431500 protein related cluster	-2.4
VVTU559_at	GSVIVP00002645001	Q2HVC0 Protein kinase related cluster	-2.4
VVTU27393_s_at	GSVIVP00000672001	Q6NKR3 At3g55600 related cluster	-2.4
VVTU2160_at	GSVIVP00016936001	O48524 Hypothetical protein At2g42160 related cluster	-2.4
VVTU4291_at	GSVIVP00015573001	Q6GKX1 Hypothetical protein At5g42330 related cluster	-2.4
VVTU15002_at	GSVIVP00030061001	Q9FZE2 T1K7.5 protein related cluster	-2.4
VVTU518_at	GSVIVP00026064001	Q9LJ45 PREG1-like negative regulator-like protein related cluster	-2.4
VVTU5243_at	GSVIVP00006008001	Q9LVN8 Similarity to SNF2 RAD54 family related cluster	-2.4
VVTU15149_at	GSVIVP00014274001	Q9LHP4 receptor protein kinase-like protein related cluster	-2.4
VVTU6298_at	GSVIVP00020390001	O22777 Expressed protein related cluster	-2.4
VVTU8482_at	GSVIVP00024971001	Q9FGZ3 Similarity to unknown protein related cluster	-2.4
VVTU14438_at	GSVIVP00033001001	Q9FH28 chaperone protein DnaJ 49 related cluster	-2.4
VVTU21904_s_at	GSVIVP00033380001	Q8VZB8 Hypothetical protein At5g55810; MDF20.25 related cluster	-2.4
VVTU35236_at	VVTU35236_at	Q0D5I1 Os07g0558900 protein related cluster	-2.4
VVTU3392_at	GSVIVP00036104001	O49607 Subtilisin proteinase-like related cluster	-2.4
VVTU23045_at	GSVIVP00014759001	Q3E9P4 Protein At4g37080 related cluster	-2.4
VVTU639_at	GSVIVP00034961001	Q8RWL1 Hypothetical protein At2g36360 related cluster	-2.4
VVTU35067_at	GSVIVP00022262001	Q38848 LRP1 related cluster	-2.4
VVTU35588_x_at	GSVIVP00035992001	Q9SW54 Hypothetical protein T11I11.40 related cluster	-2.4
VVTU621_at	GSVIVP00031931001	Q0E3L1 Os02g0168400 protein related cluster	-2.4
VVTU14281_at	CF605098	Q6ZAA9 Hypothetical protein P0429B05.21 related cluster	-2.4
VVTU6767_at	GSVIVP00012221001	Q39862 Homeobox-leucine zipper protein related cluster	-2.4
VVTU35732_at	GSVIVP00036012001	Q681Q1 auxin-induced protein-like protein related cluster	-2.4
VVTU10672_at	GSVIVP00006959001	Q9FI58 Arabidopsis thaliana genomic DNA, chromosome 5, TAC clone:K3K7 related cluster	-2.5
VVTU5010_at	GSVIVP00036093001	Q8L883 auxin transporter-like protein 5 related cluster	-2.5
VVTU5628_at	GSVIVP00027472001	O81829 Indole-3-acetic acid-amido synthetase GH3.5 related cluster	-2.5
VVTU11508_at	GSVIVP00037142001	Q6AWV1 Probable ribonuclease P protein subunit 2 related cluster	-2.5
VVTU37859_x_at	TC58795	Q7XNY0 OSJNBb0015N08.11 protein related cluster	-2.5
VVTU169_at	GSVIVP00030285001	Q9SAF8 F3F19.27 protein related cluster	-2.5
VVTU12483_at	GSVIVP00029573001	Q94K16 Hypothetical protein F21O3.22 related cluster	-2.5
VVTU11687_at	GSVIVP00037464001	Q10LQ3 Expressed protein related cluster	-2.5
VVTU9021_at	GSVIVP00022593001	Q94D04 Putative p40 related cluster	-2.5
VVTU7593_at	GSVIVP00034404001	P46819 DNA-directed RNA polymerase beta chain related cluster	-2.5
VVTU38012_at	GSVIVP00027806001	Q9LU44 Similarity to transcription or splicing factor related cluster	-2.5
VVTU9243_at	GSVIVP00017243001	Q2R3D7 Exonuclease family protein, expressed related cluster	-2.5
VVTU7674_at	GSVIVP00020927001	Q8GW32 Hypothetical protein related cluster	-2.5
VVTU40684_s_at	GSVIVP00023064001	Q3EAK4 Protein At3g53470 related cluster	-2.5
VVTU351_at	VVTU351_at	Q25A64 H0306F03.11 protein related cluster	-2.5
VVTU10455_at	VVTU10455_at	Q2QWU4 Expressed protein related cluster	-2.5
VVTU17519_at	GSVIVP00013562001	Q6Z688 Putative UDP-glucose Glucosyltransferase related cluster	-2.5
VVTU3227_at	GSVIVP00018616001	Q52UU1 Squamosa promoter binding-like protein related cluster	-2.5
VVTU4681_at	GSVIVP00028904001	Q9M9X3 F18C1.5 protein related cluster	-2.5
VVTU11808_at	GSVIVP00016523001	Q93XX0 Hypothetical protein At4g10430; F7L13.10 related cluster	-2.5
VVTU6331_at	GSVIVP00026258001	Q6AWX7 Growth-regulating factor 12 related cluster	-2.5
VVTU22394_at	GSVIVP00026140001	O82459 Rac GTPase activatinG protein 2 related cluster	-2.5
VVTU27516_at	GSVIVP00024790001	Q1S3J9 Rieske [2Fe-2S] region related cluster	-2.5
VVTU4351_at	GSVIVP00033936001	Q10BC8 HAT family dimerisation domain containing protein, expressed related cluster	-2.5
VVTU5833_s_at	GSVIVP00022255001	Q1SA17 Pleckstrin-like; Regulator of chromosome condensation beta-lactamase- inhibitor protein II; Zinc finger, FYVE PHD-type related cluster	-2.5
VVTU12942_at	GSVIVP00004581001	Q9LVB8 HSR203J protein-like protein related cluster	-2.5
VVTU39221_at	GSVIVP00022610001	Q0D3F4 Os07g0689300 protein related cluster	-2.5

Probe set	Unique Gene ID	Annotation	Fold-change
VVTU17343_at	GSVIVP00036403001	Q3E9R6 Protein At4g34430 related cluster	-2.5
VVTU4314_at	GSVIVP00020758001	Q9SGH2 T13O15.10 protein related cluster	-2.5
VVTU2394_s_at	GSVIVP00032309001	Q8VWP9 Fiddlehead-like protein related cluster	-2.5
VVTU3023_at	GSVIVP00035397001	Q1A3R6 POLLUX protein related cluster	-2.5
VVTU4383_at	GSVIVP00017959001	Q8H965 Phosphatidylglycerol specific phospholipaseC related cluster	-2.5
VVTU6691_at	GSVIVP00034569001	Q3S345 Zinc finger protein-like protein related cluster	-2.5
VVTU31646_x_at	CB345641	Q6YSQ6 Hypothetical protein orf108a related cluster	-2.6
VVTU16712_at	GSVIVP00024567001	Q9LZM8 Putative homeodomain protein related cluster	-2.6
VVTU22403_at	GSVIVP00027120001	Q9M1S8 Probable glutamate carboxypeptidase 2 related cluster	-2.6
VVTU29734_at	GSVIVP00015453001	Q69SU3 Hypothetical protein P0470G10.14 related cluster	-2.6
VVTU26973_at	GSVIVP00015080001	Q6IMT1 SAB related cluster	-2.6
VVTU39442_at	GSVIVP00019530001	Q10SV7 Expressed protein related cluster	-2.6
VVTU7622_at	GSVIVP00000510001	AY043235 Vitis vinifera putative cellulase CEL2 mRNA, partial cds.	-2.6
VVTU501_at	GSVIVP00026004001	Q1W1G0 CK25 related cluster	-2.6
VVTU4792_at	GSVIVP00017887001	Q9M7K3 HAK2 related cluster	-2.6
VVTU4018_s_at	GSVIVP00004655001	Q8S6Y3 Putative ATFP3 related cluster	-2.6
VVTU13045_at	GSVIVP00008891001	Q5JMK3 Putative VAP27 related cluster	-2.6
VVTU16319_at	GSVIVP00035189001	Q94EF5 Putative meiotic serine proteinase related cluster	-2.6
VVTU2826_at	GSVIVP00037187001	Q9FJB8 Gb|AAB63610.1 related cluster	-2.6
VVTU21689_at	GSVIVP00037515001	Q6YWF9 NucleoPorin-like protein related cluster	-2.6
VVTU4864_at	GSVIVP00036960001	Q6L416 Putative ZF-HD Homeobox protein, identical related cluster	-2.6
VVTU16329_at	GSVIVP00032704001	Q9S775 CHD3-type chromatin remodeling factor PICKLE related cluster	-2.6
VVTU10170_at	VVTU10170_at	Q1SP54 Hypothetical protein related cluster	-2.6
VVTU15843_at	GSVIVP00009918001	Q9FJQ3 genomic DNA, chromosome 5, P1 clone:MQN23 related cluster	-2.6
VVTU1633_at	GSVIVP00008438001	Q93VM6 AT4g39900 T5J17_70 related cluster	-2.6
VVTU20633_at	GSVIVP00024817001	Q6NKU3 At1g55340 related cluster	-2.6
VVTU10523_at	GSVIVP00018042001	Q9LY30 Copine-like protein related cluster	-2.6
VVTU11659_at	GSVIVP00034994001	Q940J7 Hypothetical protein related cluster	-2.6
VVTU38174_at	TC65028	Q688X8 putative Heat shock protein, HSP40 related cluster	-2.6
VVTU39817_at	GSVIVP00037662001	Q3E972 Protein At5g23870 related cluster	-2.6
VVTU2752_s_at	GSVIVP00020103001	Q9LIJ8 Arabidopsis thaliana genomic DNA, chromosome 3, BAC clone:F5N5 related cluster	-2.6
VVTU35376_at	GSVIVP00037302001	Q945L6 AT3g11590 F24K9_26 related cluster	-2.6
VVTU11449_at	GSVIVP00025970001	O48626 Centromere kinetochore protein zw10 homolog related cluster	-2.6
VVTU28198_s_at	GSVIVP00029783001	Q8MA69 ATP synthase epsilon subunit related cluster	-2.6
VVTU865_at	GSVIVP00019628001	Q1SED0 Orn DAP Arg decarboxylase 2; protease-associated PA; proteinase inhibitor I9, Subtilisin propeptide related cluster	-2.6
VVTU9073_x_at	GSVIVP00009968001	Q2PEP3 Putative Glucosyltransferase related cluster	-2.6
VVTU2795_at	GSVIVP00001488001	Q8S522 D-type Cyclin related cluster	-2.6
VVTU40146_at	GSVIVP00018271001	Q0WMU6 Hypothetical protein At3g09670 related cluster	-2.6
VVTU15479_at	GSVIVP00034532001	Q9C941 Putative non-phototropic hypocotyl; 25081-26618 related cluster	-2.6
VVTU23302_at	GSVIVP00027255001	Q5Z4M1 Putative microtubule-associated protein related cluster	-2.6
VVTU5959_at	GSVIVP00000450001	Q7XCT3 Expressed protein related cluster	-2.6
VVTU2642_at	GSVIVP00025336001	Q9M3U4 Beta 1-3 Glucanase related cluster	-2.6
VVTU12570_at	GSVIVP00009622001	Q9SXE7 T3P18.6 related cluster	-2.6
VVTU10016_at	GSVIVP00019675001	UPI0000197181 Cluster related to UPI0000197181; unknown protein	-2.6
VVTU15962_at	GSVIVP00004558001	Q1S8C2 Armadillo related cluster	-2.6
VVTU15408_at	GSVIVP00027195001	Q9LK65 Similarity to hemolysin related cluster	-2.6
VVTU8237_at	GSVIVP00024984001	Q9LEB8 Common plant regulatory factor 5 related cluster	-2.6
VVTU13121_s_at	CF200899	Q84LH8 PIF3 like basic Helix Loop Helix protein related cluster	-2.6
VVTU22531_at	GSVIVP00026845001	Q6XWB7 Resistance protein Cvi2 related cluster	-2.6
VVTU37485_at	GSVIVP00035986001	Q1S388 YEATS related cluster	-2.6
VVTU3507_at	TC62219	Q2HV27 Hypothetical protein related cluster	-2.7
VVTU3589_at	GSVIVP00015324001	Q9LLS0 Putative phosphatidylinositol 4-phosphate 5-kinase related cluster	-2.7
VVTU22796_s_at	GSVIVP00010417001	Q1SF39 Zinc finger, RING-type related cluster	-2.7
VVTU13990_at	GSVIVP00023017001	Q9M378 TATA box bindinG protein (TBP) associated factor (TAF)-like protein related cluster	-2.7
VVTU39744_at	TC59567	Q1SBU3 Hypothetical protein related cluster	-2.7
VVTU32255_s_at	GSVIVP00028587001	Q2TPW5 seed storage protein related cluster	-2.7
VVTU31911_at	CB343938	O22795 50S ribosomal protein L28, chloroplast precursor related cluster	-2.7
VVTU13611_at	GSVIVP00014847001	Q6Z107 Putative receptor protein kinase PERK1 related cluster	-2.7
VVTU10376_at	GSVIVP00032526001	Q9XGI4 Cyclin A2 related cluster	-2.7
VVTU8470_at	CA817147	Q42962 phosphoglycerate kinase, cytosolic related cluster	-2.7
VVTU8752_at	GSVIVP00027060001	Q8VXX4 Putative replication factor C related cluster	-2.7
VVTU6309_at	GSVIVP00030688001	Q7XUI6 OSJNBa0005N02.4 protein related cluster	-2.7
VVTU35312_at	GSVIVP00019320001	Q5NDD2 Putative Myb transcription factor related cluster	-2.7
VVTU8493_at	GSVIVP00035356001	Q0ITH9 Os11g0243300 protein related cluster	-2.7
VVTU12835_at	GSVIVP00031370001	Q9FKM3 Similarity to AAA-type ATPase related cluster	-2.7
VVTU4306_at	GSVIVP00037509001	Q10LV1 Serine threonine-protein kinase AtPK19, putative, expressed related cluster	-2.7
VVTU39519_x_at	TC65085	Q2QNF1 Retrotransposon protein, putative, unclassified related cluster	-2.7
VVTU12088_at	GSVIVP00033163001	Q1SWA4 Helix-loop-helix DNA-binding related cluster	-2.7
VVTU6067_s_at	TC66389	Q8GUP3 Hypothetical protein At4g31880 related cluster	-2.7
VVTU4430_at	GSVIVP00003249001	Q9LMR0 F7H2.8 protein related cluster	-2.7
VVTU1895_at	GSVIVP00021481001	O80437 Glycerol-3-phosphate acyltransferase 6 related cluster	-2.7
VVTU14370_at	CF373179	Q1T4I5 Hypothetical protein related cluster	-2.7
VVTU14860_at	GSVIVP00010604001	Q3SC87 ACI13 related cluster	-2.7
VVTU9869_at	TC67844	Q8VY54 Hypothetical protein At1g06500 related cluster	-2.7

Probe set	Unique Gene ID	Annotation	Fold-change
VVTU24830_at	GSVIVP00016044001	Q9CAL2 Hypothetical protein F24J13.10 related cluster	-2.7
VVTU39764_s_at	GSVIVP00024306001	Q7X9Q3 expansin related cluster	-2.7
VVTU9200_at	GSVIVP00012449001	Q0Q097 AP2 EREBP transcription factor AINTEGUMENTA-like related cluster	-2.7
VVTU10495_at	VVTU10495_at	Q1S579 Hypothetical protein related cluster	-2.7
VVTU7076_x_at	GSVIVP00011890001	Q1SPW3 Hypothetical protein related cluster	-2.7
VVTU1659_at	GSVIVP00030338001	Q8RWD0 Zinc finger protein CONSTANS-LIKE 16 related cluster	-2.7
VVTU2801_s_at	GSVIVP00027690001	Q94BM7 Putative Phytochrome A supressor spa1 protein related cluster	-2.7
VVTU13703_at	GSVIVP00001938001	Q9LX31 Tetrapyrrole-binding protein, chloroplast precursor related cluster	-2.7
VVTU3053_at	GSVIVP00021322001	Q7XBQ1 Phantastica transcription factor related cluster	-2.7
VVTU2343_at	GSVIVP00034550001	O24329 Hypothetical protein related cluster	-2.7
VVTU7075_at	GSVIVP00024899001	O23230 Trichohyalin like protein related cluster	-2.8
VVTU25274_at	GSVIVP00034220001	Q1RTQ9 Hypothetical protein related cluster	-2.8
VVTU21695_at	VVTU21695_at	Q0E4H9 Os02g0118800 protein related cluster	-2.8
VVTU40198_at	TC52735	Q75L11 Probable Histone H2A.6 related cluster	-2.8
VVTU22672_at	GSVIVP00003615001	Q6EPR2 Enhancer of polycomb-like protein related cluster	-2.8
VVTU21748_at	VVTU21748_at	Q8SA93 Putative Polyprotein related cluster	-2.8
VVTU6893_s_at	GSVIVP00026920001	Q84LI7 Polygalacturonase-like protein related cluster	-2.8
VVTU9338_at	GSVIVP00022798001	Q9LUL5 Emb|CAB66100.1 related cluster	-2.8
VVTU21865_s_at	GSVIVP00015703001	O82293 Expressed protein related cluster	-2.8
VVTU2835_at	GSVIVP00007419001	Q6EP75 Hypothetical protein P0135D07.45 related cluster	-2.8
VVTU2862_at	GSVIVP00029183001	Q94A22 AT4g28240 F26K10_120 related cluster	-2.8
VVTU293_at	GSVIVP00016640001	Q9M0F0 Hypothetical protein AT4g29310 related cluster	-2.8
VVTU1440_at	GSVIVP00002554001	Q84LI7 Polygalacturonase-like protein related cluster	-2.8
VVTU27061_at	GSVIVP00033023001	Q9FH23 Arabidopsis thaliana genomic DNA, chromosome 5, TAC clone:K20J1 related cluster	-2.8
VVTU21589_at	GSVIVP00034327001	O82319 Hypothetical protein At2g25780 related cluster	-2.8
VVTU4751_at	GSVIVP00028647001	Q9LYR0 Hypothetical protein T22N19_110 related cluster	-2.8
VVTU10509_at	GSVIVP00014863001	Q9FJ91 Dbj|BAA78737.1 related cluster	-2.8
VVTU10745_at	GSVIVP00036270001	Q8S348 auxin-induced SAUR-like protein related cluster	-2.8
VVTU40393_at	GSVIVP00015132001	Q6T2Z7 Cyclin d2 related cluster	-2.8
VVTU3103_at	GSVIVP00015380001	Q9LPF5 T12C22.3 protein related cluster	-2.8
VVTU5492_at	GSVIVP00019793001	Q6A332 Always early protein 3 related cluster	-2.8
VVTU14092_s_at	GSVIVP00002889001	Q9LM88 F2D10.15 related cluster	-2.8
VVTU3336_s_at	TC66870	Q0ZJ30 RNA polymerase beta subunit related cluster	-2.8
VVTU22442_at	GSVIVP00032257001	Q4ADY3 ACAULIS5 protein related cluster	-2.8
VVTU734_at	GSVIVP00023945001	Q0WNY2 Hypothetical protein At1g06900 related cluster	-2.8
VVTU13248_at	TC60412	Q9LXK1 Hypothetical protein F3C22_30 related cluster	-2.8
VVTU8394_at	DV219303	Q94JV9 AT4g15140 dl3615c related cluster	-2.8
VVTU24771_at	GSVIVP00004577001	Q9FGD5 Emb|CAB16785.1 related cluster	-2.8
VVTU608_at	GSVIVP00038141001	Q6Z553 Hypothetical protein OSJNBa0007M04.36 related cluster	-2.8
VVTU2505_at	GSVIVP00019179001	O49741 GATA transcription factor 2 related cluster	-2.8
VVTU27242_at	GSVIVP00020384001	Q1S897 helicase, C-terminal related cluster	-2.8
VVTU34773_at	GSVIVP00021374001	Q2VWB7 Prf interactor 30137 related cluster	-2.9
VVTU2560_at	GSVIVP00018366001	Q5N9T4 Hypothetical protein P0414E03.11 related cluster	-2.9
VVTU8769_at	GSVIVP00001076001	O64647 Putative PCF2-like DNA bindinG protein related cluster	-2.9
VVTU22_at	GSVIVP00028373001	Q7XPZ2 OSJNBa0004N05.16 protein related cluster	-2.9
VVTU24220_at	DT020611	UPI00000A079C Cluster related to UPI00000A079C; P0458E05.30	-2.9
VVTU14848_at	CF605077	Q9SA83 T5I8.13 related cluster	-2.9
VVTU4290_at	GSVIVP00035401001	Q9LTX5 Similarity to alpha beta hydrolase related cluster	-2.9
VVTU20857_at	GSVIVP00027435001	Q1SGR1 Kinesin, motor region; prefoldin related cluster	-2.9
VVTU3516_at	GSVIVP00029273001	Q1I1D7 Cytochrome P450 related cluster	-2.9
VVTU9193_at	TC70900	Q60D21 Putative kinase interActing protein, identical related cluster	-2.9
VVTU26538_at	GSVIVP00033204001	Q10MF7 Expressed protein related cluster	-2.9
VVTU529_at	GSVIVP00034902001	Q1SQA9 Hypothetical protein related cluster	-2.9
VVTU21509_s_at	GSVIVP00029370001	Q9SS17 40S ribosomal protein S24-1 related cluster	-2.9
VVTU16618_s_at	GSVIVP00024375001	Q43724 Histone 2B related cluster	-2.9
VVTU14485_at	GSVIVP00034108001	Q9SLL2 F20D21.2 protein related cluster	-2.9
VVTU37641_at	TC61767	Q1S2B3 Hypothetical protein related cluster	-2.9
VVTU39765_at	GSVIVP00016095001	Q8S3S1 Putative Kinesin light chain gene related cluster	-2.9
VVTU38163_at	GSVIVP00001827001	Q9SK99 Similar to LeOPT1 [Lycopersicon esculentum] related cluster	-2.9
VVTU7974_at	GSVIVP00025255001	O49855 Acid phosphatase related cluster	-2.9
VVTU10597_at	GSVIVP00028929001	Q9LFX1 T7N9.27 related cluster	-3.0
VVTU483_at	GSVIVP00001802001	Q0DRV3 Os03g0352200 protein related cluster	-3.0
VVTU37709_at	GSVIVP00036929001	O81059 Putative calmodulin related cluster	-3.0
VVTU6319_at	GSVIVP00007311001	Q9SXE5 T3P18.8 related cluster	-3.0
VVTU25813_at	GSVIVP00024124001	Q8LF75 Putative receptor ser thr protein kinase related cluster	-3.0
VVTU15156_at	GSVIVP00005165001	Q1RVD3 AMP-binding enzyme, putative related cluster	-3.0
VVTU9641_at	TC56018	Q8VY60 Hypothetical protein At1g15260 related cluster	-3.0
VVTU5708_at	GSVIVP00038610001	Q1T2M9 PIK-related kinase, FAT; PIK-related kinase, FATC; peptidase M, neutral zinc metallopeptidases, zinc-binding site; FKBP12-rapamycin- associated protein, FKBP12-rapamycin-binding related cluster	-3.0
VVTU1212_at	GSVIVP00015855001	Q9LPJ0 F6N18.12 related cluster	-3.0
VVTU24911_at	GSVIVP00024165001	Q5YD56 Calcium calmodulin-regulated receptor-like kinase related cluster	-3.0
VVTU7328_at	GSVIVP00010064001	O23689 Hypothetical protein T19D16.22 related cluster	-3.0
VVTU13996_at	GSVIVP00019181001	Q1RY91 CCR4-Not complex component, Not1 related cluster	-3.0
VVTU4292_at	GSVIVP00030459001	Q9AWU8 P0044F08.14 protein related cluster	-3.0

Probe set	Unique Gene ID	Annotation	Fold-change
VVTU6065_at	GSVIVP00038890001	Q9M033 Hypothetical protein T10O8_110 related cluster	-3.0
VVTU6653_at	GSVIVP00035364001	Q2QW40 ATPase, AAA family protein, expressed related cluster	-3.0
VVTU4625_at	TC52429	Q41255 arabinogalactan-protein related cluster	-3.0
VVTU1134_at	GSVIVP00038524001	P93205 SBT2 protein related cluster	-3.1
VVTU17310_at	GSVIVP00038854001	Q6DBG3 At3g08955 related cluster	-3.1
VVTU27945_at	GSVIVP00002558001	Q9LN01 T6D22.15 related cluster	-3.1
VVTU12711_at	GSVIVP00028093001	Q1SDB1 Tetratricopeptide repeat, putative related cluster	-3.1
VVTU24186_at	GSVIVP00026093001	Q7XIJ3 Putative phototropic response protein family related cluster	-3.1
VVTU6230_at	GSVIVP00032373001	O80763 T13D8.29 protein related cluster	-3.1
VVTU32611_at	CB349580	Q8GYX1 Hypothetical protein At4g37460 F6G17_110 related cluster	-3.1
VVTU10817_at	GSVIVP00025876001	Q9M306 Hypothetical protein T21J18_30 related cluster	-3.1
VVTU9886_at	TC60042	Q04716 Mitochondrial ribosomal protein S3 related cluster	-3.2
VVTU5912_at	GSVIVP00017727001	Q9M9Q2 T15D22.8 related cluster	-3.2
VVTU14518_at	GSVIVP00001082001	Q1L5X6 RNA polymerase IV largest subunit related cluster	-3.2
VVTU32695_s_at	GSVIVP00036771001	Q8LBL4 Putative Thaumatin-like protein related cluster	-3.2
VVTU15362_at	TC62063	Q1SF84 Histone-fold related cluster	-3.2
VVTU37434_x_at	VVTU37434_x_at	Q9M4H3 Putative Metallothionein-like protein related cluster	-3.2
VVTU2258_at	GSVIVP00021072001	Q1SSK0 Methyladenine glycosylase related cluster	-3.2
VVTU2764_s_at	GSVIVP00034436001	Q9C6D2 Hypothetical protein F10F5.1 related cluster	-3.2
VVTU12188_s_at	GSVIVP00018131001	Q27U75 Pectate lyase related cluster	-3.3
VVTU9251_at	GSVIVP00021307001	Q9LD90 H ACA ribonucleoprotein complex subunit 4 related cluster	-3.3
VVTU7655_at	GSVIVP00027269001	Q9FXL4 Elicitor inducible beta-1,3-Glucanase NtEIG-E76 related cluster	-3.3
VVTU10786_at	GSVIVP00014064001	Q2QB51 Fused related cluster	-3.3
VVTU12819_at	GSVIVP00025490001	Q76DY1 AG-motif binding protein-3 related cluster	-3.3
VVTU9804_at	GSVIVP00021238001	Q259S0 H0403D02.14 protein related cluster	-3.3
VVTU7213_at	GSVIVP00007725001	Q948Z4 Snakin-1 related cluster	-3.3
VVTU9086_at	GSVIVP00027433001	Q9XF70 Thioredoxin h related cluster	-3.3
VVTU20577_at	GSVIVP00016840001	Q1RVL3 E-class P450, group I related cluster	-3.3
VVTU38361_at	GSVIVP00017583001	Q1RSF8 Mis12 related cluster	-3.4
VVTU20271_at	GSVIVP00016362001	Q3E989 Protein At5g20860 related cluster	-3.4
VVTU8981_at	CA813901	Q04218 TNP1 related cluster	-3.4
VVTU5192_at	GSVIVP00016401001	Q1SGW0 Hypothetical protein related cluster	-3.4
VVTU16916_s_at	TC70718	Q0W9E8 Putative stress-induced protein related cluster	-3.4
VVTU28517_at	GSVIVP00023952001	Q0JI54 Os01g0823500 protein related cluster	-3.4
VVTU9698_at	GSVIVP00034194001	Q8L741 AT3g13690 MMM17_12 related cluster	-3.4
VVTU25681_at	GSVIVP00016088001	Q1T4S0 PIK-related kinase, FATC related cluster	-3.4
VVTU14078_at	GSVIVP00028307001	O82002 Putative cullin protein related cluster	-3.4
VVTU5624_at	GSVIVP00022735001	P25470 Histone H2A.1 related cluster	-3.4
VVTU20963_at	GSVIVP00030533001	Q9AXH3 Phosphoethanolamine N-methyltransferase related cluster	-3.4
VVTU22448_at	GSVIVP00030128001	Q948W7 Zinc-bindinG protein related cluster	-3.4
VVTU31854_at	CB344450	Q19KB4 PSI P700 apoprotein A2 related cluster	-3.5
VVTU2267_at	GSVIVP00031369001	Q9FKM3 Similarity to AAA-type ATPase related cluster	-3.5
VVTU1856_at	GSVIVP00019383001	Q9SVA9 Cytochrome P450-like protein related cluster	-3.5
VVTU36710_at	GSVIVP00027351001	Q9FFU3 genomic DNA, chromosome 5, P1 clone:MBG8 related cluster	-3.5
VVTU13334_s_at	GSVIVP00027149001	Q2MCJ5 Xylan 1,4-beta-xylosidase related cluster	-3.6
VVTU13251_at	GSVIVP00035580001	Q5M9T5 Hypothetical protein orf116b related cluster	-3.6
VVTU4627_at	GSVIVP00014628001	Q9STG6 DUTP pyrophosphatase-like protein related cluster	-3.6
VVTU18442_at	GSVIVP00005112001	Q9CAZ7 strictosidine synthase-like protein related cluster	-3.6
VVTU40323_at	GSVIVP00017134001	Q9SUD1 Hypothetical protein T13J8.190 related cluster	-3.6
VVTU8520_at	GSVIVP00019293001	Q9SIT6 White-brown complex homoloG protein 5 related cluster	-3.6
VVTU15303_at	GSVIVP00035896001	Q8L7S5 AT4g18560 F28J12_220 related cluster	-3.6
VVTU11499_at	GSVIVP00019642001	Q1SRF6 GRAS transcription factor related cluster	-3.6
VVTU28244_at	GSVIVP00028662001	Q6T284 Predicted protein related cluster	-3.6
VVTU23225_at	GSVIVP00035579001	Q5MA17 Hypothetical protein orf315 related cluster	-3.6
VVTU22428_at	GSVIVP00017797001	Q1SYZ0 homeodomain-related related cluster	-3.6
VVTU13250_s_at	GSVIVP00022144001	AF056622 Vitis vinifera putative Cu Zn superoxide dismutase precursor, mRNA, nuclear gene encoding chloroplast protein, complete cds.	-3.6
VVTU6696_at	GSVIVP00025873001	Q1SJL6 Hypothetical protein related cluster	-3.7
VVTU11049_at	GSVIVP00021959001	UPI00000ABE75 Cluster related to UPI00000ABE75; unknown protein	-3.7
VVTU15818_at	GSVIVP00028054001	Q1RV39 Calponin-like Actin-binding; Kinesin, motor region related cluster	-3.7
VVTU3144_at	GSVIVP00019678001	Q9LNG3 F21D18.18 related cluster	-3.7
VVTU5224_at	GSVIVP00006512001	AB240539 Vitis vinifera pVvSPMS mRNA for spermine synthase, partial cds.	-3.8
VVTU10506_at	GSVIVP00022862001	Q05929 EDGP precursor related cluster	-3.8
VVTU39711_at	GSVIVP00027802001	Q9MAH1 F12M16.20 related cluster	-3.8
VVTU12815_at	GSVIVP00029240001	Q93Z79 AT5g24910 F6A4_120 related cluster	-3.9
VVTU26115_at	GSVIVP00035798001	Q9C6G3 receptor-like serine threonine kinase (RFK1), putative related cluster	-3.9
VVTU32106_at	CB349113	Q10BS5 elongation factor P family protein, expressed related cluster	-3.9
VVTU8383_at	GSVIVP00000349001	Q9XI33 F9L1.31 protein related cluster	-3.9
VVTU27256_at	CF405610	Q3T4H1 9,10[9 ,10]carotenoid cleavage dioxygenase related cluster	-3.9
VVTU36136_at	GSVIVP00032732001	Q0IZQ1 Os09g0556700 protein related cluster	-3.9
VVTU8833_at	DT006575	Q7XDP3 Expressed protein related cluster	-4.0
VVTU1706_at	GSVIVP00004380001	Q41495 STS14 protein precursor related cluster	-4.0
VVTU9703_at	GSVIVP00030737001	Q9LIP5 Cytochrome P450 71B35 related cluster	-4.0
VVTU25909_at	GSVIVP00013289001	Q2HTG5 Hypothetical protein related cluster	-4.0
VVTU17592_at	GSVIVP00016349001	Q3HTV1 Ternary complex factor MIP1-like related cluster	-4.0
VVTU9710_at	GSVIVP00022519001	O80535 F14J9.18 protein related cluster	-4.0

Probe set	Unique Gene ID	Annotation	Fold-change
VVTU2867_at	GSVIVP00025741001	Q9LDD1 Arabidopsis thaliana genomic DNA, chromosome 3, P1 clone: MGH6 related cluster	-4.0
VVTU24341_at	GSVIVP00032172001	P48731 Homeobox protein ATH1 related cluster	-4.1
VVTU25603_s_at	GSVIVP00031336001	Q9SUM5 Hypothetical protein F9N11.30 related cluster	-4.2
VVTU14818_at	CK138186	Q6L4Y9 Hypothetical protein OSJNBb0092E21.5 related cluster	-4.2
VVTU24716_s_at	GSVIVP00034648001	Q1SZE7 Hypothetical protein related cluster	-4.3
VVTU23904_at	DT031319	UPI00000A11FF Cluster related to UPI00000A11FF; putative gag pol Polyprotein	-4.4
VVTU8940_at	TC64347	Q9MA43 Histone-lysine N-methyltransferase ATX2 related cluster	-4.4
VVTU13307_at	GSVIVP00036679001	Q8GU24 Orcinol O-methyltransferase related cluster	-4.4
VVTU482_at	VVTU482_at	Q9S839 F1C9.26 protein related cluster	-4.5
VVTU3891_at	GSVIVP00002293001	Q9SRD1 Putative translation Initiation factor IF-2; 74568-78972 related cluster	-4.6
VVTU39779_at	GSVIVP00022469001	UPI000034F21F Cluster related to UPI000034F21F; EMB2247; ATP binding tRNA ligase valine-tRNA ligase	-4.6
VVTU12374_at	GSVIVP00024085001	Q8H1X7 Microsomal omega-3 fatty acid desaturase related cluster	-4.7
VVTU20234_at	GSVIVP00033131001	Q1T0F2 PAPA-1-like conserved region; Zinc finger, HIT-type related cluster	-4.9
VVTU24876_at	GSVIVP00030281001	Q9LPR2 F15H18.5 related cluster	-4.9
VVTU15632_at	GSVIVP00028855001	Q5VRG7 Putative RING finger 1 related cluster	-5.0
VVTU8676_at	GSVIVP00006779001	Q75VK7 CC-NB-LRR protein related cluster	-5.0
VVTU16070_at	GSVIVP00003139001	Q4V3D9 At3g57030 related cluster	-5.1
VVTU4071_at	GSVIVP00030552001	Q9LQU1 F10B6.30 related cluster	-5.1
VVTU25265_at	CX016363	Q09MD9 ribosomal protein S3 related cluster	-5.1
VVTU24285_at	GSVIVP00010655001	Q9T068 Hypothetical protein AT4g37810 related cluster	-5.2
VVTU38338_x_at	GSVIVP00035995001	Q9SW54 Hypothetical protein T11I11.40 related cluster	-5.2
VVTU11160_at	GSVIVP00022793001	Q9MA51 F22F7.18 protein related cluster	-5.3
VVTU9509_at	TC65277	Q8LPF0 At1g73960 F2P9_17 related cluster	-5.3
VVTU22814_at	GSVIVP00005721001	Q9LQ30 F14M2.10 protein related cluster	-5.3
VVTU8163_at	CB350096	Q8LT03 Leaf thionin Asthi1 related cluster	-5.4
VVTU34022_at	GSVIVP00002231001	Q9M837 T27C4.15 protein related cluster	-5.5
VVTU8716_at	GSVIVP00036789001	Q0IYQ9 Os10g0176300 protein related cluster	-5.5
VVTU3771_at	GSVIVP00027772001	Q0GPI0 BZIP transcription factor bZIP50 related cluster	-5.5
VVTU164_at	GSVIVP00015103001	Q6Z2W0 Chromosome-associated Kinesin-like related cluster	-5.5
VVTU19090_s_at	GSVIVP00035985001	Q94B76 Putative auxin-regulated protein related cluster	-5.6
VVTU2068_at	GSVIVP00022959001	Q0W9E8 Putative stress-induced protein related cluster	-5.7
VVTU5431_at	GSVIVP00025322001	Q2HRH3 Gibberellin regulated protein related cluster	-5.8
VVTU30293_at	GSVIVP00038386001	Q2MCJ5 Xylan 1,4-beta-xylosidase related cluster	-5.9
VVTU8304_at	GSVIVP00026695001	Q6F5D8 Putative ammonium transporter related cluster	-5.9
VVTU22691_s_at	GSVIVP00016335001	Q49RB3 Gip1-like protein related cluster	-6.8
VVTU15369_at	GSVIVP00035100001	Q9LU93 Mitotic spindle checkpoint protein MAD2 related cluster	-6.9
VVTU28233_at	GSVIVP00033422001	UPI0000163166 Cluster related to UPI0000163166; unknown protein	-7.8
VVTU459_x_at	VVTU459_x_at	Q53IB4 Hypothetical protein related cluster	-7.9
VVTU39410_s_at	GSVIVP00030739001	Q944N6 Cytochrome P450 related cluster	-8.2
VVTU1199_at	GSVIVP00002593001	Q6Z1Z2 Putative HMG type nucleosome chromatin assembly factor D related cluster	-8.3
VVTU10809_at	GSVIVP00016157001	Q1SAT8 Haem peroxidase, plant fungal bacterial related cluster	-9.5
