# Supplementary material for: Transcriptome changes in grapevine (Vitis vinifera L.) cv. Malbec leaves induced by ultraviolet-B radiation
Source: BMC Plant Biol. 2010 Oct 20;10:224. doi: 10.1186/1471-2229-10-224 (PMC3017828; doi:10.1186/1471-2229-10-224)
Supplement: Additional file 2 — Full list of genes differentially expressed under low UV-B radiation. PDFgenes file showing a complete list of the genes differentially expressed in the low UV-B treatment including Probe-set ID, Unique grapevine gene ID, Annotation and fold-change. [file 1471-2229-10-224-S2.PDF]

Low UVB treatment

Probe set	Unique Gene ID	Annotation	Fold-change
VVTU9622_at	TC64430	Q0WYB9 Hypothetical protein related cluster	33.5
VVTU16103_at	GSVIVP00015343001	Q2PHJ7 flavonol synthase related cluster	23.9
VVTU34551_x_at	GSVIVP00031875001	AY670148 Vitis vinifera clone 357851_M2 stilbene synthase mRNA, partial cds.	20.8
VVTU22923_at	GSVIVP00018625001	Q0IS70 Os11g0552000 protein related cluster	20.1
VVTU5874_s_at	CF204867	Q8LC91 Hypothetical protein related cluster	16.7
VVTU14066_at	GSVIVP00022375001	Q6QB11 Little protein 1 related cluster	16.2
VVTU2839_at	GSVIVP00001453001	Q4AEC3 Cys2-His2 type Zinc finger protein related cluster	15.9
VVTU12705_s_at	GSVIVP00024561001	Q6L3H6 Adenylatekinase isoenzyme 6 , putative related cluster	15.5
VVTU3104_at	GSVIVP00009608001	O80432 Mitochondrial small Heat shock protein related cluster	14.1
VVTU8172_at	GSVIVP00009541001	O80337 ethylene-responsive transcription factor 1A related cluster	12.5
VVTU11765_at	GSVIVP00004049001	AY670089 Vitis vinifera clone 325905_S3 stilbene synthase mRNA, partial cds.	12.0
VVTU13949_at	GSVIVP00000688001	Q9ZVC3 Putative Embryo-abundant protein related cluster	11.4
VVTU17051_at	TC69703	Q6H515 Hypothetical protein OSJNBa0073A21.9 related cluster	10.5
VVTU12825_at	GSVIVP00032689001	Q6RZW9 Putative WRKY4 transcription factor related cluster	10.4
VVTU5290_at	GSVIVP00017370001	Q1SXN3 Harpin-induced 1 related cluster	9.9
VVTU3375_at	GSVIVP00014947001	Q9XEU0 Zinc-finger protein 1 related cluster	9.8
VVTU25456_at	GSVIVP00020045001	Q2R227 Zinc finger, C3HC4 type family protein, expressed related cluster	9.4
VVTU9041_at	TC63592	Q75KH8 Hypothetical protein OJ1057_G07.4 related cluster	9.4
VVTU7830_at	GSVIVP00029583001	Q9LJB7 Emb|CAA19725.1 related cluster	8.8
VVTU2102_at	GSVIVP00002706001	O64594 F17O7.4 related cluster	8.5
VVTU1998_at	GSVIVP00033242001	Q1RW45 Hypothetical protein related cluster	8.4
VVTU1866_at	GSVIVP00006872001	Q0JP37 Os01g0246700 protein related cluster	8.3
VVTU8153_at	GSVIVP00017555001	Q9C9B0 Putative Glucosyltransferase; 88035-86003 related cluster	8.2
VVTU17078_at	TC62881	Q1RT15 Hypothetical protein related cluster	8.1
VVTU12582_at	GSVIVP00001179001	Q9C893 Hypothetical protein F7A10.19 related cluster	7.6
VVTU9258_at	TC60871	Q9FKM0 Arabidopsis thaliana genomic DNA, chromosome 5, P1 clone:MUA2 related cluster	7.6
VVTU11030_at	GSVIVP00023009001	Q5ZEM1 Putative lectin 2 related cluster	7.6
VVTU22577_at	GSVIVP00014854001	Q1T4E2 Transferase related cluster	7.6
VVTU34913_at	AJ862932	AY670143 Vitis vinifera clone 357844_R1 stilbene synthase mRNA, partial cds.	7.5
VVTU35597_at	VVTU35597_at	Q0WYB9 Hypothetical protein related cluster	7.5
VVTU12359_at	VVTU12359_at	Q1T1S6 Curculin-like (Mannose-binding) lectin; Apple-like related cluster	7.4
VVTU33878_s_at	GSVIVP00016808001	Q8L6S5 IAA16 protein related cluster	7.3
VVTU13085_at	GSVIVP00033125001	VVU73709 Vitis vinifera beta-1,3-Glucanase mRNA, partial cds.	7.3
VVTU23243_at	GSVIVP00036549001	Q1SZH7 Hypothetical protein related cluster	7.3
VVTU33818_at	GSVIVP00009607001	O80432 Mitochondrial small Heat shock protein related cluster	7.2
VVTU1137_at	GSVIVP00033036001	Q5ICP0 RING-H2 subgroup RHE protein related cluster	7.2
VVTU9349_at	TC67083	Q337C0 IMP dehydrogenase GMP reductase domain containing protein, expressed related cluster	7.1
VVTU876_at	GSVIVP00029253001	Q9FQ21 Putative Hs1pro-1-like receptor related cluster	7.0
VVTU709_at	GSVIVP00036931001	Q52QR5 NAC domain protein NAC1 related cluster	7.0
VVTU37055_at	GSVIVP00002261001	Q1SHY7 U box related cluster	6.9
VVTU14440_at	GSVIVP00023927001	O04682 Pathogenesis-related genes transcriptional activator PTI6 related cluster	6.9
VVTU6191_at	GSVIVP00014004001	Q9C6E4 Phosphate-induced (Phi-1) protein, putative related cluster	6.8
VVTU2850_at	GSVIVP00019287001	Q6RH27 NAC domain protein related cluster	6.8
VVTU21888_at	GSVIVP00011639001	Q9ATW1 Cinnamyl alcohol dehydrogenase related cluster	6.7
VVTU26707_at	TC71212	Q9LKG8 TIP related cluster	6.6
VVTU24126_x_at	GSVIVP00017943001	Q5CDC4 LacOPZ-alpha peptide from pUC9 related cluster	6.4
VVTU2514_at	GSVIVP00029304001	Q0GIK4 Cys-3-His Zinc finger protein related cluster	6.4
VVTU2377_at	GSVIVP00009741001	Q2HTZ5 At1g61340 related cluster	6.3
VVTU1581_s_at	TC53821	Q9LW49 ethylene-responsive transcription factor 4 related cluster	6.3
VVTU1528_at	GSVIVP00022724001	Q1I1D6 Hypoxia-responsive family protein related cluster	6.2
VVTU40803_s_at	GSVIVP00033165001	Q2PJR6 WRKY54 related cluster	6.1
VVTU28485_at	GSVIVP00014244001	Q6RZW8 Putative ethylene response factor 4 related cluster	6.1
VVTU4467_s_at	TC58280	Q9FWQ7 F17F16.6 protein related cluster	6.0
VVTU15353_at	GSVIVP00017017001	Q9M4U0 Cinnamate 4-hydroxylase CYP73 related cluster	6.0
VVTU10895_at	GSVIVP00020809001	Q9XH76 Putative Zinc finger protein related cluster	6.0
VVTU3091_at	GSVIVP00003524001	Q9ZSD5 Syntaxin-related protein Nt-syr1 related cluster	6.0
VVTU6420_at	GSVIVP00038540001	Q941F6 Leucine-rich repeat receptor-like kinase F21M12.36 related cluster	6.0
VVTU15513_at	GSVIVP00037696001	Q0WWD0 Hypothetical protein At4g25640 related cluster	5.9
VVTU39708_s_at	GSVIVP00031477001	Q6VAB2 UDP-glycosyltransferase 71E1 related cluster	5.7
VVTU16659_at	GSVIVP00028243001	Q1SEN8 Cyclic peptide transporter related cluster	5.7
VVTU36562_at	GSVIVP00029172001	Q49L63 GEX1 related cluster	5.7
VVTU39801_at	GSVIVP00006230001	Q9FZ93 F3H9.15 protein related cluster	5.7
VVTU38576_at	GSVIVP00018787001	Q0JEB3 Os04g0303100 protein related cluster	5.7
VVTU6595_s_at	GSVIVP00015077001	Q9FME9 Emb|CAB89373.1 related cluster	5.7
VVTU34740_s_at	GSVIVP00029302001	Q9XEE6 Hypothetical Cys-3-His Zinc finger protein related cluster	5.6
VVTU1920_at	GSVIVP00020018001	AY156051 Vitis vinifera putative alanine acetyl transferase (AAT) mRNA, partial cds.	5.6
VVTU875_s_at	TC60860	Q259M7 H0723C07.4 protein related cluster	5.6
VVTU15680_at	GSVIVP00020726001	Q8LDZ7 Cinnamoyl CoA reductase-like protein related cluster	5.5
VVTU34592_at	GSVIVP00022733001	Q6F2U9 Lysyl-tRNA synthetase related cluster	5.5
VVTU13009_s_at	GSVIVP00027001001	Q6R7N3 Putative WRKY transcription factor 30 related cluster	5.5
VVTU21823_at	GSVIVP00016657001	Q1SND5 Protein kinase related cluster	5.4
VVTU4088_s_at	TC70295	Q8GYA5 Hypothetical protein related cluster	5.3
VVTU12853_at	GSVIVP00033158001	Q9SWP6 Hypersensitive reaction associated Ca2+-bindinG protein related cluster	5.3
VVTU1632_at	GSVIVP00030524001	Q9AXU0 Major latex-like protein related cluster	5.2

Probe set	Unique Gene ID	Annotation	Fold-change
VVTU3968_s_at	TC57893	Q9FLD2 Arabidopsis thaliana genomic DNA, chromosome 5, TAC clone:K18I23 related cluster	5.2
VVTU15537_at	GSVIVP00000686001	Q9ZVC3 Putative Embryo-abundant protein related cluster	5.2
VVTU21267_s_at	GSVIVP00036623001	Q1SWF3 Hypothetical protein related cluster	5.2
VVTU1524_at	TC64418	Q1SBD7 Hypothetical protein related cluster	5.1
VVTU4057_at	GSVIVP00007673001	Q2PAJ1 Putative Laccase related cluster	5.1
VVTU12900_at	GSVIVP00031553001	Q9LU67 Phosphatidylserine decarboxylase related cluster	5.1
VVTU11112_at	VVTU11112_at	Q8LGJ9 Hypothetical protein related cluster	5.1
VVTU7250_s_at	GSVIVP00020826001	Q1S520 AAA ATPase related cluster	5.1
VVTU12806_at	GSVIVP00011451001	Q84KA9 RING C3HC4 PHD Zinc finger-like protein related cluster	5.1
VVTU12901_at	GSVIVP00010117001	DQ366302 Vitis vinifera resveratrol synthase (STS2) mRNA, complete cds.	5.0
VVTU3450_at	GSVIVP00033193001	Q9XEJ7 GalActinol synthase related cluster	5.0
VVTU15081_at	GSVIVP00036272001	Q8LEZ7 Hypothetical protein related cluster	5.0
VVTU33588_at	GSVIVP00007928001	Q8VYV9 Putative chloroplast nucleoid DNA-bindinG protein related cluster	4.9
VVTU16405_at	CD801282	UPI000034F181 Cluster related to UPI000034F181; aspartic-type endopeptidase pepsin A	4.9
VVTU2511_at	GSVIVP00013642001	Q6ZD72 Hypothetical protein P0450B04.17 related cluster	4.9
VVTU15006_at	GSVIVP00014758001	Q2V454 Protein At2g26530 related cluster	4.8
VVTU2167_s_at	GSVIVP00015811001	Q9ZVJ5 Expressed protein related cluster	4.8
VVTU13677_x_at	GSVIVP00036892001	AY634281 Vitis viniferadehydrin mRNA, complete cds.	4.7
VVTU7676_at	GSVIVP00006413001	Q402G4 Hypothetical protein GmTDF-5 related cluster	4.7
VVTU22090_at	GSVIVP00021134001	Q9LHJ2 UDP-glucose Glucosyltransferase-like protein related cluster	4.7
VVTU4884_at	GSVIVP00002825001	Q2YHM9 Caffeoyl-CoA O-methyltransferase related cluster	4.7
VVTU3717_at	GSVIVP00027568001	Q9LSQ8 Arabidopsis thaliana genomic DNA, chromosome 5, BAC clone:F24B18 related cluster	4.6
VVTU17659_at	GSVIVP00002892001	P26585 HMG1 2-like protein related cluster	4.5
VVTU12958_s_at	GSVIVP00005633001	Q1SP35 Hypothetical protein related cluster	4.5
VVTU2346_at	GSVIVP00000517001	Q1SFJ9 Leucine zipper, Homeobox-associated; homeodomain-related related cluster	4.5
VVTU19113_s_at	TC62328	Q7XYW1 seed specific protein Bn15D18B related cluster	4.5
VVTU1951_at	GSVIVP00025595001	Q7XI29 Hypothetical protein P0506C07.1 related cluster	4.5
VVTU14989_at	CA816537	O22086 ZPT2-14 related cluster	4.4
VVTU1670_at	GSVIVP00023211001	O64963 Phenylalanine ammonia-lyase 1 related cluster	4.4
VVTU12952_x_at	GSVIVP00006725001	P37707 B2 protein related cluster	4.4
VVTU13727_at	GSVIVP00016280001	Q9CA77 Putative receptor protein kinase; 10992-14231 related cluster	4.3
VVTU35417_at	GSVIVP00030754001	Q9FXE3 F12A21.6 related cluster	4.3
VVTU13088_at	GSVIVP00019566001	AY953543 Vitis vinifera sucrose responsive element bindinG protein (SREBP) mRNA, complete cds.	4.3
VVTU6000_at	GSVIVP00001184001	Q3HNF4 ABA 8 -hydroxylase CYP707A1 related cluster	4.2
VVTU17706_at	GSVIVP00037207001	Q5MJW2 Avr9 Cf-9 rapidly elicited protein 102 related cluster	4.2
VVTU6040_x_at	GSVIVP00013930001	X75967 V.vinifera PAL mRNA for phenylalanine ammonia lyase.	4.2
VVTU2527_at	GSVIVP00026904001	O82357 3-methyl-2-oxobutanoate hydroxy-methyl-transferase related cluster	4.2
VVTU10469_at	VVTU10469_at	Q3EA29 Protein At4g14746 related cluster	4.2
VVTU31924_at	CB350300	Q9LNC5 F9P14.8 protein related cluster	4.2
VVTU13534_s_at	GSVIVP00023496001	AY971515 Vitis vinifera glutathione S-transferase (GST4) mRNA, complete cds.	4.1
VVTU37918_at	GSVIVP00010608001	Q1RXS0 RNA-directed DNA polymerase(Reverse transcriptase); Integrase, catalytic region related cluster	4.0
VVTU35637_at	GSVIVP00018316001	Q8S9A7 Glucosyltransferase-2 related cluster	4.0
VVTU32772_at	CB345571	Q7XS60 OSJNBa0019G23.7 protein related cluster	4.0
VVTU12323_at	GSVIVP00017121001	Q944U3 S-adenosyl-L-methionine decarboxylase related cluster	4.0
VVTU22537_at	GSVIVP00007523001	P93392 S25-XP1 DNA bindinG protein related cluster	4.0
VVTU2810_at	GSVIVP00009601001	Q39079 chaperone protein DnaJ 13 related cluster	4.0
VVTU17421_s_at	GSVIVP00003172001	Q84V47 Alternative oxidase 1a related cluster	4.0
VVTU1752_at	GSVIVP00023625001	Q0PIN1 Gibberellic acid receptor related cluster	4.0
VVTU20253_x_at	TC65749	Q84TK8 Hypothetical protein related cluster	3.9
VVTU12860_at	GSVIVP00011638001	Q5I6D6 Sinapyl alcohol dehydrogenase-like protein related cluster	3.9
VVTU9420_at	GSVIVP00006193001	Q9LD82 Putative heme bindinG protein 2 related cluster	3.9
VVTU16451_at	GSVIVP00002806001	Q8LEW4 Hypothetical protein related cluster	3.9
VVTU31624_at	CB345765	Q1S3A8 Hypothetical protein related cluster	3.9
VVTU12881_at	GSVIVP00033758001	Q1T5K7 Protein kinase; NAF related cluster	3.9
VVTU14413_at	GSVIVP00033009001	Q1M0P1 UDP-glucuronic acid decarboxylase 2 related cluster	3.8
VVTU13180_s_at	GSVIVP00009468001	O04287 Peptidyl-prolyl isomerase FKBP12 related cluster	3.8
VVTU13621_at	GSVIVP00014352001	O49284 F22K20.8 protein related cluster	3.8
VVTU27760_at	GSVIVP00007714001	Q40489 Cyclin A-like protein related cluster	3.8
VVTU38545_at	GSVIVP00017721001	Q8VYG0 Hypothetical protein At5g38210 related cluster	3.7
VVTU1828_at	GSVIVP00025998001	Q5XEV3 At4g03410 related cluster	3.7
VVTU4133_s_at	GSVIVP00019488001	Q9SXK9 Heat shock factor related cluster	3.7
VVTU21_at	GSVIVP00025478001	Q9LVC3 Similarity to RNA-bindinG protein related cluster	3.7
VVTU13708_at	GSVIVP00002550001	P51819 Heat shock protein 83 related cluster	3.7
VVTU8356_at	GSVIVP00028087001	Q1SDA9 Lipase, class 3 related cluster	3.7
VVTU25737_at	GSVIVP00015435001	Q67UZ3 Chloroplast thylakoidal processing peptidase-like protein related cluster	3.7
VVTU2469_at	GSVIVP00029153001	Q9AR56 Putative membrane protein related cluster	3.7
VVTU37515_at	GSVIVP00036684001	Q1SR68 Zinc finger, RING-type related cluster	3.7
VVTU16909_at	GSVIVP00036634001	Q155L4 Cysteine protease related cluster	3.7
VVTU12218_at	GSVIVP00014419001	Q2QCX5 Gibberellin 20-oxidase 1 related cluster	3.6
VVTU8151_at	GSVIVP00018450001	O22282 Expressed protein related cluster	3.6
VVTU32112_x_at	CB349093	Q42428 Chitinase Ib related cluster	3.6
VVTU2631_at	GSVIVP00014887001	Q1XAN1 Sucrose responsive element bindinG protein related cluster	3.6

Probe set	Unique Gene ID	Annotation	Fold-change
VVTU38380_at	TC69215	Q1SCY9 Integrase, catalytic region; Zinc finger, CCHC-type related cluster	3.6
VVTU9489_at	GSVIVP00032680001	Q3E959 Protein At5g25320 related cluster	3.6
VVTU3517_at	GSVIVP00015738001	Q1T6L3 Ribosome-binding factor A related cluster	3.6
VVTU14850_at	GSVIVP00018698001	Q9SLC3 RING-H2 finger protein ATL2L related cluster	3.6
VVTU24019_at	GSVIVP00032350001	Q8GZN1 Pollen-specific calmodulin-bindinG protein related cluster	3.6
VVTU34699_at	TC65948	Q4YQV6 Hypothetical protein related cluster	3.5
VVTU34474_at	GSVIVP00009744001	Q9S9T5 T32N4.4 protein related cluster	3.5
VVTU7639_s_at	GSVIVP00037700001	Q6V7U8 Putative anthocyanin permease related cluster	3.5
VVTU34812_s_at	GSVIVP00031317001	Q9SXP4 DNA-bindinG protein NtWRKY3 related cluster	3.5
VVTU21891_s_at	DV218500	Q6IM91 DVL10 related cluster	3.5
VVTU12870_s_at	GSVIVP00024457001	Q0R4I2 Trichoderma-induced protein kinase related cluster	3.5
VVTU6973_at	GSVIVP00003030001	Q8GU24 Orcinol O-methyltransferase related cluster	3.5
VVTU35944_at	GSVIVP00037648001	Q6RZW9 Putative WRKY4 transcription factor related cluster	3.5
VVTU4107_at	GSVIVP00028179001	Q1SSW0 Protein phosphatase 2C related cluster	3.4
VVTU22069_at	GSVIVP00037998001	Q2PET4 Hypothetical protein related cluster	3.4
VVTU68_at	GSVIVP00038107001	Q0WW94 Kanadaptin-like protein related cluster	3.4
VVTU15954_s_at	GSVIVP00026185001	Q56TL1 lateelongated hypocotyl related cluster	3.4
VVTU17005_at	GSVIVP00014683001	Q1S566 GNS1 SUR4 membrane protein related cluster	3.4
VVTU1996_x_at	GSVIVP00026516001	Q8LA49 globulin-like protein related cluster	3.4
VVTU21860_at	GSVIVP00017875001	Q7Y036 MutT-like protein related cluster	3.4
VVTU28352_at	GSVIVP00024745001	Q9C523 dirigent protein, putative related cluster	3.3
VVTU31762_at	CB345042	P62577 Chloramphenicol acetyltransferase related cluster	3.3
VVTU15154_s_at	GSVIVP00022182001	Q7XE25 Heavy metal-associated domain containing protein, expressed related cluster	3.3
VVTU15394_s_at	GSVIVP00019630001	Q1SC00 Hypothetical protein related cluster	3.3
VVTU33319_at	GSVIVP00001069001	Q9T059 Hypothetical protein AT4g11910 related cluster	3.3
VVTU27967_at	GSVIVP00001320001	Q93X02 Putative ammonium transporter AMT2 related cluster	3.3
VVTU10306_at	GSVIVP00032404001	Q94LF5 Hypothetical protein related cluster	3.3
VVTU24692_at	GSVIVP00003403001	Q1EPJ3 DNA-binding WRKY domain-containinG protein related cluster	3.2
VVTU6002_s_at	TC64484	Q6NN03 At5g04080 related cluster	3.2
VVTU2500_at	GSVIVP00015755001	Q0VJB4 Putative zinc-bindinG protein related cluster	3.2
VVTU3038_at	GSVIVP00032812001	Q84NG9 2S albumin related cluster	3.2
VVTU13148_at	GSVIVP00018441001	Q9M2S4 Probable serine threonine-specific protein kinase related cluster	3.2
VVTU4436_s_at	GSVIVP00003042001	Q5QMP4 No apical meristem (NAM) protein-like related cluster	3.2
VVTU25318_s_at	GSVIVP00014931001	Q9M5B0 cytochrome b5 DIF-F related cluster	3.2
VVTU13081_at	GSVIVP00023994001	Q40090 SPF1 protein related cluster	3.2
VVTU27218_at	CF405875	Q9M3Y5 25.7 kDa protein related cluster	3.2
VVTU26934_at	GSVIVP00027084001	Q67UZ4 Putative fiber Annexin related cluster	3.2
VVTU33585_at	CB341021	Q17E94 Hypothetical protein related cluster	3.1
VVTU21498_at	GSVIVP00016086001	Q9ZUC4 F5O8.26 protein related cluster	3.1
VVTU35459_at	GSVIVP00020589001	Q9FM29 Dbj|BAA95711.1 related cluster	3.1
VVTU11587_at	GSVIVP00028163001	O49020 Myb-like DNA-binding domain protein related cluster	3.1
VVTU6036_at	GSVIVP00019956001	AY538261 Vitis vinifera hexose transporter (HT5) mRNA, complete cds.	3.1
VVTU9645_at	GSVIVP00033458001	Q7XQN1 OSJNBa0089K21.5 protein related cluster	3.1
VVTU2622_at	GSVIVP00015121001	Q2PEQ8 Putative mitochondrial dicarboxylatecarrier protein related cluster	3.1
VVTU5434_at	GSVIVP00021464001	UPI000034EE33 Cluster related to UPI000034EE33; carbohydrate transporter organic anion transporter sugar porter	3.1
VVTU15920_at	TC52642	Q9LRV7 Gb|AAF04880.1 related cluster	3.1
VVTU7764_at	GSVIVP00020272001	Q9FQE4 glutathione S-transferase GST 14 related cluster	3.1
VVTU2507_s_at	GSVIVP00025952001	Q5VLJ53-hydroxy-3-methylglutaryl oxidase related cluster	3.1
VVTU6665_s_at	GSVIVP00036772001	Q9LQT4 T4O12.3 related cluster	3.0
VVTU37933_at	GSVIVP00028930001	O04568 T7N9.26 related cluster	3.0
VVTU2436_at	GSVIVP00008328001	Q9SUU4 Hypothetical protein F8B4.180 related cluster	3.0
VVTU3303_s_at	GSVIVP00035835001	Q3SAJ9 WRKY-A1244 related cluster	3.0
VVTU9498_at	GSVIVP00002561001	Q5VRN4 Hypothetical protein OSJNBa0004I20.1 related cluster	3.0
VVTU19891_at	GSVIVP00022898001	Q9FQE1 glutathione S-transferase GST 17 related cluster	3.0
VVTU26668_at	CF517509	Q1T553 Reverse transcriptase (RNA-dependent DNA polymerase), putative related cluster	3.0
VVTU22207_at	GSVIVP00037958001	Q1SMR9 Pathogenesis-related transcriptional factor and ERF related cluster	3.0
VVTU2837_s_at	GSVIVP00009539001	Q6RZW7 Putative ethylene response factor 5 related cluster	3.0
VVTU4665_at	TC70031	P13940 lateembryogenesis abundant protein D-29 related cluster	3.0
VVTU29087_at	GSVIVP00037544001	O23380 Glucosyltransferase related cluster	3.0
VVTU40324_at	GSVIVP00014328001	Q6ZD72 Hypothetical protein P0450B04.17 related cluster	3.0
VVTU14997_s_at	GSVIVP00024538001	Q9FF51 Emb|CAB72159.1 related cluster	2.9
VVTU32256_x_at	GSVIVP00014992001	UPI00005DC1C7 Cluster related to UPI00005DC1C7; protein binding	2.9
VVTU29284_at	GSVIVP00011194001	Q8S8Z5 Syringolide-induced protein B13-1-1 related cluster	2.9
VVTU34108_at	CA816863	Q7XU53 OSJNBa0006A01.18 protein related cluster	2.9
VVTU15030_at	GSVIVP00034555001	Q6Z6D9 Putative 6-4 photolyase related cluster	2.9
VVTU11909_at	GSVIVP00019747001	Q8RXW0 Protein At3g17611 related cluster	2.9
VVTU7470_at	GSVIVP00029825001	Q1S4Y7 Berberine and berberine like, putative related cluster	2.9
VVTU21014_at	GSVIVP00022423001	Q0EDB1 Myb-related transcription factor VvMybA22-cs related cluster	2.9
VVTU13480_s_at	GSVIVP00015436001	Q39134 Amino acid permease related cluster	2.9
VVTU21530_s_at	GSVIVP00032899001	Q7XBP8 Ubiquitin Actin fusion protein 2 related cluster	2.9
VVTU37609_at	GSVIVP00029823001	Q3E9Y2 Protein At4g20830 related cluster	2.9
VVTU17697_at	GSVIVP00038482001	Q93WL3 Hypothetical protein At4g25370 related cluster	2.9
VVTU16974_s_at	GSVIVP00038536001	Q8LIX4 Hypothetical protein OSJNBb0053G03.20 related cluster	2.9
VVTU12858_at	GSVIVP00000396001	Q946Z2 Putative acyl-CoA synthetase related cluster	2.9

Probe set	Unique Gene ID	Annotation	Fold-change
VVTU32683_at	GSVIVP00024357001	Q9SKZ2 Putative CCR4-associated factor related cluster	2.9
VVTU38420_at	GSVIVP00028665001	O22216 Glycerol-3-phosphate dehydrogenase related cluster	2.9
VVTU15831_at	TC55733	Q3E9K3 Protein At5g06980 related cluster	2.9
VVTU28418_at	GSVIVP00016194001	Q1S9M3 Lipase, active site related cluster	2.9
VVTU2863_at	GSVIVP00036239001	Q43537 ORF related cluster	2.9
VVTU14707_at	GSVIVP00029375001	Q1S126 Hypothetical protein related cluster	2.9
VVTU20947_at	VVTU20947_at	UPI0000D5738B Cluster related to UPI0000D5738B; PREDICTED: similar to CG4792-PA	2.9
VVTU8211_s_at	GSVIVP00030292001	Q4G3H5 RAV transcription factor related cluster	2.9
VVTU40342_at	TC55783	Q1S379 auxin responsive SAUR protein related cluster	2.9
VVTU2645_at	GSVIVP00031383001	O24145 4-coumarate--CoA ligase 1 related cluster	2.8
VVTU30536_at	CB969943	Q8H6Q8 CTV.20 related cluster	2.8
VVTU1222_at	GSVIVP00020394001	Q1L5W8 Beta-amylase 1 related cluster	2.8
VVTU11054_at	GSVIVP00029466001	Q9M6E1 DNA-bindinG protein 3 related cluster	2.8
VVTU5830_at	GSVIVP00016176001	Q1S9M3 Lipase, active site related cluster	2.8
VVTU14447_at	VVTU14447_at	Q1SAF9 Mov34 MPN PAD-1 related cluster	2.8
VVTU17013_at	GSVIVP00002446001	Q6R7N3 Putative WRKY transcription factor 30 related cluster	2.8
VVTU14689_at	TC71044	Q1S8G9 Hypothetical protein related cluster	2.8
VVTU17039_at	GSVIVP00028150001	Q1RU28 Hypothetical protein related cluster	2.8
VVTU10418_at	VVTU10418_at	Q5ZA84 Embryogenesis transmembrane protein-like related cluster	2.8
VVTU11927_at	GSVIVP00033537001	Q52QX9 Aldo keto reductase AKR related cluster	2.8
VVTU13872_at	GSVIVP00011453001	Q9FKQ1 Emb|CAB89401.1 related cluster	2.8
VVTU26268_at	CF606083	P46286 60S ribosomal protein L8-1 related cluster	2.8
VVTU11701_at	VVTU11701_at	Q40545 pyruvate kinase isozyme A, chloroplast precursor related cluster	2.8
VVTU16581_at	GSVIVP00016120001	Q9XIJ5 T10O24.19 related cluster	2.8
VVTU14579_at	GSVIVP00003419001	Q3ECB0 Protein At1g78520 related cluster	2.8
VVTU1989_at	GSVIVP00002589001	Q9LHE6 Arabidopsis thaliana genomic DNA, chromosome 3, P1 clone: MZE19 related cluster	2.8
VVTU5052_at	GSVIVP00038099001	Q1SHV7 GDSL-like lipaseAcylhydrolase related cluster	2.8
VVTU6339_at	GSVIVP00020606001	P52425 Glycerol-3-phosphate dehydrogenase [NAD+] related cluster	2.8
VVTU40693_at	TC66801	Q17CQ1 Hypothetical protein related cluster	2.7
VVTU9364_at	TC55587	Q1SV12 Kunitz inhibitor ST1-like related cluster	2.7
VVTU5436_x_at	GSVIVP00037740001	Q84TK8 Hypothetical protein related cluster	2.7
VVTU11329_at	GSVIVP00030027001	UPI000034F3E8 Cluster related to UPI000034F3E8; catalytic hydrolase	2.7
VVTU6661_at	GSVIVP00005104001	Q1RVF4 dirigent-like protein related cluster	2.7
VVTU1315_at	TC61492	Q9M4H3 Putative Metallothionein-like protein related cluster	2.7
VVTU3310_s_at	GSVIVP00016789001	Q1SD74 Hypothetical protein related cluster	2.7
VVTU16440_s_at	GSVIVP00003062001	Q6TKQ4 Putative ethylene response factor ERF3a related cluster	2.7
VVTU13941_at	GSVIVP00002118001	Q1SSK6 Heat shock protein HSP20 related cluster	2.7
VVTU6018_at	GSVIVP00005362001	Q9SGP6 F3M18.8 related cluster	2.7
VVTU21038_x_at	GSVIVP00003759001	Q5MJV5 Avr9 Cf-9 rapidly elicited protein 256 related cluster	2.7
VVTU40826_s_at	TC54289	Q9SQ55 Nuclear RNA bindinG protein A related cluster	2.7
VVTU11955_s_at	GSVIVP00035908001	Q9C5G6 Hypothetical protein At1g23430 related cluster	2.7
VVTU14975_s_at	GSVIVP00013875001	Q6E593 Benzoyl coenzyme A: benzyl alcohol benzoyl transferase related cluster	2.7
VVTU10904_at	VVTU10904_at	Q1SDX7 Cyclin-like F-box related cluster	2.7
VVTU32030_at	GSVIVP00014535001	Q9AR82 Thioredoxin h related cluster	2.7
VVTU15195_at	CB982913	Q9MAA7 Probable Gibberellin receptor GID1L1 related cluster	2.7
VVTU35979_at	GSVIVP00016087001	Q1T4R9 IMP dehydrogenase GMP reductase related cluster	2.7
VVTU5051_at	GSVIVP00007983001	Q9FIK4 4-nitrophenylphosphatase-like protein related cluster	2.7
VVTU31480_at	CB341933	Q1RXY0 Hypothetical protein related cluster	2.6
VVTU35889_at	GSVIVP00033975001	Q2PF04 Putative transporter-like protein related cluster	2.6
VVTU15000_s_at	GSVIVP00015670001	Q28EK6 CHEK1 related cluster	2.6
VVTU9962_at	GSVIVP00020821001	Q1S528 AAA ATPase related cluster	2.6
VVTU15204_at	GSVIVP00016057001	Q6NPD1 At5g62960 related cluster	2.6
VVTU12678_at	GSVIVP00022076001	Q3L8J0 CBF-like transcription factor related cluster	2.6
VVTU2456_s_at	GSVIVP00015347001	Q2PGC5 flavonol synthase related cluster	2.6
VVTU40387_at	TC68944	UPI000034F094 Cluster related to UPI000034F094; Hypothetical protein At4g15180	2.6
VVTU17573_at	GSVIVP00032646001	AF487826 Vitis vinifera putative serine hydrolase (BIG8.1) mRNA, complete cds.	2.6
VVTU17564_s_at	GSVIVP00009898001	AB097925 Vitis vinifera VvMybA3 mRNA for Myb-related transcription factor VvMybA3, complete cds.	2.6
VVTU31255_at	GSVIVP00023317001	Q0JIM3 Os01g0790900 protein related cluster	2.6
VVTU609_at	GSVIVP00003294001	Q1SH60 Disease Resistance protein; AAA ATPase related cluster	2.6
VVTU652_s_at	GSVIVP00019830001	AY634283 Vitis vinifera nhx1 antiporter mRNA, complete cds.	2.6
VVTU1286_s_at	GSVIVP00027444001	Q69VG1 Chitin-inducible Gibberellin-responsive protein 1 related cluster	2.6
VVTU24498_at	GSVIVP00024296001	Q9LNX6 F22G5.9 related cluster	2.6
VVTU246_at	GSVIVP00038692001	O82702 Vacuolar ATP synthase subunit G 1 related cluster	2.6
VVTU5819_at	CD801312	Q7Y1B3 Mitochondrial alternative oxidase 2 related cluster	2.6
VVTU28502_at	GSVIVP00034310001	Q1T3I2 AAA ATPase related cluster	2.6
VVTU21514_x_at	GSVIVP00024741001	Q9LID5 Disease Resistance response protein-like related cluster	2.6
VVTU9329_at	GSVIVP00035949001	Q76DY0 AG-motif binding protein-4 related cluster	2.6
VVTU17513_s_at	GSVIVP00008836001	Q9LHJ8 Similarity to unknown protein related cluster	2.6
VVTU25586_at	CN545722	Q01561 NADH-ubiquinone oxidoreductase chain 5 related cluster	2.6
VVTU11732_at	GSVIVP00000442001	Q9FN67 Gb|AAD56319.1 related cluster	2.6
VVTU11871_s_at	GSVIVP00025506001	AF305093 Vitis vinifera polygalacturonase inhibitinG protein mRNA, complete cds.	2.6
VVTU21508_at	GSVIVP00003703001	Q9ZVN3 T22H22.1 protein related cluster	2.6
VVTU13128_x_at	GSVIVP00018345001	Q6WHC0 Chloroplast small Heat shock protein class I related cluster	2.6
VVTU17777_at	GSVIVP00020317001	Q9C7Y2 Plastid protein, putative; 23108-24430 related cluster	2.6

Probe set	Unique Gene ID	Annotation	Fold-change
VVTU23567_at	GSVIVP00030672001	Q940S0 T5E21.14 T5E21.14 related cluster	2.5
VVTU1843_at	GSVIVP00029268001	Q8LE51 Yippee-like protein related cluster	2.5
VVTU9265_at	GSVIVP00001190001	UPI000034F0B9 Cluster related to UPI000034F0B9; electron carrier	2.5
VVTU6790_at	GSVIVP00031117001	Q8GXW7 Hypothetical protein At5g28040 F15F15_110 related cluster	2.5
VVTU22595_at	VVTU22595_at	Q9LFS2 Hypothetical protein F1N13_160 related cluster	2.5
VVTU3248_at	GSVIVP00028564001	Q6K989 Putative ATP-dependent transporter related cluster	2.5
VVTU12948_at	GSVIVP00010265001	Q5HZ39 At4g27460 related cluster	2.5
VVTU17855_at	GSVIVP00026374001	Q8VYK7 AT4g16520 dl4285c related cluster	2.5
VVTU4048_at	GSVIVP00034298001	Q9LIG0 Clavaminate synthase-like protein At3g21360 related cluster	2.5
VVTU25373_at	GSVIVP00024309001	Q6KAC4 Putative X1 related cluster	2.5
VVTU35718_at	GSVIVP00024738001	Q9LID5 Disease Resistance response protein-like related cluster	2.5
VVTU33373_s_at	GSVIVP00013871001	Q9FGC2 DNA helicase-like related cluster	2.5
VVTU1808_at	TC70535	Q9SUD9 Hypothetical protein T13J8.110 related cluster	2.5
VVTU23936_at	DT030644	UPI00003C004D Cluster related to UPI00003C004D; PREDICTED: similar to centromere protein E	2.5
VVTU10167_at	VVTU10167_at	Q2HVT9 Thioredoxin-related related cluster	2.5
VVTU40505_at	TC69489	Q0PEL6 Putative Integrase related cluster	2.5
VVTU27091_at	CF414894	Q9FQZ5 Avr9 Cf-9 rapidly elicited protein 169 related cluster	2.5
VVTU1079_at	GSVIVP00015115001	Q9LTA2 Similarity to AT-hook DNA-bindinG protein related cluster	2.5
VVTU36158_at	TC63558	Q27GM0 Protein At2g07695 related cluster	2.5
VVTU29073_at	CD012132	Q1SS89 Integrase, catalytic region related cluster	2.5
VVTU38266_s_at	GSVIVP00035348001	Q1S4B7 TGF-beta receptor, type I II extracellular region; ABC transporter related related cluster	2.5
VVTU10042_s_at	GSVIVP00028001001	Q1RSR6 Glycoside hydrolase, family 1 related cluster	2.5
VVTU13775_at	GSVIVP00004134001	Q9LDL7 SCARECROW gene regulator-like related cluster	2.5
VVTU7427_at	GSVIVP00017622001	Q9M9A2 F27J15.21 related cluster	2.5
VVTU24805_at	GSVIVP00004762001	Q3ZTF5 Trehalose-phosphate phosphatase related cluster	2.5
VVTU33494_at	CA809289	Q8S8K8 Expressed protein related cluster	2.5
VVTU27988_at	GSVIVP00036599001	Q8LF37 Cytochrome P450, putative related cluster	2.5
VVTU21831_at	VVTU21831_at	Q1RZK4 Hypothetical protein related cluster	2.5
VVTU33583_at	CB341046	Q1SB30 HSF ETS, DNA-binding related cluster	2.5
VVTU38412_at	GSVIVP00026569001	Q19PN8 TIR-NBS-LRR type Disease Resistance protein related cluster	2.5
VVTU11627_at	GSVIVP00019873001	Q8W0Z6 At2g20790 F5H14.24 related cluster	2.5
VVTU181_at	GSVIVP00017888001	Q1SIZ0 Zinc finger, NF-X1-type; Single-stranded Nucleic acid binding R3H; Zinc finger, RING-type; Zinc finger, PHD-type related cluster	2.5
VVTU33048_at	GSVIVP00024211001	Q9ZW24 Putative glutathione S-transferase related cluster	2.5
VVTU2530_at	GSVIVP00031791001	Q9C8N5 Zinc finger protein, putative; 58191-56692 related cluster	2.5
VVTU2403_at	GSVIVP00012657001	Q9ZT07 receptor-like protein kinase related cluster	2.5
VVTU25414_at	GSVIVP00031796001	Q9LNK3 F12K21.25 related cluster	2.5
VVTU1533_at	GSVIVP00030581001	Q7Y036 MutT-like protein related cluster	2.5
VVTU21931_s_at	GSVIVP00002880001	Q7XTK8 catalase related cluster	2.5
VVTU21329_at	GSVIVP00013897001	Q2LAK3 Cytochrome P450 monooxygenase CYP89H3 related cluster	2.5
VVTU5931_at	GSVIVP00003135001	Q9LHE8 Arabidopsis thaliana genomic DNA, chromosome 3, P1 clone: MZE19 related cluster	2.5
VVTU7906_at	GSVIVP00023866001	Q9SXS8 ethylene-responsive transcription factor 3 related cluster	2.5
VVTU16493_at	GSVIVP00029379001	Q9SIY5 Chloroplast lumen common protein family related cluster	2.4
VVTU13390_x_at	GSVIVP00027238001	Q39Q09 Hypothetical protein related cluster	2.4
VVTU39783_at	GSVIVP00022086001	Q6JJ29 Prephenate dehydratase related cluster	2.4
VVTU31457_s_at	GSVIVP00029527001	O80631 Hypothetical protein At2g39440 related cluster	2.4
VVTU2157_at	GSVIVP00020259001	Q84JP7 Phosphoenolpyruvate carboxylase kinase 2 related cluster	2.4
VVTU15742_at	CN545589	Q10M91 VQ motif family protein, expressed related cluster	2.4
VVTU20403_at	VVTU20403_at	UPI00003C0762 Cluster related to UPI00003C0762; PREDICTED: similar to ribosomal protein L18 CG8615-PA	2.4
VVTU1572_at	GSVIVP00002810001	Q6UA14 Fiber protein Fb25 related cluster	2.4
VVTU2674_at	GSVIVP00017934001	Q7XYY0 AKIN gamma related cluster	2.4
VVTU2849_at	GSVIVP00001464001	Q9LJN4 Beta-1,4-xylosidase related cluster	2.4
VVTU35489_at	GSVIVP00036966001	Q9CA57 Putative glutathione S-transferase; 80986-80207 related cluster	2.4
VVTU2502_at	TC66625	Q9SX33 Putative phospholipid-transporting ATPase 9 related cluster	2.4
VVTU6246_at	GSVIVP00021444001	Q1T2U5 SOUL heme-binding protein; Bacterial regulatory factor, effector related cluster	2.4
VVTU22798_s_at	GSVIVP00021411001	Q3E7K3 Protein At5g02020 related cluster	2.4
VVTU21231_at	GSVIVP00037796001	Q1SK92 AAA ATPase, central region; DEAD DEAH box helicase, N-terminal related cluster	2.4
VVTU9934_at	TC54704	Q6RX30 RPP13-like protein related cluster	2.4
VVTU4318_at	DY473668	Q1EPJ3 DNA-binding WRKY domain-containinG protein related cluster	2.4
VVTU19781_at	GSVIVP00006687001	Q03686 Luminal-bindinG protein 8 related cluster	2.4
VVTU20129_at	GSVIVP00036284001	Q0IZR6 Os09g0554000 protein related cluster	2.4
VVTU5788_at	GSVIVP00016580001	Q1S019 Hypothetical protein related cluster	2.4
VVTU15263_at	GSVIVP00012561001	Q1T3J4 Protein kinase; EPSP synthase related cluster	2.4
VVTU30295_at	GSVIVP00034242001	UPI000034EDBF Cluster related to UPI000034EDBF; ATP binding protein binding protein kinase protein serine threonine kinase protein-tyrosine kinase	2.4
VVTU34655_at	GSVIVP00024134001	Q9LUX9 1,4-benzoquinone reductase-like related cluster	2.4
VVTU13518_at	GSVIVP00032463001	P46644 Aspartate aminotransferase, chloroplast precursor related cluster	2.4
VVTU32400_at	CB347187	Q9M4H3 Putative Metallothionein-like protein related cluster	2.4
VVTU4325_s_at	GSVIVP00032752001	Q1S365 Lissencephaly type-1-like homology motif; CTLH, C-terminal to LisH motif; WD40-like; Quinonprotein alcohol dehydrogenase-like related cluster	2.4
VVTU8177_at	GSVIVP00013392001	Q9LYV6 ABA-responsive protein-like related cluster	2.4
VVTU33466_at	CA809580	Q6L3K5 Senescence-associated protein, putative related cluster	2.4

Probe set	Unique Gene ID	Annotation	Fold-change
VVTU24878_at	DT006469	Q0E285 Os02g0261600 protein related cluster	2.4
VVTU13299_at	GSVIVP00028448001	Q1SA57 Hypothetical protein related cluster	2.4
VVTU10036_at	GSVIVP00037441001	Q1RZM6 Hypothetical protein related cluster	2.4
VVTU35332_at	GSVIVP00035070001	Q9FN75 AAA-type ATPase-like protein related cluster	2.4
VVTU38112_at	GSVIVP00027507001	Q40100 Secreted glycoprotein 3 related cluster	2.4
VVTU3583_at	GSVIVP00011024001	Q38M75 Ripening regulated protein-like related cluster	2.4
VVTU25716_at	GSVIVP00026074001	Q9LRJ9 Similarity to receptor kinase related cluster	2.4
VVTU15983_at	CF518888	Q2R3B9 Expressed protein related cluster	2.4
VVTU25694_s_at	GSVIVP00026451001	Q6ZI82 Putative T-complex protein 11 related cluster	2.3
VVTU15775_at	GSVIVP00035886001	Q1SVR0 Helix-loop-helix DNA-binding related cluster	2.3
VVTU12337_at	GSVIVP00002618001	Q84VQ7 Alpha-galactosidase related cluster	2.3
VVTU24739_at	GSVIVP00037835001	O64757 Putative Disease Resistance protein related cluster	2.3
VVTU6768_at	GSVIVP00022492001	Q0IPV0 Os12g0166000 protein related cluster	2.3
VVTU32491_s_at	GSVIVP00029440001	Q2QYK6 Chalcone-flavanone isomerase family protein, expressed related cluster	2.3
VVTU38619_at	GSVIVP00000528001	O22980 T19F6.9 protein related cluster	2.3
VVTU32121_at	CB349010	P48502 Ubiquinol-cytochrome c reductase complex 14 kDa protein related cluster	2.3
VVTU22385_x_at	GSVIVP00029189001	O82615 T9A4.6 protein related cluster	2.3
VVTU33702_at	CB339544	Q5M9R1 Hypothetical protein orf138c related cluster	2.3
VVTU14486_at	GSVIVP00033200001	Q1SGV9 Dimeric alpha-beta barrel related cluster	2.3
VVTU1412_at	GSVIVP00001132001	Q651M3 Hypothetical protein OSJNBa0047P18.32-1 related cluster	2.3
VVTU30962_at	GSVIVP00025209001	Q1SGQ5 Protein kinase related cluster	2.3
VVTU13165_at	GSVIVP00001048001	Q9M4G8 Putative Ripening-related P-450 enzyme related cluster	2.3
VVTU34378_at	GSVIVP00035821001	Q9SXK8 Heat shock factor related cluster	2.3
VVTU15957_s_at	GSVIVP00006668001	Q6YEY5 glutathione S-transferase related cluster	2.3
VVTU32208_at	CB348377	Q5YJR0 Ubiquitin-conjugating enzyme 9 related cluster	2.3
VVTU7240_at	GSVIVP00015883001	Q1T3X9 Response regulator, RegA PrrA ActR type related cluster	2.3
VVTU16717_at	GSVIVP00013415001	Q9T074 Phosphoenolpyruvate carboxykinase [ATP] related cluster	2.3
VVTU22840_s_at	GSVIVP00000070001	Q9SSF9 F25A4.28 protein related cluster	2.3
VVTU26621_at	CF518299	Q1SD84 Integrase, catalytic region related cluster	2.3
VVTU19505_at	GSVIVP00018516001	UPI00005C0629 Cluster related to UPI00005C0629; PREDICTED: similar to germinal Histone H4 gene	2.3
VVTU9490_s_at	GSVIVP00008846001	P25766 Ras-related protein RGP1 related cluster	2.3
VVTU6812_at	GSVIVP00036588001	Q6VVW7 DEM2 related cluster	2.3
VVTU11742_at	GSVIVP00025032001	Q9M9P8 T17B22.1 protein related cluster	2.3
VVTU39468_at	CB979639	Q1SSC3 Hypothetical protein related cluster	2.3
VVTU26285_at	GSVIVP00013936001	O80406 Phenylalanine ammonia-lyase related cluster	2.3
VVTU8264_at	GSVIVP00023306001	Q2MJ10 Cytochrome P450 monooxygenase CYP98A37 related cluster	2.3
VVTU4986_at	TC69247	Q9LU44 Similarity to transcription or splicing factor related cluster	2.3
VVTU3559_at	TC52576	Q10PL7 Poly polymerase catalytic domain containing protein, expressed related cluster	2.3
VVTU26850_at	GSVIVP00035638001	Q9MFE3 cytochrome c biogenesis protein related cluster	2.3
VVTU17126_s_at	GSVIVP00020034001	Q1HL00 Selenocysteine methyltransferase related cluster	2.3
VVTU14346_s_at	GSVIVP00025925001	Q1S4G4 Hypothetical protein related cluster	2.3
VVTU21899_at	GSVIVP00027054001	Q9M3B1 Hypothetical protein F2K15.70 related cluster	2.3
VVTU1500_at	GSVIVP00032358001	Q0GPE8 BZIP transcription factor bZIP16 related cluster	2.3
VVTU20006_at	GSVIVP00028924001	Q4JIY3 Pyruvate dehydrogenase related cluster	2.3
VVTU15590_at	GSVIVP00014074001	Q8LDN6 Putative gluconokinase related cluster	2.3
VVTU28623_at	GSVIVP00030454001	Q8LCU9 Hypothetical protein related cluster	2.3
VVTU31097_s_at	TC53372	Q2HUL7 Integrase, catalytic region; Zinc finger, CCHC-type related cluster	2.3
VVTU21439_at	TC61731	Q1S7J8 Cyclin-like F-box related cluster	2.3
VVTU4824_at	GSVIVP00032938001	Q8L8L6 Hypothetical protein related cluster	2.3
VVTU39003_at	TC57222	Q6L975 GAG-POL related cluster	2.3
VVTU32098_at	CB349140	Q8L9Y1 ADP,ATP carrier-like protein related cluster	2.3
VVTU37156_at	GSVIVP00028797001	Q76FX0 CCAAT-box binding factor HAP2 homolog related cluster	2.3
VVTU6550_at	TC60019	Q1SBN3 Hypothetical protein related cluster	2.3
VVTU463_at	VVTU463_at	Q86HC0 Hypothetical protein related cluster	2.3
VVTU15463_x_at	GSVIVP00021730001	Q6Q3H2 terpenoid synthetase related cluster	2.3
VVTU36598_at	GSVIVP00035257001	Q5M9Y0 Hypothetical protein orf155 related cluster	2.3
VVTU31346_x_at	GSVIVP00035596001	Q0ZIZ0 Photosystem II phosphoprotein related cluster	2.3
VVTU24254_at	GSVIVP00028952001	Q9SY81 F14N23.27 related cluster	2.3
VVTU8346_at	GSVIVP00000626001	Q8S8F1 Expressed protein related cluster	2.3
VVTU25680_x_at	GSVIVP00031201001	Q6VAB3 UDP-glycosyltransferase 85A8 related cluster	2.3
VVTU15608_at	GSVIVP00018812001	Q9SXK8 Heat shock factor related cluster	2.3
VVTU932_s_at	GSVIVP00014676001	Q9M9N8 NAM-like protein related cluster	2.3
VVTU40465_at	GSVIVP00023110001	Q940P8 AT5g20890 F22D1_60 related cluster	2.3
VVTU265_at	VVTU265_at	Q8S8T8 Expressed protein related cluster	2.3
VVTU2139_at	TC56922	Q9LXJ1 Hypothetical protein F3C22_140 related cluster	2.3
VVTU13256_at	GSVIVP00034299001	Q9LIG0 Clavaminate synthase-like protein At3g21360 related cluster	2.3
VVTU6092_at	TC65987	Q8L8G0 Nam-like protein 1 related cluster	2.2
VVTU1687_at	GSVIVP00020668001	Q9FI63 Arabidopsis thaliana genomic DNA, chromosome 5, TAC clone:K21I16 related cluster	2.2
VVTU9298_at	GSVIVP00015274001	Q5N9Z8 Putative Aspartate aminotransferase related cluster	2.2
VVTU35479_at	GSVIVP00007533001	Q270G6 PE-PGRS family protein precursor related cluster	2.2
VVTU31231_at	GSVIVP00029924001	Q1SMG9 Extradiol ring-cleavage dioxygenase, class III enzyme, subunit B related cluster	2.2
VVTU16467_s_at	GSVIVP00025486001	Q9SVQ9 Short-chain alcohol dehydrogenase like protein related cluster	2.2
VVTU728_s_at	CK136902	P19023 ATP synthase subunit beta, mitochondrial precursor related cluster	2.2
VVTU20779_at	GSVIVP00037841001	Q9FM30 Similarity to unknown protein related cluster	2.2

Probe set	Unique Gene ID	Annotation	Fold-change
VVTU13257_s_at	GSVIVP00000491001	Q0IYU2 Os10g0162100 protein related cluster	2.2
VVTU2687_s_at	GSVIVP00027636001	Q9SZS0 Hypothetical protein F27G19.50 related cluster	2.2
VVTU703_s_at	GSVIVP00018175001	Q94C45 Phenylalanine ammonia-lyase 1 related cluster	2.2
VVTU1061_at	TC61308	AY159561 Vitis vinifera submergence induced protein 2-like (SIP) mRNA, partial cds.	2.2
VVTU15740_at	GSVIVP00016199001	Q9FNR3 Leucine zipper protein-like related cluster	2.2
VVTU34796_s_at	GSVIVP00036823001	Q651Z9 Hypothetical protein OJ1439_F07.29 related cluster	2.2
VVTU5774_at	GSVIVP00002724001	Q8L5Y9 Pantothenate kinase 2 related cluster	2.2
VVTU11966_s_at	TC67774	Q9C9D1 Putative cytochrome P-450; 4810-6511 related cluster	2.2
VVTU14375_s_at	GSVIVP00021335001	Q9M4H5 Putative Ripening-related protein related cluster	2.2
VVTU36233_at	TC57541	Q10M26 Retrotransposon protein, putative, Ty1-copia subclass, expressed related cluster	2.2
VVTU3077_at	GSVIVP00017234001	P93338 NADP-dependent Glyceraldehyde-3-phosphate dehydrogenase related cluster	2.2
VVTU15268_at	GSVIVP00021629001	Q9SJM8 Expressed protein related cluster	2.2
VVTU35551_s_at	GSVIVP00022807001	Q8LKS5 Long chain acyl-CoA synthetase 7 related cluster	2.2
VVTU28714_at	CD717464	Q2QZX3 Retrotransposon protein, putative, Ty1-copia subclass related cluster	2.2
VVTU22671_at	VVTU22671_at	Q0DHC6 Os05g0476000 protein related cluster	2.2
VVTU31548_at	CB341404	Q5M9Z9 Hypothetical protein orf106b related cluster	2.2
VVTU33015_at	CA814508	Q940V6 Heat shock transcription factor related cluster	2.2
VVTU13276_s_at	GSVIVP00037218001	Q0INB8 Os12g0484700 protein related cluster	2.2
VVTU20293_at	GSVIVP00023167001	Q6PP04 peroxidase related cluster	2.2
VVTU2471_at	TC67236	Q8L8Z0 Hypothetical protein related cluster	2.2
VVTU2504_s_at	GSVIVP00030663001	Q6TY49 Reductase 1 related cluster	2.2
VVTU38345_at	GSVIVP00027204001	Q5JMF1 Hypothetical protein P0512C01.34 related cluster	2.2
VVTU15417_at	GSVIVP00027372001	Q69Y38 Hypothetical protein P0021H10.8-1 related cluster	2.2
VVTU33470_s_at	GSVIVP00025542001	Q5ZA08 Putative protease related cluster	2.2
VVTU1456_s_at	GSVIVP00000683001	Q9M1J1 Hypothetical protein F24I3.170 related cluster	2.2
VVTU32458_at	CB346720	P62577 Chloramphenicol acetyltransferase related cluster	2.2
VVTU37383_s_at	GSVIVP00012010001	Q9SDM7 Hypothetical protein related cluster	2.2
VVTU22137_at	GSVIVP00020168001	Q3Y6V1 cellulose synthase-like protein CslG related cluster	2.2
VVTU22327_at	GSVIVP00028997001	P40973 Pectate lyase precursor related cluster	2.2
VVTU12576_at	GSVIVP00019400001	Q9SJ02 Expressed protein related cluster	2.2
VVTU28268_at	CF074523	Q0UUC3 Hypothetical protein related cluster	2.2
VVTU19941_at	GSVIVP00018981001	Q8S7E5 Putative phragmoplastin related cluster	2.2
VVTU17058_at	GSVIVP00026068001	O23378 Hypothetical protein dl3665c related cluster	2.2
VVTU1905_x_at	GSVIVP00025581001	Q1SMG9 Extradiol ring-cleavage dioxygenase, class III enzyme, subunit B related cluster	2.2
VVTU10900_at	VVTU10900_at	Q1S323 Hypothetical protein related cluster	2.2
VVTU17747_at	GSVIVP00021034001	Q32ZJ3 DND1-like protein related cluster	2.2
VVTU25111_at	DT004114	Q1S7J8 Cyclin-like F-box related cluster	2.2
VVTU12350_at	GSVIVP00021636001	Q9SJM6 Zinc finger A20 and AN1 domains-containinG protein At2g36320 related cluster	2.2
VVTU8140_at	GSVIVP00014946001	Q0PN09 Lateral organ boundaries domain protein related cluster	2.2
VVTU12385_at	GSVIVP00028094001	Q94KD8 At1g02640 T14P4_11 related cluster	2.2
VVTU33153_x_at	CA812309	Q9M4H3 Putative Metallothionein-like protein related cluster	2.2
VVTU17057_s_at	GSVIVP00032572001	Q8S8K8 Expressed protein related cluster	2.2
VVTU105_at	GSVIVP00000651001	Q6K4N6 Amino acid transporter-like related cluster	2.2
VVTU13862_at	GSVIVP00029417001	Q9LYL4 Hypothetical protein F18O21_250 related cluster	2.1
VVTU3914_s_at	GSVIVP00036021001	O82062 39 kDa EF-Hand containinG protein related cluster	2.1
VVTU24741_x_at	GSVIVP00009234001	P28343 stilbene synthase 1 related cluster	2.1
VVTU6376_at	GSVIVP00017259001	Q2PF06 Putative hydroxymethylglutaryl-CoA lyase related cluster	2.1
VVTU37177_at	GSVIVP00027346001	Q7XIL3 Putative 5-alpha-taxadienol-10-beta-hydroxylase related cluster	2.1
VVTU17030_s_at	GSVIVP00021908001	Q76DL0 LEDI-5c protein related cluster	2.1
VVTU1660_at	CB917669	Q655Y3 Dreg-2 like protein related cluster	2.1
VVTU26945_at	CF512551	Q8GYD0 Hypothetical protein At1g61670 T13M11_2 related cluster	2.1
VVTU39640_x_at	GSVIVP00004974001	Q1SVV2 AAA ATPase related cluster	2.1
VVTU26352_at	GSVIVP00006600001	P93378 Tumor-related protein related cluster	2.1
VVTU38943_s_at	GSVIVP00003476001	Q5CD68 Monoterpene Glucosyltransferase related cluster	2.1
VVTU14638_at	GSVIVP00037325001	P42036 40S ribosomal protein S14-3 related cluster	2.1
VVTU27648_x_at	GSVIVP00014518001	Q94AP3 Putative nodulin protein related cluster	2.1
VVTU2944_at	GSVIVP00018860001	Q9FQD6 glutathione S-transferase GST 22 related cluster	2.1
VVTU27412_at	GSVIVP00017745001	Q1S9T2 E-class P450, group I related cluster	2.1
VVTU35782_at	GSVIVP00008615001	Q9ZPY9 Expressed protein related cluster	2.1
VVTU31250_at	CB343306	Q9S7V6 F17A17.13 protein related cluster	2.1
VVTU26793_at	GSVIVP00038789001	Q6T3R3 Bacterial spot Disease Resistance protein 4 related cluster	2.1
VVTU35047_at	GSVIVP00036443001	Q5GLZ7 Non-symbiotic hemoglobin class 1 related cluster	2.1
VVTU465_at	VVTU465_at	Q8L9U8 Hypothetical protein related cluster	2.1
VVTU14402_at	GSVIVP00025041001	O80798 T8F5.4 protein related cluster	2.1
VVTU22839_at	GSVIVP00012684001	Q949G3 pleiotropic drug Resistance protein 1 related cluster	2.1
VVTU29362_at	GSVIVP00037785001	Q1S8T9 Protein kinase related cluster	2.1
VVTU17024_at	GSVIVP00002729001	Q8LR34 Putative iron-sulfur cofactor synthesis protein iscU related cluster	2.1
VVTU13111_at	GSVIVP00016337001	Q1SMY1 homeodomain-related related cluster	2.1
VVTU11695_s_at	GSVIVP00023610001	Q40487 Cationic peroxidase isozyme 40K precursor related cluster	2.1
VVTU16784_s_at	GSVIVP00000409001	Q6A4W8 Glutathione peroxidase related cluster	2.1
VVTU19123_at	GSVIVP00029513001	P51117 Chalcone--flavonone isomerase related cluster	2.1
VVTU2490_at	GSVIVP00018449001	Q1SGH7 Zinc finger, RING-type; Thioredoxin-related related cluster	2.1
VVTU32173_at	CB348577	Q9LKK8 Putative 7-transmembrane G-protein-coupled receptor related cluster	2.1
VVTU9800_at	GSVIVP00033430001	Q0PN11 BZIP transcription factor related cluster	2.1
VVTU11975_s_at	GSVIVP00015547001	Q41111dehydrin related cluster	2.1
VVTU5567_at	GSVIVP00022575001	Q9ZR47 Neutral invrtase related cluster	2.1

Probe set	Unique Gene ID	Annotation	Fold-change
VVTU165_at	GSVIVP00038115001	Q1S9P5 CAMP response element binding (CREB) protein related cluster	2.1
VVTU30441_s_at	GSVIVP00007023001	Q19PL2 TIR-NBS-LRR-TIR type Disease Resistance protein related cluster	2.1
VVTU2415_at	GSVIVP00025011001	Q8L3R2 calmodulin-like protein 41 related cluster	2.1
VVTU2434_at	GSVIVP00009599001	Q1SPA8 Glycosyl transferase, family 8 related cluster	2.1
VVTU2288_at	TC58973	Q6NMS1 At2g07725 related cluster	2.1
VVTU30648_s_at	GSVIVP00020998001	Q9FLJ2 NAM (No apical meristem)-like protein related cluster	2.1
VVTU2851_at	GSVIVP00016128001	Q0KII0 IS10 transposase, putative related cluster	2.1
VVTU2101_at	GSVIVP00011750001	Q1RVD7 Phosphoserine aminotransferase related cluster	2.1
VVTU11038_at	GSVIVP00006924001	Q9LXG3 peroxidase 56 precursor related cluster	2.1
VVTU2676_at	GSVIVP00015401001	Q1SJZ0 Cyclin-like F-box related cluster	2.1
VVTU25513_at	GSVIVP00020552001	Q2QUY9 Retrotransposon protein, putative, LINE subclass related cluster	2.1
VVTU11227_at	GSVIVP00037364001	Q9FMQ6 Arabidopsis thaliana genomic DNA, chromosome 5, P1 clone:MWD9 related cluster	2.1
VVTU20570_x_at	GSVIVP00036130001	Q1T416 SKP1 component related cluster	2.1
VVTU31170_x_at	TC59094	Q3HKB2 Hypothetical protein related cluster	2.1
VVTU13522_s_at	GSVIVP00018061001	Q64EX4 MtN19-like protein related cluster	2.1
VVTU5095_at	GSVIVP00009618001	Q96569 L-lactate dehydrogenase related cluster	2.1
VVTU33985_at	TC67922	Q9M510 Dicyanin related cluster	2.1
VVTU8229_at	GSVIVP00008737001	Q6ZH46 Hypothetical protein OJ1217_F02.5 related cluster	2.1
VVTU13570_s_at	GSVIVP00027239001	Q9XF70 Thioredoxin h related cluster	2.1
VVTU18585_at	GSVIVP00014791001	Q9LT08 26S proteasome non-ATPase regulatory subunit 14 related cluster	2.1
VVTU31996_at	CB349848	O04545 F20P5.27 protein related cluster	2.1
VVTU17500_at	GSVIVP00011972001	Q93YB1 Granule-bound Starch synthase precursor related cluster	2.1
VVTU3155_s_at	GSVIVP00038254001	Q4ACU1 Delta7 sterol C-5 desaturase related cluster	2.1
VVTU13620_at	GSVIVP00032630001	Q1S5C8 ATPase, AFG1 family, putative related cluster	2.1
VVTU34558_at	GSVIVP00036222001	Z54234 V.vinifera mRNA for Chitinase.	2.1
VVTU38757_at	TC54571	Q2HUL7 Integrase, catalytic region; Zinc finger, CCHC-type related cluster	2.1
VVTU4485_at	GSVIVP00014565001	Q9LW93 Nicotiana tabacum Wound inducive mRNA , complete cds related cluster	2.1
VVTU7778_at	GSVIVP00034070001	Q0PNH1 Cytochrome P450 related cluster	2.1
VVTU17570_at	GSVIVP00026605001	AF365879 Vitis vinifera clone GLP1-12 Resistance protein gene, partial cds.	2.1
VVTU2573_at	GSVIVP00031761001	Q1T0K8 Hypothetical protein related cluster	2.1
VVTU13738_at	GSVIVP00037718001	O49814 beta-carotene hydroxylase 2 related cluster	2.1
VVTU34383_at	BQ793147	Q0GEA1 26S proteasome regulatory particle non-ATPase subunit 12 related cluster	2.1
VVTU29965_at	GSVIVP00004097001	Q1SXF6 EGF-like related cluster	2.1
VVTU2474_at	TC61710	Q1SSW0 Protein phosphatase 2C related cluster	2.1
VVTU18949_s_at	GSVIVP00037910001	Q9FLM1 Similarity to unknown protein related cluster	2.1
VVTU4093_at	TC65025	Q9C9V8 Hypothetical protein T23K23.23 related cluster	2.1
VVTU914_at	GSVIVP00038153001	Q6L5E8 Putative cinnamoyl CoA reductase related cluster	2.1
VVTU10659_at	GSVIVP00018127001	Q9ZUV4 nodulin-like protein related cluster	2.1
VVTU12040_at	GSVIVP00028595001	Q08062 malatedehydrogenase, cytoplasmic related cluster	2.1
VVTU10613_at	GSVIVP00026922001	Q6YY41 Putative UDP-Glucosyltransferase related cluster	2.1
VVTU35789_at	GSVIVP00028994001	AY159555 Vitis vinifera putative hypersensitive-induced response protein (HIR) mRNA, partial cds.	2.1
VVTU11169_at	VVTU11169_at	Q1T1H3 Cadmium-transporting ATPase; ATPase, E1-E2 type related cluster	2.1
VVTU10671_at	GSVIVP00002922001	Q9S7N8 seed maturation protein PM21 related cluster	2.0
VVTU3082_at	GSVIVP00028977001	Q9LFY0 T7N9.13 related cluster	2.0
VVTU8469_at	VVTU8469_at	Q94JW8 Squamosa promoter-binding-like protein 6 related cluster	2.0
VVTU5854_at	GSVIVP00012529001	Q9SRT0 F21O3.7 protein related cluster	2.0
VVTU32255_s_at	GSVIVP00028587001	Q2TPW5 seed storage protein related cluster	2.0
VVTU4299_at	GSVIVP00026023001	P49608 Aconitate hydratase, cytoplasmic related cluster	2.0
VVTU6800_at	GSVIVP00023467001	Q0PJH7 Myb transcription factor Myb177 related cluster	2.0
VVTU31626_at	CB345759	P93394 Uracil phosphoribosyltransferase related cluster	2.0
VVTU9977_at	GSVIVP00035825001	Q84QD7 Avr9 Cf-9 rapidly elicited protein 276 related cluster	2.0
VVTU12307_s_at	GSVIVP00019627001	Q9S7N8 seed maturation protein PM21 related cluster	2.0
VVTU21549_at	GSVIVP00018322001	Q8S9A7 Glucosyltransferase-2 related cluster	2.0
VVTU12031_at	GSVIVP00033228001	Q8H6R4 Jp18 related cluster	2.0
VVTU4817_at	GSVIVP00032789001	Q8GZW5 Putative mitochondrial inner membrane protein related cluster	2.0
VVTU10888_at	GSVIVP00014235001	Q1SW57 HEC Ndc80p related cluster	2.0
VVTU401_at	GSVIVP00025768001	Q9LSI2 Gb|AAF27147.1 related cluster	2.0
VVTU544_at	GSVIVP00024147001	P48422 Cytochrome P450 86A1 related cluster	2.0
VVTU5791_at	GSVIVP00010274001	Q0IWN4 Os10g0498300 protein related cluster	2.0
VVTU1535_s_at	GSVIVP00037194001	Q1SHH7 auxin responsive SAUR protein related cluster	2.0
VVTU8656_at	GSVIVP00036870001	Q9SD45 Epoxide hydrolase-like protein related cluster	2.0
VVTU10017_s_at	GSVIVP00026898001	Q9LMN0 F22L4.5 protein related cluster	2.0
VVTU36933_at	GSVIVP00009475001	Q9XHP4 Peroxisomal copper-containing amine oxidase related cluster	2.0
VVTU4732_at	GSVIVP00018495001	O48813 Hypothetical protein At2g39650 related cluster	2.0
VVTU13407_s_at	GSVIVP00020717001	UPI00000A4A7E Cluster related to UPI00000A4A7E; putative ADP-ribosylation factor	2.0
VVTU22349_at	VVTU22349_at	Q9ZS34 Geranylgeranyl reductase related cluster	2.0
VVTU12747_s_at	GSVIVP00022869001	Q84KA9 RING C3HC4 PHD Zinc finger-like protein related cluster	2.0
VVTU2531_s_at	GSVIVP00015254001	Q94JT6 At1g78070 F28K19_28 related cluster	2.0
VVTU5686_at	GSVIVP00033126001	Q1HW69 BZIP transcription factor related cluster	2.0
VVTU31641_x_at	GSVIVP00000364001	Q9SSR1 F6D8.19 related cluster	2.0
VVTU36957_at	TC61352	Q8L7W4 AT4g17080 dl4570w related cluster	2.0
VVTU25750_x_at	CF207020	Q1SCC5 2OG-Fe(II) oxygenase related cluster	2.0
VVTU16150_at	GSVIVP00033117001	Q9MAW5 ribosomal protein L29 related cluster	2.0
VVTU12249_at	GSVIVP00011656001	Q1SMR1 Hypothetical protein related cluster	2.0

Probe set	Unique Gene ID	Annotation	Fold-change
VVTU12644_s_at	GSVIVP00017815001	Q93YA8 Calcium bindinG protein related cluster	2.0
VVTU31736_at	CB345203	P62577 Chloramphenicol acetyltransferase related cluster	2.0
VVTU36245_at	GSVIVP00012846001	Q43260 glutamate dehydrogenase related cluster	2.0
VVTU12032_s_at	GSVIVP00023932001	Q3HM04 Cinnamate-4-hydroxylse related cluster	2.0
VVTU39347_at	GSVIVP00020948001	Q4W5U8 FtsH protease related cluster	2.0
VVTU4140_at	TC65977	UPI00000A8F8C Cluster related to UPI00000A8F8C; P0410E03.16	2.0
VVTU17485_at	TC69038	Q1STD7 UDP-glucuronosyl UDP-Glucosyltransferase related cluster	2.0
VVTU39157_at	GSVIVP00003763001	Q9SEL2 Gag-pol Polyprotein related cluster	2.0
VVTU12641_s_at	GSVIVP00021421001	Q6I645 Hypothetical protein OJ1126_D01.5 related cluster	2.0
VVTU4019_at	GSVIVP00017764001	Q9FHB6 genomic DNA, chromosome 5, TAC clone:K24M7 related cluster	2.0
VVTU13916_s_at	GSVIVP00027738001	Q688X9 Hypothetical protein OJ1115_B06.15 related cluster	2.0
VVTU8476_at	DT025084	Q8L900 Putative RING Zinc finger protein related cluster	2.0
VVTU13583_s_at	GSVIVP00023673001	Q0DID5 Os05g0399100 protein related cluster	2.0
VVTU35075_s_at	GSVIVP00031723001	Q94IQ1 peroxidase related cluster	2.0
VVTU7096_at	GSVIVP00014035001	Q9M9M5 Putative 26S proteosome regulatory subunit related cluster	2.0
VVTU5825_at	GSVIVP00002884001	Q6ZH46 Hypothetical protein OJ1217_F02.5 related cluster	2.0
VVTU37360_at	TC67737	Q5MAL3 Coat protein related cluster	2.0
VVTU11881_at	GSVIVP00029394001	Q1RXL8 Amino acid polyamine transporter II related cluster	2.0
VVTU11941_at	GSVIVP00015396001	Q8S7E1 chlorophyllide a oxygenase, chloroplast precursor related cluster	2.0
VVTU268_at	GSVIVP00035712001	Q2QYA4 Hypothetical protein related cluster	2.0
VVTU15656_at	GSVIVP00024881001	Q534S9 SAMT related cluster	2.0
VVTU20262_at	VVTU20262_at	UPI000034F475 Cluster related to UPI000034F475; unknown protein	2.0
VVTU16011_at	GSVIVP00022331001	Q9ZU37 Predicted by genscan and genefinder related cluster	2.0
VVTU6382_at	GSVIVP00002541001	Q9SMH1 ACC synthase related cluster	2.0
VVTU15060_s_at	GSVIVP00025103001	Q8L9P3 Hypothetical protein related cluster	2.0
VVTU15921_at	GSVIVP00002927001	Q9SYQ1 Inorganic Phosphate transporter 1-8 (AtPht1;8) (H(+) Pi cotransporter) related cluster	2.0
VVTU36836_x_at	GSVIVP00027126001	Q0D8F9 Os07g0162700 protein related cluster	2.0
VVTU4946_at	GSVIVP00016957001	Q8RX87 AT5g20250 F5O24_140 related cluster	2.0
VVTU5860_s_at	GSVIVP00020332001	Q9SL05 Expressed protein related cluster	2.0
VVTU3965_at	GSVIVP00015100001	Q949K1 Hypothetical protein related cluster	2.0
VVTU7335_at	GSVIVP00023858001	Q9M9Y5 F4H5.13 protein related cluster	2.0
VVTU21843_at	GSVIVP00033339001	Q1SI93 E-class P450, group I related cluster	2.0
VVTU36526_at	GSVIVP00022757001	Q0ZCD0 Integrase related cluster	2.0
VVTU2860_at	GSVIVP00027730001	Q6TKQ3 Putative ethylene response factor ERF3b related cluster	2.0
VVTU36714_at	TC57105	Q6T6V4 Hypothetical protein related cluster	2.0
VVTU18269_s_at	GSVIVP00035360001	Q39819 HSP22.3 related cluster	2.0
VVTU7837_at	GSVIVP00014576001	Q0ZR50 Hypothetical protein related cluster	2.0
VVTU9099_s_at	GSVIVP00023117001	Q941D7 Hypothetical protein related cluster	2.0
VVTU19950_at	GSVIVP00007022001	Q39648 Non-photosynthetic Ferredoxin precursor related cluster	2.0
VVTU1874_at	GSVIVP00000520001	Q9SGU9 F1N19.23 related cluster	2.0
VVTU11804_at	GSVIVP00002571001	Q7M1N9 Transformer-2-like protein related cluster	2.0
VVTU22272_at	GSVIVP00028232001	Q9ZSI7 Probable WRKY transcription factor 47 related cluster	2.0
VVTU14454_at	GSVIVP00005756001	P49295 Glutamyl-tRNA reductase 2, chloroplast precursor related cluster	2.0
VVTU33709_at	CB339508	Q0ZIZ0 Photosystem II phosphoprotein related cluster	2.0
VVTU39179_at	GSVIVP00010971001	Q84MP5 Hypothetical protein OSJNBb0036F07.14 related cluster	2.0
VVTU738_at	GSVIVP00038899001	Q6DW76 Digalactosyldiacylglycerol synthase 1, chloroplast precursor related cluster	2.0
VVTU11268_at	VVTU11268_at	Q9M0C3 Hypothetical protein AT4g30370 related cluster	2.0
VVTU33718_x_at	CB339429	Q1RXY1 Hypothetical protein related cluster	2.0
VVTU34548_at	GSVIVP00009228001	AY670138 Vitis vinifera clone 357836_G2 stilbene synthase mRNA, partial cds.	2.0
VVTU35457_at	GSVIVP00005658001	Q1RW50 Hypothetical protein related cluster	2.0
VVTU37561_at	TC64748	Q4K6E8 Polysaccharide biosynthesis protein related cluster	2.0
VVTU11661_at	GSVIVP00001056001	Q9LWA3 Subtilisin-like protease related cluster	2.0
VVTU25406_at	GSVIVP00017356001	Q2RB18 GDSL-like lipaseAcylhydrolase family protein, expressed related cluster	2.0
VVTU6572_at	GSVIVP00029166001	Q9MBC0 polygalacturonase related cluster	2.0
VVTU12724_at	GSVIVP00026045001	Q0PJH5 Myb transcription factor Myb73 related cluster	2.0
VVTU3877_at	GSVIVP00018781001	Q9SJI1 Expressed protein related cluster	1.9
VVTU39343_at	TC54164	Q9XEB9 Putative Polyprotein related cluster	1.9
VVTU4756_at	GSVIVP00016152001	Q8W437 PBng143 related cluster	1.9
VVTU12237_at	GSVIVP00028403001	Q84MA5 At4g21120 related cluster	1.9
VVTU35658_at	GSVIVP00037020001	Q2HTW4 Protein kinase related cluster	1.9
VVTU16073_at	GSVIVP00003667001	Q9FQ14 proteinase inhibitor se60-like protein related cluster	1.9
VVTU4581_at	GSVIVP00003018001	O04681 Pathogenesis-related genes transcriptional activator PTI5 related cluster	1.9
VVTU1405_at	GSVIVP00038119001	Q6R3R2 CONSTANS-like protein CO1 related cluster	1.9
VVTU13756_at	GSVIVP00002451001	Q9FQZ4 Avr9 Cf-9 rapidly elicited protein 194 related cluster	1.9
VVTU1775_at	GSVIVP00032128001	Q9XGZ3 T1N24.3 protein related cluster	1.9
VVTU20648_at	VVTU20648_at	Q5U185 RE26656p related cluster	1.9
VVTU19944_at	TC63033	Q54L72 Putative GATA-binding transcription factor related cluster	1.9
VVTU31366_at	CB342849	Q0DZ68 Os02g0642600 protein related cluster	1.9
VVTU34661_at	GSVIVP00024948001	O82663 Succinate dehydrogenase [ubiquinone] flavoprotein subunit 1, mitochondrial precursor related cluster	1.9
VVTU1504_at	GSVIVP00023521001	Q94BY2 At1g65820 F1E22_4 related cluster	1.9
VVTU17103_at	TC67943	O81225 Extra-large G-protein related cluster	1.9
VVTU11707_at	GSVIVP00037978001	Q9FFY3 Arabidopsis thaliana genomic DNA, chromosome 5, P1 clone:MAC12 related cluster	1.9
VVTU14128_at	GSVIVP00020745001	Q7XHJ0 Formate dehydrogenase related cluster	1.9

Probe set	Unique Gene ID	Annotation	Fold-change
VVTU2433_at	GSVIVP00024282001	Q1SGH7 Zinc finger, RING-type; Thioredoxin-related related cluster	1.9
VVTU5025_at	GSVIVP00013574001	Q9SAC8 T16B5.12 protein related cluster	1.9
VVTU936_s_at	GSVIVP00024609001	Q3ECS6 Protein At1g51340 related cluster	1.9
VVTU6969_at	GSVIVP00030348001	Q1SUN0 Alpha beta hydrolase related cluster	1.9
VVTU27675_at	CF371758	Q69P58 Hypothetical protein OJ1740_D06.21 related cluster	1.9
VVTU3021_at	TC59474	O81977 Rudimentary enhancer related cluster	1.9
VVTU4898_at	GSVIVP00029908001	Q3ZTF5 Trehalose-phosphate phosphatase related cluster	1.9
VVTU8701_s_at	TC60646	Q9SRM8 T19F11.9 protein related cluster	1.9
VVTU15727_at	GSVIVP00025598001	Q2PYZ2 3 -5 -exoribonuclease RNA binding protein-like protein related cluster	1.9
VVTU18259_s_at	GSVIVP00011410001	Q6U1L7 Bax inhibitor related cluster	1.9
VVTU23330_at	DV938706	UPI00000A2CBF Cluster related to UPI00000A2CBF; P0408G07.7	1.9
VVTU25030_at	GSVIVP00027214001	Q8GZU1 Kinesin related protein related cluster	1.9
VVTU1377_at	GSVIVP00017237001	Q9SWV8 ethylene-responsive small GTP-bindinG protein related cluster	1.9
VVTU8144_at	GSVIVP00027099001	Q2HVN6 Hypothetical protein related cluster	1.9
VVTU34309_at	GSVIVP00016037001	Q0JM23 Os01g0548600 protein related cluster	1.9
VVTU26941_at	GSVIVP00014711001	Q6QLL5 WAK-like kinase related cluster	1.9
VVTU16183_at	GSVIVP00036363001	Q3KN67 Isoflavone reductase-like protein 6 related cluster	1.9
VVTU5783_s_at	GSVIVP00014477001	Q2MJ14 Cytochrome P450 monooxygenase CYP83H2 related cluster	1.9
VVTU5389_at	GSVIVP00015865001	Q1W203 NAK-type protein kinase related cluster	1.9
VVTU6196_at	GSVIVP00015262001	Q6Z8J0 Putative phosphoglycerate mutase related cluster	1.9
VVTU6851_at	GSVIVP00024794001	Q1RV75 Hypothetical protein related cluster	1.9
VVTU13252_s_at	GSVIVP00032890001	Q9M0W3 PolyUbiquitin related cluster	1.9
VVTU3358_at	GSVIVP00005208001	O64894 Acyl-coenzyme A oxidase, peroxisomal precursor related cluster	1.9
VVTU9687_at	GSVIVP00027228001	Q8S397 Sodium hydrogen exchanger 4 (Na(+) H(+) exchanger 4) related cluster	1.9
VVTU22365_at	GSVIVP00028223001	Q4VWW7 cellulose synthase related cluster	1.9
VVTU17273_s_at	GSVIVP00016633001	O48628 Pyrophosphate-dependent phosphofructo-1-kinase related cluster	1.9
VVTU28014_at	GSVIVP00024340001	Q9SNC4 Hypothetical protein F12A12.60 related cluster	1.9
VVTU16654_at	TC63371	O23193 Hypothetical protein C7A10.450 related cluster	1.9
VVTU29102_at	CD012055	Q5PP70 At5g44450 related cluster	1.9
VVTU5712_at	GSVIVP00006731001	Y18471 Vitis vinifera mRNA for Zinc finger protein (SINA1p).	1.9
VVTU16141_at	GSVIVP00027993001	Q8LEA8 Phytochrome A-associated F-box protein related cluster	1.9
VVTU36919_at	CF205562	Q1SP38 Hypothetical protein related cluster	1.9
VVTU4085_s_at	GSVIVP00010428001	Q9ZVR5 lectin-like protein related cluster	1.9
VVTU3108_at	GSVIVP00025564001	Q9SCL8 Guanylatekinase-like protein related cluster	1.9
VVTU8163_at	CB350096	Q8LT03 Leaf thionin Asthi1 related cluster	1.9
VVTU31362_x_at	CB342902	Q9SWB5 seed maturation protein PM37 related cluster	1.9
VVTU33796_s_at	GSVIVP00031667001	Q9LK38 Selenium-bindinG protein related cluster	1.9
VVTU11140_at	GSVIVP00023220001	Q3EAP5 Protein At3g45090 related cluster	1.9
VVTU33869_s_at	GSVIVP00002205001	Q8W5R4 Phosphoenolpyruvate carboxylase kinase related cluster	1.9
VVTU10204_at	GSVIVP00016857001	Q9LT81 Gb|AAC36178.1 related cluster	1.9
VVTU6970_at	TC57624	Q1SZ16 Protein kinase related cluster	1.9
VVTU5673_at	GSVIVP00024946001	Q6ZDY8 Succinate dehydrogenase [ubiquinone] flavoprotein subunit, mitochondrial precursor related cluster	1.9
VVTU16208_at	GSVIVP00001496001	Q9M2I9 Hypothetical protein F9D24.180 related cluster	1.9
VVTU11931_at	GSVIVP00020593001	Q8LB02 Succinate dehydrogenase [ubiquinone] iron-sulfur protein 2, mitochondrial precursor related cluster	1.9
VVTU35584_s_at	GSVIVP00017878001	Q84XG6 Erwinia induced protein 2 related cluster	1.9
VVTU36047_at	TC59698	Q1SD84 Integrase, catalytic region related cluster	1.9
VVTU3678_s_at	GSVIVP00019334001	Q8GXJ5 Hypothetical protein related cluster	1.9
VVTU5238_at	GSVIVP00001356001	Q9SR96 Putative DnaJ protein related cluster	1.9
VVTU23815_at	GSVIVP00024880001	Q534U6 SAMT related cluster	1.9
VVTU4249_at	GSVIVP00026876001	Q2HTK4 Protein kinase; U box related cluster	1.9
VVTU16015_at	GSVIVP00020756001	Q67YT8 MRNA, complete cds, clone: RAFL24-09-C04 related cluster	1.9
VVTU13141_at	GSVIVP00015763001	Q700B9 MYC transcription factor related cluster	1.9
VVTU13787_at	GSVIVP00026135001	Q1SFJ9 Leucine zipper, Homeobox-associated; homeodomain-related related cluster	1.9
VVTU39324_at	TC58373	Q8RU52 Putative copia-like retrotransposon Hopscotch Polyprotein related cluster	1.9
VVTU12168_s_at	GSVIVP00018415001	P13905 elongation factor 1-alpha related cluster	1.9
VVTU35532_at	GSVIVP00035334001	Q6E438 ACT11D09.4 related cluster	1.9
VVTU14205_at	GSVIVP00030766001	Q1SDM5 Zinc finger, RING-type; RINGv related cluster	1.9
VVTU26302_at	CF605782	P91374 60S ribosomal protein L15 related cluster	1.9
VVTU31786_at	CB344886	P56774 cytochrome b6-f complex subunit 4 related cluster	1.9
VVTU1390_s_at	GSVIVP00013080001	Q3HVL7 TSJT1-like protein related cluster	1.9
VVTU2671_at	GSVIVP00010116001	AF274281 Vitis vinifera resveratrol synthase (RS1) mRNA, complete cds.	1.9
VVTU31820_at	CB344665	Q1T5X1 Hypothetical protein related cluster	1.9
VVTU36650_at	TC57350	Q8HD79 Orf5 protein related cluster	1.9
VVTU18172_at	VVTU18172_at	Q9M4H3 Putative Metallothionein-like protein related cluster	1.9
VVTU14420_at	GSVIVP00027264001	Q9SZ19 Putative APG protein related cluster	1.9
VVTU18108_at	GSVIVP00024648001	Q8GTD6 polygalacturonase inhibitor-like protein related cluster	1.9
VVTU35544_at	GSVIVP00021538001	Q1ZZ69 Secoisolariciresinol dehydrogenase related cluster	1.9
VVTU5769_at	GSVIVP00020777001	Q0WN35 Hypothetical protein At3g48030 related cluster	1.9
VVTU25069_at	GSVIVP00011578001	Q0IZV3 Os09g0547200 protein related cluster	1.9
VVTU37061_at	GSVIVP00026196001	Q3ED30 Protein At1g33060 related cluster	1.9
VVTU39568_at	GSVIVP00001592001	Q9LZ27 Hypothetical protein T1E3_60 related cluster	1.9
VVTU14035_at	GSVIVP00000341001	Q5VNK5 Hypothetical protein B1109A06.18 related cluster	1.9
VVTU24090_at	GSVIVP00033262001	Q1RWV0 Ankyrin related cluster	1.9
VVTU6288_at	GSVIVP00022846001	Q84MC0 Putative GPI-anchored protein At3g06035 precursor related cluster	1.9

Probe set	Unique Gene ID	Annotation	Fold-change
VVTU22155_at	GSVIVP00009530001	Q9FGM5 Gb|AAF25996.1 related cluster	1.9
VVTU7114_at	GSVIVP00032375001	Q10LK1 Desiccation-related protein PCC13-62, putative, expressed related cluster	1.9
VVTU7113_at	GSVIVP00009273001	Q4R1I9 Anthocyanidin 5,3-O-Glucosyltransferase related cluster	1.9
VVTU11005_at	GSVIVP00020022001	Q9LJ86 Gb|AAD48513.1 related cluster	1.9
VVTU40199_at	GSVIVP00023330001	Q4R0I0 Pinoresinol-lariciresinol reductase related cluster	1.9
VVTU17962_s_at	GSVIVP00030537001	Q2HTL2 Hypothetical protein related cluster	1.9
VVTU519_at	VVTU519_at	Q1SU64 Plant lipid transfer protein Par allergen related cluster	1.9
VVTU16052_at	GSVIVP00035869001	Q8S8E3 Expressed protein related cluster	1.9
VVTU23568_at	GSVIVP00014579001	Q8LLJ2 ADP-glucose pyrophosphorylase large subunit related cluster	1.9
VVTU32810_at	GSVIVP00011801001	Q53M53 Hemolysin A, putative related cluster	1.9
VVTU3605_at	GSVIVP00017935001	Q7XYY0 AKIN gamma related cluster	1.9
VVTU7201_at	CD801412	Q75IK2 Hypothetical protein OSJNBb0016G07.10 related cluster	-1.9
VVTU32990_x_at	CB341370	P62577 Chloramphenicol acetyltransferase related cluster	-1.9
VVTU29720_at	GSVIVP00023198001	Q54PM6 Hypothetical protein related cluster	-1.9
VVTU10054_at	GSVIVP00032978001	Q0E0Q2 Os02g0530800 protein related cluster	-1.9
VVTU1122_at	GSVIVP00008668001	Q3ED49 Protein At1g29980 related cluster	-1.9
VVTU16916_s_at	TC70718	Q0W9E8 Putative stress-induced protein related cluster	-1.9
VVTU3642_s_at	GSVIVP00033507001	Q1SF97 RHO protein GDP dissociation inhibitor related cluster	-1.9
VVTU5837_at	GSVIVP00016348001	UPI000034EFAB Cluster related to UPI000034EFAB; guanyl-nucleotide exchange factor	-1.9
VVTU8305_at	GSVIVP00021649001	Q9LZS6 Cucumisin-like protein related cluster	-1.9
VVTU39443_at	GSVIVP00018142001	Q93YU5 Probable exocyst complex component 4 related cluster	-1.9
VVTU7149_at	GSVIVP00031783001	UPI0000447264 Cluster related to UPI0000447264; PREDICTED: similar to Histone protein Hist2h3c1	-1.9
VVTU3072_at	GSVIVP00015539001	Q93ZJ4 At1g76160 T23E18_10 related cluster	-1.9
VVTU10730_at	GSVIVP00036746001	Q8H241 Hypothetical protein related cluster	-1.9
VVTU35635_at	GSVIVP00021203001	Q96519 peroxidase 11 precursor related cluster	-1.9
VVTU7825_at	GSVIVP00000104001	Q9FJJ2 Dehydrogenase related cluster	-1.9
VVTU15632_at	GSVIVP00028855001	Q5VRG7 Putative RING finger 1 related cluster	-1.9
VVTU20618_at	VVTU20618_at	Q5VQJ1 Hypothetical protein P0691E06.14 related cluster	-1.9
VVTU24475_at	DT012285	Q1S5C0 Polynucleotidyl transferase, Ribonuclease H fold related cluster	-1.9
VVTU15962_at	GSVIVP00004558001	Q1S8C2 Armadillo related cluster	-1.9
VVTU20668_s_at	GSVIVP00038507001	Q9LVB6 Similarity to Embryo-specific protein 3 related cluster	-1.9
VVTU2819_at	GSVIVP00019323001	Q9LM69 RING-H2 finger protein ATL1B related cluster	-1.9
VVTU9373_at	GSVIVP00037398001	O80918 Hypothetical protein At2g38330 related cluster	-1.9
VVTU35247_at	TC67561	Q1SA49 Response regulator receiver related cluster	-1.9
VVTU13070_at	GSVIVP00031348001	Q9ZTY8 Glucose acyltransferase related cluster	-1.9
VVTU14131_at	GSVIVP00026315001	Q8VYD9 Hypothetical protein At4g31010 related cluster	-1.9
VVTU32871_at	CB344034	Q1RUP9 Ferredoxin [2Fe-2S], plant related cluster	-1.9
VVTU2291_s_at	GSVIVP00035855001	Q1SMM2 Protein kinase; ribosomal protein S2 related cluster	-1.9
VVTU22047_s_at	GSVIVP00000681001	Q9FFZ0 Similarity to unknown protein related cluster	-1.9
VVTU15291_s_at	GSVIVP00028378001	Q6RC06 serine threonine protein kinase related cluster	-1.9
VVTU4613_at	GSVIVP00037163001	AF239740 Vitis vinifera caffeic acid O-methyltransferase mRNA, complete cds.	-1.9
VVTU7623_at	GSVIVP00030538001	AF378125 Vitis vinifera GAI-like protein 1 (GAI1) gene, complete cds.	-1.9
VVTU37736_at	GSVIVP00015837001	Q9FJQ9 Emb|CAB87688.1 related cluster	-1.9
VVTU17006_at	GSVIVP00006031001	Q941D6 AT4g33470 F17M5_230 related cluster	-1.9
VVTU7208_at	GSVIVP00032511001	Q4VT47 RD22-like protein related cluster	-1.9
VVTU16585_at	TC64107	Q1SGG3 RNA-directed DNA polymerase(Reverse transcriptase); Endonuclease exonuclease phosphatase related cluster	-1.9
VVTU11096_at	GSVIVP00015655001	Q1S3L9 TRNA pseudouridine synthase related cluster	-1.9
VVTU25666_at	CN007392	Q1SJP8 D-galactoside L-rhamnose binding SUEL lectin; Integrase, catalytic region; Galactose-binding like; peptidase aspartic, catalytic related cluster	-1.9
VVTU3228_at	GSVIVP00025109001	Q8GZA4 Hypothetical protein At1g65320 T8F5_10 related cluster	-1.9
VVTU3851_at	GSVIVP00002467001	Q84TH1 Hypothetical protein At4g23760 related cluster	-1.9
VVTU19938_at	GSVIVP00020962001	Q49RB3 Gip1-like protein related cluster	-1.9
VVTU14334_at	GSVIVP00026217001	Q9ZNU6 Light-mediated development protein DET1 related cluster	-1.9
VVTU422_at	GSVIVP00025894001	Q27JA2 dirigent-like protein pDIR10 related cluster	-1.9
VVTU4290_at	GSVIVP00035401001	Q9LTX5 Similarity to alpha beta hydrolase related cluster	-1.9
VVTU14960_at	TC68504	Q1KUN0 Hypothetical protein related cluster	-1.9
VVTU17023_s_at	GSVIVP00026057001	Q70XZ2 Photosystem I assembly protein ycf4 related cluster	-1.9
VVTU4335_at	GSVIVP00003721001	Q6NMI3 At1g31780 related cluster	-1.9
VVTU11236_at	VVTU11236_at	O81754 Hypothetical protein F17I5.50 related cluster	-1.9
VVTU27751_at	GSVIVP00030154001	Q68EC5 ARE1-like protein P2 related cluster	-1.9
VVTU3258_at	GSVIVP00001483001	Q8SBC8 transcription factor LIM related cluster	-1.9
VVTU6744_at	GSVIVP00016595001	Q1SD65 Tetratricopeptide-like helical related cluster	-1.9
VVTU34465_at	GSVIVP00014736001	Q1S2L8 Transcriptional factor B3; Cupredoxin; TonB box, N-terminal related cluster	-1.9
VVTU16616_at	GSVIVP00032824001	Q8S9J6 AT5g10770 T30N20_40 related cluster	-1.9
VVTU15677_at	CN548040	Q67WQ6 Putative Senescence-associated protein 5 related cluster	-1.9
VVTU9215_at	GSVIVP00031618001	Q5CCQ1 Beta-D-galactosidase related cluster	-1.9
VVTU732_at	GSVIVP00031334001	O22613 Plasma membrane proton ATPase related cluster	-1.9
VVTU21386_at	GSVIVP00028906001	O65840 IAA amidohydrolase related cluster	-1.9
VVTU14126_at	GSVIVP00017855001	Q93X83 HSP70 interActinG protein Thioredoxin chimera related cluster	-1.9
VVTU7420_at	GSVIVP00026978001	O82340 Hypothetical protein At2g46300 related cluster	-1.9
VVTU7805_at	GSVIVP00009334001	Q8L7S6 At1g65600 F5I14_13 related cluster	-1.9
VVTU28304_s_at	GSVIVP00000864001	Q6ZHE3 Putative cyclic nucleotide-binding transporter 1 related cluster	-1.9
VVTU34467_at	GSVIVP00002017001	Q1T152 IMP dehydrogenase GMP reductase related cluster	-1.9
VVTU10307_x_at	GSVIVP00006738001	Q1SI69 Harpin-induced 1 related cluster	-1.9

Probe set	Unique Gene ID	Annotation	Fold-change
VVTU36122_at	GSVIVP00036077001	Q1SF95 Amidase related cluster	-1.9
VVTU3586_at	GSVIVP00013352001	Q9SZ83 Probable oxidoreductase At4g09670 related cluster	-1.9
VVTU26973_at	GSVIVP00015080001	Q6IMT1 SAB related cluster	-1.9
VVTU12619_at	GSVIVP00020743001	Q84WP5 Hypothetical protein At2g36330 related cluster	-1.9
VVTU8927_at	GSVIVP00014909001	O04388 A-type Cyclin related cluster	-1.9
VVTU20599_s_at	GSVIVP00008351001	Q9LDC8 F28G4.6 protein related cluster	-1.9
VVTU38699_at	TC55761	Q60D59 Polyprotein, putative related cluster	-1.9
VVTU8189_at	GSVIVP00023266001	Q2R3G8 serine carboxypeptidase family protein, expressed related cluster	-1.9
VVTU8588_at	GSVIVP00000387001	Q9C8T4 Hypothetical protein F9N12.8 related cluster	-1.9
VVTU34458_at	GSVIVP00016179001	Q9FJI6 Similarity to transportin-SR related cluster	-1.9
VVTU23048_s_at	GSVIVP00001895001	Q5ZF82 Hypothetical protein related cluster	-1.9
VVTU3198_at	GSVIVP00005660001	Q8H0U2 Hypothetical protein At2g12400 related cluster	-1.9
VVTU15898_at	GSVIVP00016168001	Q8W4J5 Putative GDP-mannose pyrophosphorylase related cluster	-1.9
VVTU8335_s_at	GSVIVP00023655001	P08281 Glutamine synthetase leaf isozyme, chloroplast precursor related cluster	-1.9
VVTU13015_s_at	GSVIVP00038286001	Q9C8W5 Hypothetical protein F17M19.12 related cluster	-1.9
VVTU19513_at	GSVIVP00018728001	Q9SIN1 Expressed protein related cluster	-1.9
VVTU22544_s_at	VVTU22544_s_at	Q0JIY4 Os01g0770800 protein related cluster	-1.9
VVTU35249_s_at	GSVIVP00016581001	Q9ZUN5 Putative Senescence-associated protein 5 related cluster	-1.9
VVTU3242_at	GSVIVP00012516001	Q9SZJ2 Hypothetical protein F20D10.20 related cluster	-1.9
VVTU20234_at	GSVIVP00033131001	Q1T0F2 PAPA-1-like conserved region; Zinc finger, HIT-type related cluster	-1.9
VVTU25312_at	CO819619	Q7XNY0 OSJNBb0015N08.11 protein related cluster	-1.9
VVTU3657_at	GSVIVP00036476001	AY226829 Vitis vinifera cytochrome P-450-like protein (CYP82) mRNA, partial cds.	-1.9
VVTU16253_at	GSVIVP00028114001	Q9LZQ1 Hypothetical protein T12C14_90 related cluster	-1.9
VVTU23317_at	TC52059	Q1RTD2 Hypothetical protein related cluster	-1.9
VVTU10962_at	VVTU10962_at	Q6T2Z3 Cyclin-dependent kinase inhibitor 1;1 related cluster	-1.9
VVTU40745_at	GSVIVP00008646001	Q1SJ09 WD-40 repeat related cluster	-1.9
VVTU14517_at	TC58309	Q10E81 ribosomal protein S14p S29e containing protein, expressed related cluster	-1.9
VVTU1455_s_at	TC62029	Q0JHY5 Os01g0835900 protein related cluster	-1.9
VVTU31582_at	CB341255	Q9LN35 F18O14.35 related cluster	-1.9
VVTU21904_s_at	GSVIVP00033380001	Q8VZB8 Hypothetical protein At5g55810; MDF20.25 related cluster	-1.9
VVTU7757_at	GSVIVP00031650001	Q6TF29 Rapid alkalinization factor 1 related cluster	-1.9
VVTU9766_at	GSVIVP00021927001	Q3ECC6 Protein At1g76680 related cluster	-1.9
VVTU15316_at	TC66005	Q9SRX5 F22D16.19 protein related cluster	-1.9
VVTU25813_at	GSVIVP00024124001	Q8LF75 Putative receptor ser thr protein kinase related cluster	-1.9
VVTU21870_at	GSVIVP00026789001	Q9LLY3 LIM domain protein PLIM-2 related cluster	-1.9
VVTU29209_at	GSVIVP00024922001	Q8LC79 GATA transcription factor 19 related cluster	-1.9
VVTU18420_x_at	GSVIVP00032717001	Q94AW5 AT5g25190 F21J6_103 related cluster	-1.9
VVTU15746_s_at	GSVIVP00022579001	Q96416 Cyclophylin related cluster	-1.9
VVTU1225_at	TC69112	O64835 Expressed protein related cluster	-1.9
VVTU1319_s_at	GSVIVP00032295001	Q1SQL3 Ctr copper transporter related cluster	-1.9
VVTU38342_at	GSVIVP00021472001	Q9ZQW5 Laccase related cluster	-1.9
VVTU23595_at	DT038142	O23757 Reverse transcriptase related cluster	-1.9
VVTU34013_at	CB001384	Q7M450 Cryptogene protein G1 related cluster	-1.9
VVTU35589_s_at	GSVIVP00028341001	Q93VD3 At1g30270 F12P21_6 related cluster	-1.9
VVTU2068_at	GSVIVP00022959001	Q0W9E8 Putative stress-induced protein related cluster	-1.9
VVTU11988_at	TC67357	Q6T5H5 Alpha-expansin 3 related cluster	-1.9
VVTU4219_at	GSVIVP00020319001	Q9FRU4 Putative nitrate transporter NRT1-3 related cluster	-1.9
VVTU14432_at	GSVIVP00013496001	Q1SF23 Cation transporting ATPase, C-terminal related cluster	-1.9
VVTU9142_at	TC69268	Q9C700 Hypothetical protein T18C15_4 related cluster	-1.9
VVTU39208_at	TC56208	O48921 Cytochrome P450 97B2 related cluster	-1.9
VVTU16701_at	TC70177	Q6I5R7 Hypothetical protein OJ1058_C01.8 related cluster	-1.9
VVTU10717_at	GSVIVP00019414001	Q8W228 Cytochrome P450 related cluster	-1.9
VVTU27891_at	GSVIVP00024748001	Q9C523 dirigent protein, putative related cluster	-1.9
VVTU36897_s_at	GSVIVP00021429001	Q1S5F3 Zinc finger, C3HC4 type (RING finger), putative related cluster	-1.9
VVTU4273_s_at	GSVIVP00017851001	Q76B42 Hypothetical protein NbPPS3 related cluster	-1.9
VVTU9668_at	GSVIVP00017821001	Q94GS4 Hypothetical protein OSJNBb0022E02.3 related cluster	-1.9
VVTU39138_at	TC57210	Q6L573 Hypothetical protein OJ1008_D08.8 related cluster	-1.9
VVTU3020_at	GSVIVP00018651001	Q9FFH6 Fasciclin-like arabinogalactan protein 13 precursor related cluster	-1.9
VVTU33037_s_at	GSVIVP00017013001	Q6H502 Hypothetical protein OSJNBa0073A21.29 related cluster	-1.9
VVTU22964_at	GSVIVP00023144001	Q1S193 Alpha beta hydrolase fold related cluster	-1.9
VVTU30146_x_at	CF403174	Q9M1P0 Hypothetical protein T18B22.40 related cluster	-1.9
VVTU32632_at	CB349078	Q9C522 ATP citrate lyase, putative; 3734-7120 related cluster	-1.9
VVTU3589_at	GSVIVP00015324001	Q9LLS0 Putative phosphatidylinositol 4-phosphate 5-kinase related cluster	-1.9
VVTU12187_at	GSVIVP00021025001	Q2HW94 Blue (Type 1) copper domain related cluster	-1.9
VVTU25313_at	GSVIVP00006202001	Q851Z3 Hypothetical protein OSJNBa0015N08.8 related cluster	-1.9
VVTU31806_at	CB344768	UPI0000DB7FA7 Cluster related to UPI0000DB7FA7; PREDICTED: similar to Chromodomain helicase-DNA-bindingG protein Mi-2 homolog (ATP-dependent helicase Mi-2) (dMi-2)	-1.9
VVTU35450_at	GSVIVP00000596001	O48818 expansin-A4 precursor related cluster	-1.9
VVTU23469_at	GSVIVP00032914001	Q9M839 T27C4.13 protein related cluster	-1.9
VVTU40748_at	GSVIVP00009534001	Q8L7W2 Nudix hydrolase 8 related cluster	-1.9
VVTU24863_at	GSVIVP00037711001	Q4P6W4 Hypothetical protein related cluster	-1.9
VVTU621_at	GSVIVP00031931001	Q0E3L1 Os02g0168400 protein related cluster	-1.9
VVTU5264_s_at	GSVIVP00013985001	Q1SZU4 Protein kinase PKN PRK1, effector related cluster	-2.0
VVTU3041_s_at	GSVIVP00019399001	Q1S9B5 Glycoside hydrolase, family 77 related cluster	-2.0
VVTU14838_at	GSVIVP00003612001	Q3LRV4 Nitrilase 4B related cluster	-2.0
VVTU27998_at	GSVIVP00000456001	UPI00000A2CBF Cluster related to UPI00000A2CBF; P0408G07.7	-2.0

Probe set	Unique Gene ID	Annotation	Fold-change
VVTU6824_at	GSVIVP00028748001	Q2QDF7 24-sterol C-methyltransferase related cluster	-2.0
VVTU18566_s_at	GSVIVP00038458001	Q1S8T9 Protein kinase related cluster	-2.0
VVTU5349_at	CF512048	Q9LYR0 Hypothetical protein T22N19_110 related cluster	-2.0
VVTU11276_at	GSVIVP00013878001	Q9FGC1 Protein kinase-like related cluster	-2.0
VVTU13003_at	GSVIVP00000042001	Q2QN86 Expressed protein related cluster	-2.0
VVTU13809_at	GSVIVP00011743001	AF236127 Vitis vinifera catalase (GCat) mRNA, complete cds.	-2.0
VVTU3409_at	GSVIVP00001062001	Q9LE33 T12C24.9 related cluster	-2.0
VVTU12492_at	GSVIVP00022617001	AF192308 Vitis vinifera H+-pyrophosphatase mRNA, partial cds.	-2.0
VVTU20359_at	GSVIVP00006183001	Q2HUL8 Aspartate kinase region related cluster	-2.0
VVTU9002_s_at	GSVIVP00030121001	P93470 Cholinephosphate cytidylyltransferase related cluster	-2.0
VVTU10447_at	GSVIVP00025489001	Q6NMJ9 At2g23840 related cluster	-2.0
VVTU23798_at	DT033962	Q2QM06 Retrotransposon protein, putative, Ty3-gypsy subclass related cluster	-2.0
VVTU12222_at	GSVIVP00002785001	Q7XLP1 OSJNBa0044M19.15 protein related cluster	-2.0
VVTU35915_x_at	GSVIVP00037593001	Q94FS9 Gamma-aminobutyrate transaminase subunit precursor related cluster	-2.0
VVTU13197_at	GSVIVP00034909001	Q8LB81 Putative GDSL-motif lipaseacylhydrolase related cluster	-2.0
VVTU4709_at	GSVIVP00035871001	Q9LP48 F28N24.12 protein related cluster	-2.0
VVTU5210_at	GSVIVP00017088001	Q9M0B9 Hypothetical protein AT4g30410 related cluster	-2.0
VVTU4551_at	GSVIVP00034342001	Q1SP67 Pathogenesis-related transcriptional factor and ERF related cluster	-2.0
VVTU8213_at	GSVIVP00016423001	Q8LCS3 Hypothetical protein related cluster	-2.0
VVTU32056_s_at	GSVIVP00000537001	Q6NLD7 At1g64640 related cluster	-2.0
VVTU4131_s_at	GSVIVP00026137001	Q1SHH7 auxin responsive SAUR protein related cluster	-2.0
VVTU1891_at	GSVIVP00010262001	Q8S459 Putative sphingolipid delta 4 desaturase DES-1 related cluster	-2.0
VVTU19699_at	GSVIVP00032637001	Q56XG6 DEAD-box ATP-dependent RNA helicase 15 related cluster	-2.0
VVTU25952_at	GSVIVP00026851001	Q5XNL4 Resistance protein-like protein related cluster	-2.0
VVTU8192_at	GSVIVP00037375001	UPI000034F013 Cluster related to UPI000034F013; bile acid:sodium symporter	-2.0
VVTU1273_at	GSVIVP00002688001	Q40161 Polygalacturonase-1 non-catalytic subunit beta precursor related cluster	-2.0
VVTU24096_at	GSVIVP00025523001	Q9M2R4 receptor-like protein kinase related cluster	-2.0
VVTU11861_s_at	GSVIVP00000605001	DQ834702 Vitis vinifera putative aquaPorin (TIP1;2) mRNA, complete cds.	-2.0
VVTU35813_at	GSVIVP00032018001	Q8VXE0 Glyceraldehyde-3-phosphate dehydrogenase related cluster	-2.0
VVTU23541_at	DT039349	Q9ZQ09 Putative Ty3-gypsy-like retroelement pol Polyprotein related cluster	-2.0
VVTU16007_at	GSVIVP00034523001	Q9M9E1 pleiotropic drug Resistance protein 12 related cluster	-2.0
VVTU35786_at	GSVIVP00010782001	Q1RWV8 IMP dehydrogenase GMP reductase related cluster	-2.0
VVTU33777_s_at	GSVIVP00037004001	Q5ZGH7 Hypothetical protein related cluster	-2.0
VVTU8273_at	GSVIVP00026716001	Q8LFH3 Hypothetical protein related cluster	-2.0
VVTU7336_at	GSVIVP00021651001	Q9LZS7 Hypothetical protein F17C15_30 related cluster	-2.0
VVTU19427_at	CF206592	Q9SF35 40S ribosomal protein S23-1 related cluster	-2.0
VVTU16329_at	GSVIVP00032704001	Q9S775 CHD3-type chromatin remodeling factor PICKLE related cluster	-2.0
VVTU16152_at	GSVIVP00037963001	Q8RWC3 Hypothetical protein At3g19340 related cluster	-2.0
VVTU10788_at	GSVIVP00034691001	O81155 Cysteine synthase, chloroplast precursor (EC 2.5.1.47) (O-acetylserine sulfhydrylase) (O-acetylserine (Thiol)-lyase) related cluster	-2.0
VVTU12912_at	GSVIVP00002441001	Q9SUP5 Putative polygalacturonase related cluster	-2.0
VVTU31704_at	CB345328	Q0ZIW0 Hypothetical chloroplast RF1 related cluster	-2.0
VVTU15369_at	GSVIVP00035100001	Q9LU93 Mitotic spindle checkpoint protein MAD2 related cluster	-2.0
VVTU17863_s_at	GSVIVP00026952001	Q43469 Delta-8 sphingolipid desaturase related cluster	-2.0
VVTU25410_s_at	GSVIVP00036466001	Q6XL72 cytochrome P-450-like protein related cluster	-2.0
VVTU15662_at	GSVIVP00013498001	Q1ENZ8 Hypothetical protein related cluster	-2.0
VVTU10920_at	GSVIVP00033011001	Q9LUR8 Arabidopsis thaliana genomic DNA, chromosome 3, P1 clone: MGL6 related cluster	-2.0
VVTU7325_at	GSVIVP00024955001	Q1S4F5 Hypothetical protein related cluster	-2.0
VVTU9538_at	GSVIVP00025938001	Q4F8J0 Putative endo-1,4-beta-Glucanase related cluster	-2.0
VVTU7674_at	GSVIVP00020927001	Q8GW32 Hypothetical protein related cluster	-2.0
VVTU22981_at	GSVIVP00035375001	Q1SKS5 SAM (And some other nucleotide) binding motif related cluster	-2.0
VVTU13881_s_at	GSVIVP00018467001	Q08707 BPF-1 protein related cluster	-2.0
VVTU15610_s_at	GSVIVP00018222001	Q9SFB5 Putative serine carboxypeptidase II related cluster	-2.0
VVTU39424_at	GSVIVP00027058001	Q96337 AMP-bindinG protein related cluster	-2.0
VVTU7057_at	GSVIVP00019337001	O04892 cytochrome P450 like_TBP related cluster	-2.0
VVTU2835_at	GSVIVP00007419001	Q6EP75 Hypothetical protein P0135D07.45 related cluster	-2.0
VVTU34941_s_at	GSVIVP00023841001	Q0PH24 auxin-regulated protein related cluster	-2.0
VVTU522_at	VVTU522_at	Q2IB39 cellulose synthase 5 related cluster	-2.0
VVTU29968_at	GSVIVP00034361001	Q9M817 Peptide transporter, putative related cluster	-2.0
VVTU34835_at	GSVIVP00017822001	Q9AXH3 Phosphoethanolamine N-methyltransferase related cluster	-2.0
VVTU21624_at	CD799281	Q2TE76 Coat protein related cluster	-2.0
VVTU37678_s_at	GSVIVP00028896001	Q8GVE0 Cyclin D1 related cluster	-2.0
VVTU40323_at	GSVIVP00017134001	Q9SUD1 Hypothetical protein T13J8.190 related cluster	-2.0
VVTU40684_s_at	GSVIVP00023064001	Q3EAK4 Protein At3g53470 related cluster	-2.0
VVTU7771_at	GSVIVP00019602001	Q6WNQ8 CYP81E8 related cluster	-2.0
VVTU18914_s_at	GSVIVP00023976001	O22926 Hypothetical protein At2g30300 related cluster	-2.0
VVTU7328_at	GSVIVP00010064001	O23689 Hypothetical protein T19D16.22 related cluster	-2.0
VVTU16009_s_at	GSVIVP00016623001	Q1SEB0 Zinc finger, CCCH-type related cluster	-2.0
VVTU2895_s_at	TC67270	Q75LJ0 Expressed protein related cluster	-2.0
VVTU10597_at	GSVIVP00028929001	Q9LFX1 T7N9.27 related cluster	-2.0
VVTU27526_at	CF403553	Q5K4K6 Cytochrome P450-like protein related cluster	-2.0
VVTU14228_at	GSVIVP00032485001	Q4VT47 RD22-like protein related cluster	-2.0
VVTU15434_at	GSVIVP00025647001	O22216 Glycerol-3-phosphate dehydrogenase related cluster	-2.0
VVTU37806_s_at	TC55630	Q9FPH6 AT3g15840 related cluster	-2.0
VVTU13629_x_at	GSVIVP00028191001	P07370 chlorophyll a-b bindinG protein 1B, chloroplast precursor related cluster	-2.0

Probe set	Unique Gene ID	Annotation	Fold-change
VVTU38049_at	GSVIVP00035710001	Q9SA48 F3O9.30 related cluster	-2.0
VVTU37286_at	TC65522	Q9SUZ4 Hypothetical protein F4F15.220 related cluster	-2.0
VVTU25265_at	CX016363	Q09MD9 ribosomal protein S3 related cluster	-2.0
VVTU5134_at	GSVIVP00026915001	Q6ID84 At2g46100 related cluster	-2.0
VVTU19282_s_at	DT039600	Q8LBL4 Putative Thaumatin-like protein related cluster	-2.0
VVTU11425_at	GSVIVP00038210001	Q9FF17 Probable dolichyl pyrophosphate Man9GlcNAc2 alpha-1,3- Glucosyltransferase related cluster	-2.0
VVTU13611_at	GSVIVP00014847001	Q6Z107 Putative receptor protein kinase PERK1 related cluster	-2.0
VVTU38571_at	GSVIVP00027638001	Q2HV43 Flavoprotein pyridine nucleotide cytochrome reductase; Flavodoxin nitric oxide synthase related cluster	-2.0
VVTU15231_s_at	GSVIVP00024344001	Q94B55 Putative RING Zinc finger Ankyrin protein related cluster	-2.0
VVTU27242_at	GSVIVP00020384001	Q1S897 helicase, C-terminal related cluster	-2.0
VVTU24082_at	GSVIVP00030732001	Q944N6 Cytochrome P450 related cluster	-2.1
VVTU25871_at	GSVIVP00016213001	Q9FNQ1 RNA helicase related cluster	-2.1
VVTU27714_at	GSVIVP00030301001	Q1SM29 Amino acid polyamine transporter II related cluster	-2.1
VVTU29470_at	CB982300	Q38945 PAP-specific phosphatase HAL2-like (3 (2),5 -bisphosphate nucleotidase) (EC 3.1.3.7) (3 (2),5-bisphosphonucleoside 3 (2)- phosphohydrolase) related cluster	-2.1
VVTU20744_at	VVTU20744_at	Q9LUB8 polygalacturonase related cluster	-2.1
VVTU35014_at	GSVIVP00032341001	Q9SDM0 Vacuolar H+-ATPase catalytic subunit related cluster	-2.1
VVTU15086_at	GSVIVP00024483001	Q6L3H5 Translation elongation factor, putative related cluster	-2.1
VVTU10016_at	GSVIVP00019675001	UPI0000197181 Cluster related to UPI0000197181; unknown protein	-2.1
VVTU22040_at	GSVIVP00008869001	Q8W418 lipoxygenase related cluster	-2.1
VVTU26554_at	GSVIVP00007133001	Q1S1I5 SART-1 protein related cluster	-2.1
VVTU5010_at	GSVIVP00036093001	Q8L883 auxin transporter-like protein 5 related cluster	-2.1
VVTU37707_at	GSVIVP00007214001	Q9FK76 Subtilisin-like protease related cluster	-2.1
VVTU3136_at	GSVIVP00025268001	Q1SGN7 D-isomer specific 2-hydroxyacid dehydrogenase, catalytic region related cluster	-2.1
VVTU8036_at	GSVIVP00025590001	Q2QDF7 24-sterol C-methyltransferase related cluster	-2.1
VVTU15167_at	GSVIVP00015839001	Q336T1 Oxidoreductase, 2OG-Fe oxygenase family protein, expressed related cluster	-2.1
VVTU11949_s_at	GSVIVP00035377001	Q5HZ48 At5g49800 related cluster	-2.1
VVTU33072_at	GSVIVP00027933001	Q6L974 GAG-POL related cluster	-2.1
VVTU8529_at	GSVIVP00034537001	Q9XI20 F9L1.44 protein related cluster	-2.1
VVTU12677_at	GSVIVP00026380001	Q9LZZ6 Hypothetical protein T4C21_80 related cluster	-2.1
VVTU37316_at	VVTU37316_at	Q51613 , complete genome related cluster	-2.1
VVTU4619_at	GSVIVP00019299001	Q5CCQ0 Beta-D-galactosidase related cluster	-2.1
VVTU9165_x_at	TC62046	Q9LKW2 Hypothetical protein related cluster	-2.1
VVTU13248_at	TC60412	Q9LXK1 Hypothetical protein F3C22_30 related cluster	-2.1
VVTU21507_s_at	GSVIVP00009138001	Q9FF84 ATP-dependent RNA helicase A-like protein related cluster	-2.1
VVTU9111_at	GSVIVP00019041001	Q40220 Rac-like GTP-bindinG protein RAC2 related cluster	-2.1
VVTU15303_at	GSVIVP00035896001	Q8L7S5 AT4g18560 F28J12_220 related cluster	-2.1
VVTU2752_s_at	GSVIVP00020103001	Q9LIJ8 Arabidopsis thaliana genomic DNA, chromosome 3, BAC clone:F5N5 related cluster	-2.1
VVTU10874_at	GSVIVP00008702001	Q1SRB6 Hypothetical protein related cluster	-2.1
VVTU20622_at	GSVIVP00027904001	Q84N43 Hypothetical protein related cluster	-2.1
VVTU13386_at	TC68882	Q8L9K4 Hypothetical protein related cluster	-2.1
VVTU2344_s_at	TC60078	Q8W0W5 Repressor protein related cluster	-2.1
VVTU14714_s_at	TC63866	Q9XED8 auxin response factor 9 related cluster	-2.1
VVTU22073_at	GSVIVP00035217001	Q1SS95 E-class P450, group I related cluster	-2.1
VVTU23533_s_at	GSVIVP00003705001	Q6ZL19 Putative carboxyl-terminal proteinase related cluster	-2.1
VVTU40141_at	GSVIVP00008763001	Q9FPN1 Putative Cytochrome P450 related cluster	-2.1
VVTU35758_at	GSVIVP00006044001	Q6ZHE5 Putative3-hydroxy-3-methylglutaryl deaminase related cluster	-2.1
VVTU12052_at	GSVIVP00030103001	Q8RX58 At2g34960 F19I3.19 related cluster	-2.1
VVTU10150_at	GSVIVP00016982001	Q1S7A9 Protein prenyltransferase related cluster	-2.1
VVTU25425_x_at	CF202745	Q5M9R1 Hypothetical protein orf138c related cluster	-2.1
VVTU23291_at	GSVIVP00024954001	Q5C996 SWI2 SNF2-like protein related cluster	-2.1
VVTU28196_at	CF207175	Q7XBD8 Putative retrotransposon RIRE1 poly protein related cluster	-2.1
VVTU32858_at	CB344325	Q1XKL4 Hypothetical protein related cluster	-2.1
VVTU12123_at	GSVIVP00024173001	AY233207 Vitis vinifera putative Photosystem I reaction center subunit N precursor (PsaN) mRNA, partial cds; nuclear gene for chloroplast product.	-2.1
VVTU39711_at	GSVIVP00027802001	Q9MAH1 F12M16.20 related cluster	-2.1
VVTU4385_at	TC59645	Q304A5 Protein At4g19160 related cluster	-2.1
VVTU4397_at	GSVIVP00014506001	Q58IJ2 MADS box protein related cluster	-2.1
VVTU37195_at	GSVIVP00000743001	Q9M3I0 Putative Glucosyltransferase related cluster	-2.1
VVTU33624_x_at	GSVIVP00036712001	Q9M4H3 Putative Metallothionein-like protein related cluster	-2.1
VVTU488_at	GSVIVP00001813001	Q1T171 Dienelactone hydrolase related cluster	-2.1
VVTU36057_at	GSVIVP00006015001	Q0KIP5 Polyprotein, putative related cluster	-2.1
VVTU12483_at	GSVIVP00029573001	Q94K16 Hypothetical protein F21O3.22 related cluster	-2.1
VVTU7013_at	GSVIVP00034270001	Q1SAY6 Lipolytic enzyme, G-D-S-L related cluster	-2.1
VVTU15149_at	GSVIVP00014274001	Q9LHP4 receptor protein kinase-like protein related cluster	-2.1
VVTU30293_at	GSVIVP00038386001	Q2MCJ5 Xylan 1,4-beta-xylosidase related cluster	-2.1
VVTU13006_at	GSVIVP00038055001	Q6TXV9 Cysteine proteinase related cluster	-2.1
VVTU33017_at	CA814476	Q7G764 Probable NAD(P)H-dependent oxidoreductase 1 related cluster	-2.1
VVTU15090_at	GSVIVP00024505001	Q2LAI9 auxin response factor 4 related cluster	-2.1
VVTU35333_at	GSVIVP00000495001	Q8L868 Putative glucan endo-1,3-beta-glucosidase 11 precursor (EC 3.2.1.39) ((1->3)-beta-glucan endohydrolase 11) ((1->3)-beta-Glucanase 11) related cluster	-2.1
VVTU6333_at	GSVIVP00002887001	Q9LDZ5 F2D10.13 related cluster	-2.1
VVTU37077_at	GSVIVP00020567001	Q9S7I4 F28J7.8 protein related cluster	-2.1
VVTU14788_at	DT031278	UPI00000A7565 Cluster related to UPI00000A7565; putative DEIH-box RNA DNA helicase	-2.1

Probe set	Unique Gene ID	Annotation	Fold-change
VVTU14933_at	GSVIVP00027943001	Q9SYM9 Hypothetical protein T30F21.14 related cluster	-2.1
VVTU4822_at	GSVIVP00036076001	Q9SW47 Amidase-like protein related cluster	-2.1
VVTU35412_at	GSVIVP00014964001	Q8SBC8 transcription factor LIM related cluster	-2.1
VVTU4106_x_at	TC57059	Q9M4H4 Ripening-related protein grip22 precursor related cluster	-2.2
VVTU12735_s_at	GSVIVP00035586001	P56757 ATP synthase subunit alpha related cluster	-2.2
VVTU16917_at	GSVIVP00024152001	O82392 Putative thiamin biosynthesis protein related cluster	-2.2
VVTU1844_at	GSVIVP00024075001	Q9LUZ6 Arabidopsis thaliana genomic DNA, chromosome 5, P1 clone:MZN1 related cluster	-2.2
VVTU21861_at	GSVIVP00035997001	Q9SW54 Hypothetical protein T11I11.40 related cluster	-2.2
VVTU11947_at	GSVIVP00022077001	Q58A16 Hypothetical protein 7E10 related cluster	-2.2
VVTU6696_at	GSVIVP00025873001	Q1SJL6 Hypothetical protein related cluster	-2.2
VVTU8470_at	CA817147	Q42962 phosphoglycerate kinase, cytosolic related cluster	-2.2
VVTU22796_s_at	GSVIVP00010417001	Q1SF39 Zinc finger, RING-type related cluster	-2.2
VVTU10999_at	GSVIVP00022132001	Q6JJ33 Putative serine carboxipeptidase related cluster	-2.2
VVTU14262_s_at	GSVIVP00023118001	Q9M3C7 Hypothetical protein T26I12.120 related cluster	-2.2
VVTU9073_x_at	GSVIVP00009968001	Q2PEP3 Putative Glucosyltransferase related cluster	-2.2
VVTU3030_s_at	CF202123	Q6F6B7 SlHDL1 related cluster	-2.2
VVTU12815_at	GSVIVP00029240001	Q93Z79 AT5g24910 F6A4_120 related cluster	-2.2
VVTU39089_at	GSVIVP00013780001	Q9ZT72 Hypothetical protein F9H3.6 related cluster	-2.2
VVTU39779_at	GSVIVP00022469001	UPI000034F21F Cluster related to UPI000034F21F; EMB2247; ATP binding tRNA ligase valine-tRNA ligase	-2.2
VVTU11843_at	GSVIVP00015407001	Q9XF61 Protein disulfide-isomerase precursor related cluster	-2.2
VVTU9135_at	GSVIVP00008963001	Q9FE98 Alliinase, putative; 28821-30567 related cluster	-2.2
VVTU7361_at	GSVIVP00021131001	Q9ZQ71 Expressed protein related cluster	-2.2
VVTU8428_at	GSVIVP00034256001	Q9C8I6 receptor protein kinase, putative related cluster	-2.2
VVTU35012_at	GSVIVP00036753001	Q9ATD1 GHMyb9 related cluster	-2.2
VVTU28710_at	GSVIVP00000796001	Q1SLW6 Gag-pol Polyprotein-related related cluster	-2.2
VVTU4751_at	GSVIVP00028647001	Q9LYR0 Hypothetical protein T22N19_110 related cluster	-2.2
VVTU3516_at	GSVIVP00029273001	Q1I1D7 Cytochrome P450 related cluster	-2.2
VVTU2125_at	GSVIVP00017546001	Q0GLE8 Dof4 related cluster	-2.2
VVTU459_x_at	VVTU459_x_at	Q53IB4 Hypothetical protein related cluster	-2.2
VVTU10861_at	GSVIVP00015969001	Q5N869 Hypothetical protein P0456E05.23 related cluster	-2.2
VVTU23354_at	TC57034	Q1SBU3 Hypothetical protein related cluster	-2.2
VVTU15964_s_at	GSVIVP00011908001	Q9SGZ8 F28K19.24 related cluster	-2.2
VVTU5708_at	GSVIVP00038610001	Q1T2M9 PIK-related kinase, FAT; PIK-related kinase, FATC; peptidase M, neutral zinc metallopeptidases, zinc-binding site; FKBP12-rapamycin- associated protein, FKBP12-rapamycin-binding related cluster	-2.2
VVTU14160_s_at	GSVIVP00036132001	Q93XV7 Hydroxypyruvate reductase related cluster	-2.2
VVTU11469_at	GSVIVP00028488001	Q1S805 DENN; dDENN; uDENN related cluster	-2.2
VVTU16934_x_at	GSVIVP00003318001	P46256 Fructose-bisphosphate aldolase, cytoplasmic isozyme 1 related cluster	-2.2
VVTU22586_s_at	GSVIVP00006180001	Q8GWB2 Hypothetical protein At1g28100 F13K9_27 related cluster	-2.2
VVTU5732_at	GSVIVP00000088001	Q2VTE6 HDZip I protein related cluster	-2.2
VVTU17622_at	GSVIVP00023204001	Q41367 24 kDa RNA bindinG protein related cluster	-2.2
VVTU10954_at	GSVIVP00034531001	Q9M9E2 T16N11.2 protein related cluster	-2.2
VVTU21683_at	GSVIVP00022504001	Q1S2G2 homeodomain-related related cluster	-2.2
VVTU25274_at	GSVIVP00034220001	Q1RTQ9 Hypothetical protein related cluster	-2.2
VVTU38290_at	GSVIVP00002784001	Q7X9E3 Hypothetical protein related cluster	-2.2
VVTU21136_s_at	GSVIVP00018632001	Q0IS70 Os11g0552000 protein related cluster	-2.2
VVTU14388_at	GSVIVP00014547001	Q7Y0Z8 Bell-like homeodomain protein 1 related cluster	-2.2
VVTU1850_at	GSVIVP00002112001	Q6YYC5 Copine I-like protein related cluster	-2.2
VVTU25616_at	GSVIVP00016049001	Q1T393 IMP dehydrogenase GMP reductase related cluster	-2.2
VVTU1440_at	GSVIVP00002554001	Q84LI7 Polygalacturonase-like protein related cluster	-2.2
VVTU1278_at	GSVIVP00003076001	Q9ST69 30S ribosomal protein S5, chloroplast precursor related cluster	-2.2
VVTU12986_at	GSVIVP00012585001	Q9SE97 Formin-like protein AHF1 related cluster	-2.2
VVTU22005_s_at	GSVIVP00014281001	Q700J9 Putative Pathogenesis-related protein related cluster	-2.2
VVTU17250_s_at	GSVIVP00020350001	Q1S206 RNA-binding region RNP-1 (RNA recognition motif); Nuclear transport factor 2 related cluster	-2.2
VVTU6544_at	GSVIVP00023771001	Q1SMI4 Sulphate transporter related cluster	-2.3
VVTU3923_at	GSVIVP00036948001	Q9LQX2 T24P13.19 related cluster	-2.3
VVTU6853_at	TC55079	O23026 T1G11.16 protein related cluster	-2.3
VVTU3110_at	GSVIVP00006214001	Q9LYR6 Peptide transporter-like protein related cluster	-2.3
VVTU11076_at	GSVIVP00024231001	Q941T0 Pentatricopeptide (PPR) repeat-containing protein-like related cluster	-2.3
VVTU1214_at	GSVIVP00024749001	Q9C893 Hypothetical protein F7A10.19 related cluster	-2.3
VVTU37412_s_at	GSVIVP00031449001	Q1SZF1 Allergen V5 Tpx-1 related related cluster	-2.3
VVTU34290_at	BQ796927	Q2PIU7 RNA polymerase II transcription initiation nucleotide excision repair factor TFIIH related cluster	-2.3
VVTU3307_at	GSVIVP00009267001	Q4U4M3 Subtilisin-like protease related cluster	-2.3
VVTU38174_at	TC65028	Q688X8 putative Heat shock protein, HSP40 related cluster	-2.3
VVTU23774_at	GSVIVP00015336001	Q9FEL6 auxin transporter-like protein 3 related cluster	-2.3
VVTU40463_at	TC66419	Q1SD84 Integrase, catalytic region related cluster	-2.3
VVTU2642_at	GSVIVP00025336001	Q9M3U4 Beta 1-3 Glucanase related cluster	-2.3
VVTU24560_at	GSVIVP00019508001	O22764 Putative serine threonine protein kinase related cluster	-2.3
VVTU11898_at	GSVIVP00038145001	Q1KUX1 Hypothetical protein related cluster	-2.3
VVTU23718_at	GSVIVP00017414001	Q5Z645 Putative ascorbate oxidase AO4 related cluster	-2.3
VVTU7793_at	GSVIVP00026053001	Q07446 peroxidase precursor related cluster	-2.3

Probe set	Unique Gene ID	Annotation	Fold-change
VVTU2174_at	GSVIVP00024750001	Q9LHC4 Arabidopsis thaliana genomic DNA, chromosome 3, BAC clone: T13O13 related cluster	-2.3
VVTU27812_s_at	GSVIVP00028342001	Q93VD3 At1g30270 F12P21_6 related cluster	-2.3
VVTU37464_at	TC62016	Q43295 Adenylyl-sulfate kinase 1, chloroplast precursor related cluster	-2.3
VVTU31692_at	CB345360	Q02071 Type III chlorophyll a b-bindinG protein precursor related cluster	-2.3
VVTU4293_at	GSVIVP00004278001	Q9LUA9 Zinc finger protein CONSTANS-LIKE 10 related cluster	-2.3
VVTU13433_at	GSVIVP00029885001	Q2VT54 DC1.2-like related cluster	-2.3
VVTU2781_s_at	GSVIVP00002701001	Q5NE21 Carbonic anhydrase related cluster	-2.3
VVTU8282_at	DT021402	Q1SJ61 Zinc finger, RING-type; RINGv related cluster	-2.3
VVTU21080_at	GSVIVP00021858001	O80543 Uncharacterized protein At1g22800 related cluster	-2.3
VVTU14674_at	GSVIVP00036883001	Q9LQ73 T1N6.24 protein related cluster	-2.3
VVTU8926_at	GSVIVP00015159001	P22195 Cationic peroxidase 1 precursor related cluster	-2.3
VVTU22526_at	GSVIVP00037776001	Q8LJ11 Glucosyltransferase-like related cluster	-2.3
VVTU14555_s_at	GSVIVP00013331001	Q8W505 Phosphoenolpyruvate carboxykinase related cluster	-2.3
VVTU22924_s_at	GSVIVP00003869001	O65629 Hypothetical protein T19K4.50 related cluster	-2.3
VVTU538_at	GSVIVP00031153001	Q10D12 Transferase family protein, expressed related cluster	-2.3
VVTU38517_at	TC68248	Q00251 elongation factor 1-alpha related cluster	-2.3
VVTU19819_at	GSVIVP00001983001	Q58L86 strictosidine synthase family protein related cluster	-2.3
VVTU18758_at	GSVIVP00036131001	Q1T416 SKP1 component related cluster	-2.3
VVTU22814_at	GSVIVP00005721001	Q9LQ30 F14M2.10 protein related cluster	-2.3
VVTU8769_at	GSVIVP00001076001	O64647 Putative PCF2-like DNA bindinG protein related cluster	-2.3
VVTU16420_at	GSVIVP00034304001	Q9LU15 Histidine-containing phosphotransfer protein 4 related cluster	-2.3
VVTU8242_s_at	GSVIVP00016474001	Q2R122 Early Flowering 4, putative, expressed related cluster	-2.3
VVTU38338_x_at	GSVIVP00035995001	Q9SW54 Hypothetical protein T11I11.40 related cluster	-2.3
VVTU11008_at	VVTU11008_at	Q2V301 Protein At5g50175 related cluster	-2.3
VVTU17575_at	GSVIVP00023881001	AF271074 Vitis vinifera alcohol dehydrogenase 2 (Adh2) gene, complete cds.	-2.4
VVTU15479_at	GSVIVP00034532001	Q9C941 Putative non-phototropic hypocotyl; 25081-26618 related cluster	-2.4
VVTU16881_at	GSVIVP00028656001	Q1T152 IMP dehydrogenase GMP reductase related cluster	-2.4
VVTU23302_at	GSVIVP00027255001	Q5Z4M1 Putative microtubule-associated protein related cluster	-2.4
VVTU5622_s_at	GSVIVP00015032001	UPI000021A6DA Cluster related to UPI000021A6DA; Hypothetical protein MG06650.4	-2.4
VVTU16206_at	GSVIVP00036015001	Q9SW57 Hypothetical protein T11I11.10 related cluster	-2.4
VVTU40277_at	GSVIVP00000459001	Q9FGG3 nodulin-like protein related cluster	-2.4
VVTU30405_at	GSVIVP00032045001	Q9FT02 (1-4)-beta-mannan endohydrolase related cluster	-2.4
VVTU1835_s_at	GSVIVP00021523001	Q93ZE7 AT3g52500 F22O6_120 related cluster	-2.4
VVTU9979_at	GSVIVP00004456001	UPI000004822F Cluster related to UPI000004822F; ATP binding carbohydrate binding kinase protein kinase protein serine threonine kinase protein-tyrosine kinase sugar binding	-2.4
VVTU11184_s_at	GSVIVP00014554001	Q6IDJ6 At1g35180 related cluster	-2.4
VVTU1248_s_at	TC70058	Q9C867 Protein kinase, putative related cluster	-2.4
VVTU21495_s_at	GSVIVP00000793001	Q10E61 Expressed protein related cluster	-2.4
VVTU17922_s_at	GSVIVP00006907001	Q9SWA8 Glycine-rich RNA-bindinG protein related cluster	-2.4
VVTU20326_at	GSVIVP00019764001	Q1SCC2 cullin related cluster	-2.4
VVTU20577_at	GSVIVP00016840001	Q1RVL3 E-class P450, group I related cluster	-2.4
VVTU38461_at	TC61677	Q1T553 Reverse transcriptase (RNA-dependent DNA polymerase), putative related cluster	-2.4
VVTU10859_at	GSVIVP00002582001	Q9ZPR1 Cell division control protein 48 homolog B related cluster	-2.4
VVTU35706_at	GSVIVP00014574001	Q9AR07 Jasmonate O-methyltransferase related cluster	-2.4
VVTU13996_at	GSVIVP00019181001	Q1RY91 CCR4-Not complex component, Not1 related cluster	-2.4
VVTU17600_at	GSVIVP00032174001	Q0PJI8 Myb transcription factor Myb127 related cluster	-2.4
VVTU39954_at	GSVIVP00028590001	Q8GZP6 Allergen Ana o 2 related cluster	-2.4
VVTU6921_at	GSVIVP00018457001	Q1SYV3 Hypothetical protein related cluster	-2.4
VVTU11499_at	GSVIVP00019642001	Q1SRF6 GRAS transcription factor related cluster	-2.4
VVTU1314_at	GSVIVP00018907001	Q9SJG7 Hypothetical protein At2g42900 related cluster	-2.4
VVTU12436_at	GSVIVP00036380001	Q58FS3 RADIALIS related cluster	-2.4
VVTU6518_at	GSVIVP00024719001	Q1SRM5 PAP fibrillin related cluster	-2.4
VVTU6536_at	GSVIVP00030518001	Q9M4G9 Putative Ripening-related protein related cluster	-2.4
VVTU37958_at	TC53353	Q1RUR7 Hypothetical protein related cluster	-2.4
VVTU34485_at	GSVIVP00007662001	Q0H3C6 Violaxanthin de-epoxidase related cluster	-2.4
VVTU16843_at	GSVIVP00011948001	Q2V469 Protein At2g22660 related cluster	-2.4
VVTU28557_at	GSVIVP00022690001	Q6NKW1 At1g56230 related cluster	-2.4
VVTU21905_at	GSVIVP00020305001	Q9LW56 Similarity to long chain fatty alcohol oxidase related cluster	-2.5
VVTU8517_s_at	GSVIVP00032792001	Q1RSU5 Biopterin transport-related protein BT1 related cluster	-2.5
VVTU13247_at	GSVIVP00029887001	Q2VT54 DC1.2-like related cluster	-2.5
VVTU20617_at	VVTU20617_at	Q0KII0 IS10 transposase, putative related cluster	-2.5
VVTU2764_s_at	GSVIVP00034436001	Q9C6D2 Hypothetical protein F10F5.1 related cluster	-2.5
VVTU38453_s_at	GSVIVP00006090001	Q9LEG2 Putative sugar transporter related cluster	-2.5
VVTU12755_at	GSVIVP00027325001	Q9AV43 Hypothetical protein OSJNBa0001O14.25 related cluster	-2.5
VVTU15007_at	GSVIVP00028109001	Q6UEJ2 Mini-chromosome maintenance 7 related cluster	-2.5
VVTU13121_s_at	CF200899	Q84LH8 PIF3 like basic Helix Loop Helix protein related cluster	-2.5
VVTU2258_at	GSVIVP00021072001	Q1SSK0 Methyladenine glycosylase related cluster	-2.5
VVTU39804_s_at	GSVIVP00020458001	AY043234 Vitis vinifera putative Pectate lyase mRNA, partial cds.	-2.5
VVTU22963_at	GSVIVP00029973001	Q1T435 Leucine-rich repeat; Leucine-rich repeat, cysteine-containing type related cluster	-2.5
VVTU1673_at	GSVIVP00015908001	Q10I42 HAD-superfamily hydrolase, subfamily IA, variant 3 containing protein, expressed related cluster	-2.5
VVTU40198_at	TC52735	Q75L11 Probable Histone H2A.6 related cluster	-2.5
VVTU6932_at	GSVIVP00016437001	Q0PNH1 Cytochrome P450 related cluster	-2.5

Probe set	Unique Gene ID	Annotation	Fold-change
VVTU1633_at	GSVIVP00008438001	Q93VM6 AT4g39900 T5J17_70 related cluster	-2.5
VVTU2766_at	GSVIVP00013365001	Q5I6D6 Sinapyl alcohol dehydrogenase-like protein related cluster	-2.5
VVTU20465_at	VVTU20465_at	Q40896 Zinc-finger DNA bindinG protein related cluster	-2.5
VVTU31473_at	GSVIVP00027051001	UPI000034F3F4 Cluster related to UPI000034F3F4; unknown protein	-2.5
VVTU34534_at	GSVIVP00029853001	Q0JKH2 Os01g0674700 protein related cluster	-2.5
VVTU2959_at	GSVIVP00024444001	Q1S0U0 Response regulator receiver; CCT related cluster	-2.5
VVTU34773_at	GSVIVP00021374001	Q2VWB7 Prf interactor 30137 related cluster	-2.5
VVTU24793_at	GSVIVP00028921001	Q3ECF1 Protein At1g70100 related cluster	-2.5
VVTU17824_x_at	GSVIVP00031447001	Q40374 Pathogenesis-related protein PR-1 precursor related cluster	-2.5
VVTU8940_at	TC64347	Q9MA43 Histone-lysine N-methyltransferase ATX2 related cluster	-2.5
VVTU116_at	GSVIVP00001978001	Q9FHI9 Arabidopsis thaliana genomic DNA, chromosome 5, P1 clone:MFC19 related cluster	-2.5
VVTU37430_at	GSVIVP00025817001	Q1S928 Hypothetical protein related cluster	-2.6
VVTU4625_at	TC52429	Q41255 arabinogalactan-protein related cluster	-2.6
VVTU19121_s_at	GSVIVP00011714001	Q5NE32 Type 1 non-specific lipid transfer protein precursor related cluster	-2.6
VVTU15820_at	GSVIVP00003649001	Q0JCZ5 Os04g0441900 protein related cluster	-2.6
VVTU1201_at	GSVIVP00020779001	Q9FL08 nodulin-like protein related cluster	-2.6
VVTU29946_x_at	GSVIVP00022096001	Q1SRU1 Glycosyl transferase, family 2 related cluster	-2.6
VVTU18660_s_at	TC67367	Q9M4H8 Putative Ripening-related protein related cluster	-2.6
VVTU35892_at	GSVIVP00006690001	Q6K4V2 Putative NEC1 related cluster	-2.6
VVTU36436_at	TC60505	Q9LQH2 F15O4.13 related cluster	-2.6
VVTU2767_at	GSVIVP00022166001	Q5BM98 Secondary cell wall-related glycosyltransferase family 47 related cluster	-2.6
VVTU21063_s_at	GSVIVP00035572001	Q0ZIW0 Hypothetical chloroplast RF1 related cluster	-2.6
VVTU13696_at	GSVIVP00015230001	Q4PSG2 Hydrolase related cluster	-2.6
VVTU27462_x_at	TC69007	P27518 chlorophyll a-b bindinG protein 151, chloroplast precursor related cluster	-2.6
VVTU32180_x_at	CB348560	Q7Y238 Hevein-like antimicrobial peptide related cluster	-2.7
VVTU351_at	VVTU351_at	Q25A64 H0306F03.11 protein related cluster	-2.7
VVTU4627_at	GSVIVP00014628001	Q9STG6 DUTP pyrophosphatase-like protein related cluster	-2.7
VVTU32695_s_at	GSVIVP00036771001	Q8LBL4 Putative Thaumatin-like protein related cluster	-2.7
VVTU2576_x_at	GSVIVP00032590001	Q1SFF5 2OG-Fe(II) oxygenase related cluster	-2.7
VVTU16116_s_at	GSVIVP00020459001	Q59296 catalase related cluster	-2.7
VVTU24287_at	GSVIVP00032666001	Q1S2K0 Aux IAA protein related cluster	-2.7
VVTU12616_s_at	GSVIVP00008840001	Q708X5 Leucine rich repeat protein precursor related cluster	-2.7
VVTU14513_s_at	TC64456	Q0PJH6 Myb transcription factor Myb142 related cluster	-2.7
VVTU16889_at	TC57062	P92557 Mitochondrial ribosomal protein S7 related cluster	-2.7
VVTU39764_s_at	GSVIVP00024306001	Q7X9Q3 expansin related cluster	-2.7
VVTU12188_s_at	GSVIVP00018131001	Q27U75 Pectate lyase related cluster	-2.7
VVTU37434_x_at	VVTU37434_x_at	Q9M4H3 Putative Metallothionein-like protein related cluster	-2.7
VVTU22869_at	GSVIVP00033440001	Q9FKU3 receptor protein kinase-like protein related cluster	-2.7
VVTU16029_at	GSVIVP00011355001	Q2HST1 Ferric reductase-like transmembrane component related cluster	-2.7
VVTU23904_at	DT031319	UPI00000A11FF Cluster related to UPI00000A11FF; putative gag pol Polyprotein	-2.7
VVTU16566_at	TC56334	Q0ZCC8 Integrase related cluster	-2.7
VVTU11839_s_at	GSVIVP00000417001	P12459 tubulin beta-1 chain related cluster	-2.7
VVTU34750_s_at	GSVIVP00019328001	Q9FKY4 Gb|AAD32907.1 related cluster	-2.8
VVTU12913_at	GSVIVP00002098001	Q9SEK4 Succinic semialdehyde dehydrogenase related cluster	-2.8
VVTU12371_at	GSVIVP00023726001	Q40434 PSI-D1 precursor related cluster	-2.8
VVTU24186_at	GSVIVP00026093001	Q7XIJ3 Putative phototropic response protein family related cluster	-2.8
VVTU25232_at	GSVIVP00037941001	Q2HUL4 Short-chain dehydrogenase reductase SDR related cluster	-2.8
VVTU15324_at	GSVIVP00015527001	Q9FND6 Selenium-binding protein-like related cluster	-2.8
VVTU31651_at	CB345590	P10690 Photosystem II 10 kDa polypeptide, chloroplast precursor related cluster	-2.8
VVTU323_at	GSVIVP00016185001	Q6Z5N1 Putative potyviral helper component protease-interActinG protein 2 related cluster	-2.8
VVTU3194_s_at	TC63633	Q9FN38 Arabidopsis thaliana genomic DNA, chromosome 5, TAC clone:K19P17 related cluster	-2.8
VVTU34351_at	GSVIVP00012883001	Q9ZPL3 Pulvinus outward-rectifying channel for potassium SPOCK1 related cluster	-2.8
VVTU17509_at	GSVIVP00027905001	Q8RZF8 Lustrin A-like related cluster	-2.8
VVTU10261_at	GSVIVP00007506001	Q710F0 CDT1a protein related cluster	-2.8
VVTU13582_at	GSVIVP00024536001	DQ358107 Vitis vinifera aquaPorin PIP2 (pip2) mRNA, complete cds.	-2.8
VVTU39534_at	GSVIVP00029182001	Q9LN91 T12C24.2 related cluster	-2.8
VVTU8464_at	GSVIVP00017793001	Q5WM33 Alpha-dioxygenase 2 related cluster	-2.8
VVTU4478_at	GSVIVP00017631001	Q0PJJ2 Myb transcription factor Myb109 related cluster	-2.8
VVTU22181_at	GSVIVP00019332001	Q9SD02 Protein phosphatase 2C-like protein related cluster	-2.8
VVTU35521_at	GSVIVP00024993001	Q40287 Anthocyanidin 3-O-Glucosyltransferase related cluster	-2.8
VVTU12449_s_at	GSVIVP00000440001	Q2LAK8 Cytochrome P450 monooxygenase CYP86A24 related cluster	-2.8
VVTU19893_at	GSVIVP00023190001	Q9SP55 Vacuolar ATP synthase subunit G related cluster	-2.9
VVTU6393_at	GSVIVP00035984001	Q9LR01 F10A5.20 related cluster	-2.9
VVTU501_at	GSVIVP00026004001	Q1W1G0 CK25 related cluster	-2.9
VVTU25681_at	GSVIVP00016088001	Q1T4S0 PIK-related kinase, FATC related cluster	-2.9
VVTU13584_s_at	GSVIVP00015267001	Q9SGZ8 F28K19.24 related cluster	-2.9
VVTU4174_s_at	GSVIVP00021820001	Q1SYD5 Transport protein particle (TRAPP) component, Bet3 related cluster	-2.9
VVTU769_s_at	GSVIVP00031570001	Q8RVP5 Class III peroxidase related cluster	-2.9
VVTU12080_s_at	GSVIVP00030705001	Q93XJ1 Pectate lyase related cluster	-3.0
VVTU7322_at	GSVIVP00001737001	Q5Z916 Putative proline-rich protein APG related cluster	-3.0
VVTU1875_at	GSVIVP00006915001	Q0PJH5 Myb transcription factor Myb73 related cluster	-3.0
VVTU4071_at	GSVIVP00030552001	Q9LQU1 F10B6.30 related cluster	-3.0
VVTU390_at	GSVIVP00006699001	Q3Y6V1 cellulose synthase-like protein CslG related cluster	-3.0

Probe set	Unique Gene ID	Annotation	Fold-change
VVTU252_at	VVTU252_at	Q9FJZ5 Arabidopsis thaliana genomic DNA, chromosome 5, TAC clone:K1F13 related cluster	-3.0
VVTU7662_at	GSVIVP00028445001	AY194361 Vitis vinifera putative GDSL-motif lipase(Lip) mRNA, partial cds.	-3.0
VVTU30338_at	TC70360	Q6ZSA2 CDNA FLJ45698 fis, clone FEBRA2017811 related cluster	-3.0
VVTU6810_at	GSVIVP00029523001	O80629 Putative phospholipaserelated cluster	-3.0
VVTU8696_at	GSVIVP00006913001	Q0WR89 Probable Xyloglucan endotransglucosylase hydrolase protein 8 precursor related cluster	-3.0
VVTU499_at	GSVIVP00030544001	Q9ZVE7 Expressed protein related cluster	-3.1
VVTU37534_at	GSVIVP00038103001	Q1KUU3 Hypothetical protein related cluster	-3.1
VVTU5405_s_at	GSVIVP00031037001	Q2HW86 Hypothetical protein related cluster	-3.1
VVTU16209_at	GSVIVP00024410001	Q1S1C2 Probable fatty acid elongase [imported]-Arabidopsis thaliana related cluster	-3.1
VVTU1134_at	GSVIVP00038524001	P93205 SBT2 protein related cluster	-3.1
VVTU13314_at	GSVIVP00009171001	Q1SHD8 F20D23.20 protein-Arabidopsis thaliana-related related cluster	-3.1
VVTU22615_at	GSVIVP00036060001	Q09JM0 10 kDa putative secreted protein related cluster	-3.1
VVTU14761_s_at	GSVIVP00022645001	Q8H2B1 DnaJ-like protein related cluster	-3.1
VVTU24974_at	GSVIVP00015158001	P22195 Cationic peroxidase 1 precursor related cluster	-3.2
VVTU16219_at	GSVIVP00028320001	Q8LBM2 Hypothetical protein related cluster	-3.2
VVTU10120_at	GSVIVP00016022001	Q5VQM7 Putative receptor-like protein kinase INRPK1 related cluster	-3.2
VVTU19090_s_at	GSVIVP00035985001	Q94B76 Putative auxin-regulated protein related cluster	-3.2
VVTU21101_s_at	GSVIVP00000516001	Q9FYG4 F1N21.11 related cluster	-3.2
VVTU26320_at	CF605686	Q5VJE1 Putative caprolactone hydrolase related cluster	-3.2
VVTU37263_at	GSVIVP00014821001	Q9FMM9 Arabidopsis thaliana genomic DNA, chromosome 5, P1 clone:MBD2 related cluster	-3.2
VVTU23899_at	DT031395	O23757 Reverse transcriptase related cluster	-3.2
VVTU10440_at	GSVIVP00002151001	Q53LP9 Hypothetical protein related cluster	-3.2
VVTU9955_at	GSVIVP00006889001	Q94AU9 Hypothetical protein At4g04330 related cluster	-3.3
VVTU13736_at	GSVIVP00021604001	O04846 Carbonic anhydrase related cluster	-3.3
VVTU18285_at	GSVIVP00037388001	Q8LEH2 Putative phospholipaserelatedprotein related cluster	-3.3
VVTU10495_at	VVTU10495_at	Q1S579 Hypothetical protein related cluster	-3.3
VVTU2854_at	TC66878	Q93WI1 At1g32920 F9L11_25 related cluster	-3.3
VVTU5431_at	GSVIVP00025322001	Q2HRH3 Gibberellin regulated protein related cluster	-3.3
VVTU12273_at	GSVIVP00029445001	Q84US9 expansin related cluster	-3.4
VVTU5192_at	GSVIVP00016401001	Q1SGW0 Hypothetical protein related cluster	-3.4
VVTU14641_at	GSVIVP00035305001	Q0JH44 Os01g0884900 protein related cluster	-3.5
VVTU3092_s_at	GSVIVP00029443001	Q6TKQ7 lipid transfer protein related cluster	-3.5
VVTU1895_at	GSVIVP00021481001	O80437 Glycerol-3-phosphate acyltransferase 6 related cluster	-3.5
VVTU13112_at	GSVIVP00018553001	Q9LFQ0 Hypothetical protein F2G14_170 related cluster	-3.5
VVTU12999_at	GSVIVP00000513001	O22977 T19F6.6 protein related cluster	-3.6
VVTU20271_at	GSVIVP00016362001	Q3E989 Protein At5g20860 related cluster	-3.7
VVTU19011_at	GSVIVP00023848001	O22511 glutathione reductase related cluster	-3.7
VVTU14552_s_at	GSVIVP00036369001	Q1S089 Helix-loop-helix DNA-binding related cluster	-3.7
VVTU22566_at	GSVIVP00006705001	Q3Y6V1 cellulose synthase-like protein CslG related cluster	-3.7
VVTU4179_at	GSVIVP00024021001	Q1SIP8 Probable indole-3-acetate beta-Glucosyltransferase T27E13.11- Arabidopsis thaliana related cluster	-3.8
VVTU21834_at	GSVIVP00025669001	Q4PSG2 Hydrolase related cluster	-3.8
VVTU39313_at	TC57796	Q9LFL5 Hypothetical protein F2K13_10 related cluster	-3.8
VVTU36246_at	TC64604	O81060 Similar to lateembryogenesis abundant proteins related cluster	-4.0
VVTU6502_at	GSVIVP00001163001	O49895 Hypothetical protein related cluster	-4.0
VVTU40732_at	TC63881	Q1RYW8 Hypothetical protein related cluster	-4.2
VVTU11244_at	VVTU11244_at	P07919 Ubiquinol-cytochrome c reductase complex 11 kDa protein, mitochondrial precursor related cluster	-4.2
VVTU8304_at	GSVIVP00026695001	Q6F5D8 Putative ammonium transporter related cluster	-4.3
VVTU25603_s_at	GSVIVP00031336001	Q9SUM5 Hypothetical protein F9N11.30 related cluster	-4.3
VVTU13122_s_at	TC64246	Q0PJH6 Myb transcription factor Myb142 related cluster	-4.3
VVTU10506_at	GSVIVP00022862001	Q05929 EDGP precursor related cluster	-4.4
VVTU8717_at	GSVIVP00037323001	Q6NLE8 At2g27385 related cluster	-4.4
VVTU31215_s_at	CB343496	Q9FMX6 Gb|AAD21732.1 related cluster	-4.4
VVTU29372_x_at	GSVIVP00014843001	Q38708 Subtilisin-like protease related cluster	-4.6
VVTU22820_at	GSVIVP00002663001	Q9SU24 Origin recognition complex subunit 1-like protein related cluster	-4.7
VVTU4276_at	GSVIVP00029891001	Q2VT54 DC1.2-like related cluster	-4.9
VVTU14923_at	GSVIVP00008972001	Q9FE98 Alliinase, putative; 28821-30567 related cluster	-5.0
VVTU38329_at	GSVIVP00035962001	Q1RYD1 Hypothetical protein related cluster	-5.0
VVTU34610_s_at	GSVIVP00033035001	Q8LFJ2 Hypothetical protein related cluster	-5.0
VVTU16070_at	GSVIVP00003139001	Q4V3D9 At3g57030 related cluster	-5.0
VVTU1706_at	GSVIVP00004380001	Q41495 STS14 protein precursor related cluster	-5.1
VVTU19563_s_at	GSVIVP00023937001	Q9FLC6 Putative RING-H2 finger protein ATL5B precursor related cluster	-5.2
VVTU40336_s_at	GSVIVP00031892001	Q9FMX6 Gb|AAD21732.1 related cluster	-5.2
VVTU2887_s_at	GSVIVP00018814001	Q8LD91 Heat shock protein, putative related cluster	-5.2
VVTU14966_at	GSVIVP00014845001	Q9ZS42 SBT4E protein related cluster	-5.3
VVTU28503_s_at	GSVIVP00036374001	Q0PJH6 Myb transcription factor Myb142 related cluster	-5.6
VVTU13627_at	GSVIVP00020991001	Q8L5J6 expansin 3 related cluster	-5.7
VVTU5847_at	GSVIVP00024028001	Q8LDM8 Hypothetical protein related cluster	-5.7
VVTU1459_at	GSVIVP00025974001	Q67ZI9 Putative GDSL-motif lipasehydrolase related cluster	-5.7
VVTU39696_at	TC63554	Q1SUI9 Reverse transcriptase-beet retrotransposon related cluster	-5.8
VVTU17653_at	GSVIVP00033575001	Q6JX04 Chitinase-like protein related cluster	-5.9
VVTU22691_s_at	GSVIVP00016335001	Q49RB3 Gip1-like protein related cluster	-6.1

Probe set	Unique Gene ID	Annotation	Fold-change
VVTU19248_s_at	GSVIVP00015118001	Q2PEZ2 Hypothetical protein related cluster	-6.4
VVTU12410_at	GSVIVP00006912001	O23787 Thiazole biosynthetic enzyme, chloroplast precursor related cluster	-6.8
VVTU8383_at	GSVIVP00000349001	Q9XI33 F9L1.31 protein related cluster	-7.2
VVTU30935_at	GSVIVP00028217001	Q75LI3 Expressed protein related cluster	-7.8
VVTU15550_s_at	GSVIVP00015920001	Q4JLV6 Pectate lyase related cluster	-8.9
VVTU35251_at	GSVIVP00015210001	Q8L5C7 UDP-glucuronosyltransferase related cluster	-19.1
