# Supplementary material for: Transcriptome changes in grapevine (Vitis vinifera L.) cv. Malbec leaves induced by ultraviolet-B radiation
Source: BMC Plant Biol. 2010 Oct 20;10:224. doi: 10.1186/1471-2229-10-224 (PMC3017828; doi:10.1186/1471-2229-10-224)
Supplement: Additional file 3 — qRT-PCR primers. PDF file describing the DNA primers used for quantitative real time reverse transcription polymerase chain reaction (qRT-PCR). [file 1471-2229-10-224-S3.PDF]

The list of PCR primers used in quantitative real-time RT-PCR

Gene	GeneChip probeset	5'-Forward primer-3'	5'-Reverse primer-3'
4CL1	VVTU2645_at	GCAGGATTTTACCCGATGGA	CTGATGCCGCTGTTGTTTCG
CHS1	VVTU39820_s_at	TCTCTTCCTTCAGACCCAGTT	GTCCCAGGGTTGATTTCCAA
CHS3	VVTU21956_s_at	CTCGGGCTTTAGGGCTAAT	TTTGGGCATCAAGGACTGGA
DIR3	VVTU39372_at	CATTGTCGCTGAATGAGTAAGTCG	CACCGATGGCAAGTATAACGG
DIR4	VVTU35718_at	GCTACCATTGTACTTGCCATCAG	GAAGCACTGAATAACTCGTCACC
MybB5	VVTU5899_at	GCAGGGTGTTGAAGCCAAAT	AGTCCAGTCGTTCGGGTTC
PAL1	VVTU1670_at	GTTCCAGCCACTGAGACAAT	CCGAACCGAATCAAGGACTG
PHB	VVTU14882_at	AGATACGCAAAGCCCTGTTG	GTGCTGCTCCCTGAAAACAA
TCP5	VVTU37508_at	TATCTGAGACCACGCTATGC	GTTTTGCTCCTGCTGTTCGT
RSGTc	VVTU1801_x_at	CTGACCTCGTCCACAAACTC	GCGGAGCTGAAGGAAAACAC
RSGTa	VVTU36151_s_at	CGAACCTCGTCGACAAAACC	GCGGAGTTGAAGCAAAACGC
