# Supplementary figures and images for: Transcriptome changes in grapevine (Vitis vinifera L.) cv. Malbec leaves induced by ultraviolet-B radiation
Source: BMC Plant Biol. 2010 Oct 20;10:224. doi: 10.1186/1471-2229-10-224 (PMC3017828; doi:10.1186/1471-2229-10-224)

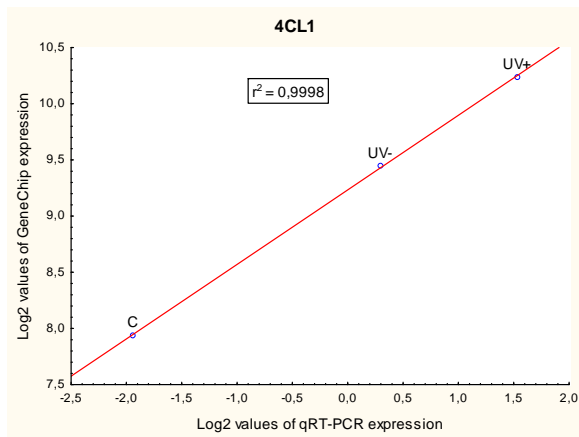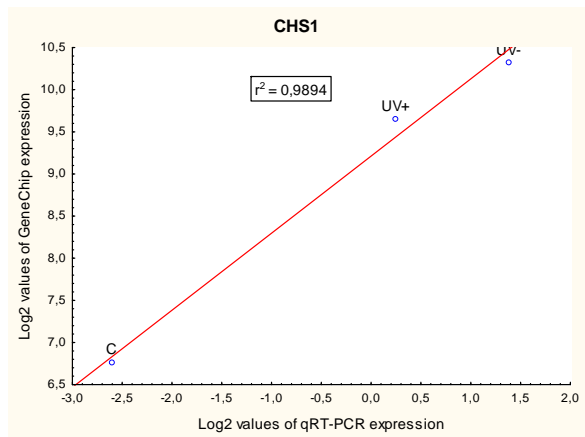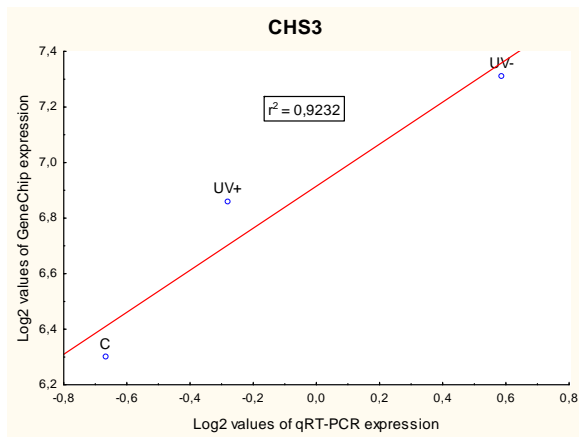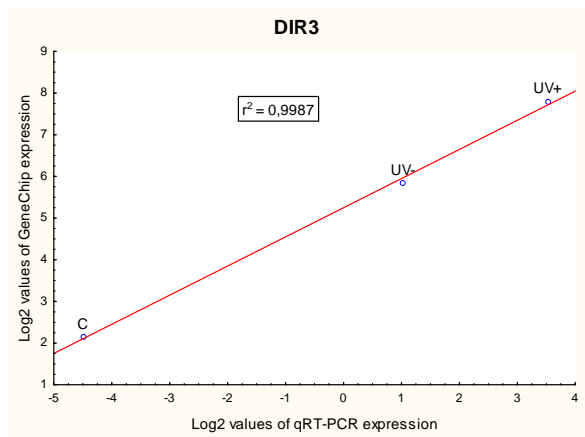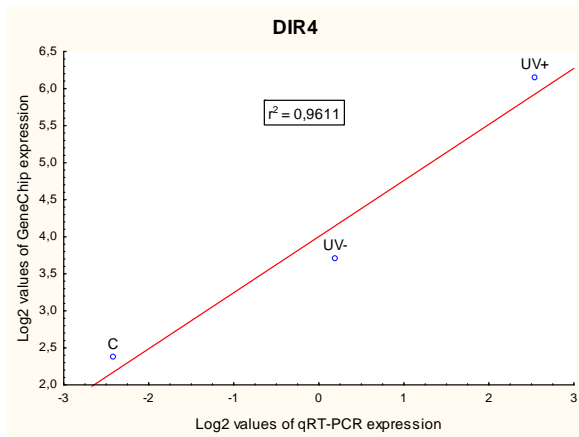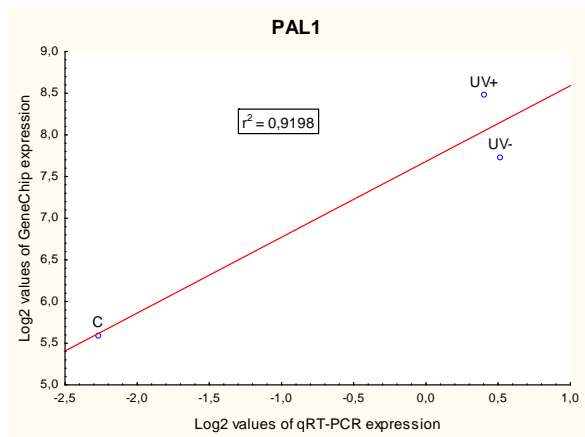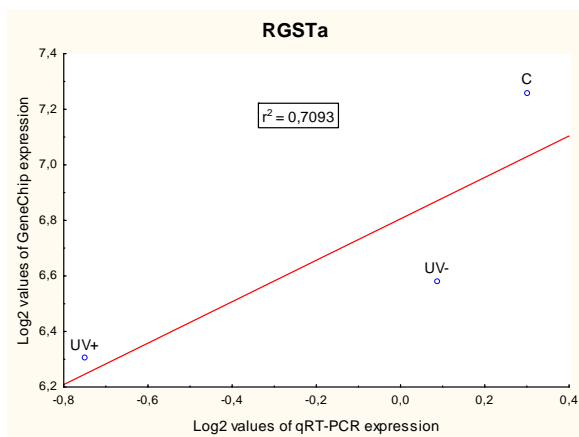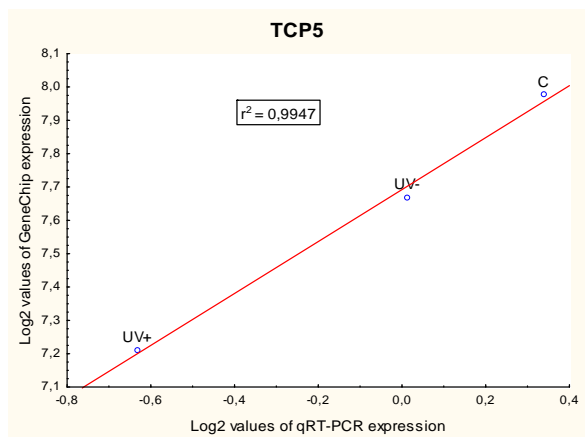

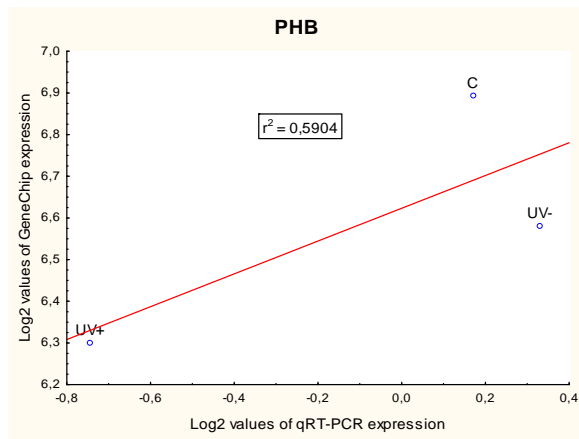

Supplement: Additional file 4 — qRT-PCR expression validation. PDF file illustrating the comparison of gene expression values reported by the GrapeGen Vitis vinifera Affymetrix GeneChip® and by quantitative real-time RT-PCR (qRT-PCR). The microarray log2 (expression ratio) values (y-axis) are plotted against the log2 (expression ratio) obtained by qRT-PCR (x-axis). Linear regression analyses (r2 values) are shown as insets. [file 1471-2229-10-224-S4.PDF]

a

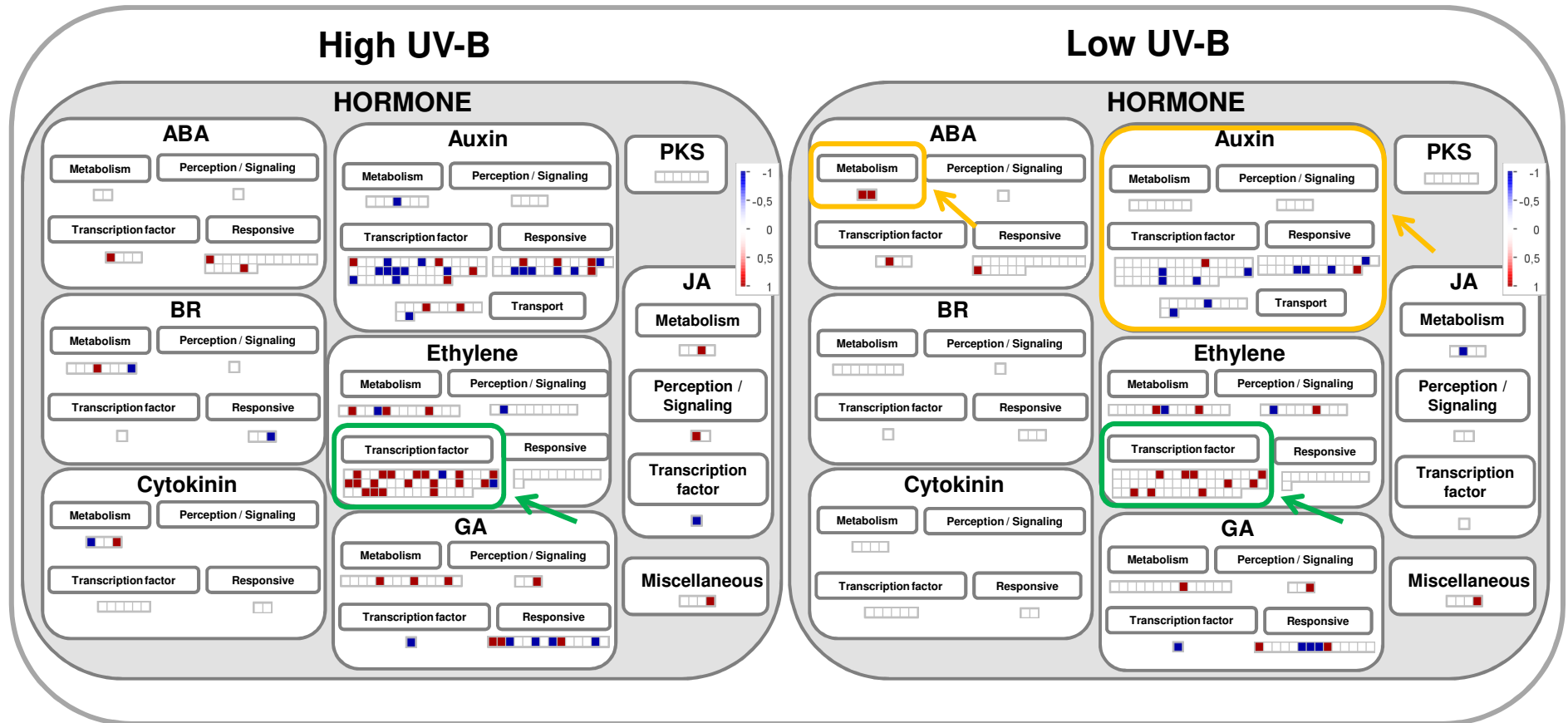

b

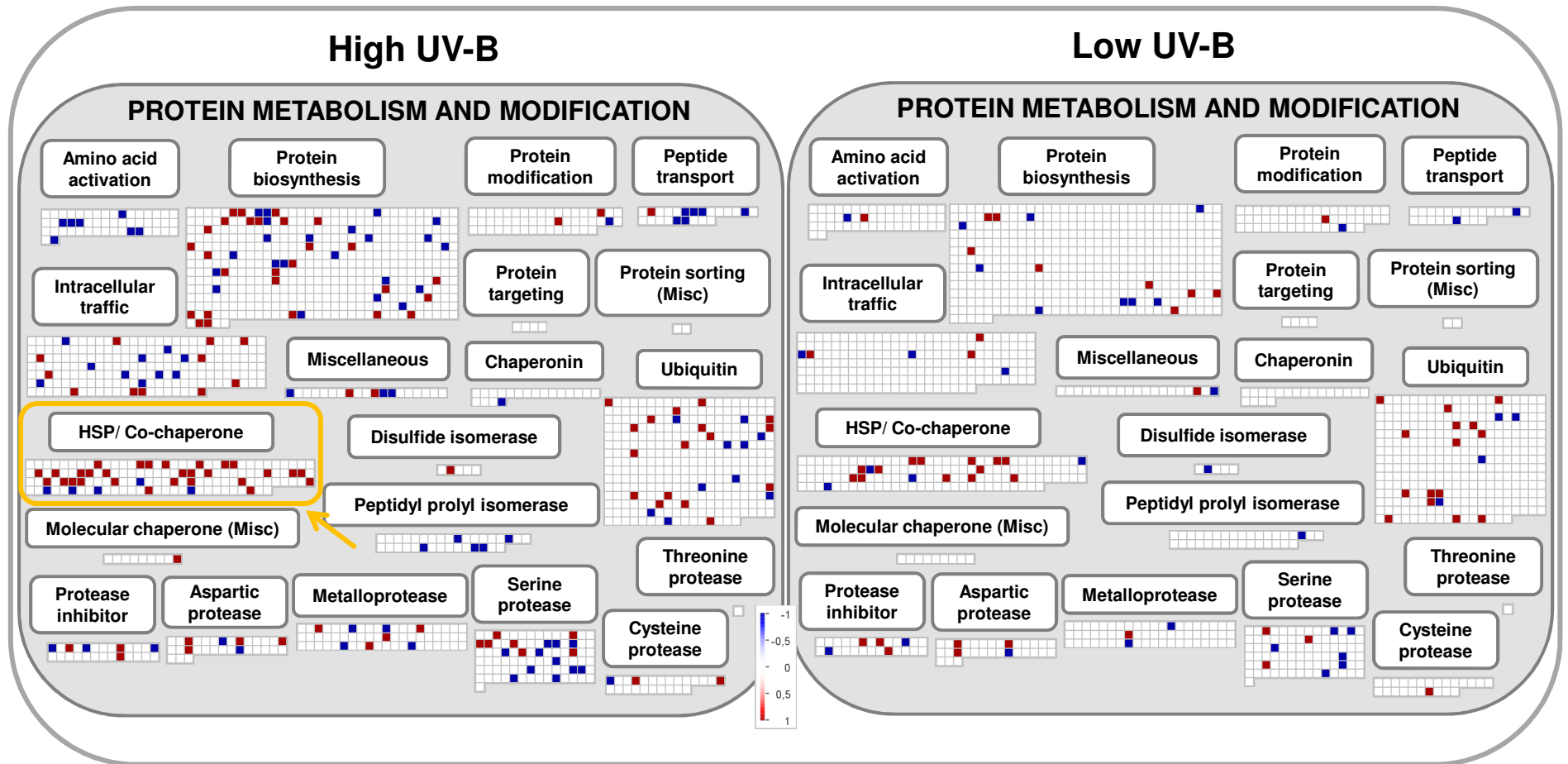

C

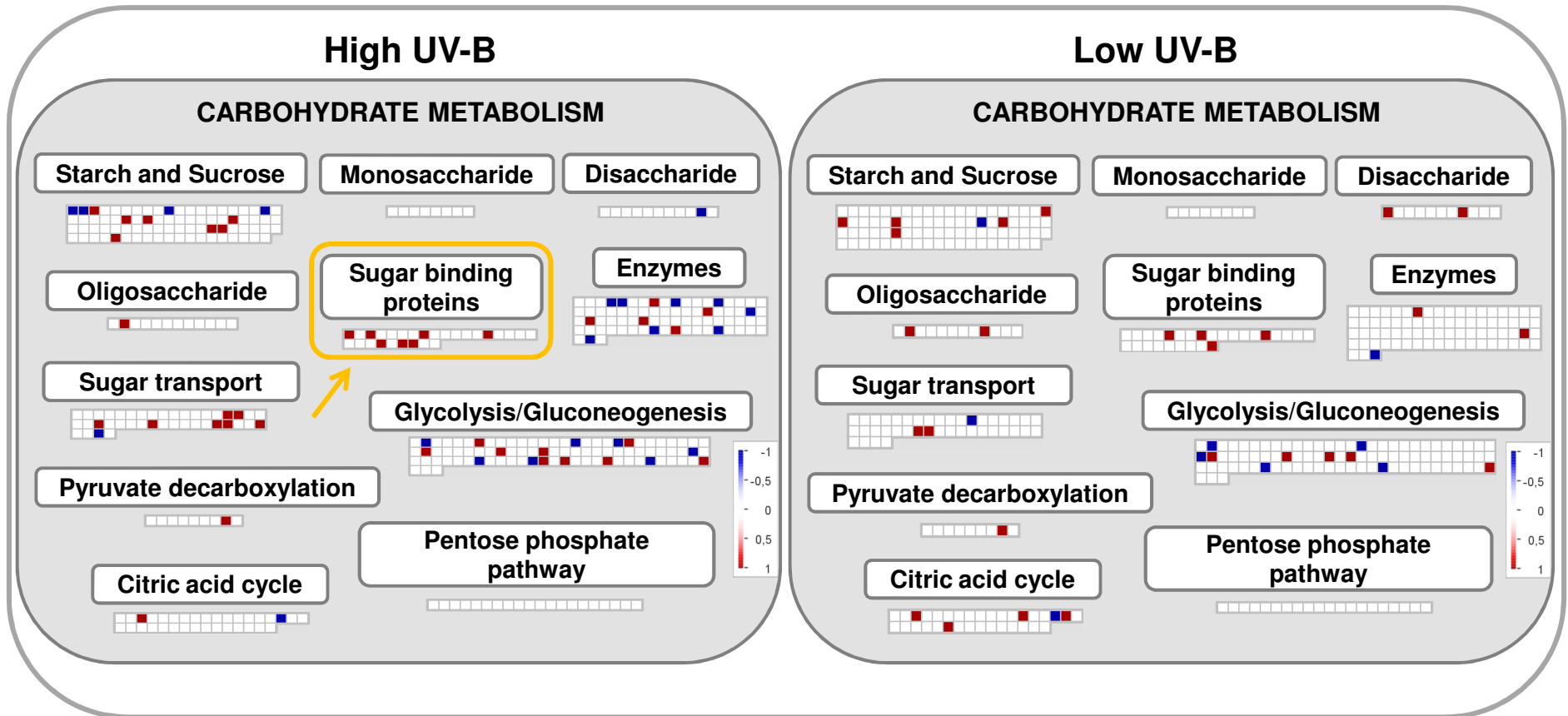

d

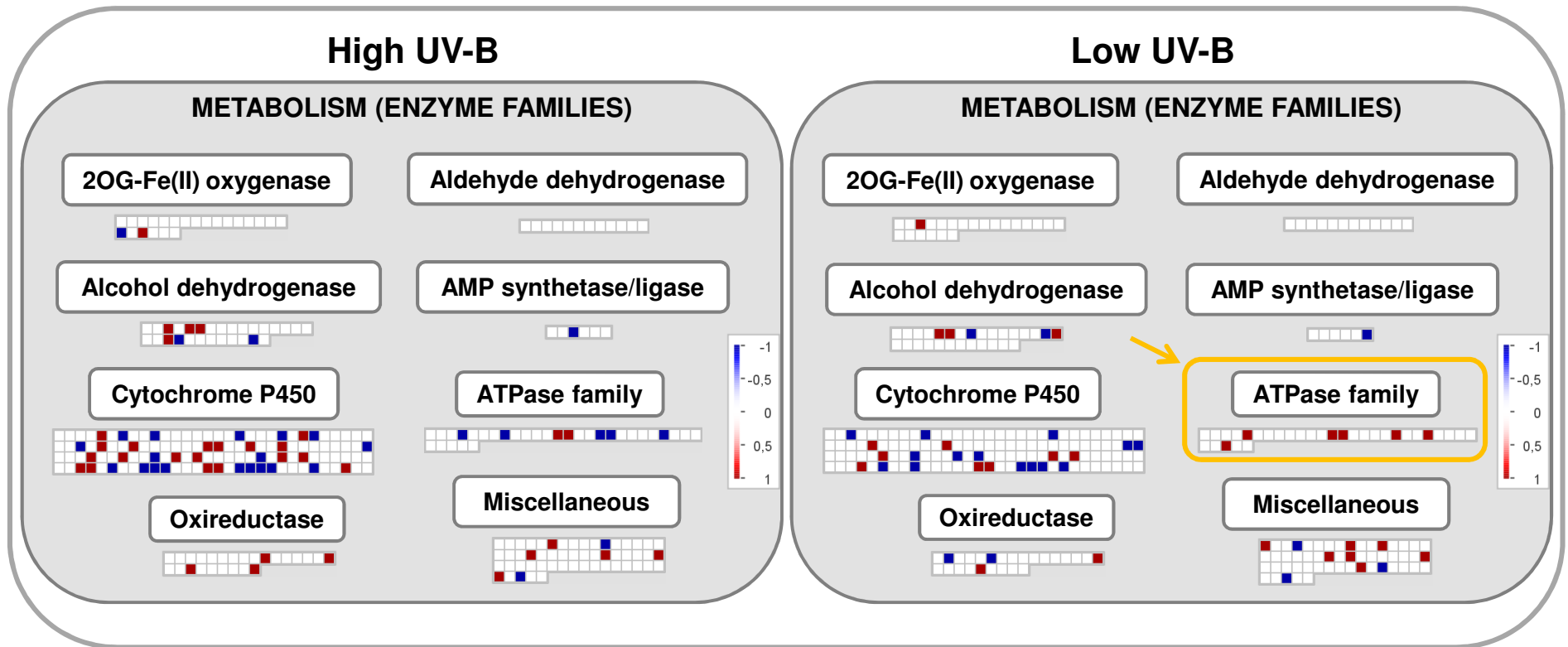

e

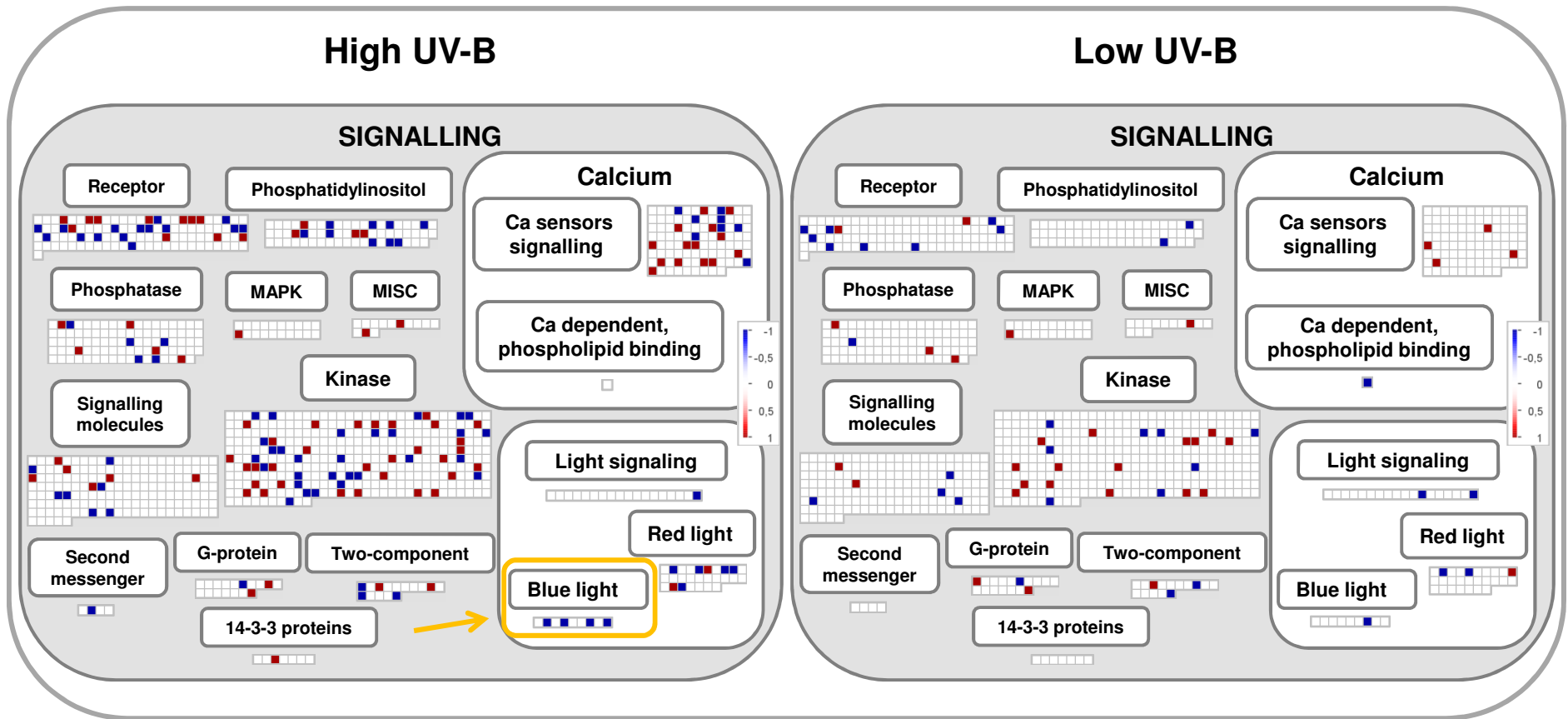

Supplement: Additional file 8 — MapMan diagrams of all significantly modulated functional categories not included as main Figures in the text. PDF file displaying the pictorial representation of the differentially expressed genes included in the following classes: (a) "Hormone", (b) "Protein metabolism and modification", (c) "Carbohydrate Metabolism", (d) "Metabolism-enzyme families", (e) "Signalling". Functional categories commonly regulated by both UV-B treatments are enclosed within green boxes, while those specifically regulated are enclosed within yellow boxes. [file 1471-2229-10-224-S8.PDF]
