# Supplementary material for: Transcriptome changes in grapevine (Vitis vinifera L.) cv. Malbec leaves induced by ultraviolet-B radiation
Source: BMC Plant Biol. 2010 Oct 20;10:224. doi: 10.1186/1471-2229-10-224 (PMC3017828; doi:10.1186/1471-2229-10-224)
Supplement: Additional file 5 — MapMan annotation. PDF file describing the MapMan BIN structure and the number of genes included in each BIN and subBIN. [file 1471-2229-10-224-S5.PDF]

BIN	BIN Name	Genes
1	Photosynthesis	181
1.1	Photosynthesis.Calvin cycle	35
1.2	Photosynthesis.Carbon assimilation	7
1.3	Photosynthesis. Chlorophyll biosynthesis	29
1.4	Photosynthesis. Photosystem I	41
1.5	Photosynthesis. Photosystem II	61
1.6	Photosynthesis. Chloroplast percursor	8
**2**	**Shikimate metabolism**	**39**
**3**	**Cell wall metabolism**	**426**
3.1	Cell wall metabolism.Cell wall biosynthesis	139
3.2	Cell wall metabolism.Cell wall modification	198
3.3	Cell wall metabolism.Structural protein	56
3.4	Cell wall metabolism.Related protein	33
**4**	**Cellular response overview**	**973**
4.1	Cellular response overview.Abiotic stress.Anoxia	9
4.2	Cellular response overview.Biotic stress	240
4.3	Cellular response overview.Abiotic stress.Cold	7
4.4	Cellular response overview.Abiotic stress.Light	11
4.5	Cellular response overview.Abiotic stress.Mineral	13
4.6	Cellular response overview.Abiotic stress.Osmotic	4
4.7	Cellular response overview.Abiotic stress.Oxidative	280
4.8	Cellular response overview.Abiotic stress.Ozone	3
4.9	Cellular response overview.Abiotic stress.Salinity	16
4.10	Cellular response overview.Stress miscellaneous	161
4.11	Cellular response overview.Abiotic stress.Drought	53
4.12	Cellular response overview.Abiotic stress.Wounding	17
4.13	Cellular response overview.Cell growth and death.Cell cycle	142
4.14	Cellular response overview.Cell growth and death.ProgrammedCell death	17
**5**	**Carbohydrate metabolism**	**401**
5.1	Carbohydrate metabolism.CHO binding proteins	27
5.2	Carbohydrate metabolism.CHO enzymes	75
5.3	Carbohydrate metabolism.Citric acid cycle	33
5.4	Carbohydrate metabolism.Disaccharides	11
5.5	Carbohydrate metabolism.Glycolysis_Gluconeogenesis	87
5.6	Carbohydrate metabolism.Monosaccharides	8
5.7	Carbohydrate metabolism.Oligosaccharides	12
5.8	Carbohydrate metabolism.Pentose phosphate pathway	20
5.9	Carbohydrate metabolism.Pyruvate decarboxylation	9
5.10	Carbohydrate metabolism.Starch and sucrose metabolism	79
5.11	Carbohydrate metabolism.Sugar transport	40
**6**	**Amino acid metabolism**	**200**
6.1	Amino acid metabolism.General metabolism	10
6.2	Amino acid metabolism.Alanine metabolism	4
6.3	Amino acid metabolism.Arginine metabolism	8
6.4	Amino acid metabolism.Asparagine, aspartate metabolism	11
6.5	Amino acid metabolism.Cysteine, homocysteine, methionine metabolism	24
6.6	Amino acid metabolism.Glutamine, glutamate metabolism	20
6.7	Amino acid metabolism.Glycine, serine metabolism	12
6.8	Amino acid metabolism.Histidine metabolism	8
6.9	Amino acid metabolism.Leucine, isoleucine, valine metabolism	24
6.10	Amino acid metabolism.Lysine metabolism	7
6.11	Amino acid metabolism.Phenylalanine, tyrosine, tryptophan metabolism	13
6.12	Amino acid metabolism.Proline metabolism	6

BIN	BIN Name	Genes
6.13	Amino acid metabolism.Threonine metabolism	4
6.14	Amino acid metabolism.GABA biosynthesis	12
6.15	Amino acid metabolism.Amino acid transport	37
**7**	**Energy**	**262**
7.1	Energy.Electron transport	234
7.2	Energy.ATP synthesis coupled proton transport	18
7.3	Energy.Photorespiration	10
**8**	**Signalling**	**948**
8.1	Signalling.14-3-3 family proteins	7
8.2	Signalling.Light signalling	54
8.2.1	Signalling.Light signalling.Blue light signalling	9
8.2.2	Signalling.Light signalling.Red light signalling	27
8.2.3	Signalling.Light signalling.General light signalling	18
8.3	Signalling.MAPK signalling	20
8.4	Signalling.Phosphatidylinositol signalling	59
8.5	Signalling.Protein kinase	320
8.6	Signalling.Protein phosphatase	89
8.7	Signalling.Receptor	101
8.8	Signalling.Signalling molecules	159
8.8.1	Signalling.Signalling molecules.Second messenger	4
8.8.2	Signalling.Signalling molecules.Signalling moleculesRegulation	15
8.9	Signalling.Two-component signalling	15
8.10	Signalling.G-protein signalling pathway	17
8.11	Signalling.Calcium	94
8.11.1	Signalling.Calcium.Calcium sensors and calcium signalling	93
8.11.2	Signalling.Calcium.Calcium dependent, phospholipid binding proteins	1
8.12	Signalling.Miscellaneous	13
**9**	**Myo-inositol**	**6**
**10**	**Lipid, fatty acid, steroid metabolism**	**392**
10.1	Lipid, fatty acid, steroid metabolism.Lipid metabolism	120
10.2	Lipid, fatty acid, steroid metabolism.Fatty acid metabolism	122
10.3	Lipid, fatty acid, steroid metabolism.Lipid and fatty acid binding	9
10.4	Lipid, fatty acid, steroid metabolism.Lipid transport	79
10.5	Lipid, fatty acid, steroid metabolism.Phospholipid metabolism	22
10.6	Lipid, fatty acid, steroid metabolism.Sphingolipid metabolism	11
10.7	Lipid, fatty acid, steroid metabolism.Steroid metabolism	29
**11**	**Protein metabolism and modification**	**1479**
11.1	Protein metabolism and modification.Amino acid activation	50
11.2	Protein metabolism and modification.Protein biosynthesis	421
11.3	Protein metabolism and modification.Protein modification	51
11.4	Protein metabolism and modification.Molecular chaperone	197
11.4.1	Protein metabolism and modification.Molecular chaperone.HSP	131
11.4.1.1	Protein metabolism and modification.Molecular chaperone.HSP.Co-chaperone HSP	46
11.4.2	Protein metabolism and modification.Molecular chaperone.Chaperonin	19
11.4.3	Protein metabolism and modification.Molecular chaperone.Disulfide isomerase	5
11.4.4	Protein metabolism and modification.Molecular chaperone.Peptidyl prolyl isomerase	33
11.4.5	Protein metabolism and modification.Molecular chaperone.Miscellaneous	9
11.5	Protein metabolism and modification.Protein sorting	218
11.5.1	Protein metabolism and modification.Protein sorting.Intracellular protein traffic	189
11.5.2	Protein metabolism and modification.Protein sorting.Peptide transport	23
11.5.3	Protein metabolism and modification.Protein sorting.Protein targeting	4
11.5.4	Protein metabolism and modification.Protein sorting.Miscellaneous	2
11.6	Protein metabolism and modification.Proteolysis	523

BIN	BIN Name	Genes
11.6.1	Protein metabolism and modification.Proteolysis.Aspartic protease	31
11.6.2	Protein metabolism and modification.Proteolysis.Cysteine protease	24
11.6.3	Protein metabolism and modification.Proteolysis.Metalloprotease	60
11.6.5	Protein metabolism and modification.Proteolysis.Serine protease	85
11.6.6	Protein metabolism and modification.Proteolysis.Threonine protease	1
11.6.7	Protein metabolism and modification.Proteolysis.Protease inhibitor	26
11.6.8	Protein metabolism and modification.Proteolysis.Ubiquitin mediated	296
11.7	Protein metabolism and modification.Miscellaneous	19
**12**	**Ankyrin domain**	**33**
**13**	**Armadillo repeat protein**	**13**
**14**	**Transport Overview**	**448**
14.1	Transport Overview.Ion transport	200
14.1.1	Transport Overview.Ion transport.Anion transport	21
14.1.2	Transport Overview.Ion transport.Cation transport	176
14.1.2.1	Transport Overview.Ion transport.Cation transport.Calcium transport	20
14.1.2.3	Transport Overview.Ion transport.Cation transport.Glutamate activated channel	8
14.1.2.5	Transport Overview.Ion transport.Cation transport.Magnesium transport	6
14.1.2.7	Transport Overview.Ion transport.Cation transport.Potassium transport	34
14.1.2.8	Transport Overview.Ion transport.Cation transport.Sodium transport	3
14.1.2.9	Transport Overview.Ion transport.Cation transport.V-ATPase	25
14.1.2.10	Transport Overview.Ion transport.Cation transport.V-PPase	12
14.3	Transport Overview.Gaseous transport	3
14.4	Transport Overview.Heavy metal ion transport	101
14.4.1	Transport Overview.Heavy metal ion transport.Ripening induced	47
14.4.2	Transport Overview.Heavy metal ion transport.Miscellaneous	30
14.5	Transport Overview.Malate transport	3
14.6	Transport Overview.Membrane proteins	141
14.6.1	Transport Overview.Membrane proteins.Aquaporins	21
14.6.1.1	Transport Overview.Membrane proteins.Aquaporins.MIP	3
14.6.1.2	Transport Overview.Membrane proteins.Aquaporins.NIP	2
14.6.1.3	Transport Overview.Membrane proteins.Aquaporins.PIP	9
14.6.1.4	Transport Overview.Membrane proteins.Aquaporins.SIP	1
14.6.1.5	Transport Overview.Membrane proteins.Aquaporins.TIP	6
14.6.2	Transport Overview.Membrane proteins.Multidrug transporters	85
14.6.2.1	Transport Overview.Membrane proteins.Multidrug transporters.ABC transporters	75
14.6.2.2	Transport Overview.Membrane proteins.Multidrug transporters.MATE transporters	7
14.6.2.3	Transport Overview.Membrane proteins.Multidrug transporters.MFS transporters	3
14.6.3	Transport Overview.Membrane proteins.Mithocondrial carrier proteins	33
**15**	**ATPase family associated with variousCellular activities**	**31**
**16**	**Regulation overview**	**1649**
16.1	Regulation overview.Hormone	279
16.1.1	Regulation overview.Hormone.GA	32
16.1.1.1	Regulation overview.Hormone.GA.Metabolism	14
16.1.1.2	Regulation overview.Hormone.GA.Perception/Signaling	3
16.1.1.3	Regulation overview.Hormone.GA.Responsive	14
16.1.1.4	Regulation overview.Hormone.GA.Transcription factor	1
16.1.2	Regulation overview.Hormone.Ethylene	86
16.1.2.1	Regulation overview.Hormone.Ethylene.Metabolism	14
16.1.2.2	Regulation overview.Hormone.Ethylene.Perception/Signaling	10
16.1.2.3	Regulation overview.Hormone.Ethylene.Responsive	11
16.1.2.4	Regulation overview.Hormone.Ethylene.Transcription factor	51
16.1.3	Regulation overview.Hormone.Auxin	93
16.1.3.1	Regulation overview.Hormone.Auxin.Metabolism	7

BIN	BIN Name	Genes
16.1.3.2	Regulation overview.Hormone.Auxin.Perception/Signaling	4
16.1.3.3	Regulation overview.Hormone.Auxin.Responsive	26
16.1.3.4	Regulation overview.Hormone.Auxin.Transport	12
16.1.3.5	Regulation overview.Hormone.Auxin.Transcription factor	44
16.1.4	Regulation overview.Hormone.ABA	26
16.1.4.1	Regulation overview.Hormone.ABA.Metabolism	2
16.1.4.2	Regulation overview.Hormone.ABA.Perception/Signaling	1
16.1.4.3	Regulation overview.Hormone.ABA.Responsive	19
16.1.4.4	Regulation overview.Hormone.ABA.Transcription factor	4
16.1.5	Regulation overview.Hormone.Brassinosteroid	13
16.1.5.1	Regulation overview.Hormone.Brassinosteroid.Metabolism	8
16.1.5.2	Regulation overview.Hormone.Brassinosteroid.Perception/Signaling	1
16.1.5.3	Regulation overview.Hormone.Brassinosteroid.Responsive	3
16.1.5.4	Regulation overview.Hormone.Brassinosteroid.Transcription factor	1
16.1.6	Regulation overview.Hormone.Cytokinin	12
16.1.6.1	Regulation overview.Hormone.Cytokinin.Metabolism	4
16.1.6.3	Regulation overview.Hormone.Cytokinin.Responsive	2
16.1.6.4	Regulation overview.Hormone.Cytokinin.Transcription factor	6
16.1.7	Regulation overview.Hormone.Jasmonic acid	7
16.1.7.1	Regulation overview.Hormone.Jasmonic acid.Metabolism	4
16.1.7.2	Regulation overview.Hormone.Jasmonic acid.Perception/Signaling	2
16.1.7.3	Regulation overview.Hormone.Jasmonic acid.Transcription factor	1
16.1.8	Regulation overview.Hormone.Phytosulfokine metabolism	6
16.1.9	Regulation overview.Hormone.Miscellaneous	4
16.2	Regulation overview.Nucleic acid metabolism	1370
16.2.1	Regulation overview.Nucleic acid metabolism.Chromatin packaging and remodeling	100
16.2.2	Regulation overview.Nucleic acid metabolism.DNA metabolism	138
16.2.3	Regulation overview.Nucleic acid metabolism.Nucleic acid binding	19
16.2.4	Regulation overview.Nucleic acid metabolism.RNA metabolism	237
16.2.5	Regulation overview.Nucleic acid metabolism.Transcription factor	738
16.2.5.1	Regulation overview.Nucleic acid metabolism.Transcription factor.Basic helix-loop-helix (bHLH)	50
16.2.5.2	Regulation overview.Nucleic acid metabolism.Transcription factor.Basic-leucine zipper (bZIP)	38
16.2.5.3	Regulation overview.Nucleic acid metabolism.Transcription factor.CO-like / B-box	12
16.2.5.4	Regulation overview.Nucleic acid metabolism.Transcription factor.DOF	15
16.2.5.5	Regulation overview.Nucleic acid metabolism.Transcription factor.E2F/DP	4
16.2.5.6	Regulation overview.Nucleic acid metabolism.Transcription factor.GATA	17
16.2.5.7	Regulation overview.Nucleic acid metabolism.Transcription factor.General transcription factor	17
16.2.5.8	Regulation overview.Nucleic acid metabolism.Transcription factor.GRAS transcription factor	15
16.2.5.9	Regulation overview.Nucleic acid metabolism.Transcription factor.Heat shock transcription factor	6
16.2.5.10	Regulation overview.Nucleic acid metabolism.Transcription factor.Homeobox domain	48
16.2.5.11	Regulation overview.Nucleic acid metabolism.Transcription factor.MADS-box transcription factor	29
16.2.5.12	Regulation overview.Nucleic acid metabolism.Transcription factor.MISC	26
16.2.5.13	Regulation overview.Nucleic acid metabolism.Transcription factor.Myb transcription factor	94
16.2.5.14	Regulation overview.Nucleic acid metabolism.Transcription factor.NAC transcription factor	26
16.2.5.15	Regulation overview.Nucleic acid metabolism.Transcription factor.Pathogenesis-related transcription factor	15
16.2.5.16	Regulation overview.Nucleic acid metabolism.Transcription factor.Plant homeodomain (PHD) finger	10
16.2.5.17	Regulation overview.Nucleic acid metabolism.Transcription factor.SBP-box	16
16.2.5.18	Regulation overview.Nucleic acid metabolism.Transcription factor.Sigma factor	7
16.2.5.19	Regulation overview.Nucleic acid metabolism.Transcription factor.TCP transcription factor	6
16.2.5.20	Regulation overview.Nucleic acid metabolism.Transcription factor.WRKY	39
16.2.5.21	Regulation overview.Nucleic acid metabolism.Transcription factor.YABBY transcription factor	5
16.2.5.22	Regulation overview.Nucleic acid metabolism.Transcription factor.Zinc finger trancription factor	59
16.2.5.23	Regulation overview.Nucleic acid metabolism.Transcription factor.Zinc finger, C2H2-type	46
16.2.5.24	Regulation overview.Nucleic acid metabolism.Transcription factor.Zinc finger, C3HC4-type	97
16.2.5.25	Regulation overview.Nucleic acid metabolism.Transcription factor.Zinc finger, CCCH-type	19

BIN	BIN Name	Genes
16.2.5.26	Regulation overview.Nucleic acid metabolism.Transcription factor.Zinc finger, CCHC-type	22
16.2.6	Regulation overview.Nucleic acid metabolism.mRNA metabolism	101
16.2.7	Regulation overview.Nucleic acid metabolism.rRNA metabolism	13
16.2.8	Regulation overview.Nucleic acid metabolism.tRNA metabolism	8
**17**	**Cytochrome P450**	**120**
**18**	**Leucine-rich domain protein**	**48**
**19**	**Secondary metabolism**	**364**
19.1	Secondary metabolism.Alkaloids	39
19.2	Secondary metabolism.Amines	4
19.3	Secondary metabolism.Aroma	9
19.4	Secondary metabolism.Phenylpropanoids	179
19.4.1	Secondary metabolism.Phenylpropanoids.Flavonoids	127
19.4.1.1	Secondary metabolism.Phenylpropanoids.Flavonoids.Anthocyanin biosyhthesis	33
19.4.1.2	Secondary metabolism.Phenylpropanoids.Flavonoids.Isoflavonoids	12
19.4.1.3	Secondary metabolism.Phenylpropanoids.Flavonoids.Flavonoids	82
19.4.2	Secondary metabolism.Phenylpropanoids.Phytoalexins	35
19.4.4	Secondary metabolism.Phenylpropanoids.General pathway	17
19.5	Secondary metabolism.Polyamines	23
19.6	Secondary metabolism.Terpenoids	80
19.7	Secondary metabolism.Miscellaneous	30
**20**	**Hypothetical protein**	**2806**
**21**	**Unclassified**	**4143**
**22**	**Phosphate metabolism**	**47**
22.1	Phosphate metabolism.General metabolism	30
22.2	Phosphate metabolism.Phosphate transport	17
**23**	**Nitrogen metabolism**	**63**
23.1	Nitrogen metabolism.General metabolism	50
23.2	Nitrogen metabolism.Nitrogen transport	13
**24**	**Sulfate metabolism**	**22**
24.1	Sulfate metabolism.General metabolism	11
24.2	Sulfate metabolism.Sulfate transport	11
**25**	**Cytoskeleton organization and biogenesis**	**99**
**26**	**Coenzyme and prosthetic group metabolism**	**74**
26.1	Coenzyme and prosthetic group metabolism.Ascorbic acid	8
26.2	Coenzyme and prosthetic group metabolism.Biotin	3
26.3	Coenzyme and prosthetic group metabolism.Cobalamin	2
26.4	Coenzyme and prosthetic group metabolism.Folic acid	10
26.5	Coenzyme and prosthetic group metabolism.Heme	2
26.6	Coenzyme and prosthetic group metabolism.Lipoic acid	4
26.7	Coenzyme and prosthetic group metabolism.Menaquinone	1
26.8	Coenzyme and prosthetic group metabolism.Molydopterin	2
26.9	Coenzyme and prosthetic group metabolism.NAD	2
26.10	Coenzyme and prosthetic group metabolism.Panthothenic acid	7
26.12	Coenzyme and prosthetic group metabolism.Plastoquinone	3
26.13	Coenzyme and prosthetic group metabolism.Pyridoxine	8
26.14	Coenzyme and prosthetic group metabolism.Riboflavin	7
26.15	Coenzyme and prosthetic group metabolism.Thiamine	8
26.16	Coenzyme and prosthetic group metabolism.Tocopherol	2
26.17	Coenzyme and prosthetic group metabolism.Transport	4
26.18	Coenzyme and prosthetic group metabolism.Miscellaneous	1
**27**	**Nodulin**	**35**

BIN	BIN Name	Genes
**28**	**Ripening induced**	**14**
**29**	**Storage proteins**	**62**
**30**	**Nucleotide metabolism**	**87**
30.1	Nucleotide metabolism.Pyrimidine	12
30.2	Nucleotide metabolism.Purine	17
30.3	Nucleotide metabolism.Miscellaneous	49
30.4	Nucleotide metabolism.Transport	9
**31**	**Metabolism**	**146**
31.1	Metabolism.2OG-Fe(II) oxygenase superfamily	22
31.2	Metabolism.Alcohol dehydrogenase superfamily	28
31.3	Metabolism.Aldehyde dehydrogenase superfamily	12
31.4	Metabolism.AMP-dependent synthetase and ligase	6
31.5	Metabolism.Oxireductase family	25
31.6	Metabolism.Miscellaneous	53
**32**	**Organic acid metabolism**	**7**
32.1	Organic acid metabolism.Malic acid	3
32.2	Organic acid metabolism.Tartaric acid	4
**33**	**Pentatricopeptide repeat domain**	**27**
**34**	**Tetratricopeptide repeat domain**	**8**
**35**	**WD-40 domain**	**77**
**36**	**Zinc finger domain**	**53**
