# Supplementary material for: Transcriptome changes in grapevine (Vitis vinifera L.) cv. Malbec leaves induced by ultraviolet-B radiation
Source: BMC Plant Biol. 2010 Oct 20;10:224. doi: 10.1186/1471-2229-10-224 (PMC3017828; doi:10.1186/1471-2229-10-224)
Supplement: Additional file 6 — Up-regulated functional classes High UV-B. PDF file showing the full list of differentially expressed genes included in the up-regulated functional categories under high UV-B radiation described in Table 1. Positive and negative symbols represent higher or lower transcript levels under UV-B light compared with the control, respectively. [file 1471-2229-10-224-S6.PDF]

Probe set ID	*P* value	Diff. expression	Annotation
Biotic stress			
CA814441	2,22E-16	+	Q9FQY9 Avr9 Cf-9 rapidly elicited protein 75 related cluster
GSVIVP00020853001	2,22E-16	+	Q9FQZ3 Avr9 Cf-9 rapidly elicited protein 231 precursor related cluster
GSVIVP00024738001	2,22E-16	+	Q9LID5 Disease Resistance response protein-like related cluster
GSVIVP00016194001	1,29E-14	+	Q1S9M3 Lipase, active site related cluster
GSVIVP00024745001	9,90E-12	+	Q9C523 dirigent protein, putative related cluster
GSVIVP00011194001	6,00E-11	+	Q8S8Z5 Syringolide-induced protein B13-1-1 related cluster
GSVIVP00037207001	1,21E-10	+	Q5MJW2 Avr9 Cf-9 rapidly elicited protein 102 related cluster
GSVIVP00029253001	1,55E-10	+	Q9FQ21 Putative Hs1pro-1-like receptor related cluster
GSVIVP00016176001	1,56E-09	+	Q1S9M3 Lipase, active site related cluster
GSVIVP00024741001	2,06E-09	+	Q9LID5 Disease Resistance response protein-like related cluster
CF414894	1,38E-08	+	Q9FQZ5 Avr9 Cf-9 rapidly elicited protein 169 related cluster
GSVIVP00031406001	2,78E-08	+	Q8S902 Syringolide-induced protein 19-1-5 related cluster
GSVIVP00037835001	4,45E-08	+	O64757 Putative Disease Resistance protein related cluster
GSVIVP00005104001	4,64E-08	+	Q1RVF4 dirigent-like protein related cluster
GSVIVP00021999001	1,99E-07	+	Q5DMW5 MRGH12 related cluster
VVTU23043_at	1,47E-06	+	Q6XWA6 Resistance protein Sorb5 related cluster
GSVIVP00003294001	1,81E-05	+	Q1SH60 Disease Resistance protein; AAA ATPase related cluster
GSVIVP00034475001	8,96E-05	+	Q8H6S3 NBS-LRR type Disease Resistance protein related cluster
GSVIVP00027396001	1,42E-04	+	Q6T3R2 NDR1-like protein related cluster
GSVIVP00032006001	1,42E-04	+	Q71RI6 Resistance protein related cluster
GSVIVP00034443001	1,42E-04	+	Q8H6R0 NBS-LRR type Disease Resistance protein related cluster
TC71212	1,98E-04	+	Q9LKG8 TIP related cluster
GSVIVP00031398001	2,67E-04	+	Q8S902 Syringolide-induced protein 19-1-5 related cluster
TC64868	2,67E-04	+	Q6XZH4 Nematode resistance-like protein related cluster
GSVIVP00026569001	3,89E-04	+	Q19PN8 TIR-NBS-LRR type Disease Resistance protein related cluster
GSVIVP00010513001	5,94E-04	+	Q949G9 HcrVf1 protein related cluster
GSVIVP00003766001	6,28E-04	+	Q6L3K6 Resistance complex protein I2C-2, putative related cluster
GSVIVP00026433001	6,28E-04	+	Q1RU52 Disease Resistance protein; AAA ATPase related cluster
GSVIVP00021538001	8,05E-04	+	Q1ZZ69 Secoisolariciresinol dehydrogenase related cluster

Probe set ID	*P* value	Diff. expression	Annotation
TC67734	8,50E-04	+	Q6XWA3 Resistance protein Tsu4 related cluster
GSVIVP00017878001	1,08E-03	+	Q84XG6 Erwinia induced protein 2 related cluster
GSVIVP00033742001	1,24E-03	+	Q9SX38 Putative Disease Resistance protein At1g50180 related cluster
GSVIVP00038789001	1,65E-03	+	Q6T3R3 Bacterial spot Disease Resistance protein 4 related cluster
GSVIVP00030420001	1,92E-03	+	Q5ZE78 Putative rust resistance kinase Lr10 related cluster
GSVIVP00034601001	1,92E-03	+	Q1SW19 Disease Resistance protein related cluster
GSVIVP00038293001	2,29E-03	+	Q19PN9 NBS type Disease Resistance protein related cluster
GSVIVP00002451001	2,35E-03	+	Q9FQZ4 Avr9 Cf-9 rapidly elicited protein 194 related cluster
CD713658	2,87E-03	+	Q40392 TMV Resistance protein N related cluster
GSVIVP00007023001	4,23E-03	+	Q19PL2 TIR-NBS-LRR-TIR type Disease Resistance protein related cluster
GSVIVP00026768001	4,54E-03	+	Q6XW51 Resistance protein RPP8-like protein related cluster
CF372116	5,87E-03	+	Q1SY04 Disease Resistance protein; AAA ATPase related cluster
TC60450	6,15E-03	+	P93384 Nicotiana tabacum ORF related cluster
GSVIVP00017750001	7,05E-03	+	Q2L360 Putative CC-NBS-LRR Resistance protein related cluster
GSVIVP00035452001	9,84E-03	+	Q19PN7 NBS type Disease Resistance protein related cluster
GSVIVP00028707001	1,12E-02	+	Q19PN7 NBS type Disease Resistance protein related cluster
GSVIVP00025008001	1,22E-02	+	Q8LF94 Avr9 Cf-9 rapidly elicited protein 231 related cluster
GSVIVP00013337001	1,27E-02	+	O49621 MLO-like protein 1 related cluster
GSVIVP00010479001	1,30E-02	+	Q7XA40 Putative Disease Resistance protein RGA3 related cluster
VVTU20698_at	1,93E-02	+	Q19PL5 NBS-LRR type Disease Resistance protein related cluster
GSVIVP00008243001	3,03E-02	+	Q1SY04 Disease Resistance protein; AAA ATPase related cluster
VVTU35131_at	3,27E-02	+	Q8LID8 Cyst nematode Resistance protein-like protein related cluster
GSVIVP00019559001	3,33E-02	+	Q84QE1 Avr9 Cf-9 rapidly elicited protein 189 related cluster
GSVIVP00003754001	3,39E-02	+	Q84TQ8 Truncated NBS-LRR resistance-like protein isoforms JA68, JA76, and JA80 related cluster
GSVIVP00035825001	3,72E-02	+	Q84QD7 Avr9 Cf-9 rapidly elicited protein 276 related cluster
TC62975	3,72E-02	+	Q6URA2 TIR-NBS-LRR type R protein 7 related cluster
GSVIVP00000261001	3,79E-02	+	Q84KB4 MRGH5 related cluster
GSVIVP00014747001	3,86E-02	+	Q1S2K5 Disease Resistance protein; AAA ATPase related cluster
GSVIVP00003283001	4,23E-02	+	Q1T0I4 Disease Resistance protein related cluster
GSVIVP00035415001	4,47E-02	-	Q71RI4 Resistance protein related cluster
GSVIVP00035146001	4,31E-02	-	Q945D8 Putative gamma-thionin related cluster

Probe set ID	*P* value	Diff. expression	Annotation
GSVIVP00028668001	3,59E-02	-	Q1RU52 Disease Resistance protein; AAA ATPase related cluster
GSVIVP00026851001	2,75E-02	-	Q5XNL4 Resistance protein-like protein related cluster
GSVIVP00025582001	2,05E-02	-	Q1S6X1 Disease Resistance protein; Short-chain dehydrogenase reductase SDR related cluster
GSVIVP00003147001	1,36E-02	-	Q4TVR0 NRG1 related cluster
GSVIVP00036207001	5,87E-03	-	Q84XG7 Erwinia induced protein 1 related cluster
GSVIVP00028467001	4,65E-03	-	Q2N1D8 CMV 1a interActinG protein 2 related cluster
GSVIVP00006180001	2,18E-03	-	Q6TAF9 Blight Resistance protein SH10 related cluster
GSVIVP00026845001	1,49E-03	-	Q6XWB7 Resistance protein Cvi2 related cluster
GSVIVP00035399001	7,62E-04	-	Q1S0D0 Glyoxalase bleomycin Resistance protein dioxygenase related cluster
GSVIVP00004380001	6,38E-06	-	Q41495 STS14 protein precursor related cluster
GSVIVP00006779001	1,38E-07	-	Q75VK7 CC-NB-LRR protein related cluster
CB350096	4,09E-08	-	Q8LT03 Leaf thionin Asthi1 related cluster
Ethylene - Transcription Factors			
GSVIVP00009541001	2,22E-16	+	O80337 ethylene-responsive transcription factor 1A related cluster
GSVIVP00014231001	2,22E-16	+	Q6RZW8 Putative ethylene response factor 4 related cluster
GSVIVP00014263001	2,22E-16	+	Q6RZW8 Putative ethylene response factor 4 related cluster
GSVIVP00014265001	2,22E-16	+	Q6RZW8 Putative ethylene response factor 4 related cluster
GSVIVP00014244001	7,55E-15	+	Q6RZW8 Putative ethylene response factor 4 related cluster
GSVIVP00014260001	4,48E-12	+	Q6RZW8 Putative ethylene response factor 4 related cluster
TC53821	1,63E-10	+	Q9LW49 ethylene-responsive transcription factor 4 related cluster
CB342852	3,75E-09	+	Q6RZW8 Putative ethylene response factor 4 related cluster
GSVIVP00027730001	1,44E-07	+	Q6TKQ3 Putative ethylene response factor ERF3b related cluster
GSVIVP00022076001	2,64E-07	+	Q3L8J0 CBF-like transcription factor related cluster
GSVIVP00023866001	2,77E-06	+	Q9SXS8 ethylene-responsive transcription factor 3 related cluster
GSVIVP00015361001	7,89E-06	+	Q8LDL8 TINY-like protein related cluster
GSVIVP00003062001	2,54E-05	+	Q6TKQ4 Putative ethylene response factor ERF3a related cluster
GSVIVP00002438001	5,17E-04	+	Q9C7W2 AP2-containing DNA-binding protein; 51686-52693 related cluster
GSVIVP00009539001	1,24E-03	+	Q6RZW7 Putative ethylene response factor 5 related cluster
GSVIVP00029809001	4,23E-03	+	O49747 ERT2 protein related cluster
GSVIVP00030292001	2,40E-02	+	Q4G3H5 RAV transcription factor related cluster

Probe set ID	*P* value	Diff. expression	Annotation
GSVIVP00019092001	2,86E-02	+	Q9SST7 ethylene-responsive element binding protein1 homolog related cluster
GSVIVP00025602001	2,65E-02	-	Q8GWK2 Putative AP2 domain transcription factor related cluster
GSVIVP00012449001	1,03E-03	-	Q0Q097 AP2 EREBP transcription factor AINTEGUMENTA-like related cluster
Phenylpropanoids general pathway			
GSVIVP00017017001	2,66E-15	+	Q9M4U0 Cinnamate 4-hydroxylase CYP73 related cluster
GSVIVP00013930001	1,22E-11	+	X75967 V.vinifera PAL mRNA for phenylalanine ammonia lyase
GSVIVP00023211001	7,72E-11	+	O64963 Phenylalanine ammonia-lyase 1 related cluster
GSVIVP00013936001	4,11E-09	+	O80406 Phenylalanine ammonia-lyase related cluster
GSVIVP00031383001	1,99E-07	+	O24145 4-coumarate--CoA ligase 1 related cluster
GSVIVP00018175001	9,02E-03	+	Q94C45 Phenylalanine ammonia-lyase 1 related cluster
GSVIVP00023932001	9,84E-03	+	Q3HM04 Cinnamate-4-hydroxylse related cluster
GSVIVP00002825001	3,46E-02	+	Q2YHM9 Caffeoyl-CoA O-methyltransferase related cluster
Heat Shock Proteins			
GSVIVP00009608001	8,97E-11	+	O80432 Mitochondrial small Heat shock protein related cluster
GSVIVP00009607001	3,75E-09	+	O80432 Mitochondrial small Heat shock protein related cluster
GSVIVP00034024001	1,38E-07	+	Q94EN7 Small heat stress protein class CIII related cluster
GSVIVP00002550001	1,69E-07	+	P51819 Heat shock protein 83 related cluster
GSVIVP00032242001	7,09E-07	+	Q9XGS6 Cytosolic class II low molecular weight Heat shock protein related cluster
GSVIVP00018481001	7,67E-07	+	P22954 Heat shock cognate 70 kDa protein 2 related cluster
GSVIVP00018812001	1,08E-06	+	Q9SXK8 Heat shock factor related cluster
GSVIVP00006687001	1,58E-05	+	Q03686 Luminal-bindinG protein 8 related cluster
GSVIVP00019488001	2,14E-05	+	Q9SXK9 Heat shock factor related cluster
GSVIVP00030478001	3,20E-05	+	Q8H2B1 DnaJ-like protein related cluster
GSVIVP00023801001	1,26E-04	+	Q6VBB2 Heat shock factor RHSF5 related cluster
GSVIVP00035360001	1,42E-04	+	Q39819 HSP22.3 related cluster
GSVIVP00003050001	2,07E-03	+	Q1RY14 Heat shock protein DnaJ related cluster
GSVIVP00035112001	2,07E-03	+	Q8L470 Small Heat shock protein related cluster
GSVIVP00009601001	2,13E-03	+	Q39079 chaperone protein DnaJ 13 related cluster
GSVIVP00024357001	2,54E-03	+	P29357 Chloroplast envelope membrane 70 kDa Heat shock-related protein related cluster
GSVIVP00032243001	2,80E-03	+	Q9XGS6 Cytosolic class II low molecular weight Heat shock protein related cluster

Probe set ID	*P* value	Diff. expression	Annotation
CB342902	3,58E-03	+	Q9SWB5 seed maturation protein PM37 related cluster
GSVIVP00037272001	3,84E-03	+	Q9SWB5 seed maturation protein PM37 related cluster
GSVIVP00032192001	8,63E-03	+	P41152 Heat shock factor protein HSF30 related cluster
GSVIVP00018308001	8,82E-03	+	Q39929 18.6 kDa Heat-shock protein related cluster
GSVIVP00037726001	9,63E-03	+	P51819 Heat shock protein 83 related cluster
GSVIVP00002118001	9,84E-03	+	Q1SSK6 Heat shock protein HSP20 related cluster
CB349128	1,57E-02	+	O82149 Low-molecular-weight Heat shock protein related cluster
CF405189	2,09E-02	+	Q9SWB5 seed maturation protein PM37 related cluster
GSVIVP00024197001	2,65E-02	+	O82149 Low-molecular-weight Heat shock protein related cluster
GSVIVP00033437001	2,86E-02	+	P55737 Heat shock protein 81-2 related cluster
GSVIVP00017271001	3,86E-02	+	P34893 10 kDa chaperonin related cluster
GSVIVP00018344001	4,71E-02	+	Q6WHC0 Chloroplast small Heat shock protein class I related cluster
GSVIVP00020058001	1,97E-02	-	O82623 T9A4.1 protein related cluster
GSVIVP00025539001	5,87E-03	-	Q1SHY3 Heat shock protein DnaJ related cluster
GSVIVP00033001001	3,75E-03	-	Q9FH28 chaperone protein DnaJ 49 related cluster
TC65028	1,87E-03	-	Q688X8 putative Heat shock protein, HSP40 related cluster
GSVIVP00024899001	9,47E-04	-	O23230 Trichohyalin like protein related cluster
Phytoalexins			
AJ862932	2,22E-16	+	AY670143 Vitis vinifera clone 357844_R1 stilbene synthase mRNA, partial cds.
GSVIVP00004049001	2,22E-16	+	AY670089 Vitis vinifera clone 325905_S3 stilbene synthase mRNA, partial cds.
GSVIVP00031875001	2,22E-16	+	AY670148 Vitis vinifera clone 357851_M2 stilbene synthase mRNA, partial cds.
GSVIVP00010117001	4,22E-15	+	DQ366302 Vitis vinifera resveratrol synthase (STS2) mRNA, complete cds.
GSVIVP00013875001	7,08E-09	+	Q6E593 Benzoyl coenzyme A: benzyl alcohol benzoyl transferase related cluster
GSVIVP00010116001	2,45E-05	+	AF274281 Vitis vinifera resveratrol synthase (RS1) mRNA, complete cds.
GSVIVP00009234001	2,71E-05	+	P28343 stilbene synthase 1 related cluster
GSVIVP00031885001	3,31E-05	+	AY670213 Vitis vinifera clone 361641_M1 stilbene synthase mRNA, partial cds.
GSVIVP00017980001	9,22E-04	+	Q94CD1 Putative N-hydroxycinnamoyl benzoyltransferase related cluster
GSVIVP00017179001	1,12E-02	+	O64470 Putative anthranilateN-hydroxycinnamoyl benzoyltransferase related cluster
GSVIVP00023980001	2,26E-02	+	Q1RZ42 Transferase related cluster
GSVIVP00031153001	4,99E-03	-	Q10D12 Transferase family protein, expressed related cluster

Probe set ID	*P* value	Diff. expression	Annotation
GSVIVP00036656001	2,18E-03	-	Q0PI14 Resveratrol hydroxycinnamic acid O-Glucosyltransferase related cluster
NAC Transcription Factors			
GSVIVP00019287001	1,63E-10	+	Q6RH27 NAC domain protein related cluster
GSVIVP00036931001	3,27E-09	+	Q52QR5 NAC domain protein NAC1 related cluster
GSVIVP00018104001	1,20E-02	+	Q8LRL7 Nam-like protein 8 related cluster
GSVIVP00011769001	1,30E-02	+	Q50J79 NAM-like protein related cluster
GSVIVP00014676001	1,64E-02	+	Q9M9N8 NAM-like protein related cluster
CB343368	1,97E-02	+	O81790 NAM CUC2 -like protein related cluster
GSVIVP00026201001	2,31E-02	+	O81790 NAM CUC2 -like protein related cluster
TC65987	2,60E-02	+	Q8L8G0 Nam-like protein 1 related cluster
GSVIVP00011870001	4,63E-02	+	Q52QR0 NAC domain protein NAC6 related cluster
GSVIVP00017686001	2,92E-02	-	Q8LKN9 Nam-like protein 15 related cluster
WRKY Transcription Factors			
GSVIVP00027001001	2,22E-16	+	Q6R7N3 Putative WRKY transcription factor 30 related cluster
GSVIVP00037648001	2,22E-16	+	Q6RZW9 Putative WRKY4 transcription factor related cluster
GSVIVP00002446001	2,66E-15	+	Q6R7N3 Putative WRKY transcription factor 30 related cluster
GSVIVP00031317001	2,14E-08	+	Q9SXP4 DNA-bindinG protein NtWRKY3 related cluster
GSVIVP00023994001	9,54E-05	+	Q40090 SPF1 protein related cluster
GSVIVP00023389001	1,22E-04	+	Q9SXP4 DNA-bindinG protein NtWRKY3 related cluster
DY473668	2,07E-03	+	Q1EPJ3 DNA-binding WRKY domain-containinG protein related cluster
GSVIVP00026902001	4,43E-03	+	O04609 WRKY transcription factor 22 related cluster
GSVIVP00035835001	2,05E-02	+	Q3SAJ9 WRKY-A1244 related cluster
CA809370	4,08E-02	+	Q9FXS1 WRKY transcription factor NtEIG-D48 related cluster
GSVIVP00004133001	5,61E-03	-	Q1SS80 DNA-binding WRKY related cluster
Sugar binding proteins			
GSVIVP00023009001	2,22E-16	+	Q5ZEM1 Putative lectin 2 related cluster
GSVIVP00037808001	1,56E-04	+	Q1RYJ4 Protein kinase; Curculin-like (Mannose-binding) lectin; Apple- like related cluster
GSVIVP00036115001	6,28E-04	+	Q1T694 D-galactoside L-rhamnose binding SUEL lectin; Galactose-binding like related cluster
CF209648	1,78E-03	+	Q1RYJ4 Protein kinase; Curculin-like (Mannose-binding) lectin; Apple- like related cluster

Probe set ID	*P* value	Diff. expression	Annotation
GSVIVP00027507001	2,66E-03	+	Q40100 Secreted glycoprotein 3 related cluster
GSVIVP00009976001	5,61E-03	+	P17840 S-locus-specific glycoprotein S13 precursor related cluster
TC56382	1,42E-02	+	Q39436 SIEP1L protein precursor related cluster
Phathogenesis related Transcription Factors			
CB348242	4,08E-02	+	Q1SHW5 Pathogenesis-related transcriptional factor and ERF related cluster
GSVIVP00003018001	1,53E-05	+	O04681 Pathogenesis-related genes transcriptional activator PTI5 related cluster
GSVIVP00007523001	6,16E-12	+	P93392 S25-XP1 DNA bindinG protein related cluster
GSVIVP00023927001	6,00E-11	+	O04682 Pathogenesis-related genes transcriptional activator PTI6 related cluster
GSVIVP00037958001	6,58E-03	+	Q1SMR9 Pathogenesis-related transcriptional factor and ERF related cluster
