# Supplementary material for: Transcriptome changes in grapevine (Vitis vinifera L.) cv. Malbec leaves induced by ultraviolet-B radiation
Source: BMC Plant Biol. 2010 Oct 20;10:224. doi: 10.1186/1471-2229-10-224 (PMC3017828; doi:10.1186/1471-2229-10-224)
Supplement: Additional file 7 — Up-regulated functional classes Low UV-B. PDF file showing the full list of differentially expressed genes included in the up-regulated functional categories under low UV-B radiation described in Table 2. Positive and negative symbols represent higher or lower transcript levels under UV-B light compared with the control, respectively. [file 1471-2229-10-224-S7.PDF]

Probe set ID	*P* value	Diff. expression	Annotation
Phenylpropanoids general pathway			
GSVIVP00017017001	1,64E-08	+	Q9M4U0 Cinnamate 4-hydroxylase CYP73 related cluster
GSVIVP00002825001	1,34E-06	+	Q2YHM9 Caffeoyl-CoA O-methyltransferase related cluster
GSVIVP00023211001	3,17E-06	+	O64963 Phenylalanine ammonia-lyase 1 related cluster
GSVIVP00013930001	5,94E-06	+	X75967 V.vinifera PAL mRNA for phenylalanine ammonia lyase
GSVIVP00031383001	1,04E-03	+	O24145 4-coumarate--CoA ligase 1 related cluster
GSVIVP00013936001	8,98E-03	+	O80406 Phenylalanine ammonia-lyase related cluster
GSVIVP00018175001	1,16E-02	+	Q94C45 Phenylalanine ammonia-lyase 1 related cluster
GSVIVP00038153001	2,36E-02	+	Q6L5E8 Putative cinnamoyl CoA reductase related cluster
GSVIVP00023932001	2,95E-02	+	Q3HM04 Cinnamate-4-hydroxylse related cluster
WRKY Transcription Factors			
GSVIVP00032689001	2,07E-13	+	Q6RZW9 Putative WRKY4 transcription factor related cluster
GSVIVP00033165001	1,33E-08	+	Q2PJR6 WRKY54 related cluster
GSVIVP00027001001	8,18E-08	+	Q6R7N3 Putative WRKY transcription factor 30 related cluster
GSVIVP00031317001	7,88E-05	+	Q9SXP4 DNA-bindinG protein NtWRKY3 related cluster
GSVIVP00037648001	1,00E-04	+	Q6RZW9 Putative WRKY4 transcription factor related cluster
GSVIVP00003403001	2,21E-04	+	Q1EPJ3 DNA-binding WRKY domain-containinG protein related cluster
GSVIVP00023994001	2,93E-04	+	Q40090 SPF1 protein related cluster
GSVIVP00035835001	5,66E-04	+	Q3SAJ9 WRKY-A1244 related cluster
GSVIVP00002446001	1,21E-03	+	Q6R7N3 Putative WRKY transcription factor 30 related cluster
DY473668	6,08E-03	+	Q1EPJ3 DNA-binding WRKY domain-containinG protein related cluster
GSVIVP00028232001	3,41E-02	+	Q9ZSI7 Probable WRKY transcription factor 47 related cluster
Abscisic acid metabolism			
GSVIVP00001184001	5,74E-06	+	Q3HNF4 ABA 8 -hydroxylase CYP707A1 related cluster
GSVIVP00036599001	4,34E-03	+	Q8LF37 Cytochrome P450, putative related cluster
Ethylene - Transcription Factors			
GSVIVP00009541001	2,22E-15	+	O80337 ethylene-responsive transcription factor 1A related cluster
TC53821	8,30E-09	+	Q9LW49 ethylene-responsive transcription factor 4 related cluster
GSVIVP00014244001	1,45E-08	+	Q6RZW8 Putative ethylene response factor 4 related cluster

Probe set ID	*P* value	Diff. expression	Annotation
GSVIVP00009539001	6,30E-04	+	Q6RZW7 Putative ethylene response factor 5 related cluster
GSVIVP00030292001	9,35E-04	+	Q4G3H5 RAV transcription factor related cluster
GSVIVP00003062001	1,80E-03	+	Q6TKQ4 Putative ethylene response factor ERF3a related cluster
GSVIVP00022076001	2,36E-03	+	Q3L8J0 CBF-like transcription factor related cluster
GSVIVP00023866001	4,87E-03	+	Q9SXS8 ethylene-responsive transcription factor 3 related cluster
GSVIVP00027730001	3,29E-02	+	Q6TKQ3 Putative ethylene response factor ERF3b related cluster
Phytoalexins			
GSVIVP00031875001	2,22E-15	+	AY670148 Vitis vinifera clone 357851_M2 stilbene synthase mRNA, partial cds.
GSVIVP00004049001	6,44E-15	+	AY670089 Vitis vinifera clone 325905_S3 stilbene synthase mRNA, partial cds.
AJ862932	2,25E-10	+	AY670143 Vitis vinifera clone 357844_R1 stilbene synthase mRNA, partial cds.
GSVIVP00010117001	4,56E-07	+	DQ366302 Vitis vinifera resveratrol synthase (STS2) mRNA, complete cds.
GSVIVP00013875001	1,99E-03	+	Q6E593 Benzoyl coenzyme A: benzyl alcohol benzoyl transferase related cluster
GSVIVP00009234001	1,63E-02	+	P28343 stilbene synthase 1 related cluster
GSVIVP00009228001	3,47E-02	+	AY670138 Vitis vinifera clone 357836_G2 stilbene synthase mRNA, partial cds.
GSVIVP00010116001	4,60E-02	+	AF274281 Vitis vinifera resveratrol synthase (RS1) mRNA, complete cds.
GSVIVP00031153001	8,25E-03	-	Q10D12 Transferase family protein, expressed related cluster
NAC Transcription Factors			
GSVIVP00036931001	9,92E-10	+	Q52QR5 NAC domain protein NAC1 related cluster
GSVIVP00019287001	1,78E-09	+	Q6RH27 NAC domain protein related cluster
GSVIVP00003042001	2,85E-04	+	Q5QMP4 No apical meristem (NAM) protein-like related cluster
GSVIVP00014676001	1,06E-02	+	Q9M9N8 NAM-like protein related cluster
TC65987	1,11E-02	+	Q8L8G0 Nam-like protein 1 related cluster
GSVIVP00020998001	2,10E-02	+	Q9FLJ2 NAM (No apical meristem)-like protein related cluster
ATPase family			
GSVIVP00020826001	3,36E-07	+	Q1S520 AAA ATPase related cluster
GSVIVP00020821001	2,30E-03	+	Q1S528 AAA ATPase related cluster
GSVIVP00034310001	2,72E-03	+	Q1T3I2 AAA ATPase related cluster
GSVIVP00037796001	5,95E-03	+	Q1SK92 AAA ATPase, central region; DEAD DEAH box helicase, N-terminal related cluster
GSVIVP00035070001	6,94E-03	+	Q9FN75 AAA-type ATPase-like protein related cluster

Probe set ID	*P* value	Diff. expression	Annotation
GSVIVP00004974001	1,66E-02	+	Q1SVV2 AAA ATPase related cluster
Biotic stress			
GSVIVP00029253001	9,06E-10	+	Q9FQ21 Putative Hs1pro-1-like receptor related cluster
TC71212	3,04E-09	+	Q9LKG8 TIP related cluster
GSVIVP00037207001	5,74E-06	+	Q5MJW2 Avr9 Cf-9 rapidly elicited protein 102 related cluster
GSVIVP00024745001	1,61E-04	+	Q9C523 dirigent protein, putative related cluster
GSVIVP00011194001	7,01E-04	+	Q8S8Z5 Syringolide-induced protein B13-1-1 related cluster
GSVIVP00016194001	9,11E-04	+	Q1S9M3 Lipase, active site related cluster
GSVIVP00016176001	1,15E-03	+	Q1S9M3 Lipase, active site related cluster
GSVIVP00005104001	1,68E-03	+	Q1RVF4 dirigent-like protein related cluster
GSVIVP00003759001	1,90E-03	+	Q5MJV5 Avr9 Cf-9 rapidly elicited protein 256 related cluster
GSVIVP00003294001	2,60E-03	+	Q1SH60 Disease Resistance protein; AAA ATPase related cluster
GSVIVP00024741001	2,93E-03	+	Q9LID5 Disease Resistance response protein-like related cluster
GSVIVP00024738001	3,70E-03	+	Q9LID5 Disease Resistance response protein-like related cluster
CF414894	3,87E-03	+	Q9FQZ5 Avr9 Cf-9 rapidly elicited protein 169 related cluster
GSVIVP00026569001	4,44E-03	+	Q19PN8 TIR-NBS-LRR type Disease Resistance protein related cluster
TC54704	5,95E-03	+	Q6RX30 RPP13-like protein related cluster
GSVIVP00037835001	7,57E-03	+	O64757 Putative Disease Resistance protein related cluster
GSVIVP00038789001	1,83E-02	+	Q6T3R3 Bacterial spot Disease Resistance protein 4 related cluster
GSVIVP00007023001	2,02E-02	+	Q19PL2 TIR-NBS-LRR-TIR type Disease Resistance protein related cluster
GSVIVP00026605001	2,27E-02	+	AF365879 Vitis vinifera clone GLP1-12 Resistance protein gene, partial cds.
GSVIVP00028994001	2,40E-02	+	AY159555 Vitis vinifera putative hypersensitive-induced response protein (HIR) mRNA, partial cds.
GSVIVP00035825001	2,54E-02	+	Q84QD7 Avr9 Cf-9 rapidly elicited protein 276 related cluster
GSVIVP00002451001	3,66E-02	+	Q9FQZ4 Avr9 Cf-9 rapidly elicited protein 194 related cluster
CB350096	4,21E-02	+	Q8LT03 Leaf thionin Asthi1 related cluster
GSVIVP00017878001	4,29E-02	+	Q84XG6 Erwinia induced protein 2 related cluster
GSVIVP00021538001	4,68E-02	+	Q1ZZ69 Secoisolariciresinol dehydrogenase related cluster
GSVIVP00025894001	4,60E-02	-	Q27JA2 dirigent-like protein pDIR10 related cluster
GSVIVP00024748001	3,73E-02	-	Q9C523 dirigent protein, putative related cluster
GSVIVP00026851001	3,17E-02	-	Q5XNL4 Resistance protein-like protein related cluster

Probe set ID	*P* value	Diff. expression	Annotation
GSVIVP00008963001	1,39E-02	-	Q9FE98 Alliinase, putative; 28821-30567 related cluster
GSVIVP00006180001	1,20E-02	-	Q6TAF9 Blight Resistance protein SH10 related cluster
GSVIVP00008972001	4,74E-07	-	Q9FE98 Alliinase, putative; 28821-30567 related cluster
GSVIVP00004380001	3,49E-07	-	Q41495 STS14 protein precursor related cluster
Anoxia			
GSVIVP00022724001	1,12E-08	+	Q1I1D6 Hypoxia-responsive family protein related cluster
TC61308	1,16E-02	+	AY159561 Vitis vinifera submergence induced protein 2-like (SIP) mRNA, partial cds.
GSVIVP00009618001	2,18E-02	+	Q96569 L-lactate dehydrogenase related cluster
