# Supplementary material for: Transcriptome changes in grapevine (Vitis vinifera L.) cv. Malbec leaves induced by ultraviolet-B radiation
Source: BMC Plant Biol. 2010 Oct 20;10:224. doi: 10.1186/1471-2229-10-224 (PMC3017828; doi:10.1186/1471-2229-10-224)
Supplement: Additional file 9 — Down-regulated functional classes High UV-B. PDF file showing the full list of differentially expressed genes included in the down-regulated functional categories under high UV-B radiation described in Table1. Positive and negative symbols represent higher or lower transcript levels under UV-B light compared with the control, respectively. [file 1471-2229-10-224-S9.PDF]

Probe set ID	*P* value	Diff. expression	Annotation
Cell cycle			
GSVIVP00030027001	5,50E-08	+	UPI000034F3E8 Cluster related to UPI000034F3E8; catalytic hydrolase
DT030644	1,31E-06	+	UPI00003C004D Cluster related to UPI00003C004D; PREDICTED: similar to centromere protein E
GSVIVP00021372001	7,21E-04	+	Q0WWY1 Regulator of chromosome condensation-like protein related cluster
GSVIVP00007714001	7,62E-04	+	Q40489 Cyclin A-like protein related cluster
GSVIVP00027214001	8,44E-03	+	Q8GZU1 Kinesin related protein related cluster
GSVIVP00024186001	1,36E-02	+	Q1SSB3 Cell division protein FtsZ; peptidase S26A, signal peptidase I related cluster
GSVIVP00018903001	1,71E-02	+	Q94L33 Ania-6a type Cyclin related cluster
GSVIVP00014235001	2,31E-02	+	Q1SW57 HEC Ndc80p related cluster
GSVIVP00011475001	3,59E-02	+	Q9ZRX9 Cyclin D2.1 protein related cluster
GSVIVP00007988001	3,79E-02	+	Q93X47 Cyclin dependent kinase C related cluster
GSVIVP00028583001	4,71E-02	+	Q6Q1P4 Structural maintenance of chromosomes 1 protein related cluster
GSVIVP00019544001	4,80E-02	-	Q8GS71 Kinesin-like protein related cluster
GSVIVP00029039001	4,23E-02	-	Q8GYL6 Regulator of chromosome condensation like related cluster
GSVIVP00034860001	4,01E-02	-	Q6Q4D0 Protein BRUSHY 1 related cluster
TC54578	3,79E-02	-	Q6T2Z2 Cyclin-dependent kinase inhibitor 1;2 related cluster
GSVIVP00019948001	2,81E-02	-	Q6EEW1 Cyclin T1 related cluster
GSVIVP00001024001	2,75E-02	-	Q5VQ09 Putative Kinesin related cluster
GSVIVP00033843001	2,50E-02	-	Q5PXG5 Cyclin dependent kinase inhibitor related cluster
GSVIVP00030704001	2,05E-02	-	Q2QTS8 Regulator of chromosome condensation, putative, expressed related cluster
TC68591	2,05E-02	-	Q9C9L3 Putative regulator of chromosome condensation; 48393-44372 related cluster
GSVIVP00014868001	1,25E-02	-	Q8GS71 Kinesin-like protein related cluster
GSVIVP00012142001	1,10E-02	-	Q8W1Y0 RAD21-3 related cluster
GSVIVP00026604001	1,05E-02	-	Q1SW57 HEC Ndc80p related cluster
GSVIVP00002262001	9,02E-03	-	Q8H6W4 Chromosome condensation regulator protein related cluster
GSVIVP00029328001	9,02E-03	-	O04389 B-type Cyclin related cluster
GSVIVP00014909001	8,44E-03	-	O04388 A-type Cyclin related cluster
GSVIVP00028896001	5,48E-03	-	Q8GVE0 Cyclin D1 related cluster
GSVIVP00026064001	3,94E-03	-	Q9LJ45 PREG1-like negative regulator-like protein related cluster

Probe set ID	*P* value	Diff. expression	Annotation
GSVIVP00001488001	1,61E-03	-	Q8S522 D-type Cyclin related cluster
GSVIVP00022255001	2,41E-03	-	Q1SA17 Pleckstrin-like; Regulator of chromosome condensation beta-lactamase- inhibitor protein II; Zinc finger, FYVE PHD-type related cluster
GSVIVP00025970001	1,73E-03	-	O48626 Centromere kinetochore protein zw10 homolog related cluster
GSVIVP00032526001	1,34E-03	-	Q9XGI4 Cyclin A2 related cluster
GSVIVP00024429001	1,05E-03	-	Q2HU33 Kinesin, motor region related cluster
GSVIVP00015132001	7,21E-04	-	Q6T2Z7 Cyclin d2 related cluster
GSVIVP00027435001	5,32E-04	-	Q1SGR1 Kinesin, motor region; prefoldin related cluster
GSVIVP00016095001	4,24E-04	-	Q8S3S1 Putative Kinesin light chain gene related cluster
GSVIVP00017583001	7,66E-05	-	Q1RSF8 Mis12 related cluster
GSVIVP00028307001	6,13E-05	-	O82002 Putative cullin protein related cluster
GSVIVP00028054001	1,58E-05	-	Q1RV39 Calponin-like Actin-binding; Kinesin, motor region related cluster
GSVIVP00002582001	2,22E-06	-	Q9ZPR1 Cell division control protein 48 homolog B related cluster
GSVIVP00015103001	2,44E-08	-	Q6Z2W0 Chromosome-associated Kinesin-like related cluster
GSVIVP00035100001	2,41E-10	-	Q9LU93 Mitotic spindle checkpoint protein MAD2 related cluster
Blue light signalling			
GSVIVP00001901001	4,63E-02	-	Q9FMF5 Root phototropism protein 3 related cluster
GSVIVP00019637001	1,93E-02	-	Q1SC08 BTB POZ; NPH3 related cluster
GSVIVP00032780001	8,82E-03	-	Q1SI22 BTB POZ; NPH3 related cluster
GSVIVP00026093001	2,04E-04	-	Q7XIJ3 Putative phototropic response protein family related cluster
