# Supplementary material for: Transcriptome changes in grapevine (Vitis vinifera L.) cv. Malbec leaves induced by ultraviolet-B radiation
Source: BMC Plant Biol. 2010 Oct 20;10:224. doi: 10.1186/1471-2229-10-224 (PMC3017828; doi:10.1186/1471-2229-10-224)
Supplement: Additional file 10 — Down-regulated functional classes Low UV-B. PDF file showing the full list of differentially expressed genes included in the down-regulated functional categories under low UV-B radiation described in Table 2. Positive and negative symbols represent higher or lower transcript levels under UV-B light compared with the control, respectively. [file 1471-2229-10-224-S10.PDF]

Probe set ID	*P* value	Diff. expression	Annotation
Cell wall modification			
GSVIVP00033125001	4,34E-10	+	VVU73709 Vitis vinifera beta-1,3-Glucanase mRNA, partial cds.
GSVIVP00025506001	3,14E-03	+	AF305093 Vitis vinifera polygalacturonase inhibitinG protein mRNA, complete cds.
GSVIVP00001464001	5,69E-03	+	Q9LJN4 Beta-1,4-xylosidase related cluster
GSVIVP00028997001	1,48E-02	+	P40973 Pectate lyase precursor related cluster
GSVIVP00028094001	1,57E-02	+	Q1SDB4 Putative beta-d-xylosidase related cluster
GSVIVP00029166001	3,53E-02	+	Q9MBC0 polygalacturonase related cluster
GSVIVP00024648001	4,68E-02	+	Q8GTD6 polygalacturonase inhibitor-like protein related cluster
TC67357	3,79E-02	-	Q6T5H5 Alpha-expansin 3 related cluster
GSVIVP00000596001	3,60E-02	-	O48818 expansin-A4 precursor related cluster
GSVIVP00002441001	2,89E-02	-	Q9SUP5 Putative polygalacturonase related cluster
GSVIVP00025938001	2,84E-02	-	Q4F8J0 Putative endo-1,4-beta-Glucanase related cluster
VVTU20744_at	2,36E-02	-	Q9LUB8 polygalacturonase related cluster
GSVIVP00019299001	2,18E-02	-	Q1SJD6 Galactose-binding like related cluster
GSVIVP00038386001	1,73E-02	-	Q2MCJ5 Xylan 1,4-beta-xylosidase related cluster
GSVIVP00000495001	1,66E-02	-	Q8L868 Putative glucan endo-1,3-beta-glucosidase 11 precursor (EC 3.2.1.39) ((1->3)-beta-glucan endohydrolase 11) ((1->3)-beta-Glucanase 11) related cluster
GSVIVP00002554001	1,13E-02	-	Q84LI7 Polygalacturonase-like protein related cluster
GSVIVP00025336001	9,78E-03	-	Q9M3U4 Beta 1-3 Glucanase related cluster
GSVIVP00029885001	9,18E-03	-	Q2VT54 DC1.2-like related cluster
GSVIVP00032045001	6,79E-03	-	Q9FT02 (1-4)-beta-mannan endohydrolase related cluster
GSVIVP00029887001	4,65E-03	-	Q2VT54 DC1.2-like related cluster
GSVIVP00020458001	4,05E-03	-	AY043234 Vitis vinifera putative Pectate lyase mRNA, partial cds.
TC67367	2,60E-03	-	Q9M4H8 Putative Ripening-related protein related cluster
GSVIVP00020459001	1,94E-03	-	AY043234 Vitis vinifera putative Pectate lyase mRNA, partial cds.
GSVIVP00024306001	1,80E-03	-	Q7X9Q3 expansin related cluster
GSVIVP00018131001	1,76E-03	-	Q27U75 Pectate lyase related cluster
GSVIVP00030705001	6,47E-04	-	Q93XJ1 Pectate lyase related cluster
GSVIVP00029445001	1,20E-04	-	Q84US9 expansin related cluster
GSVIVP00029891001	6,65E-07	-	Q2VT54 DC1.2-like related cluster

Probe set ID	*P* value	Diff. expression	Annotation
GSVIVP00020991001	5,24E-08	-	Q8L5J6 expansin 3 related cluster
GSVIVP00015920001	5,99E-12	-	Q4JLV6 Pectate lyase related cluster
Auxin			
GSVIVP00016808001	4,34E-10	+	Q8L6S5 IAA16 protein related cluster
TC55783	9,60E-04	+	Q1S379 auxin responsive SAUR protein related cluster
GSVIVP00026137001	3,23E-02	-	Q1SHH7 auxin responsive SAUR protein related cluster
GSVIVP00036093001	2,31E-02	-	Q8L883 auxin transporter-like protein 5 related cluster
TC63866	1,94E-02	-	Q9XED8 auxin response factor 9 related cluster
GSVIVP00024505001	1,70E-02	-	Q2LAI9 auxin response factor 4 related cluster
GSVIVP00035997001	1,57E-02	-	Q9SW54 Hypothetical protein T11I11.40 related cluster
GSVIVP00015336001	9,99E-03	-	Q9FEL6 auxin transporter-like protein 3 related cluster
GSVIVP00035995001	7,41E-03	-	Q9SW54 Hypothetical protein T11I11.40 related cluster
GSVIVP00036015001	6,94E-03	-	Q9SW57 Hypothetical protein T11I11.10 related cluster
GSVIVP00032666001	1,94E-03	-	Q1S2K0 Aux IAA protein related cluster
GSVIVP00035985001	2,62E-04	-	Q94B76 Putative auxin-regulated protein related cluster
Stress miscellaneous			
GSVIVP00030524001	2,03E-07	+	Q9AXU0 Major latex-like protein related cluster
CB349093	5,13E-05	+	Q42428 Chitinase Ib related cluster
CA809580	6,50E-03	+	Q6L3K5 Senescence-associated protein, putative related cluster
GSVIVP00018061001	2,18E-02	+	Q64EX4 MtN19-like protein related cluster
GSVIVP00036222001	2,27E-02	+	Z54234 V.vinifera mRNA for Chitinase.
TC70718	4,92E-02	-	Q0W9E8 Putative stress-induced protein related cluster
CN548040	4,52E-02	-	Q67WQ6 Putative Senescence-associated protein 5 related cluster
GSVIVP00016581001	4,14E-02	-	Q9ZUN5 Putative Senescence-associated protein 5 related cluster
GSVIVP00022959001	3,79E-02	-	Q0W9E8 Putative stress-induced protein related cluster
DT039600	2,45E-02	-	Q8LBL4 Putative Thaumatin-like protein related cluster
GSVIVP00014281001	1,11E-02	-	Q700J9 Putative Pathogenesis-related protein related cluster
GSVIVP00031449001	1,02E-02	-	Q1SZF1 Allergen V5 Tpx-1 related related cluster
GSVIVP00028590001	6,08E-03	-	Q8GZP6 Allergen Ana o 2 related cluster

Probe set ID	*P* value	Diff. expression	Annotation
GSVIVP00024719001	5,32E-03	-	Q1SRM5 PAP fibrillin related cluster
GSVIVP00030518001	5,32E-03	-	Q9M4G9 Putative Ripening-related protein related cluster
GSVIVP00031447001	3,37E-03	-	Q40374 Pathogenesis-related protein PR-1 precursor related cluster
CB348560	2,19E-03	-	Q7Y238 Hevein-like antimicrobial peptide related cluster
GSVIVP00036771001	2,04E-03	-	Q8LBL4 Putative Thaumatin-like protein related cluster
GSVIVP00033575001	2,20E-08	-	Q6JX04 Chitinase-like protein related cluster
